# High-Entropy Alloy Nanocrystals: From Synthesis to Characterization and Catalytic Application

**DOI:** 10.1021/acs.chemrev.6c00030

**Published:** 2026-05-18

**Authors:** Jianlong He, Yueh-Chun Hsiao, Chia-Ying Wu, Younan Xia, Tung-Han Yang

**Affiliations:** † School of Chemistry and Biochemistry, 1372 Georgia Institute of Technology, Atlanta, Georgia 30332, United States; ‡ Department of Chemical Engineering, 34881 National Tsing Hua University, Hsinchu 300044, Taiwan; § The Wallace H. Coulter Department of Biomedical Engineering, Georgia Institute of Technology and Emory University, Atlanta, Georgia 30332, United States; ∥ Department of Materials Science and Engineering, Department of Biomedical Engineering, 1466 Johns Hopkins University, Baltimore, Maryland 21218, United States; ⊥ College of Semiconductor Research, National Tsing Hua University, Hsinchu 300044, Taiwan; ¶ High Entropy Materials Center, National Tsing Hua University, Hsinchu 300044, Taiwan

## Abstract

High-entropy alloy (HEA) nanocrystals, characterized by four core effects, including high entropy, lattice distortion, sluggish diffusion, and cocktail mixing, represent a transformative class of catalytic materials that combine compositional diversity with nanoscale complexity. By combining five or more elements into single-phase solid solutions, HEA nanocrystals offer unique opportunities to tailor local atomic environments, electronic structures, and catalytic properties. This Review offers a comprehensive overview of recent advances in the syntheses, compositional and structural controls, and catalytic applications of these materials, with a focus on achieving uniform atomic mixing and well-defined surface structures. We start with key synthetic methodologies, including both top-down and bottom-up approaches, and highlight wet-chemical synthesis that allows for precise regulation of reduction, nucleation, growth, and alloying dynamics to overcome challenges such as reduction kinetics, elemental immiscibility, and crystallographic incompatibility. We then discuss advanced characterization techniques and theoretical modeling approaches used to probe the multicomponent solid-solution phases, local coordination environments, interelement interactions, and synergistic effects that collectively govern the catalytic behaviors of HEA nanocrystals. We further highlight how these fundamental features manifest at the nanoscale to enhance performance across diverse catalytic reactions, including electro-, thermo-, and photocatalysis. Finally, we identify 15 critical aspects that provide a roadmap for the rational design of next-generation HEA catalysts for sustainable energy conversions and chemical transformations.

## Graphical abstract

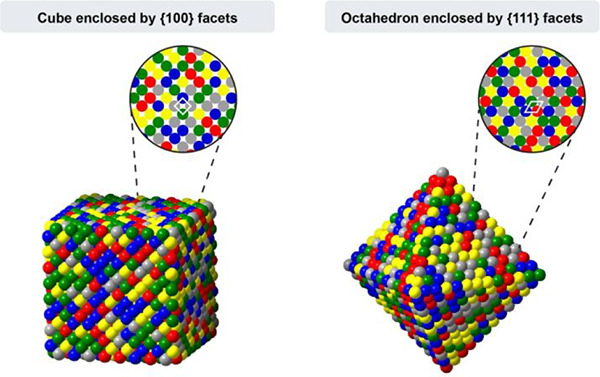

## 1 Introduction

From the very beginning of human civilization, the discovery and utilization of new metals and their combinations have kept driving technological progress and shaping modern society. Since the concept of high-entropy alloys (HEAs) was first proposed in 2004, multicomponent alloys containing at least five principal elements in near-equiatomic ratios have rapidly emerged as one of the most dynamic fields in materials science and engineering.^1^ In this context, it is helpful to distinguish HEAs from a broader class of materials known as multimetallic alloys. Multimetallic alloys are an umbrella category encompassing any alloy comprised of three or more metallic elements, ranging from trimetallic to higher order systems and including both disordered solid solutions and ordered intermetallic compounds over a wide range of compositions. By contrast, HEAs represent a specific subset of multimetallic alloys that emphasize multiple principal elements in comparable fractions, for which the contribution from configurational entropy becomes sufficiently significant to thermodynamically bias the system toward disordered solid solution stabilization rather than compound formation, although multiphase microstructures may also occur depending on composition and processing.^2,3^

One can define HEAs from either a compositional or a thermodynamic perspective.^1^ From the compositional viewpoint, HEA is typically defined as a metallic system comprising five or more principal elements, each present in an atomic fraction of approximately 5–35 at. %, and it forms a single-phase solid solution rather than an intermetallic compound. From the thermodynamic viewpoint, the degree of atomic disorder can be quantified using the configurational entropy of mixing (Δ*S*_*mix*_):

1 $$\Delta S_{mix}=-R\overset{n}{\underset{i=1}{\sum }}x_{i}\ln x_{i}$$

where *R* is the ideal gas constant, *x*_*i*_ is the molar fraction of the i^th^ element, and *n* is the total number of constituent elements. According to this definition, alloys can be categorized as low-entropy alloys (Δ*S*_*mix*_ < 1 *R*), medium-entropy alloys (1 *R* ≤ Δ*S*_*mix*_ ≤ 1.5 *R*), and high-entropy alloys (Δ*S*_*mix*_ > 1.*5 R*). For an equimolar quinary alloy, the calculated Δ*S*_*mix*_ is approximately 1.61 *R*, surpassing the commonly accepted threshold for high-entropy alloy classification. High configurational entropy stabilizes single-phase solid solutions by lowering the Gibbs free energy through the *T*Δ*S*_*mix*_ term, suppressing phase separation and intermetallic formation.^4–6^ Early research on HEAs mainly focused on bulk materials and thin films, aiming to understand their phase stability, mechanical strength, and thermal properties.^5,7–9^ These investigations revealed that HEAs could form simple solid-solution phases with exceptional hardness, high-temperature stability, and corrosion resistance, challenging the conventional alloy-design paradigm based on one dominant host element. These discoveries not only expand the compositional space of alloys but also introduce a new framework for exploring the relationships between entropy, atomic configuration, and macroscopic functionality. As of October 2025, over 20,000 papers related to HEAs have been published, according to the Web of Science database, highlighting the remarkable and accelerating development of this field.

At the fundamental level, the distinctive physicochemical properties of HEAs originate from four principal effects arising from high entropy, lattice distortion, sluggish diffusion, and cocktail mixing (Figure 1).^1,10–12^ Among them, the high-entropy effect serves as the defining concept and thermodynamic foundation of HEAs. In equimolar multicomponent systems, configurational entropy of mixing increases with the number of components and can contribute to lowering the free energy of a homogeneous solid-solution phase relative to more ordered or less uniformly mixed competing states, such as ordered intermetallics or phase-separated structures. However, the stability of a single-phase HEA solid solution should not be attributed to configurational entropy alone, but rather to the balance between enthalpic (Δ*H*) and entropic contributions (*T*Δ*S*_*mix*_), as described by the Gibbs free energy relationship (Δ*G* = Δ*H*–*T*Δ*S*_*mix*_).

**Figure 1:**
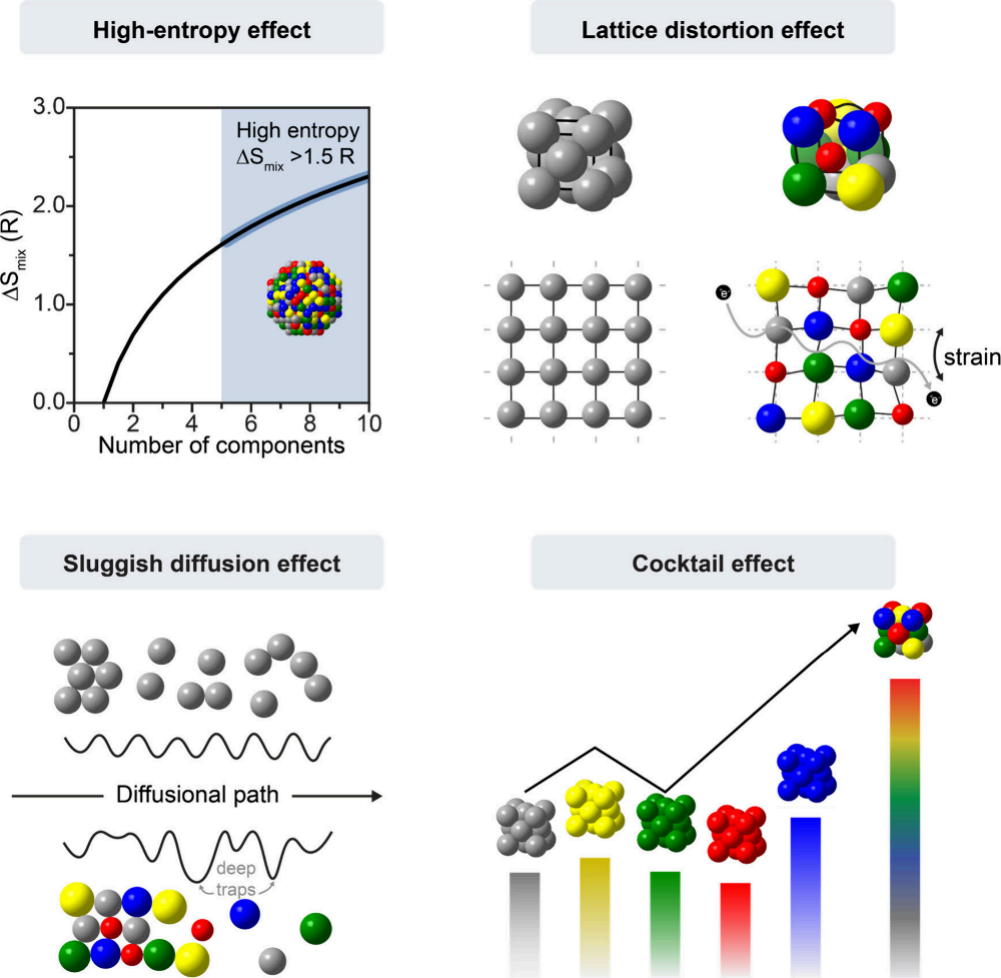
Schematic illustration of the four principal effects of HEAs enabled by high entropy, lattice distortion, sluggish diffusion, and cocktail mixing.

The mixing enthalpy is not necessarily strongly negative, but depends on the elemental combinations and their competing interactions, such as atomic size mismatch, lattice distortion, and unfavorable interactions between specific elemental pairs. Since single-phase solid solutions are often found within an empirical mixing-enthalpy window of approximately −15 to +5 kJ mol^–1^,^13^ configurational entropy can play an important role in lowering the Gibbs free energy and thereby stabilizing the solid-solution phase. This entropic stabilization effectively suppresses phase segregation and promotes the formation of homogeneous solid-solution phases instead of ordered intermetallic compounds.^4–6^ In addition to this thermodynamic stabilization, the other three effects further enhance and enrich the structure and functionality of HEAs. The lattice distortion effect arises from the incorporation of elements with different atomic sizes and electronegativities, causing deviations of constituent atoms from their ideal lattice positions and thus generating local strain fields. This structural irregularity modifies the electronic structure, strengthens atomic bonding, and enhances mechanical robustness.^14^ The sluggish diffusion effect refers to the significantly reduced atomic mobility in HEAs compared with conventional alloys, which is attributed to lattice distortion and compositional complexity.^15,16^ This slow diffusion kinetically suppresses phase segregation and promotes the retention of metastable and amorphous structures, contributing to improved thermal and electrochemical stability. Finally, the cocktail effect captures the synergistic and often unpredictable properties arising from the coexistence of multiple elements.^17^ The collective interactions among different atoms give rise to emergent behaviors that cannot be predicted from the individual components alone. Together, these four effects govern the thermodynamic stability, structural flexibility, and functional tunability of HEAs across different length scales.

Building upon these fundamental understandings, recent efforts have extended the concept of high-entropy design from bulk solids and thin films to the nanoscale.^18–23^ Incorporating multiple elements into a single nanocrystal not only preserves the entropic stabilization characteristic of HEAs but also introduces unprecedented opportunities to manipulate the surface composition, atomic arrangement, electronic structure, and distribution of active sites. As a result, HEA nanocrystals have emerged as a new frontier in catalysis, bridging the gap between bulk solids and individual atoms.^24–26^ Downsizing HEAs to the nanoscale provides immense scientific and technological potential, as it transforms a thermodynamically stable solid-solution concept into a structurally and electronically tunable catalytic platform. Moreover, HEA nanocrystals possess a distinctive combination of compositional tunability and structural complexity that makes them fundamentally different from conventional mono- or bimetallic nanocatalysts. By integrating multiple elements within a single nanoscale lattice, they generate a vast number of local atomic configurations and electronic environments, creating an enormous ensemble of active sites with tunable adsorption characteristics. This configurational richness provides a powerful platform for optimizing catalytic activity, selectivity, and durability through a rational manipulation of composition, surface structure, and electronic coupling.^27,28^ However, it remains a major challenge to synthesize such multicomponent nanocrystals, as successful synthesis requires precise controls over elemental distribution, atomic arrangement, phase uniformity, and particle morphology. Methodology development is expected to establish a library of systematic nanoscale alloys, opening the door to exceptional catalytic performance.

With recent synthetic advances, increasing attention has turned toward understanding how compositional complexity and nanoscale effects influence the intrinsic physicochemical properties of HEAs.^29,30^ The unique thermodynamic and kinetic behaviors of HEA nanocrystals, governed by their compositional complexity and size effect, render the four principal effects more pronounced and catalytically more favorable compared to their bulk counterparts. For example, the high-entropy effect stabilizes single-phase multimetallic structures and prevents surface segregation or dealloying under reaction conditions, ensuring a homogeneous and stable distribution of active sites.^31^ The lattice distortion effect can introduce a distribution of local atomic environments and adsorption energies, which may modulate the adsorption free energies of critical intermediates such as H*, OH*, CO*, OOH*, and N*.^32^ This effect may be advantageous for certain reactions, such as the hydrogen evolution reaction (HER), where different sites can facilitate different elementary steps.^33^ The effect, however, is not universally beneficial, as the reactions that require well-defined and uniform active sites to minimize activation barriers and maintain high selectivity may be negatively affected by excessive site-to-site variability. The sluggish diffusion effect suppresses atomic migration and sintering during a catalytic process, enabling exceptional thermal and electrochemical stability.^34^ Finally, the cocktail mixing effect induces synergistic interactions among constituent elements, enabling cooperative reaction pathways that are unattainable in simpler alloy systems.^35^ Collectively, these intertwined effects endow HEA nanocrystals with a highly flexible catalytic landscape capable of overcoming the conventional activity–stability trade-off that constrains most heterogeneous catalysts. By tailoring the elemental composition and crystal facets, it becomes feasible to engineer HEA nanocrystals with electronic and structural features that are intrinsically optimal toward electro-, thermo-, and photocatalytic reactions, including HER and hydrogen oxidation reaction (HOR),^33,36–40^ oxygen evolution and reduction (OER and ORR),^41–46^ carbon dioxide reduction (CO_2_RR),^47–49^ methanol and alcohol oxidation (MOR and AOR),^50–54^ as well as ammonia production and decomposition.^55–57^ Thus, HEA nanocrystals represent not merely an extension of multimetallic catalysis but a transformative materials platform that unites compositional complexity with nanoscale precision to achieve tunable and durable catalytic performance. As of now, a Web of Science search using the keywords “*high-entropy alloy nanoparticles*” and “*high-entropy alloy nanocrystals*” yields over 1,440 publications, reflecting a growing yet still emerging interest in this dynamic research arena.

In pursuit of synthetic controls, recent progress in HEA nanocrystals has been inspired by more than three decades of research on colloidal metal nanocrystals, especially monometallic and bimetallic systems.^58–60^ Colloidal nanocrystals composed of noble or transition metals and their alloys, such as Fe, Co, Ni, Cu, Ru, Rh, Pd, Ag, Ir, Pt, and Au, have garnered significant attention due to their unique physicochemical properties and diverse applications in catalysis,^61^ energy conversion,^62^ photonics,^63^ electronics,^64^ sensing,^65^ and biomedicine.^66^ It is well established that the properties of a metal nanocrystal are governed by a set of key parameters, including composition, shape, size, crystal structure, and phase state (e.g., solid solution, phase-separated, or intermetallic), and each of which can be independently tuned to optimize their performance in a specific application.^67,68^ Among these parameters, composition and shape have been most extensively explored owing to their strong correlations with catalytic performance. For example, variations in composition result in distinct electronic structures and adsorption characteristics to suit different catalytic reactions. In the case of HER, platinum-group metals (PGMs) such as Ru, Rh, Pd, Ir, and Pt lie near the apex of the volcano plot, where the hydrogen adsorption free energy (Δ*G*_H*_) approaches zero, yielding an ideal balance between adsorption and desorption and thus the highest catalytic activity. By contrast, most non-noble metals either bind hydrogen too strongly (e.g., Fe, Co, Ni, Mo, and W) or too weakly (e.g., Al, Cu, and Bi), resulting in compromised activity.^69,70^ In addition to catalytic activity, composition can also influence selectivity. For example, in Cu-based bimetallic catalysts toward CO_2_RR, the identity of the second metal can markedly alter the product distribution, with Cu–Ag, Cu–Au, and Cu–Zn systems often enhancing CO formation and, in some cases, promoting multicarbon products, whereas Cu–In and Cu–Sn systems tend to suppress HER and favor HCOO^–^ formation.^71,72^ The Cu–Pd system can also exhibit distinct selectivity depending on the degree of mixing or phase separation,^73^ further highlighting that not only the overall composition but also the spatial distribution of the constituent elements can influence reaction pathways relative to the parental metals. In addition to the compositional effect, catalytic activity and selectivity can also be tailored by manipulating the shape of nanocrystals to control the atomic arrangement on their surface. Taking ORR as an example, the specific activity of Pt octahedra enclosed by {111} facets was reported to be greater than that of Pt cubes enclosed by {100} facets.^74,75^ This difference arises primarily from variations in surface atomic symmetry: {100} facets exhibit a square atomic arrangement, whereas {111} facets possess a hexagonal arrangement, leading to distinct adsorption strengths, reaction barriers, and desorption behaviors of reaction intermediates and products. Similar correlations between catalytic performance and surface structures have also been observed for many other structure-sensitive reactions, highlighting the critical importance of shape control in tailoring the surface chemistry of metal nanocrystals and thereby optimizing their catalytic performance.^76^

Here, we aim to provide a comprehensive overview of recent advances in the synthesis, characterization, and utilization of HEA nanocrystals with controllable compositions and facets. In this Review, we deliberately use the term “HEA nanocrystals” rather than the more general “HEA nanoparticles” to emphasize a crystalline lattice with resolvable crystallographic order. Accordingly, we focus on crystallographically defined HEA systems and discuss how crystal structure, phase, lattice strain and distortion, defects, and facet-dependent atomic arrangements govern catalytic behavior and enable quantitative structure–composition–property relationships that are difficult to extract from ill-defined nanoparticle ensembles. Emphasis is placed on the synthetic challenges, underlying formation mechanisms, and catalytic behaviors revealed through the combined insights of experimental observations and theoretical modeling. We summarize representative synthetic methodologies encompassing both top-down and bottom-up approaches, with special focus on wet-chemical colloidal synthesis that allows precise regulation of reduction, nucleation, growth, and alloying dynamics in the solution phase. These methods have proven effective for producing HEA nanocrystals with uniform atomic mixing and well-defined surface atomic arrangements. Such materials can serve as model systems for elucidating atomic-level correlations between composition, structure, and catalytic properties. In addition, we discuss how these fundamental effects manifest uniquely at the nanoscale to enhance catalytic performance across diverse reactions, including electro-, thermo-, and photocatalysis. Finally, we identify 15 critical aspects that highlight the need for synergistically integrating synthetic controls, operando characterizations, and predictive theoretical modeling to unlock the full potential of HEA-based nanocatalysts. Attaining uniform atomic mixing, tuning surface strain, and modulating oxidation states, crystal phases, and reaction energetics are indispensable for developing a comprehensive understanding of how compositional complexity dictates catalytic behavior. Through this Review, we aim to establish a conceptual and methodological foundation for the rational synthesis of HEA nanocrystals and to stimulate future developments of high-performance, long-lasting catalysts for sustainable energy conversion and chemical transformation.

## 2 Challenges in Working with HEA Nanocrystals

Despite significant advances in the synthesis of multimetallic nanocrystals, achieving precise controls over the composition and shape of HEA nanocrystals containing five or more principal elements remains a formidable challenge. HEA formation involves simultaneous interactions among multiple elements with distinct chemical potentials, reduction kinetics, atomic sizes, and preferred crystal structures. As a result, achieving atomic-level mixing and regulating surface morphology requires one to balance a complex set of thermodynamic and kinetic factors far exceeding those involved in mono- or bimetallic systems.^77,78^ Table 1 summarizes representative metals together with selected metal-ion/metal redox couples, along with key physicochemical parameters such as standard reduction potential, atomic radius, electronegativity, enthalpy of formation, bond dissociation energy, surface free energies, and preferred crystal structure. These parameters collectively determine the reduction sequence, diffusion behavior, and thermodynamic stability of multicomponent alloys. Large disparities in elemental properties, combined with inherent immiscibility, often lead to limited mutual solubility and localized compositional segregation during synthesis. Such thermodynamic incompatibility results in compositional heterogeneity, phase separation, or partial ordering, preventing the formation of homogeneous solid-solution HEAs.^79^

**Table 1:** Representative Physicochemical Parameters of the Metals Commonly Used in the Synthesis of HEA Nanocrystals

Metal	Standard reduction potential^a^ (V vs SHE)		Metallic radius^b^ (pm)	Electronegativity^c^ (eV^–1/2^, Pauling scale)	Enthalpy of Formation^d^ (kJ mol^–1^)	Bond dissociation^d^ (kJ mol^–1^)	Surface free energy^e^ (J m^–2^)	Crystal lattice
Al	–1.66	(Al^3+^ + 3e^–^→ Al)	125	1.61	331 ± 4.0	264 ± 0.5	1.20/1.35/1.27	FCC
Fe	–0.04	(Fe^3+^ + 3e^–^→ Fe)	117	1.83	416 ± 1.3	118	2.43/2.22/2.59	BCC
Co	–0.28	(Co^2+^ + 2e^–^→ Co)	116	1.88	427	<127	2.78/3.04/3.79	HCP
Ni	–0.26	(Ni^2+^ + 2e^–^→ Ni)	115	1.91	430 ± 8.4	204	2.01/2.43/2.37	FCC
Cu	0.34	(Cu^2+^ + 2e^–^→ Cu)	117	1.90	337 ± 1.2	201	1.95/2.17/2.24	FCC
Mo	–0.20	(Mo^3+^ + 3e^–^→ Mo)	129	2.16	659 ± 3.8	436 ± 1.0	3.45/3.84/3.60	BCC
Ru	0.46	(Ru^2+^ + 2e^–^→ Ru)	124	2.20	651 ± 6.3	193 ± 19.3	3.93/4.24/4.86	HCP
Rh	0.76	(Rh^3+^ + 3e^–^→ Rh)	125	2.28	556 ± 4	236 ± 0.05	2.47/2.80/2.90	FCC
Pd	0.95	(Pd^2+^ + 2e^–^→ Pd)	128	2.20	377 ± 2.1	>136	1.92/2.33/2.23	FCC
Ag	0.80	(Ag^+^ + e^–^→ Ag)	134	1.93	285 ± 0.8	163 ± 2.9	1.17/1.20/1.24	FCC
W	0.10	(W^3+^ + 3e^–^ → W)	130	2.36	851 ± 6.3	666	4.00/4.64/4.18	BCC
Ir	1.16	(Ir^3+^ + 3e^–^→ Ir)	126	2.20	669 ± 4	361 ± 68	2.97/3.72/3.61	FCC
Pt	1.18	(Pt^2+^ + 2e^–^→ Pt)	129	2.28	566 ± 1.3	307 ± 1.9	2.30/2.73/2.82	FCC
Au	1.50	(Au^3+^ + 3e^–^→ Au)	134	2.54	368 ± 2.1	226 ± 0.5	1.28/1.63/1.70	FCC
Bi	0.31	(Bi^3+^ + 3e^–^→ Bi)	152	2.02	210 ± 2.1	204	0.54/0.54	Rhombohedral

a Standard reduction potentials measured at 25 °C and 1 atm. SHE: standard hydrogen electrode.

b Values taken from ref 85.

c Electronegativity values on the Pauling scale, taken from the supplementary of ref 86.

d Values taken from ref 87.

e Values taken from ref 88. Theoretical surface free energies derived from the full charge density (FCD) method. Values are listed in the order of the most stable crystallographic surfaces for each structure: FCC: (111), (100), (110); HCP: (0001), (1010)_A_, (1010)_B_; BCC: (110), (100), (211). Bi adopts a rhombohedral crystal structure derived from a distorted simple cubic lattice.

The intrinsic disparities also pose significant challenges to shape control. The coexistence of elements with differing surface energies and crystallographic preferences disrupts regular atomic arrangements during crystal growth, leading to lattice strain, surface reconstruction, and morphological distortion. As a result, maintaining both random atomic mixing and well-defined symmetric surface structures becomes extremely difficult. Figure 2 shows schematics of two examples of facet-controlled solid-solution HEA nanocrystals in cubic and octahedral shapes, respectively. The HEA cube is enclosed by {100} facets, which exhibit square atomic arrangements, whereas the octahedron is enclosed by {111} facets with hexagonal atomic arrangements.^80–82^ Stabilizing such specific low-index facets while simultaneously achieving uniform atomic mixing requires precise control over reduction kinetics and surface energy balance. Moreover, it is critical to suppress structural relaxation that would otherwise drive the particles toward an isotropic or thermodynamically preferred shape.

**Figure 2:**
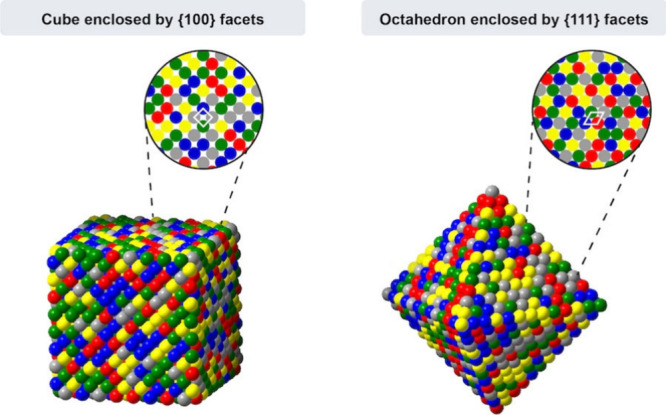
Schematic illustration of two representative HEA nanocrystals in the cubic and octahedral shapes, featuring uniform atomic mixing and distinct {100} and {111} facets, respectively.

Beyond synthesis, characterizing HEA nanocrystals is equally important in correlating the structural features with their exceptional performance. However, this task remains highly challenging because of the compositional complexity. A wide range of commonly used analytical techniques, including transmission electron microscopy (TEM), energy-dispersive X-ray spectroscopy (EDS), X-ray photoelectron spectroscopy (XPS), and X-ray absorption spectroscopy (XAS), have been employed to investigate the elemental distribution and structural characteristics of HEAs. Although these techniques are instrumental in providing valuable information about composition, structure, and chemical states, they also have inherent limitations. The information obtained is often incomplete or ensemble-averaged, and distinguishing elements with similar atomic numbers or overlapping spectral features remains difficult to resolve.^83,84^ Such constraints obscure the intrinsic heterogeneity of HEA systems and make it challenging to establish direct correlations between atomic arrangements and their performance, which remains one of the central bottlenecks in this rapidly evolving field.

Understanding these intertwined challenges in synthesis and characterization is essential to the rational design and optimization of HEA nanocrystals. Reduction kinetics, elemental immiscibility, and interfacial energetics collectively govern atomic mixing and morphological evolution, while advanced characterization is required to resolve atomic configurations and validate synthetic control. The following sections, therefore, focus on three key aspects: *i*) controlling composition and atomic mixing, *ii*) controlling shape and surface structure, and *iii*) characterizing HEA nanocrystals at the atomic level. Together, these discussions highlight the thermodynamic and kinetic origins of the difficulties in achieving precise controls and detailed understandings of HEA nanocrystals.

### 2.1 Controlling the Composition and Atomic Mixing of HEA Nanocrystals

Among the various challenges in synthesizing HEA nanocrystals, achieving homogeneous atomic mixing represents the most fundamental yet difficult objective. The simultaneous reduction of multiple metal precursors with distinct redox potentials and chemical affinities (Table 1) often leads to asynchronous nucleation and sequential deposition, producing compositional gradients or core–shell structures instead of uniform solid solutions. A representative example is the synthesis of PdPtRhIrRu HEA nanocrystals, one of the most widely studied PGM-based systems.^78^ When a conventional one-pot polyol synthesis was carried out at 180 °C using ethylene glycol (EG) and l-ascorbic acid (AA) as the reductants and poly­(vinylpyrrolidone) (PVP) as the stabilizer, the reduction rate constants of Pd­(II), Pt­(II), Rh­(III), Ir­(IV), and Ru­(III) precursors followed a descending order of 6.50 × 10^–2^, 3.18 × 10^–2^, 5.29 × 10^–3^, 2.28 × 10^–3^, and 1.04 × 10^–3^ s^–1^, respectively. Nearly all Pd­(II) ions were reduced within the first minute, while Ir­(IV) and Ru­(III) had not yet been reduced (Figure 3B). The resultant nanocrystals exhibited a Pd-rich core surrounded by an outer region enriched in Pt, Rh, Ir, and Ru, as revealed by high-angle annular dark-field scanning transmission electron microscopy (HAADF-STEM) and EDS mapping (Figure 3C–E), directly reflecting the kinetic and thermodynamic mismatch among the precursors.

**Figure 3:**
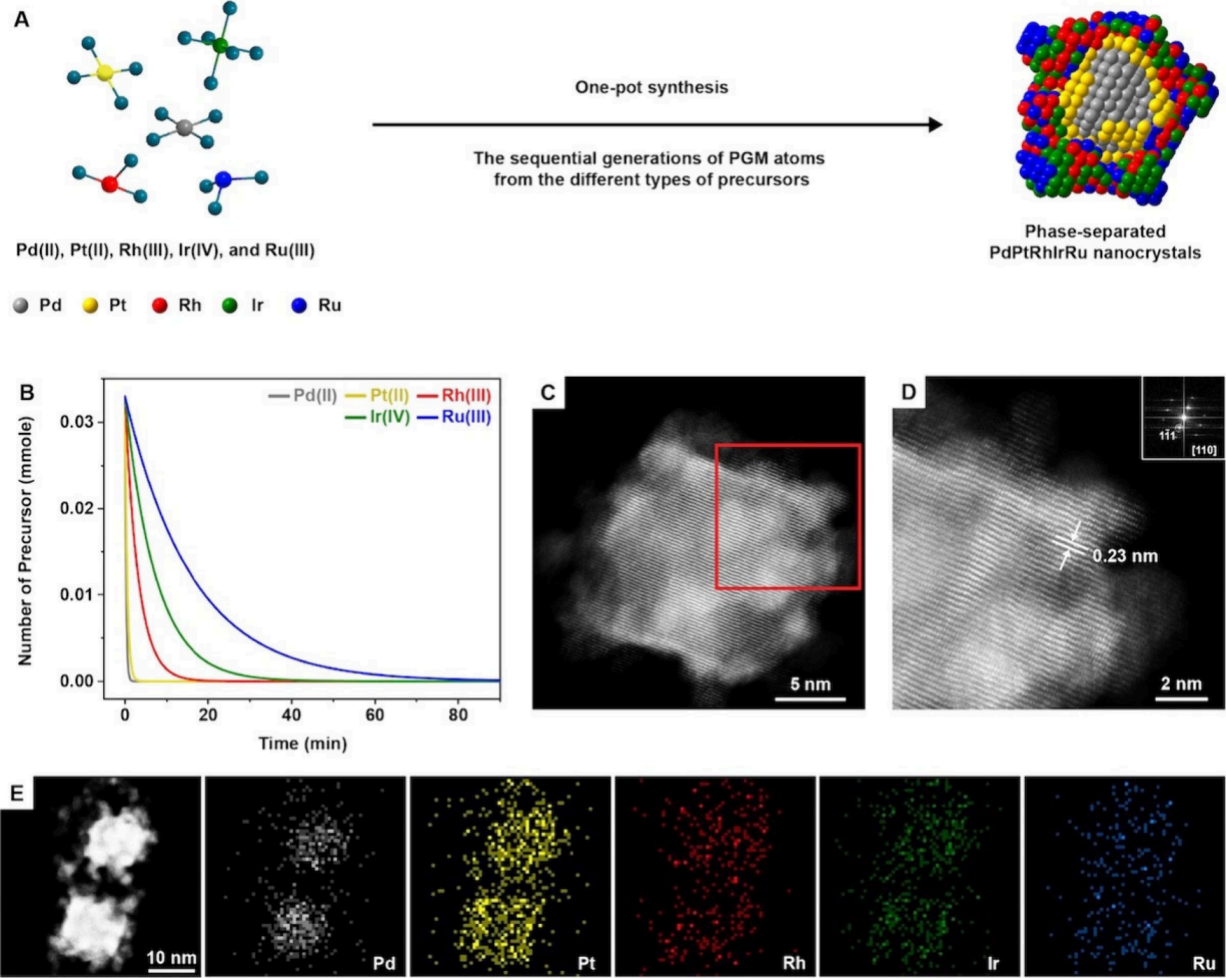
Kinetic disparity and phase-separated structure in the one-pot synthesis of PdPtRhIrRu multicomponent nanocrystals. (A) Schematic illustration of the one-pot synthesis leading to phase-separated nanocrystals. (B) Simulated concentration profiles of Pd­(II), Pt­(II), Rh­(III), Ir­(IV), and Ru­(III) precursors during the reduction process. (C) HAADF-STEM image of a representative phase-separated nanocrystal. (D) Atomic-resolution HAADF-STEM image taken from the marked region in (C). (E) EDS elemental maps of Pd, Pt, Rh, Ir, and Ru for the phase-separated nanocrystals. Reproduced with permission from ref 78. Copyright 2023 AAAS.

This challenge becomes even more severe when noble metals are combined with earth-abundant transition metals to construct multicomponent HEA nanocrystals. The alloying of PGMs with iron-group metals (IGMs; Fe, Co, and Ni) to form IGM–PGM HEA nanocrystals provides a representative example.^81^ The substantial difference in reduction potentials between PGMs and IGMs creates a large imbalance in thermodynamic driving forces. In conventional polyol systems, the temperatures adequate for reducing PGM ions (typically below 200 °C) are inadequate for the complete reduction of IGM precursors such as Fe­(III), Co­(II), and Ni­(II). Elevating the temperature to enhance reduction kinetics often exacerbates the disparity in elemental reactivity, leading to nonsimultaneous nucleation and inadequate alloying. To mitigate this issue, oleylamine-based systems have been developed to promote the thermal decomposition of acetylacetonate organometallic precursors such as Fe­(acac)_3_, Co­(acac)_2_, and Ni­(acac)_2_ under moderate heating conditions. This decomposition-driven pathway enables concurrent reduction and alloying of PGM and IGM components, facilitating the formation of single-phase HEA nanocrystals with homogeneous atomic mixing. Even in gas-phase reduction environments, similar challenges persist. For instance, in the hydrogen reduction synthesis of CoNiCuRuPd HEA nanocrystals on TiO_2_ supports, Ru­(III) and Pd­(II) species are readily reduced below 150 °C, whereas Co­(II), Ni­(II), and Cu­(II) require temperatures exceeding 200 °C.^89^ Achieving uniform alloying thus depends on subsequent autocatalytic reduction and interdiffusion among the metallic species during prolonged annealing. These examples collectively highlight that overcoming reduction-potential disparities is a prerequisite for obtaining homogeneous solid-solution HEA nanocrystals and that the choice of reductant, solvent, and temperature must be delicately balanced to synchronize the reduction kinetics of different precursors.

Although diverse synthetic strategies have been developed for preparing HEA nanocrystals, the degree of atomic-level mixing can vary substantially, leading to distinct catalytic activity and selectivity. Such compositional heterogeneity primarily arises from intrinsic thermodynamic and crystallographic incompatibilities among constituent elements.^74^ Differences in preferred crystal structures (e.g., FCC, HCP, or BCC), interatomic bonding energies, and mixing enthalpies strongly affect how atoms distribute during nucleation and growth. When certain elemental pairs exhibit positive Δ*H* values or unfavorable bonding interactions, local segregation becomes thermodynamically favored over random solid-solution formation, resulting in short-range ordering or phase separation. According to the Gibbs free energy relation (Δ*G* = Δ*H* – *T*Δ*S*), the formation of a homogeneous solid solution depends on the balance between Δ*H* and the configurational entropy, with Δ*S* serving as the main entropy contributor. Large positive Δ*H* values (e.g., Au–Pt, Au–Ni) promote phase immiscibility, whereas highly negative values (e.g., Pt–Ni, Pt–Sn) favor ordered intermetallic formation. Ideally, a true HEA solid solution forms only when Δ*H* among all elemental pairs approaches zero, allowing the entropy term to dominate.^90,91^ However, this condition is rarely achieved due to disparities in atomic size, bonding strength, and electronegativity, which often result in compositional segregation. To overcome these limitations, effective synthetic strategies often rely on kinetic control to suppress atomic rearrangement, phase separation, or ordering during a synthesis, thereby promoting random atomic mixing and trapping metastable single-phase structures under nonequilibrium conditions.^92,93^ In other words, although the term “high-entropy” suggests a thermodynamic origin, the formation of nanoscale HEAs during a synthesis is often determined by kinetic factors.

Taking the RuPtPdFeCo HEA system as an example, substituting FCC-structured Rh with HCP-structured Ru induces a transition from homogeneous elemental mixing to local ordering (Figure 4A).^92^ At moderate Ru concentrations (10–20 at. %), Ru atoms tended to form nanoscale clusters embedded within the FCC solid solution, whereas higher Ru contents (50 at. %) drove partial transformation toward an HCP-enriched heterostructure, reflecting relaxation toward the thermodynamically favored configuration. Density functional theory (DFT) calculations further indicated that this transition is primarily governed by chemical-affinity disparity rather than the atomic-size effect. The calculated formation free energies obtained by introducing equimolar Rh and Ru into the PtPdFeCo system are −0.055 and +0.028 eV atom^–1^, respectively, suggesting that Rh incorporation favors the formation of a well-mixed solid-solution phase, whereas Ru addition promotes phase segregation in the quinary RuPtPdFeCo system. A similar phenomenon occurs in PdSnFeCoNi HEAs (Figure 4B), where Pd–Sn pairs exhibit a significantly negative formation energy (−0.579 eV atom^–1^) and lower surface energy compared to Pd–Fe, Pd–Co, or Pd–Ni pairs.^93^ During pulsed annealing, these factors drive Pd and Sn atoms to associate and migrate toward the surface, forming ultrafine PdSn local ensembles within the HEA matrix. This microsegregation reflects a balance between the strong enthalpic driving force of Pd–Sn bonding and the configurational entropy that stabilizes the overall solid solution. Collectively, these examples demonstrate that thermodynamic immiscibility, arising from crystal-structure incompatibility and chemical-affinity disparity, is a key intrinsic factor that limits uniform atomic mixing in multicomponent systems. Addressing this challenge requires optimization of the composition and reaction temperature to modulate thermodynamic tendencies, together with kinetic control during synthesis to suppress local ordering, phase separation, and atomic rearrangement.

**Figure 4:**
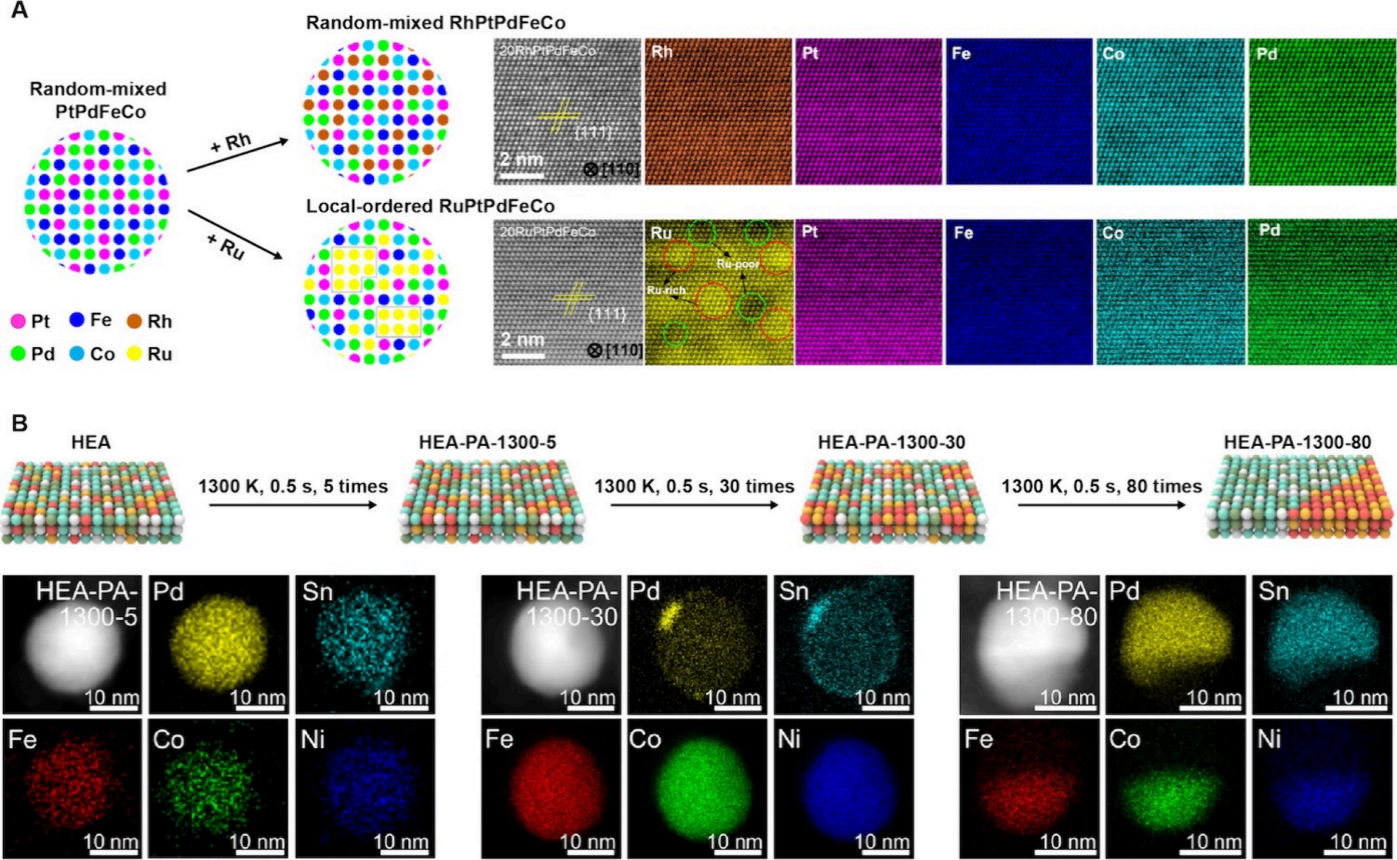
Composition–atomic mixing relationship in multicomponent HEA nanocrystals. (A) Composition-dependent structural evolution of PtPdFeCo-based HEAs, along with corresponding HAADF-STEM images and elemental maps. Incorporating Rh led to random elemental mixing and the formation of homogeneous RhPtPdFeCo solid solutions, whereas substituting Rh with Ru induced localized Ru aggregation within the FCC matrix, forming a locally ordered RuPtPdFeCo HEA while maintaining a single-phase structure. (B) Structural modulation and surface segregation in PdSnFeCoNi HEAs during pulsed annealing at 1300 K for different heating cycles (5, 30, and 80 times). Corresponding HAADF-STEM images and elemental maps reveal that increasing the number of pulses promotes Pd–Sn association and surface migration, leading to the formation of ultrafine PdSn local ensembles while preserving the overall HEA framework. The image in (A) was reproduced with permission from ref 92. Copyright 2024 American Chemical Society. The image in (B) was reproduced with permission from ref 93. Copyright 2025 Springer Nature.

### 2.2 Controlling the Shape and Surface Structure of HEA Nanocrystals

Beyond achieving uniform composition, controlling the shape and surface structure of HEA nanocrystals presents another fundamental challenge. In multicomponent systems, maintaining random atomic mixing while preserving long-range crystallographic symmetry requires a delicate balance among configurational entropy, crystal symmetry, and interfacial energy. The random incorporation of elements with different atomic sizes, electronegativities, and cohesive energies disrupts the regular surface atomic arrangement, inducing lattice strain, local defects, and morphological distortion during growth. Consequently, obtaining nanocrystals that simultaneously exhibit both atomic-level randomness and well-defined shapes, such as cubes enclosed by {100} facets with square symmetry or octahedra enclosed by {111} facets with hexagonal configuration, demands precise regulation of reduction kinetics and surface energy differences. These competing thermodynamic and kinetic factors make it inherently difficult to couple atomic disorder with geometric order in HEA nanocrystals.

Enhancing atomic intermixing in HEA nanocrystals generally requires elevated temperatures to accelerate atomic diffusion among constituent elements. However, the high temperatures simultaneously drive the system toward thermodynamic equilibrium, leading to isotropic shapes or morphologies such as spheres, Wulff-type, or irregular shapes that minimize the total surface energy.^94–96^ Consequently, most reported HEA nanocrystals exhibit quasi-spherical shapes with poorly defined facets. In contrast, low-temperature syntheses can preserve well-defined facets but often result in incomplete alloying or compositional heterogeneity in these kinetically controlled systems. This intrinsic competition between thermodynamic and kinetic factors constitutes the central obstacle to facet engineering in HEA nanocrystals. In other words, achieving an optimal balance between sufficient atomic diffusion and the preservation of facet-defined morphology remains a key challenge for obtaining HEA nanocrystals with both uniform atomic mixing and well-defined crystallographic orientations.

This delicate thermodynamic–kinetic balance not only governs morphology formation but also determines the structural stability of HEA nanocrystals under thermal activation. Recent *in situ* analyses provided valuable insights into the interplay between these competing factors.^81^ Using *in situ* HAADF-STEM, a metastable phase was identified in PtRuFeCoNi atomic layers supported on Pd nanocubes (Figure 5). Upon heating to 200 °C, the cubic morphology remained intact with a solid-solution FCC structure, indicating kinetic stabilization of the high-entropy configuration. Further heating to 300 and 400 °C induced local disorder-to-order transitions, forming L1_0_- and L1_2_-type intermetallic phases, respectively. These ordered phases originated from the inward diffusion of Fe, Co, and Ni atoms from the shell into the Pd core and the outward migration of Pt and Ru atoms, driven by the differences in atomic radii and diffusion barriers. When the temperature reached 500 °C, phase separation occurred, accompanied by the formation of a Pt-rich skin and distinct intermetallic and Pd domains. The morphology concurrently evolved into a more rounded form, confirming the thermodynamic instability of the initially high-entropy configuration. This sequence of structural evolution, from a random solid solution (25 °C), to ordered intermetallics (300–400 °C), and finally to phase-separated structures (500 °C), reveals the metastable nature of the far-from-equilibrium PtRuFeCoNi HEA nanocrystals. These observations demonstrated that while high-temperature annealing facilitates multielemental diffusion, it also drives the system toward thermodynamically favored planes. Therefore, precise tuning of temperature and reaction kinetics is essential for achieving HEA nanocrystals that possess both well-defined facets and preserved high-entropy disorder.

**Figure 5:**
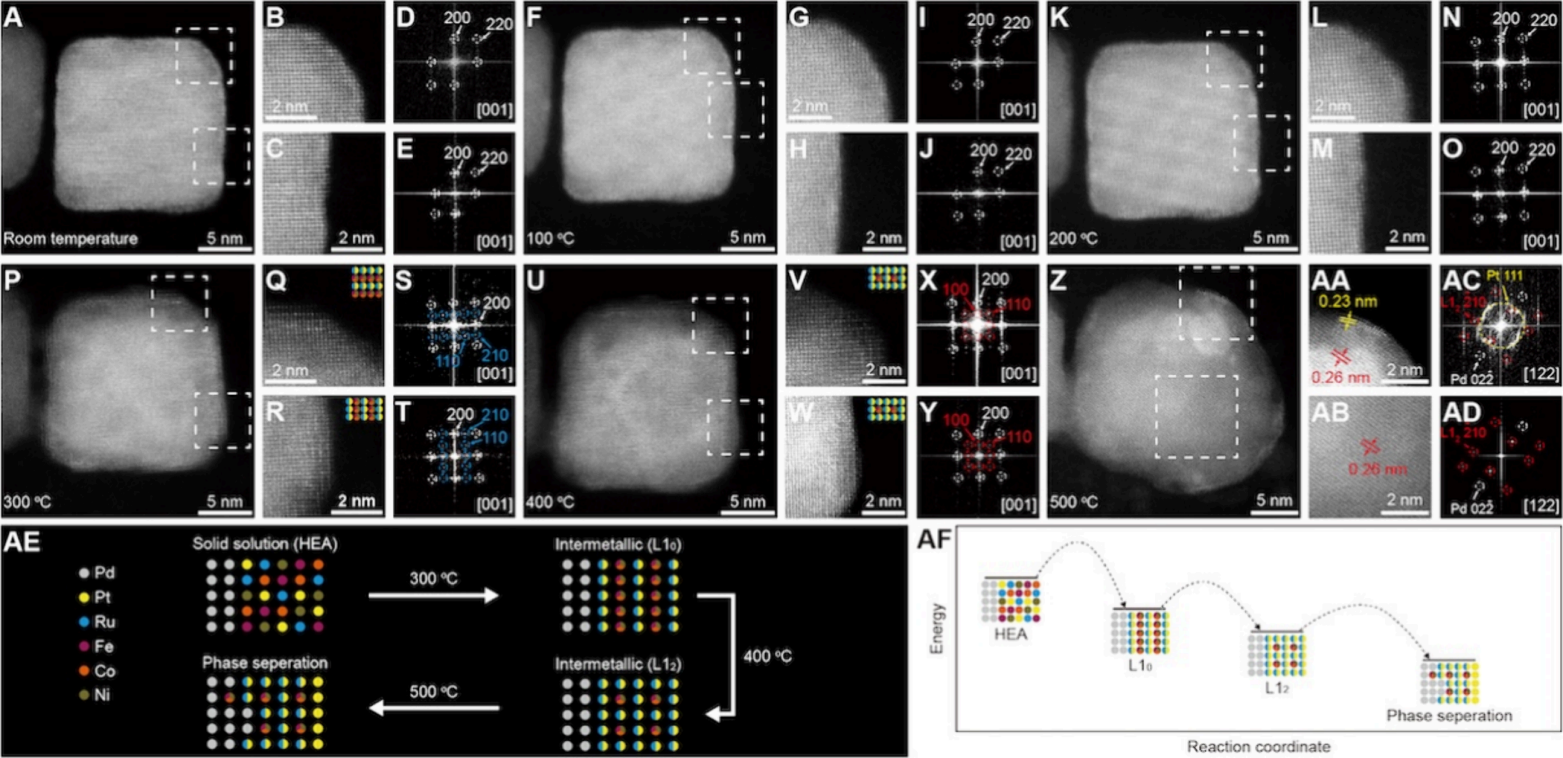
*In situ* observation of temperature-dependent structural evolution in Pd@PtRuFeCoNi HEA nanocubes. HAADF-STEM images and corresponding FFT patterns of Pd@PtRuFeCoNi core–shell nanocubes recorded at various temperatures: (A-E) room temperature, (F–J) 100 °C, (K–O) 200 °C, (P–T) 300 °C, (U–Y) 400 °C, and (Z–AD) 500 °C. (AE) Schematic illustration showing the temperature-driven phase evolution of PtRuFeCoNi atomic layers. (AF) Schematic energy landscape of PtRuFeCoNi atomic layers, revealing the transition from solid-solution to ordered intermetallic and phase-separated states. Reproduced with permission from ref 81. Copyright 2024 AAAS.

The rational construction of high-index or low-coordination surfaces adds another level of complexity. These surfaces, rich in atomic steps and kinks, provide abundant active sites that enhance intrinsic catalytic activity by lowering activation barriers for bond cleavage and formation.^97,98^ Yet their high surface energies make them susceptible to reconstruction into low-index facets unless kinetically stabilized. At elevated temperatures, extensive atomic diffusion promotes solid-solution formation but simultaneously drives the system toward smooth, low-energy planes. Under low-diffusion conditions, however, kinetic limitations lead to incomplete alloying and local segregation. Achieving both compositional uniformity and high-index surface exposure, therefore, requires precise controls over reduction, nucleation, and growth dynamics. Recent advances have introduced promising strategies to address these trade-offs. Kinetic regulation through dropwise precursor addition allows simultaneous control of atomic deposition and diffusion, leading to HEA dendritic nanocrystals that expose multiple low-coordination sites.^78^ Alternatively, epitaxial growth on concave nanocrystal templates can stabilize high-index or under-coordinated facets in HEA shells by preserving the atomic registry of preformed cores.^82,99^ These approaches demonstrate that coupling kinetic control with structural templating can effectively reconcile the competing demands of atomic mixing and facet definition.

In conventional wet-chemical synthesis, the control of crystal facets primarily depends on surface capping ligands that selectively adsorb on specific crystallographic planes.^60^ In multicomponent HEA systems, however, the random surface composition significantly reduces the facet selectivity of these ligands because the local chemical environments vary across the surface. This compositional heterogeneity makes it difficult to identify capping molecules that can simultaneously coordinate with multiple metal species, thereby weakening their ability to direct anisotropic growth. As a result, only a small proportion of HEA nanocrystals expose well-defined facets, and the overall shape uniformity within an ensemble remains limited. Consequently, successful examples of shape-controlled HEA nanocrystals synthesized through surface capping ligands remain very limited. One representative case is the use of carbon monoxide as a small-molecule capping agent to direct the formation of octahedral Ru-doped PtFeNiCuW HEA nanocrystals supported on carbon nanotubes.^100^ This study demonstrated that selective adsorption of small molecules can modulate surface energy and guide facet evolution even in compositionally complex systems. Nevertheless, the underlying mechanism remains poorly understood and appears to depend strongly on the specific interactions between the HEA surface, the adsorbate, and the support.

### 2.3 Characterizing HEA Nanocrystals

Accurate characterization is essential for verifying the compositional uniformity, atomic-scale coordination environments, and surface facets of HEA nanocrystals, all of which collectively determine their physicochemical properties and catalytic performance. Unlike monometallic or bimetallic systems, HEAs are composed of five or more principal elements with often similar atomic numbers, making it extremely challenging to establish an unambiguous structural description.^101^ Therefore, developing reliable, quantitative, and correlative characterization strategies is crucial for elucidating how atomic mixing, crystal orientation, and surface configuration collectively dictate the behavior of multicomponent systems under synthetic and catalytic conditions.

TEM, STEM, and EDS are fundamental tools for analyzing morphology and structure, providing information on particle size, lattice structure, and elemental distribution. When combined with electron energy loss spectroscopy (EELS), they can also be used to analyze elemental composition and chemical bonding at the nanoscale, offering insights into overall structural and compositional uniformity. However, limited sampling areas, beam-induced damage, and nanometer-scale spatial resolution can make it difficult to distinguish true random atomic mixing from local segregation, particularly for elements with similar atomic numbers.^84^ In such cases, apparently uniform EDS maps may mask atomic-scale heterogeneity, which is especially important for HEAs because subtle differences in atomic mixing can affect local coordination environments and catalytic properties. Surface-sensitive techniques such as XPS provide additional information on surface composition and oxidation states, but in multicomponent systems, the signals can be affected by peak overlaps and averaging effects. Therefore, although TEM, EDS, and XPS provide valuable structural and chemical information, they are often insufficient on their own to fully resolve atomic-level distributions in HEA nanocrystals.

To complement these techniques, synchrotron-based XAS has been widely used to probe local atomic environments in HEAs.^102,103^ XAS, including both X-ray absorption near-edge structure (XANES) and extended X-ray absorption fine structure (EXAFS), provides element-specific information such as coordination number, interatomic distance, and structural disorder. Because it can distinguish heterometallic from homometallic coordination, XAS is particularly useful for evaluating whether apparent compositional uniformity is consistent with atomic-level mixing or instead reflects local ordering or segregation. In well-mixed HEAs, each element statistically coordinates with others, whereas segregation or ordering tends to increase homometallic coordination and reduce heterometallic pairing. In this way, XAS provides an important complement to microscopy and spectroscopy for assessing local structure. Nevertheless, XAS also has its own limitations, particularly in resolving atomic distributions of the outermost layer where catalytic reactions occur.

Overall, reliable structural identification of HEA nanocrystals requires the combined use of multiple characterization techniques. Electron microscopy and surface spectroscopy are indispensable for morphological and chemical analyses, while coordination-resolved methods such as XAS provide additional insight into local atomic environments. However, resolving the composition and oxidation states of the outermost layer remains challenging because most available techniques either average over several atomic layers or mainly probe bulk and near-bulk coordination environments. This difficulty is further compounded by the compositional complexity of HEAs, as well as possible surface reconstruction, segregation, and dynamic redox under reaction conditions. Addressing these challenges requires more surface-sensitive and operando approaches with improved spatial and chemical specificity, together with rigorous cross-validation using complementary techniques.

## 3 Synthetic Strategies

Achieving HEA nanocrystals with well-defined shapes and uniform atomic-level mixing requires precise controls over thermodynamic and kinetic parameters (Table 1), as discussed in Section 2. Small variations in these parameters can alter alloying extent, lattice strain, facet exposure, and ultimately catalytic performance. These challenges indicate that HEA nanocrystal synthesis (Table 2) is a complex multivariable problem rather than a simple extension of conventional metal nanocrystal synthesis. Recent studies have developed diverse strategies that can be broadly divided into top-down and bottom-up approaches. Top-down methods generate nanoscale materials from bulk HEAs through fragmentation, but they often suffer from high energy consumption and limited controls over atomic-level mixing and particle shape. Bottom-up methods, by contrast, prepare HEA nanocrystals through precursor reduction, nucleation, growth, and alloying, providing greater controls over composition, phase, and crystal structure, although precise controls remain challenging. In this section, we provide an overview of key synthetic strategies, broadly categorized into top-down (Section 3.1) and bottom-up (Section 3.2) approaches. Particular emphasis is placed on wet-chemical synthesis (Section 3.3), a bottom-up approach that merits a standalone section because it provides the finest controls over reduction, nucleation, growth, and alloying dynamics, critical factors for addressing challenges such as reduction kinetics mismatch, elemental immiscibility, and crystallographic incompatibility.

**Table 2:** Summary of Synthetic Strategies, Compositions, Structures, Shapes/Morphologies, and Catalytic Applications of HEA Nanocrystals^a^

	Synthetic Strategy	Composition	Structure	Shape/morphology	Catalytic Application	Ref.
Top-down methods	Cryo-milling	FeCoNiZnGa	FCC	Spherical	OER	^104^
	Dealloying	AlFeCoNiCuPdPtAu	FCC	Porous	HER	^105^
	Dealloying	AlMnNiCuPt	FCC	Porous	ORR	^106^
	Dealloying	AlNiCuMoPd	FCC	Porous	Ethanol oxidation reaction (EOR)	^107^
	Dealloying	VMnFeCoNiCuMoRuPdIrPtAu	FCC	Porous	HER, ORR	^108^
	Electrical discharge ablation	VNbMoTaW	FCC	Spherical	HER	^109^
	Laser ablation	CrMnFeCoNi	FCC	Spherical	OER	^110^
	Laser ablation	CrNiCuIrPt, FeNiPdPtAu	FCC	Spherical	HER, OER	^111^
	Melting and cryogrinding	CuPdAgPtAu	FCC	Spherical	CO_2_RR	^47^
Bottom-up methods	Carbothermal shock	FeCoNiCuPdSnPtAu	FCC	Spherical	NH_3_ oxidation	^18^
	Carbothermal shock	FeCoNiCuMo	FCC	Spherical	NH_3_ decomposition	^55^
	Continuous-flow reactor	RuRhPdIrPt	FCC	Spherical	HER	^21^
	Co-reduction and annealing	FeCoNiRhPt	FCC	Spherical	HER	^19^
	Droplet-to-particle	FeCoNiRuIr	FCC	Hollow spherical	ORR, OER	^112^
	Fast-moving bed pyrolysis	FeCoPdIrPt	FCC	Spherical	HER	^34^
	Freeze–thaw method	CuPdAgInAuBi	FCC	Porous	CO_2_RR	^48^
	Freeze–thaw method	RuRhPdIrPt	FCC	Aerogel-like	HOR	^39^
	Hydrogen spillover-driven synthesis	CoNiCuRuPd	FCC	Spherical	CO_2_RR	^89^
	High-temperature shock	FeCoNiPdSn	FCC	Spherical	EOR	^113^
	Joule heating	MnFeCoMoRu	HCP	Spherical	OER	^44^
	Nanodroplet-mediated electrodeposition	FeCoNiLaPt	Amorphous	Irregular	HER, OER	^114^
	Polymer nanofiber reactor	MnFeCoNiRu	FCC	Spherical	HER, OER	^35^
	Shock-heating and shock-cooling strategy	MnFeCoNiPt	FCC	Spherical	ORR	^115^
	Solvothermal method	MnFeCoNiPdSnPt	FCC	Irregular	EOR	^116^
	Solvothermal method	AlFeCoNiCu	FCC	Irregular	CO_2_RR	^49^
	Sparking synthesis	FeCoNiCuMoSn	FCC	Spherical	Lithium–sulfur redox reaction	^117^
	Thermal decomposition reduction	FeCoNiCuRuPt	FCC	Spherical	MOR	^118^
	Wet-chemical synthesis	FeCoNiCuPt	FCC	Spherical	HER, MOR	^119^
	Wet-chemical synthesis	RuRhPdOsIrPt	FCC	Spherical	EOR	^120^
	Wet-chemical synthesis	FeCoNiMoRuPt	FCC	Wire-like	HOR	^20^
	Wet-chemical synthesis	FeCoNiCuRu	HCP	Spherical	Nitrogen reduction reaction (NRR)	^121^
	Wet-chemical synthesis	FeNiCuMoRuPt	FCC	Convex cubic	HER, OER, and ORR	^122^
	Wet-chemical synthesis	RuRhPdIrPt	FCC	Spherical	HER	^123^
	Wet-chemical synthesis	CoNiCuPdPt, FeNiCuPdPt	FCC	Spherical	ORR	^124^
	Wet-chemical synthesis	RuPdAgIrPt	FCC	Ribbon-like	ORR	^125^
	Wet-chemical synthesis	NiGaMoPdIn	FCC	Sheet-like	HER	^126^
	Wet-chemical synthesis	RhSnSbPtBi	HCP	Plate-like	MOR	^127^
	Wet-chemical synthesis	FeCoNiPdPt	FCC	Irregular	ORR	^128^
	Wet-chemical synthesis	RuRhPdIrPt	FCC	Dendritic	HER, HOR	^78^
	Wet-chemical synthesis	NiRuRhPdIr	FCC	Irregular	HOR	^36^
	Wet-chemical synthesis	FeCoNiRuPt	FCC	Cubic	HER, HOR	^81^
	Wet-chemical synthesis	RuRhPdIrPt	HCP	Spherical	HER	^129^
	Wet-chemical synthesis	RuRhPdIrPt	FCC	Cubic	HER	^82^
	Wet-chemical synthesis	FeCoNiMoIrPt	FCC	Dendritic	HOR	^130^
	Wet-chemical synthesis	CoNiMoRuIr	FCC	Irregular	OER	^131^
	Wet-chemical synthesis	RuRhPdIrPt	FCC	Cage-like	HER, OER	^132^
	Wet-chemical synthesis	FeNiCuWPt	FCC	Octahedral	HER	^100^
	Wet-chemical synthesis	RuRhPdIrPt	FCC	Frame-like	HER	^133^
	Wet-chemical synthesis	NiCuRuInPt	FCC	Irregular	HOR	^134^
	Wet-chemical synthesis	FeCoNiCuPt	FCC	Irregular	Nitrate reduction reaction (NO_3_RR)	^135^
	Wet-chemical synthesis	CoNiCuMoIrPt	FCC	Octahedral	MOR	^31^

a For consistency, alloy compositions are listed with increasing atomic numbers and grouped by the synthetic strategies.

### 3.1 Top-Down Methods

#### 3.1.1 Milling

A variety of milling techniques have been developed to mechanically downsize bulk high-entropy materials (HEMs) into nanoscale particles. In conventional ball milling, the grinding medium within a rotating cylindrical chamber generates repeated impact and shear forces that induce fracture and gradual particle size reduction. In contrast, cryo-milling is performed at cryogenic temperatures, typically achieved by cooling the milling vial with liquid nitrogen to embrittle the material, thereby enhancing its fragmentation efficiency. Moreover, the low-temperature environment effectively suppresses undesirable phase transformations and compositional changes that may otherwise occur during high-energy milling processes.

##### 3.1.1.1 Mechanical Alloying (MA)

MA is a solid-state processing method for synthesizing HEA powders under high-energy milling conditions. In a typical MA process, a mixture of elemental powders and grinding medium is loaded into a high-energy ball milling vessel and subjected to vigorous rotation or vibration for a predetermined duration.^136,137^ The repeated impact of the milling balls transfers mechanical energy to the powders, inducing severe plastic deformation and promoting atomic-scale interdiffusion through persistent collisions between the powder particles and the milling medium. This cyclic welding–fracturing mechanism gradually leads to the formation of a homogeneous solid-solution phase.^138^ A major achievement in this field was first reported by Varalakshmi and co-workers in 2008, who successfully synthesized a nanocrystalline Al–Cr–Cu–Fe–Ti–Zn HEA through MA. Their work demonstrated the feasibility of producing nanocrystalline HEAs through purely mechanical means.^139^

Several key factors determine the ultimate phase constitution and chemical uniformity of mechanically alloyed HEAs. These include: *i*) the powder blending environment, which must ensure homogeneous microscale mixing of the elemental components prior to milling; *ii*) milling parameters, such as the type of milling medium, ball-to-powder ratio, milling duration, and rotational speed, all of which influence the energy input and mixing efficiency; and *iii*) postmilling treatments, including annealing or sintering, which can further homogenize the alloy, refine the microstructure, and stabilize the desired phases.^138,140^ The importance of processing conditions has been evaluated by Custodio and co-workers, who studied the AlCoCrFeNi HEA system under different thermodynamic and milling regimes. After 24 h of conventional high-energy ball milling, the alloy exhibited a dual-phase structure comprising FCC and BCC phases, with respective fractions of 44.74% and 55.26%. By contrast, when a sequential milling strategy was employed, progressively introducing Fe, Ni, Co, Cr, and Al at 4 h intervals, the same alloy evolved into a single-phase BCC structure after 24 h. This phase stabilization is attributed to the role of Al as a strong BCC stabilizer, owing to its negative enthalpy of mixing with the other constituent elements, which enhances its affinity and promotes uniform BCC phase formation (Figure 6A, B).^141^

**Figure 6:**
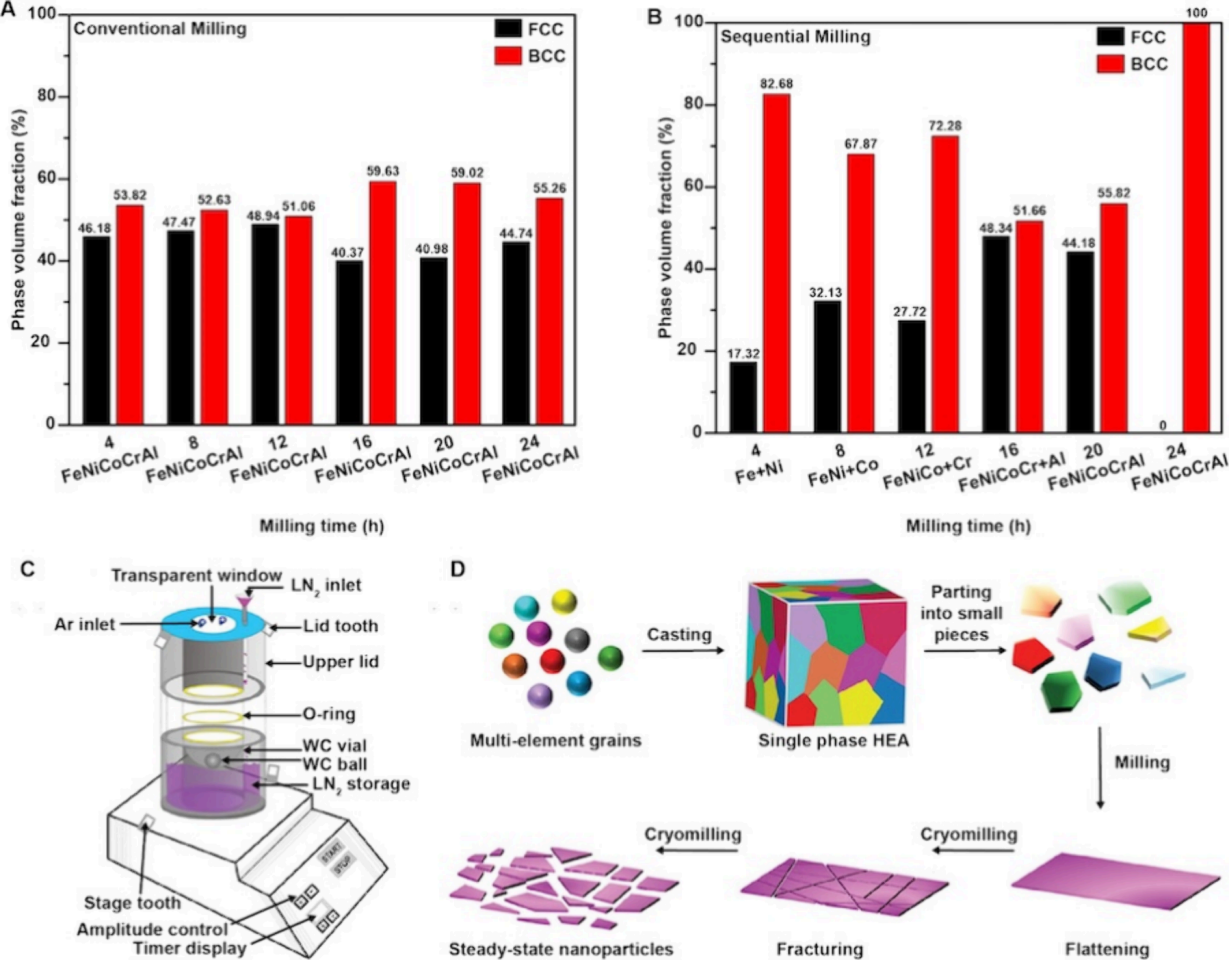
Top-down milling strategies for synthesizing nanoscale HEAs. (A, B) Phase-volume diagrams of FCC and BCC phases derived from X-ray diffraction (XRD) analysis of (A) FeNiCoCrAl during conventional milling and (B) FeNiCoCrAl under sequential milling. (C) Schematic of a custom-built tungsten carbide ball cryo-mill equipped with liquid-nitrogen cooling and an inert-gas protection system. (D) Schematic of the top-down cryo-milling process, showing the conversion of a bulk single-phase HEA into nanocrystals through sequential flattening, fracturing, and steady-state nanoparticle formation. The images in (A, B) were reproduced with permission from ref 141. Copyright 2024 Elsevier. The images in (C, D) were reproduced with permission from ref 143. Copyright 2024 Wiley-VCH.

##### 3.1.1.2 Cryo-Milling

To physically reduce HEA ingots to the nanometer scale, Kumar and co-workers developed a cryo-milling strategy that enables effective size reduction under an extremely low temperature.^104,142^ As illustrated in Figure 6C, a typical cryo-milling apparatus is equipped with a tungsten carbide milling ball and incorporates several essential design features: *i*) physical separation between the cryogenic liquid and the metal powder, *ii*) protection of the powder against oxidation and nitridation by maintaining an inert gas atmosphere (e.g., Ar) inside the vial, *iii*) precise control of the powder temperature throughout the milling process, and *iv*) a visual monitoring system to assess the energy transfer from the milling medium to the powder.^143^

Figure 6D schematically illustrates the top-down cryo-milling pathway from bulk single-phase HEA ingots to nanoscale particles, rather than implying that the intermediate flattening and fracturing behaviors are unique characteristics of HEA nanocrystals.^143^ The process typically begins with the preparation of single-phase HEA ingots through arc melting of high-purity elemental metals in an Ar-protected environment, followed by homogenization at 1000 °C for 10 h and subsequent casting. Both FCC alloys (e.g., FeCrMnNiCo and CuAgAuPtPd) and BCC alloys (e.g., FeCrMnVAl) can be used as starting materials. The resulting ingots are first sectioned into smaller fragments using a diamond saw and then loaded into the cryo-mill. During milling, liquid nitrogen is employed as the cryogenic medium (−160 ± 10 °C), and a continuous Ar purge is applied to further minimize oxidative contamination.

Cryo-milling proceeds through three distinct microstructural refinement stages: *i*) particle flattening, during which the ingot fragments are deformed into thin sheets; *ii*) sheet fracturing, facilitated by the increased brittleness of the alloy at a cryogenic temperature; and *iii*) steady-state nanocrystal formation, in which repeated fracture and limited cold welding yield uniform nanoscale particles. Although slight cold welding occurs during the initial flattening stage, the bond strength of these welds is weak, allowing easy fragmentation in subsequent impacts. The suppressed dynamic recovery at low temperature significantly enhances brittleness, accelerating fracture and minimizing particle merging. These intermediate deformation and fracture steps are not unique to HEAs, but in the present context they enable direct size reduction of existing single-phase HEA bulk alloys into nanocrystals while largely preserving compositional uniformity. An additional advantage of cryo-milling is the reduction in contamination and oxidation, as the required milling duration is typically much shorter than in conventional milling. Continuous milling for around 6 h is generally sufficient to produce HEA nanocrystals directly from ingots. For instance, single-phase FCC FeCrMnNiCo nanocrystals synthesized by this method retained their equimolar composition and exhibited a uniform average particle size of 4 ± 1 nm.

In summary, although milling-based techniques provide a straightforward, solvent-free, and scalable route to produce nanoscale HEAs, they have important limitations, especially for catalysis-oriented applications. They offer limited controls over particle morphology and facet exposure, while contamination from the milling media and oxidation during processing can alter both bulk composition and surface chemistry. In addition, severe plastic deformation often induces amorphization or metastable phases, making postannealing necessary but also increasing the risk of grain growth, phase segregation, and compositional heterogeneity. Milling also typically requires long processing times and high energy input. Although cryo-milling can mitigate oxidation and cold welding, it introduces additional complexity in equipment, handling, and scale-up.

#### 3.1.2 Dealloying

Dealloying is viewed as a top-down, postsynthetic transformation of preformed alloy materials, in which selective dissolution of one or more components generates porous or layered architectures.^144,145^ Rather than constituting a distinct primary route for the synthesis of discrete HEA nanocrystals, its role in the high-entropy field is mainly to modify morphology, porosity, and surface structure after a multicomponent material has already been prepared. This distinction is important in the context of HEA nanocrystal synthesis because the chemical complexity and phase constitution of the preformed material are established prior to dealloying, whereas the dealloying process primarily serves to tailor postsynthetic structural and surface features, including pore architecture, surface area, local coordination environments, oxidation state, and lattice strain.^146,147^

##### 3.1.2.1 Preparation of Layered HEAs by Dealloying

A notable example of dealloying in multicomponent systems is the preparation of layered high-entropy MXenes through the selective removal of the A-site element (e.g., Al, Si, Ga, or Sn) from pre-existing high-entropy MAX phases. Nemani and co-workers demonstrated this approach by deriving two quaternary high-entropy two-dimensional (2D) carbides, TiVNbMoC_3_T_*x*_ (T_*x*_ refers to the surface termination groups of the MXenes, such as −O, −F, and −OH) and TiVCrMoC_3_T_*x*_, from their respective TiVNbMoAlC_3_ and TiVCrMoAlC_3_ MAX-phase precursors.^148^ The precursor MAX phases were first synthesized via high-temperature sintering of stoichiometric elemental powders at 1600 °C under an inert atmosphere. For the etching process, approximately 2 g of the MAX powder was gradually introduced (over about 60 s) into a polyethylene vessel containing 20 mL of the etchant made of 48 wt % hydrofluoric acid solution. The reaction was conducted at 55 °C under continuous stirring (400 rpm) for 4 days to selectively remove the Al component. This controlled etching process produced multilayered TiVNbMoC_3_T_*x*_ and TiVCrMoC_3_T_*x*_ MXenes, each exhibiting a near-equimolar transition-metal ratio of 1:1:1:1 (±0.2) for M_1_:M_2_:M_3_:M_4_, confirming the successful preservation of high-entropy compositional uniformity in the resulting 2D structures (Figure 7A).

**Figure 7:**
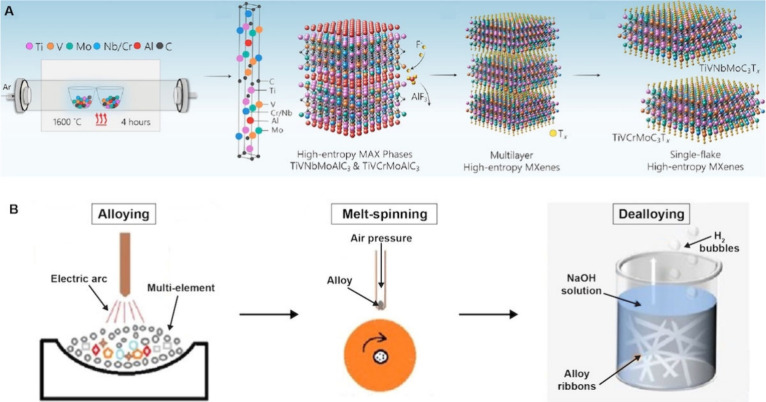
Preparation of nanoscale HEAs by dealloying: (A) formation of HEA nanosheets and (B) generation of highly porous HEA nanostructures. The image in (A) was reproduced with permission from ref 148. Copyright 2021 American Chemical Society. The image in (B) was reproduced with permission from ref 150. Copyright 2021 American Chemical Society.

##### 3.1.2.2 Preparation of Highly Porous HEAs by Dealloying

Qiu and co-workers demonstrated the successful fabrication of highly porous HEA systems with up to 16 elements through chemical dealloying of bulk precursor alloys (Figure 7B).^105,106,108,149,150^ Representative examples include senary AlNiCuPtPdAu, octonary AlNiCuPtPdAuCoFe, and senary all-non-noble-metal AlNiCuMoCoFe porous HEAs prepared through dealloying of Al-based alloys. Each sample of porous HEAs had an ultrafine ligament size of 2–3 nm and a high Brunauer–Emmett–Teller (BET) surface area of approximately 52 m^2^ g^–1^. The Al-rich precursor alloys (>95 at. % Al) were synthesized by induction melting under an argon atmosphere, followed by melt spinning to produce uniform alloy ribbons.^105^ Subsequent chemical dealloying was performed in a 0.5 M NaOH solution, where the preferential dissolution of Al resulted in the formation of a bicontinuous and porous network. To eliminate surface oxide layers and obtain clean metallic ligaments, the dealloyed samples were further immersed in 1.0 M H_2_SO_4_ for 30 min. This two-step treatment effectively preserved the intrinsic metallic character of the HEA framework. Importantly, this dealloying-based strategy was extended to synthesize more complex hybrid nanostructures. For instance, HEA-oxide or HEA-hydroxide composites have also been generated by controlling the dealloying parameters.^107,151^ Such structural diversity greatly broadens the potential applications of porous HEA nanomaterials in catalysis, sensing, and energy conversion.

In summary, dealloying is a postsynthetic strategy for transforming preformed alloys or high-entropy materials into porous or layered high-entropy architectures. Its main advantage lies in generating high specific surface area, large porosity, and compositionally complex surfaces that are sought for catalysis and energy-related applications. However, several inherent limitations constrain its broader applicability to compositionally complex systems. Effective dealloying depends strongly on the composition and uniformity of the initial alloy and often requires sufficient electrochemical potential differences among the constituent elements, which restricts the range of viable element combinations for generating homogeneous high-entropy solid solutions. Small deviations in stoichiometry may lead to incomplete leaching, irregular pore formation, or compositional drift. In addition, the dealloying process is highly sensitive to corrosion conditions such as etchant concentration, temperature, and reaction time, making precise control difficult and often resulting in heterogeneous microstructures or partial collapse of the porous framework. The precursor preparation itself also typically relies on energy-intensive alloying methods, such as arc or induction melting, which require specialized equipment and increase processing cost. Moreover, although dealloying produces porous structures with high surface accessibility, it is less suitable for applications that require discrete, shape-controlled nanocrystals with well-defined facets.

#### 3.1.3 Ablation

Ablation synthesis involves the application of physical shock to vaporize bulk material targets composed of single or multiple elements, followed by reduction, condensation, and deposition simultaneously to form nanoscale HEAs. This technique has emerged as a highly promising method for the rapid production of HEAs and high-entropy ceramic nanocrystals in a single step, owing to its high energy density and rapid heating and cooling cycles. These synthetic characteristics effectively mitigate phase separation during the process, enabling the preservation of homogeneous elemental distribution. It is noteworthy that ablation methods inherently encompass both top-down and bottom-up characteristics, as the process involves not only the atomization of bulk materials but also the subsequent condensation and crystallization of the ablated species, leading to homogeneous atomic mixing.

Variations of the ablation technique include electrical discharge ablation via electric arcs, laser ablation with electromagnetic pulses, and sputtering or ion beam ablation, which utilize ions to fragment the precursor bulk HEMs into smaller particles. These methods can be employed for both equiatomic and nonequiatomic compositions, offering versatility in the synthesis of a broad range of nanoscale HEAs. Process control knobs include source energy, pulse width, repetition rate, spot size, and scanning speed. The properties of the reaction medium (e.g., viscosity, polarity, and additives) may also affect stabilization, oxidation state, and aggregation.

##### 3.1.3.1 Electrical Discharge Ablation

Wire-cut electrical discharge machining is a noncontact, thermally driven process in which material is removed via a series of precisely controlled electrical discharges between a thin, continuously fed wire electrode and a conductive workpiece, such as HEA, immersed in a dielectric medium. Upon application of a pulsed direct current voltage, once the electric field exceeds the dielectric breakdown threshold, a transient plasma channel forms across the narrow interelectrode gap (typically, 10–50 μm). Each discharge event generates localized extreme temperatures that melt and vaporize a minute portion of the HEA surface. The molten material is then rapidly quenched and carried away by the flowing dielectric fluid (Figure 8A). Niu and co-workers successfully applied this technique to a bulk high-entropy carbide (MoWVNbTa)­C.^109^ Sub-10 nm high-entropy carbide nanocrystals with identical composition and abundant surface defect sites were obtained by collecting and centrifuging the waste dielectric liquid (deionized water) flushed out during the wire-cut electrical discharge process.

**Figure 8:**
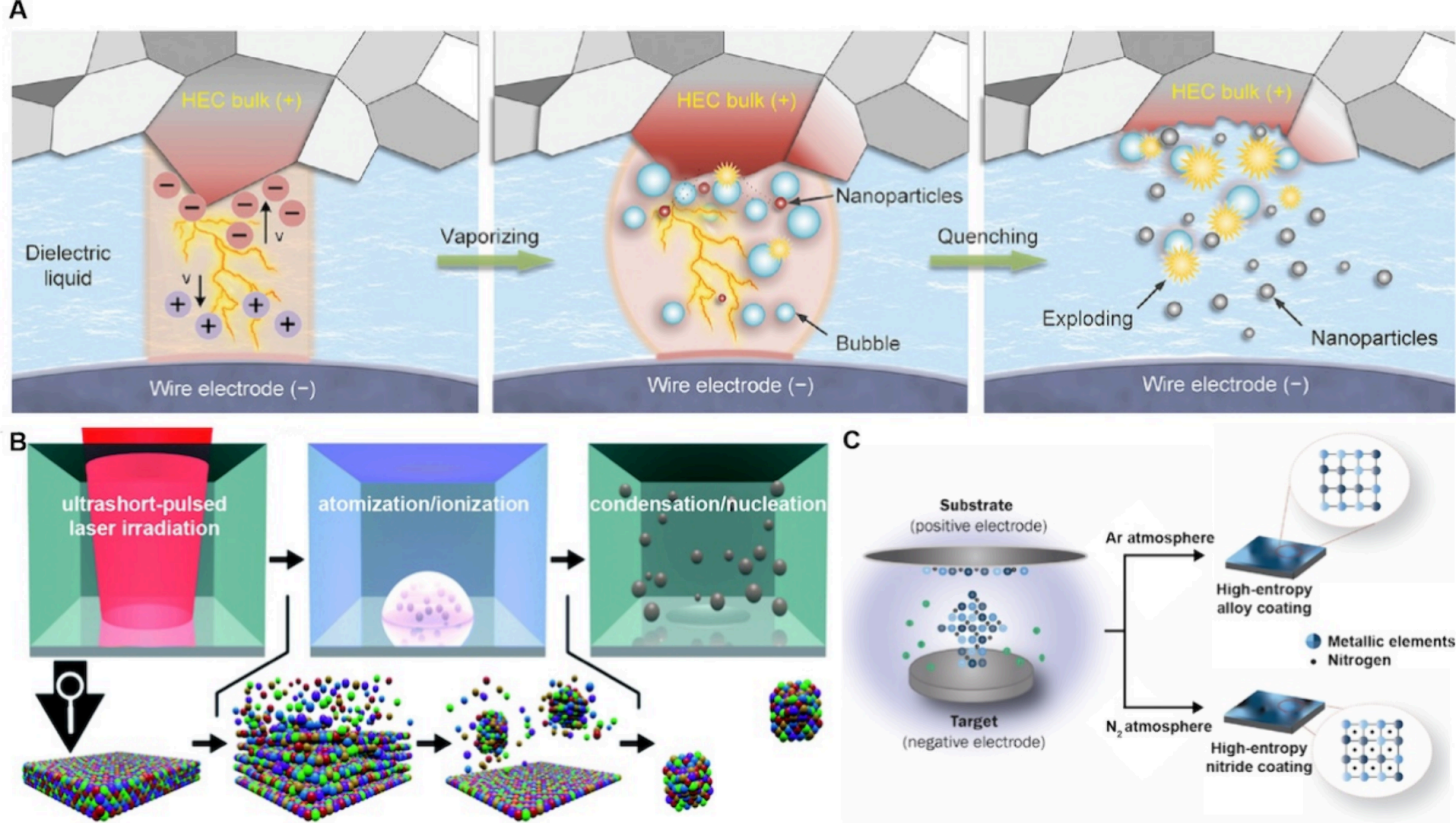
Ablation synthesis of nanoscale HEAs. (A) Schematic illustration of electrical discharge ablation for producing high-entropy carbide nanocrystals from bulk targets. (B) Schematic illustration of laser ablation, involving ultrashort-pulse irradiation, atomization/ionization, and rapid condensation into multicomponent nanocrystals. (C) Schematic illustration of sputtering under different reactive atmospheres. The image in (A) was reproduced with permission from ref 109. Copyright 2022 Wiley-VCH. The image in (B) was reproduced with permission from ref 110. Copyright 2019 Royal Society of Chemistry. The image in (C) was reproduced with permission from ref 156. Copyright 2023 MDPI.

##### 3.1.3.2 Pulsed Laser Ablation

Pulsed laser ablation utilizes short, high-intensity laser pulses to dissociate bulk HEA targets, ejecting material from the surface in the form of atomized or vaporized fragments. The process can be conducted under vacuum, in a controlled gas atmosphere, or within a liquid medium.^152^ When a laser pulse impinges on the target, photons are absorbed, and their energy is transferred to the crystal lattice, leading to rapid surface heating to near-boiling or superheated temperatures. At sufficiently high laser fluence, a plasma plume forms above the surface. Repeated pulsing promotes stoichiometric ablation of the HEA, ensuring homogeneous vaporization of all constituent elements and effective mixing within the expanding plume. During plume expansion, the ejected material experiences ultrafast cooling rates on the order of 10^6^–10^9^ K s^–1^. This rapid quenching drives nucleation and growth of multielement condensates, yielding nanocrystals that are directly released into the surrounding medium, gas or liquid, depending on the setup (Figure 8B).^110^ In liquid environments, successive pulses can further fragment larger particles, induce surface melting and recrystallization, and narrow particle size distribution. The resulting nanocrystals are typically collected on substrates, within liquid suspensions, or through filtration systems.^153^ As a notable example, Waag and co-workers synthesized colloidal CoCrFeMnNi HEA nanocrystals with diameters below 5 nm via picosecond laser ablation of bulk targets in ethanol.^110^ This method allowed for a notably high production yield, generating approximately 3 g of colloidal HEA nanocrystals per hour, underscoring the efficiency and scalability of pulsed laser ablation for HEA nanocrystal synthesis.

##### 3.1.3.3 Sputtering

Sputtering deposition is a physical vapor deposition (PVD) technique in which atoms are physically ejected from one or more solid targets due to bombardment by energetic ions. The ejected atoms subsequently travel and condense as alloyed nanocrystals or films on a substrate or within a trapping medium.^154^ Because metallic bonds in the target must be broken to generate vapor-phase atoms, the process requires substantial energy input, typically achieved under high-temperature conditions. In a standard configuration, the reaction occurs in a low-pressure vacuum chamber filled with an inert working gas (typically, Ar), which is ionized to form a plasma of energetic ions. Among the variants of this method, magnetron sputtering is the most widely adopted for HEA film fabrication. Introducing reactive gases such as nitrogen during deposition enables control over chemical composition and phase evolution, as shown in Figure 8C.^155,156^ In one study, Shi and co-workers investigated (FeMnNiCoCr)­N_*x*_ coatings and found that low nitrogen content (ca. 6 at. %) yielded an FCC structure, whereas higher nitrogen concentrations (15–26 at. %) induced transformation to a BCC phase.^157^ This transition was attributed to the self-organization of metallic atoms facilitated by nitrogen incorporation, where metal atoms randomly occupied lattice sites while nitrogen segregated preferentially along grain boundaries, stabilizing the BCC configuration.

Recently, sputtering into ionic liquids as a trapping medium has emerged as a powerful version of this technique for producing nanoscale HEAs. Ionic liquids, characterized by their negligible vapor pressure and exceptional thermal stability, serve simultaneously as the reaction medium and as stabilizing agents that regulate nucleation, growth, and morphology.^158^ Löffler and co-workers demonstrated this approach by cosputtering five elemental targets (Cr, Mn, Fe, Co, and Ni) into 1-butyl-3-methylimidazolium bis­(trifluoromethylsulfonyl)­imide to synthesize nanoscale HEAs.^159^ The independent control of sputtering rates for each elemental target allowed precise adjustment of deposition rates, thereby enabling stoichiometric tuning and compositional flexibility.

In summary, ablation-based techniques, including electrical discharge ablation, pulsed laser ablation, and sputtering, allow for the solvent-free synthesis of pure, multimetallic nanocrystals. However, these methods still face challenges in maintaining compositional stability, preventing surface oxidation, and achieving uniform particle size. Furthermore, they require high-vacuum environments, precise optical alignment, and sophisticated power modulation, which increase operational complexity. More critically, the inherently violent and nonequilibrium nature of the ablation–cooling process typically results in broad particle size distributions and irregular morphologies. Consequently, these methods do not readily permit facet- or shape-controlled synthesis, an essential requirement for correlating catalytic structure with activity in mechanistic studies.

#### 3.1.4 Limitations of the Top-Down Methods

Traditional top-down methods provide simple and versatile routes to access nanoscale HEAs through direct fragmentation or atomization of bulk alloys. In addition to these conventional approaches, discharge-based physical routes such as arc discharge have also been explored. For example, an arc-discharge strategy incorporating vapor-pressure regulation has been used to synthesize compositionally complex HEA nanocrystals containing up to a record number of twenty-one elements from a compacted mixture of multiple single-metal powders.^160^ These methods offer several notable strengths, including their solvent-free nature, diverse compositions, and potential to generate metastable solid-solution phases via rapid quenching. They are particularly advantageous for producing highly porous frameworks or surfactant-free metallic nanocrystals. However, these techniques also suffer from intrinsic limitations: poor controls of particle size, shape, and, most importantly in catalysis, facet engineering; stoichiometric fidelity often drifts during the synthesis due to unequal sputtering or evaporation rates; and oxidation or contamination can alter surface chemistry. Furthermore, their high energy consumption and equipment complexity limit the general applicability. These challenges have motivated the development of bottom-up synthetic approaches, where reaction thermodynamics and kinetics can be precisely tuned to achieve compositionally homogeneous, size-controlled, and facet-defined HEA nanocrystals.

### 3.2 Bottom-Up Methods

#### 3.2.1 Carbothermal Shock

The carbothermal shock (CTS) technique utilizes the intense Joule heating generated when a high current passes through conductive carbon supports, enabling ultrafast heating of multielement precursors to induce transient melting. The formation of a liquid phase dramatically enhances atomic diffusivity, promoting homogeneous elemental mixing that effectively minimizes enthalpy differences (Δ*H*) while maximizing the contribution from configurational entropy (*T*Δ*S*). Upon cooling, the resulting phase structure is governed by the solidification kinetics, as illustrated in the time–temperature–transformation diagram (Figure 9A).^18^ To retain a high-entropy, single-phase solid-solution structure, rapid quenching is essential to bypass the crystallization regime, often referred to as the “nose” of the time–temperature–transformation curve, and to suppress atomic segregation. In contrast, slower cooling rates allow sufficient atomic mobility for phase separation and compositional inhomogeneity, leading to the formation of multiphase or phase-segregated alloys (Figure 9A, right). Conversely, excessively rapid cooling can immobilize atomic motion entirely, trapping atoms in a disordered arrangement and yielding amorphous metallic glass (Figure 9A, left). Overall, precise kinetic controls over atomic mixing, crystallization dynamics, and diffusion suppression are all critical factors for stabilizing high-entropy, compositionally uniform nanostructures.

**Figure 9:**
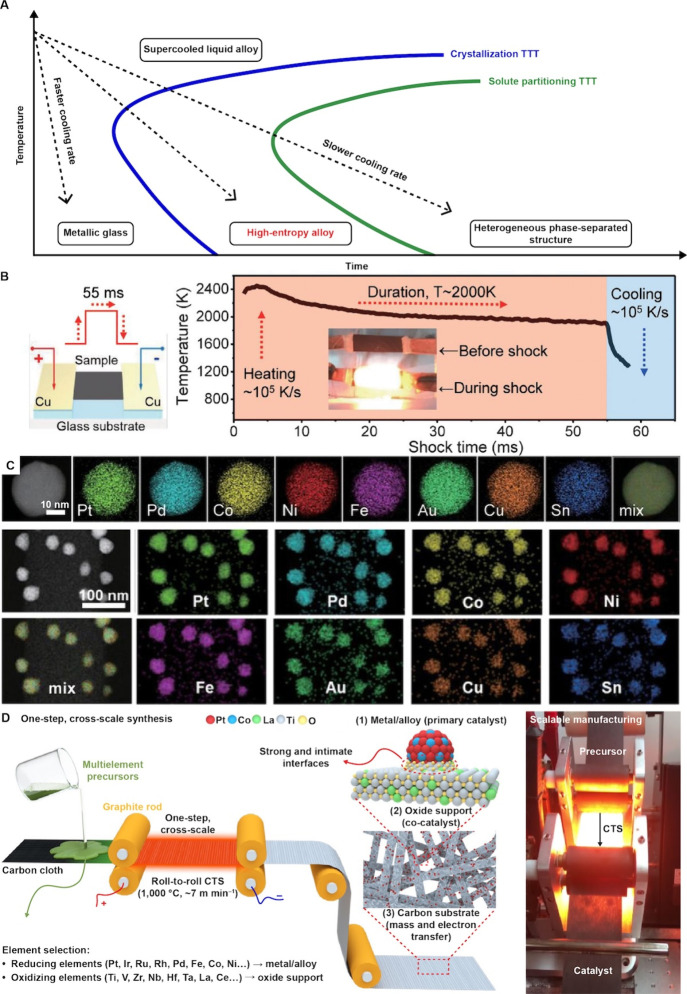
Carbothermal shock synthesis of HEA nanocrystals. (A) Time–temperature–transformation diagram illustrating the kinetic formation pathways of amorphous metallic glass, single-phase HEA, and phase-separated structures from the molten state as a function of cooling rate. (B) Schematic of the CTS process, showing the rapid heating–cooling event (55 ms) that drives the formation of HEA nanocrystals on carbon supports. (C) Representative HAADF-STEM image and corresponding EDS elemental maps of an octonary PtPdCoNiFeAuCuSn HEA-NPs. (D) Illustration of a roll-to-roll CTS setup. The images in (A–C) were reproduced with permission from ref 18. Copyright 2018 AAAS. The image in (D) was reproduced with permission from ref 161. Copyright 2025 Springer Nature.

Hu and co-workers are among the first to report the synthesis of HEA nanocrystals using the CTS method.^18^ In this process, as shown in Figure 9B, carbon nanofiber supports are infiltrated with a mixture of metal chloride precursors in equimolar proportions. A controlled electrical pulse, applied using a Keithley 2425 digital source meter, passes through the carbon fiber, generating intense Joule heating. Within approximately 55 ms, the temperature of the system surged to ca. 2000 K, achieving heating and cooling rates as high as 10^5^ K s^–1^. Under these extreme yet short-lived thermal conditions, homogeneous alloying occurred, yielding single-phase FCC HEA nanocrystals (*ca*. 5 nm in size) composed of eight distinct elements (Pt, Pd, Co, Ni, Fe, Au, Cu, and Sn, Figure 9C). A similar shock-assisted process has also been employed to synthesize CoMoFeNiCu HEA nanocrystals with tunable Co/Mo ratio. The particles had a size of *ca*. 22 nm, together with an FCC structure, in which some of the compositions broke the conventional Co–Mo bimetallic phase diagram, because of the high-entropy effect and shock method.^55^ Shi and co-workers developed a roll-to-roll CTS technique that enabled the one-step, continuous, and scalable synthesis of HEA nanocrystals (Figure 9D).^161^ In this system, mixed metal and oxide precursors were deposited on conductive carbon cloth, which was then passed between two graphite rollers under a high electrical current to generate the ultrafast Joule heating required for CTS. Large-area electrodes, up to 10 × 100 cm^2^, could be fabricated within 10 s as the substrate moved at a linear speed of 7 m min^–1^. In contrast to conventional, batch-based CTS, which is limited to milligram-scale outputs, the roll-to-roll configuration integrates rapid thermal processing with continuous material transport. This advancement achieves industrial-level throughput (116.7 cm^2^ s^–1^) while maintaining uniform nanocrystal dispersion and compositional homogeneity across the large substrate, marking a significant step toward scalable manufacturing of HEA nanocatalysts.

CTS method overcomes traditional miscibility barriers between immiscible metals, enabling the formation of single-phase solid solutions that would otherwise be thermodynamically unfavorable.^161,162^ Moreover, CTS method can be extended to synthesize other high-entropy systems, including high-entropy sulfides and oxides.^163,164^ The inherent use of conductive carbon supports provides additional advantages: the substrate prevents nanocrystal agglomeration while enhancing electrical conductivity for catalytic applications. However, the reliance on Joule heating confines this method to conductive substrates, posing challenges for synthesizing HEA nanocrystals on insulating materials. In addition, isolating or purifying the alloy nanocrystals from the carbon matrix remains difficult.^165^ Another limitation stems from the high-temperature nature of the CTS process, which typically yields spherical particles rather than nanocrystals with well-defined shapes or exposed facets.

#### 3.2.2 Liquid-Metal-Mediation

The formation of HEA phases fundamentally relies on minimizing the enthalpy of mixing or enhancing the configurational entropy term to lower the overall Gibbs free energy. In this context, Cao and co-workers introduced the use of liquid metals—such as Ga, In, and Sn, as dynamic reaction media for HEA nanocrystal synthesis.^166^ Among the liquid metals, Ga is particularly advantageous due to its negative mixing enthalpy with a wide range of metallic elements, which offers thermodynamic stabilization and facilitates atomic interdiffusion. As illustrated in Figure 10A, nanoscale-dispersed liquid metal droplets serve as atomic mixing reservoirs that are combined with multiple metal salt precursors. Upon heating under a mixed Ar/H_2_ atmosphere, the precursors undergo thermal decomposition or reduction, and the liberated metal atoms dissolve into the matrix of liquid metal. Subsequent atomic intermixing and alloying occur within this fluidic environment, leading to the formation of single-phase HEA nanocrystals at approximately 923 K. The system is then allowed to cool naturally to room temperature at a moderate rate.

**Figure 10:**
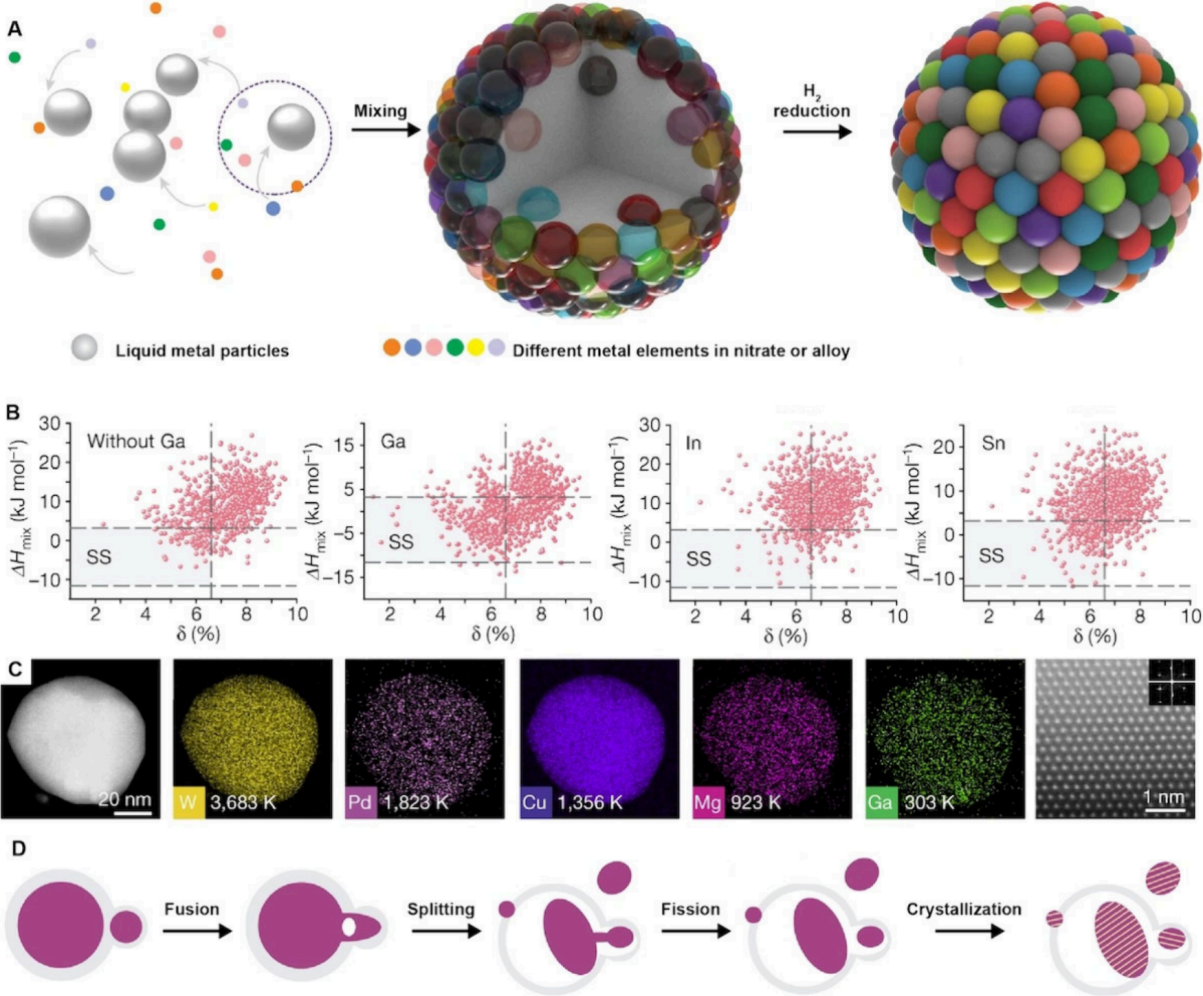
Liquid-metal-mediated synthesis of HEA nanocrystals via thermal decomposition and hydrogen reduction. (A) Schematic of the formation pathway of HEA nanocrystals. (B) Comparison of the Δ*H*_mix_ and δ parameters for HEAs without and with incorporating Ga, In, or Sn. (C) HAADF-STEM image and corresponding elemental mapping of a representative WPdCuMgGa HEA nanocrystal. (D) Schematic of the dynamic fusion–fission events among the particles during the alloying process. Reproduced with permission from ref 166. Copyright 2023 Springer Nature.

From a thermodynamic standpoint, the formation of a stable HEA phase requires that the Δ*H*_mix_ lies between −11.6 and +3.2 kJ mol^–1^ and that the atomic radius mismatch (δ) among constituent elements is below 6.6%. As shown in Figure 10B, computational analysis of equimolar combinations containing 5–10 metallic elements (selected from Fe, Co, Ni, Cu, Mn, Mg, Cr, W, Au, and Ag) reveals that incorporating Ga, In, or Sn effectively reduces Δ*H*_mix_, thereby increasing the number of compositions meeting the solid-solution phase formation criteria. This strategy has enabled the successful synthesis of HEA nanocrystals comprising up to 17 elements (GaFeNiCuZnScVMnMgZrPtRhRuIrHfMoNb). A representative example is the quinary WPdCuMgGa HEA system, which integrates metals with deviated melting points from 303 to 3,683 K (Figure 10C). The alloying process proceeds through repeated fusion–fission events within the liquid metal environment, promoting rapid elemental migration and uniform atomic-scale mixing during the 6 h annealing at 923 K (Figure 10D).

Zhang and co-workers also introduced a liquid-metal-mediated isothermal solidification method for synthesizing HEA nanocrystals with tailored morphology, crystallinity, and composition.^167^ The strategy exploits liquid metals, most notably Ga or Ga-based alloys, as sacrificial reaction media for alloy formation. In this process, Ga nanocrystals were dispersed into an aqueous solution containing multiple metal cations. At the liquid–liquid interface, Ga atoms serve as a powerful reductant, converting the dissolved metal ions into their metallic form. The newly generated metal atoms immediately dissolved into the liquid Ga phase, replacing Ga atoms while maintaining a homogeneous liquid environment. As the reaction proceeded, Ga was gradually depleted, and the droplet became enriched with other metallic constituents, resulting in the spontaneous formation of HEAs (Figure 11A).^168^

**Figure 11:**
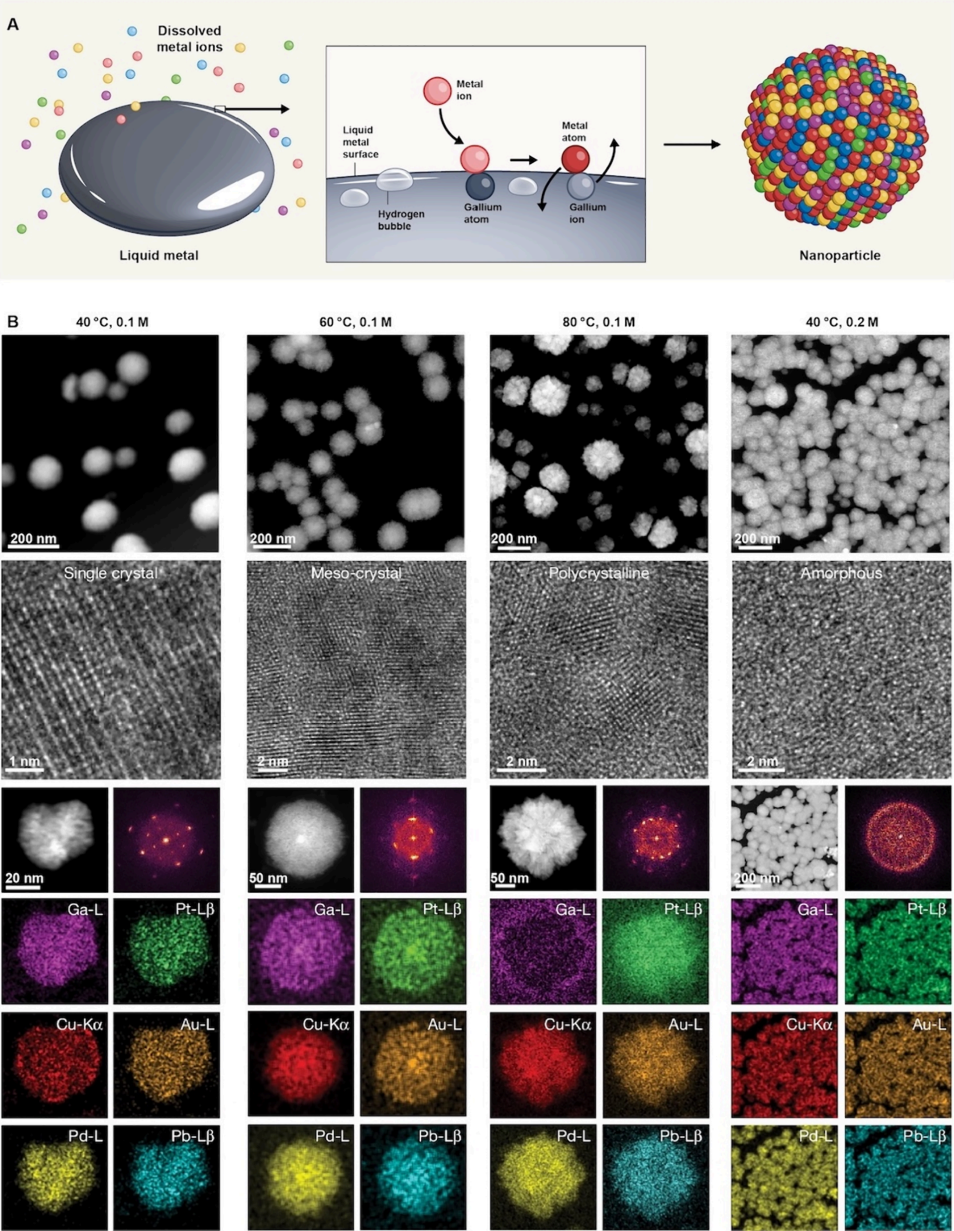
Liquid-metal-mediated synthesis of HEA nanocrystals via isothermal solidification. (A) Schematic of the formation pathway of HEA nanocrystals. (B) Controlled synthesis of GaPtPdPbAuCu HEA nanocrystals with tunable crystallinity and morphology achieved by varying the reaction temperature and the concentration of metal-ion precursors. The image in (A) was reproduced with permission from ref 168. Copyright 2025 Springer Nature. The image in (B) was reproduced with permission from ref 167. Copyright 2025 Springer Nature.

The interfacial replacement reaction can occur under mild conditions, typically between 25–80 °C, and proceeds within seconds to minutes. The rapid kinetics effectively “freeze” the system in a high-entropy configuration, preventing the atoms from ordering. *In situ* TEM has revealed that alloy formation followed an isothermal solidification pathway, driven by hydrogen-bubble-assisted mixing between the Ga-based liquid and the incoming metal atoms. The process consisted of oscillatory crystallization and amorphization, during which crystalline domains continuously emerged and dissolved within milliseconds due to H_2_-bubble-induced mixing. The continuous nucleation–melting cycle inhibited long-range ordering and kinetically trapped the multielement system in a metastable, high-entropy alloy phase.

The degree of crystallinity in the resulting HEAs can be adjusted by tuning the reaction temperature and precursor concentration. Under relatively mild conditions (40 °C and 0.1 M), the foreign metal atoms dissolved into liquid Ga and mixed thoroughly before solidification, yielding highly crystalline HEA nanocrystals, as supported by HRTEM/FFT analysis, SAED from larger particles, and elemental mapping. By contrast, increasing the temperature to 60 °C accelerated solidification and produced a mesocrystalline product rather than a single-crystalline particle: the resulting porous spheres are described as large particles assembled from numerous smaller domains with imperfect crystallographic alignment and local lattice distortion. At 80 °C, faster kinetics favored polycrystalline HEAs with a flower morphology, whereas doubling the metal precursor concentration at 40 °C led to amorphous HEAs because the more rapid influx of foreign atoms promoted swift solidification before ordered crystallization could be established. These results highlight the strong kinetic sensitivity of this liquid–liquid interfacial synthesis (Figure 11B).^167^ Moreover, by varying the type of Ga-based precursor, HEA nanocrystals with distinct shapes and porosities can be obtained. In this synthesis, Ga can either remain in the final alloy or be fully consumed during the reaction, enabling the production of both Ga-containing and Ga-free HEA nanocrystals using the same liquid-metal platform.

Liquid-metal-mediated synthesis takes advantage of the exceptional miscibility and intrinsic reducing power of liquid metal nanoparticles to achieve multielement alloying beyond the limits imposed by conventional solid-state diffusion. The thermal decomposition and hydrogen reduction-based liquid-metal alloying strategy introduced by Cao and co-workers provides strong thermodynamic driving forces and broad elemental compatibility, enabling the formation of compositionally complex HEAs. However, the synthesis is conducted at elevated temperatures (923 K), which restricts control over particle shape or morphology. In contrast, the isothermal solidification route developed by Zhang and co-workers employs low-temperature interfacial reactions to kinetically trap high-entropy states. This method offers better precision in tailoring crystallinity, shape, and compositional uniformity under mild conditions. Nevertheless, its scalability remains a major challenge, as the process has so far been demonstrated only within microscale aqueous droplets. Additionally, the porosity of the resulting HEA nanocrystals is inherently random as a result of uncontrolled hydrogen-bubble evolution during the reaction, making it difficult to control the pore architecture.

#### 3.2.3 Laser Irradiation

For laser irradiation methods, they can directly produce HEA nanocrystals from metal precursors or multiple elemental targets through *in situ* melting, condensation, and nucleation. In these bottom-up approaches, the formation of homogeneous solid-solution phases critically depends on the synergy between rapid heating and ultrafast quenching, which effectively suppresses elemental segregation during particle formation. By contrast, when metallic powders are used as the starting materials, the process should be considered as a top-down route, because the initial feedstock already exists in the metallic state and is subsequently reprocessed into nanoscale products under intense energy input. Therefore, the classification of these methods depends on the nature of the initial material and the formation pathway.

Li and co-workers introduced a continuous-wave visible laser irradiation strategy that utilizes transient laser annealing to create thermal shocks for the synthesis of highly crystalline HEA, high-entropy oxide, and high-entropy nitride nanocrystals.^169^ As shown in Figure 12A, metal salts were first deposited onto a CNF scaffold via drop-casting and drying. The resulting precursor/CNF composite was then subjected to laser irradiation at powers ranging from 2 to 12 W, inducing photothermal melting and alloying under either nitrogen (in a custom-built quartz-window chamber) or ambient air. This rapid irradiation enables precise modulation of both composition and phase structure through control of laser dwell time and power density. For example, an irradiation dwell time of 0.25 ms (corresponding to 1,830 °C) produced AuPdFeCuNi HEA nanocrystals (*ca*. 14 nm) in a single FCC phase (Figure 12B). In contrast, extending the dwell time to 2.5 ms (3,165 °C) led to substantial coarsening, yielding truncated nanocrystals of 30–60 nm that exhibited a mixed FCC and BCC phases. Although laser irradiation enables rapid nonequilibrium alloying, its practical limitations remain significant. Despite its precise spatial control, the method generally suffers from low scalability. In addition, the intense localized heating may damage the substrate and typically yields only microgram-scale products, while control over morphology and surface facets remains limited.

**Figure 12:**
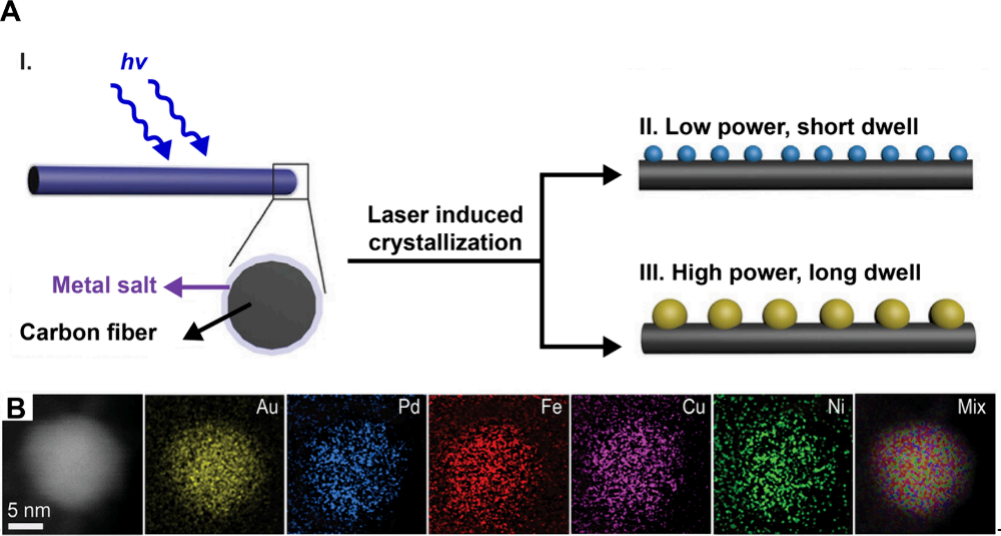
Laser irradiation-assisted synthesis of HEA nanocrystals. (A) Laser irradiation. (B) HAADF-STEM image of quinary AuPdFeCuNi HEA nanocrystals. Reproduced with permission from ref 169. Copyright 2023 Wiley-VCH.

#### 3.2.4 Flame-Based Spray Pyrolysis

Flame-based spray synthesis represents one of the most mature and scalable approaches to producing nanomaterials, and is commonly discussed under flame spray pyrolysis.^170^ A major advantage of this method is its continuous and single-step operation, making it well suited for the high-throughput production of inorganic nanoparticles. Owing to these features, flame-based processes have been widely implemented in industry for the large-scale manufacture of carbon black, fumed silica, titania, and other oxide materials, with reactor capacities reaching the ton-per-hour level. In a typical process, either a precursor solution or a vaporized gaseous precursor is delivered into a high-temperature flame zone. When the precursor is dissolved in a combustible organic solvent, the heat released from solvent combustion can help sustain the flame. In contrast, aqueous precursor solutions generally require an external flame as the main heat source, and this configuration is often referred to as flame-assisted spray pyrolysis. The high-temperature environment then drives rapid decomposition of the precursor(s), followed by nucleation and particle growth in the gas phase or within droplets, ultimately leading to the formation of nanoparticles.

Recently, flame-based spray synthesis has been increasingly extended to the preparation of HEA and high-entry oxide nanocrystals, highlighting its promise as a scalable route for compositionally complex materials. For example, a flame spray pyrolysis strategy has been developed for the continuous-flow synthesis of freestanding HEA nanocrystals (Figure 13A-D), including AuPtPdRuIr (Figure 13E) and other multimetallic systems.^171^ In this approach, the precursor solution undergoes gas shearing and microexplosion, generating nanodroplets that act as confined microreactors. The ultrafast conversion from droplets to sub-10 nm nanoparticles within less than 5 ms enables the formation of binary to septenary alloys under conditions combining extremely high temperatures with ultrafast cooling, facilitating both rapid alloying and kinetic stabilization. This method has also been successfully extended to the synthesis of high-entropy oxide nanocrystals.^172–175^ For instance, single-phase solid-solution quinary high-entropy oxide nanocrystals composed of conventionally immiscible Cu, Mn, Fe, Ni, and Zn oxide species have been synthesized using this route.^172^ In brief, aerosol droplets containing dissolved metal-salt precursors in ethanol are passed through a high-temperature flame zone of approximately 1900 °C, followed by ultrafast quenching. The resulting nanocrystals exhibit a single-phase spinel structure with a high-entropy atomic configuration, demonstrating the effectiveness of this method for stabilizing complex multicomponent oxide nanocrystals under highly nonequilibrium conditions.

**Figure 13:**
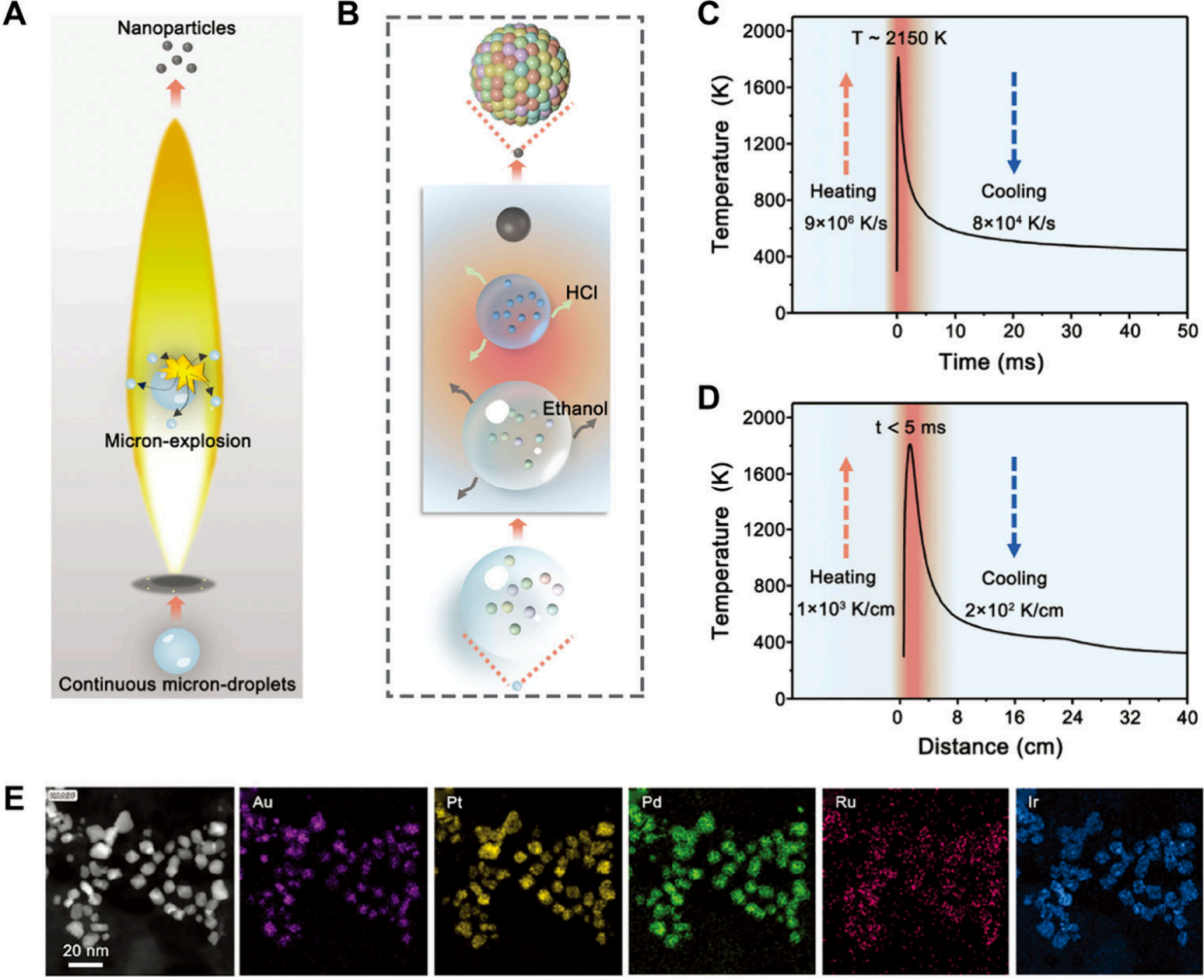
Flame-based spray pyrolysis synthesis of HEA nanocrystals. (A) Flame spray pyrolysis. (B) Droplet-to-particle evolution within a micron-droplet-confined reactor. (C) Temperature–time profile in the flame core. (D) Temperature-distance profile in the flame core. (E) HAADF-STEM image and corresponding EDS maps of AuPtPdRuIr HEA nanocrystals. Reproduced with permission from ref 171. Copyright 2024 Wiley-VCH.

Flame-based spray pyrolysis offers several notable advantages for the synthesis of HEA and high-entropy oxide nanocrystals, including continuous processing, high throughput, surfactant-free products, and ultrafast heating and cooling, which can facilitate the formation of multicomponent phases. These features make it particularly attractive for the scalable production of compositionally complex nanocrystals. However, precise control overs particle size distribution, morphology, and surface facets remain challenging compared with the wet-chemical routes. In addition, compositional uniformity may be influenced by differences in precursor volatility, decomposition kinetics, and transport behavior within the flame. Further refinement of precursor design, flame conditions, and quenching dynamics will therefore be important for advancing this method toward the controlled synthesis of well-defined HEA nanocrystals.

#### 3.2.5 Microwave and Ultrasonication

Microwave irradiation and ultrasonication have emerged as highly efficient, localized energy-driven approaches for the rapid synthesis of HEA nanocrystals. Both techniques deliver concentrated energy to the reaction sites within seconds, inducing extreme nonequilibrium conditions that enable reduction of metal precursors, alloying, and quenching. Unlike CTS methods that depend on direct Joule heating through conductive substrates, microwave and ultrasonication operate wirelessly, transferring energy directly to materials that exhibit strong electromagnetic or acoustic absorption without requiring an external electrical circuit. In microwave heating, oscillating electromagnetic fields generate intense internal friction through dipolar polarization, resulting in uniform volumetric heating across the material. The rapid rise in temperature allows metal salts to decompose and transiently melt, followed by rapid solidification into alloy nanocrystals during cooling. Owing to its speed and homogeneity, microwave processing has been extensively applied to the fabrication of diverse functional materials, including organic compounds,^176^ ceramics,^177^ carbon-based materials,^178,179^ and metal–carbon hybrids.^180,181^

Qiao and co-workers reported an efficient and scalable method for producing HEA nanocrystals with a precise compositional control using reduced graphene oxide (rGO) as a microwave-absorbing substrate.^182^ In their protocol, graphene oxide films were first partially reduced at ca. 570 K under argon (rGO-570) and subsequently impregnated with metal salt precursors via drop-casting. Upon microwave irradiation, the abundant defects and oxygen functionalities on the rGO surface acted as hotspots, rapidly elevating the temperature to ca. 1850 K within seconds while ensuring a uniform distribution of heat (Figure 14A). Under these conditions, they obtained single-phase PtPdFeCoNi HEA nanocrystals of 12 ± 5 nm in average size, together with a homogeneous elemental distribution. This approach offers exceptional versatility as it can be extended to other carbonaceous supports such as one-dimensional CNFs and three-dimensional (3D) porous carbons like carbonized wood.

**Figure 14:**
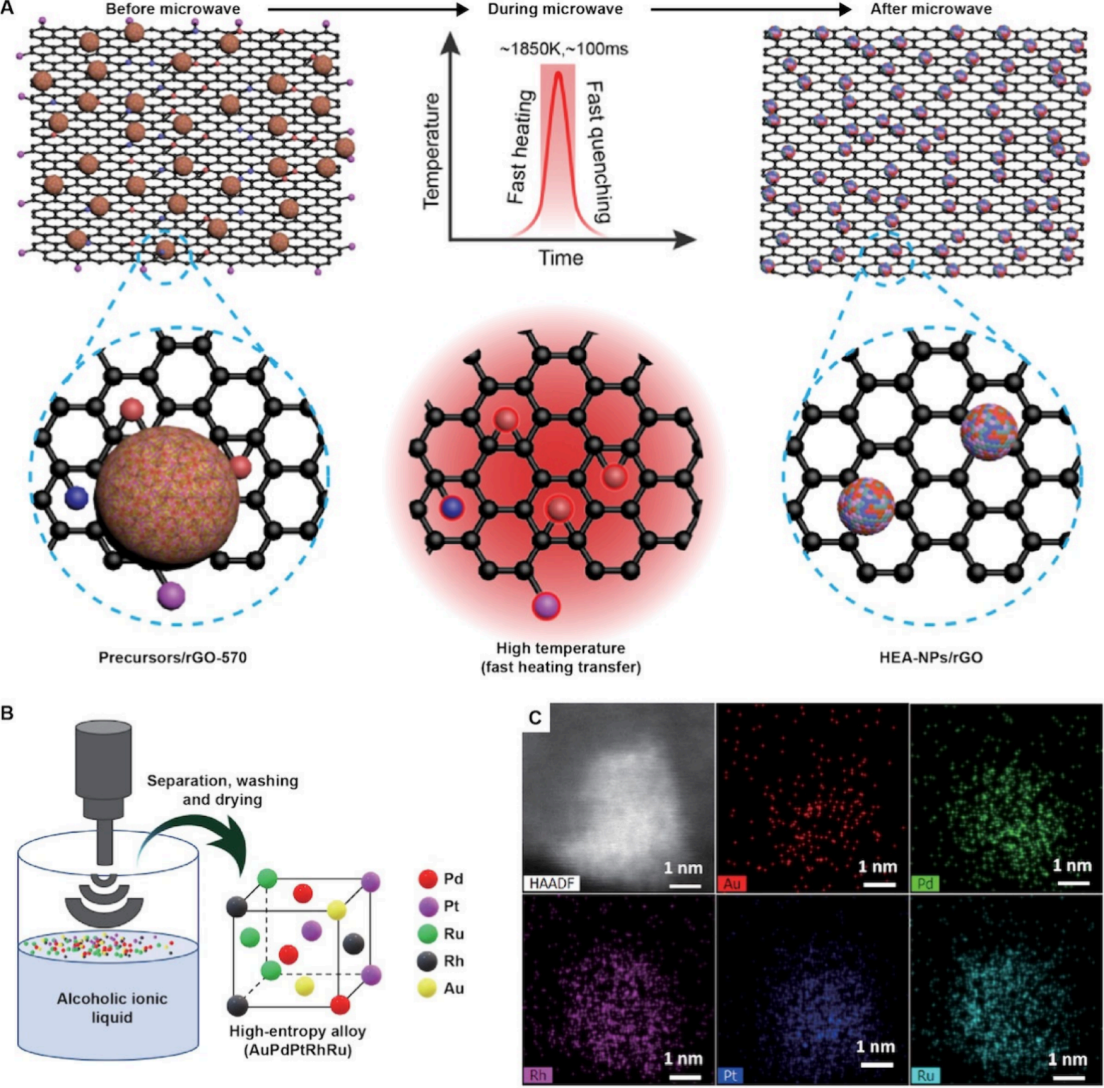
Microwave and ultrasonication-assisted synthesis of HEA nanocrystals. (A) Microwave heating. (B) Ultrasonication. (C) HAADF-STEM image and corresponding EDS maps of a AuPdPtRhRu HEA nanocrystal supported on carbon, which was fabricated using an ultrasonication method. The image in (A) was reproduced with permission from ref 182. Copyright 2021 American Chemical Society. The images in (B, C) were reproduced with permission from ref 185. Copyright 2021 Springer Nature.

The ultrasonication route for synthesizing HEA nanocrystals relies on transient, localized “hotspot” environments generated by acoustic cavitation, which involves the formation, growth, and violent collapse of microscopic bubbles in a liquid subjected to high-frequency ultrasonic irradiation. The implosive collapse of these bubbles produces extreme local conditions, with pressures approaching 2000 atm and temperatures up to 5,000 K, sufficient to drive rapid reduction and alloying reactions in solution.^183,184^ Okejiri and co-workers demonstrated a simple ultrasound-assisted strategy for fabricating HEA nanocrystals by using alcoholic ionic liquids as both solvent and reducing agent.^185^ As shown in Figure 14B, an aqueous solution of metal precursors was mixed with a predispersed carbon support in 30 mL of the alcoholic ionic liquids, N-(2-hydroxyethyl)-*N*-methylmorpholinium bis­(trifluoromethylsulfonyl)­imide. The resulting suspension was subjected to ultrasonication using a titanium horn operating at 20 kHz for 10 min under ambient conditions. The intense ultrasound-induced cavitation promoted *in situ* reduction of Au^3+^, Pd^2+^, Pt^2+^, Rh^3+^, and Ru^3+^ ions and alloying of their atoms, yielding single-phase FCC AuPdPtRhRu HEA nanocrystals without requiring postsynthesis calcination (Figure 14C).

Microwave and ultrasonication methods provide rapid, energy-driven bottom-up routes to HEA nanocrystal synthesis; both still face major challenges in terms of control. Microwave synthesis enables extreme temperatures and rapid quenching, yet remains limited by substrate response, heat-transfer efficiency, and poor morphological control. Ultrasonication-assisted synthesis proceeds under milder conditions, but stochastic cavitation, uneven reduction kinetics, and agglomeration often lead to a heterogeneous particle population. In addition, prolonged sonication may degrade ionic liquids or generate reactive radicals, causing surface destruction or contamination. As a result, both methods offer limited controls over facet exposure and crystal phase, which constrains their use in systematic catalytic studies.

#### 3.2.6 Hydrogen-Assisted Reduction

The hydrogen-reduction-assisted synthesis of HEA nanocrystals involves thermally treating a mixture of multiple metal precursors under a reducing atmosphere of hydrogen. Upon heating, hydrogen molecules dissociate into atomic hydrogen, which serves as a strong reducing agent to convert metal ions to their metallic forms. The simultaneous reduction facilitates the co-nucleation and atomic-level mixing of different metal species, enabling the direct formation of single-phase, solid-solution HEA nanocrystals. The high temperature offers sufficient atomic mobility for interdiffusion, while the hydrogen atmosphere prevents oxidation, allowing for the formation of compositionally homogeneous and phase-pure HEA structures without the need for additional chemical reducing agents.

Zhao and co-workers recently combined hydrogen-assisted reduction with spray drying to synthesize single-phase Pt-based HEA nanocrystals containing up to ten elements.^118^ In their method, metal precursors were first atomized and dried onto a carbon support. The sample was then subjected to hydrogen reduction under a moderate heating rate (3 °C min^–1^) at decomposition temperatures ranging from 300–850 °C, followed by slow cooling (5–10 °C min^–1^). A key feature of this method lies in the autocatalytic behavior of Pt, which facilitates the reduction of other metal ions at lower temperatures. This autocatalytic effect enables the formation of HEA nanocrystals at temperatures as low as 300 °C, offering a general and low-temperature route to compositionally complex alloys with homogeneous mixing.

Mori and co-workers also developed a hydrogen-spillover-mediated strategy to synthesize HEA nanocrystals on reducible oxide supports at low temperatures.^89^ In this approach, TiO_2_–supported Pd nuclei catalyzed H_2_ dissociation, generating spillover hydrogen that migrated across the TiO_2_ surface via coupled proton–electron transfer. These mobile hydrogen species simultaneously reduced multiple metal precursors (Co^2+^, Ni^2+^, Cu^2+^, Ru^3+^, Pd^2+^) at *ca*. 400 °C, promoting homogeneous alloying and yielding 2 nm CoNiCuRuPd HEA nanocrystals (Figure 15A). Specifically, the synthesis involved depositing equimolar metal salts onto TiO_2_, followed by reduction under flowing H_2_ (20 mL min^–1^) at 400 °C for 2 h. Temperature-programmed reduction (TPR), as shown in Figure 15B, revealed a single broad peak around 170 °C, confirming concurrent reduction of all components and strong coupling between the elements. The formation proceeded through sequential steps: partial reduction of Pd^2+^ to metallic Pd, H_2_ dissociation on Pd to form Pd–H species, hydrogen spillover to the TiO_2_ surface accompanied by Ti^4+^→ Ti^3+^ reduction, proton–electron migration through the oxide lattice, and synchronized reduction of all metal ions to form a uniform HEA phase (Figure 15C). Hydrogen-reduction-assisted strategies offer a versatile route to synthesizing HEA nanocrystals by simultaneously reducing multiple metal precursors and promoting atomic-level mixing under a reductive atmosphere. These methods are well-suited for producing single-phase HEAs with uniform elemental distributions without additional chemical reducing agents. However, the resultant nanocrystals often display spherical or irregular morphologies because the elevated temperatures favor rapid atomic diffusion and thermodynamically stable shapes over facet-controlled or anisotropic structures.

**Figure 15:**
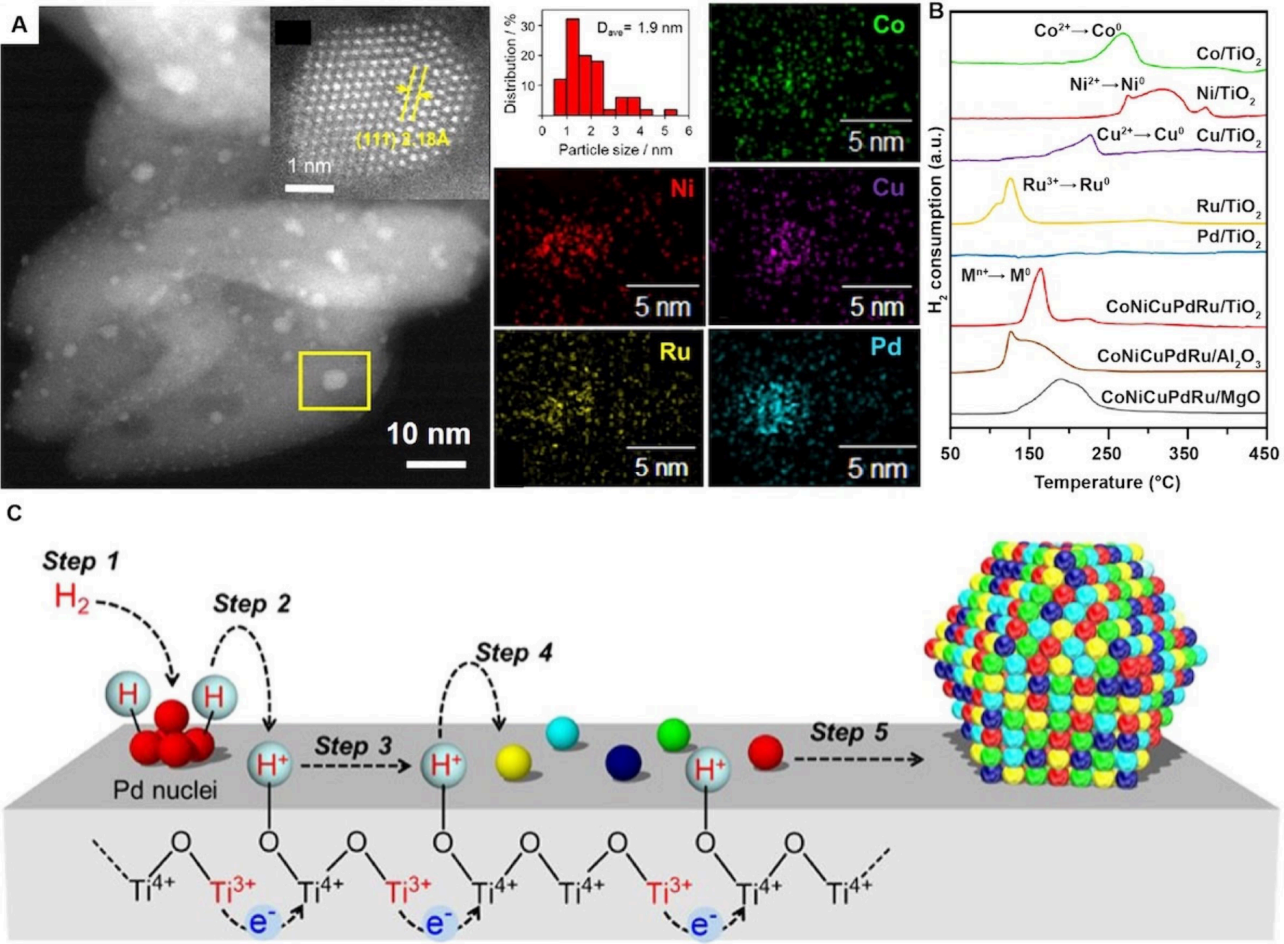
Hydrogen-assisted reduction synthesis of HEA nanocrystals. (A) HAADF-STEM images, particle size distribution, and EDS elemental mapping of CoNiCuRuPd nanocrystals supported on TiO_2_. (B) H_2_-TPR profiles of mono- and quinary-component samples deposited on TiO_2_, Al_2_O_3_, and MgO. (C) Schematic of the formation pathway of HEA nanocrystals on TiO_2_ via hydrogen-spillover-driven synthesis. Reproduced with permission from ref 89. Copyright 2021 Springer Nature.

#### 3.2.7 Electrochemical Deposition

Electrodeposition provides a versatile route to the synthesis of HEA nanocrystals through the reduction of multiple metal ions on a conductive substrate. By tuning experimental parameters, such as applied potential, current density, pH, precursor ratio, and electrolyte composition, metals with distinct reduction potentials can be driven to nucleate and alloy into a single-phase solid solution. In confined spaces or pulsed systems, including nanodroplet electrosynthesis and electrochemical-shock methods, ultrafast reduction and limited atomic diffusion kinetically trap atoms into disordered, high-entropy configurations. The alloy composition, crystallinity, and particle size are determined by factors such as potential waveform, ion concentration, and mass-transport dynamics, enabling controllable synthesis spanning from amorphous metallic glasses to crystalline HEAs.

Glasscott and co-workers introduced a nanodroplet-mediated electrodeposition strategy to produce high-entropy metallic glass nanocrystals comprising up to eight equimolar elements (Co, Cr, Cu, Gd, In, Mn, Ni, and V, Figure 16).^114,186^ In this method, aqueous nanodroplets (*ca*. 450 nm in radius) containing multiple metal salts are dispersed in 1,2-dichloroethane and stochastically collide with a cathodically biased electrode (−1.5 V vs Ag/AgCl). Upon impact, each droplet undergoes an instantaneous electro-shock reduction (*ca*. 100 ms), leading to complete coreduction of all confined metal ions and formation of amorphous, compositionally uniform alloy nanocrystals. The confined microreactor environment ensures nearly quantitative precursor reduction (>98%), minimal interdroplet diffusion, and precise stoichiometric control (within 2–5%) by adjusting the precursor concentrations. Theoretically, the method exploits confined electrochemical reduction kinetics analogous to nanosecond thermal shocks, producing a nonequilibrium alloy characterized by high configurational entropy. The resulting metallic-glass-like structure originates from rapid nucleation coupled with inhibited atomic diffusion, effectively suppressing crystallization and phase segregation.

**Figure 16:**
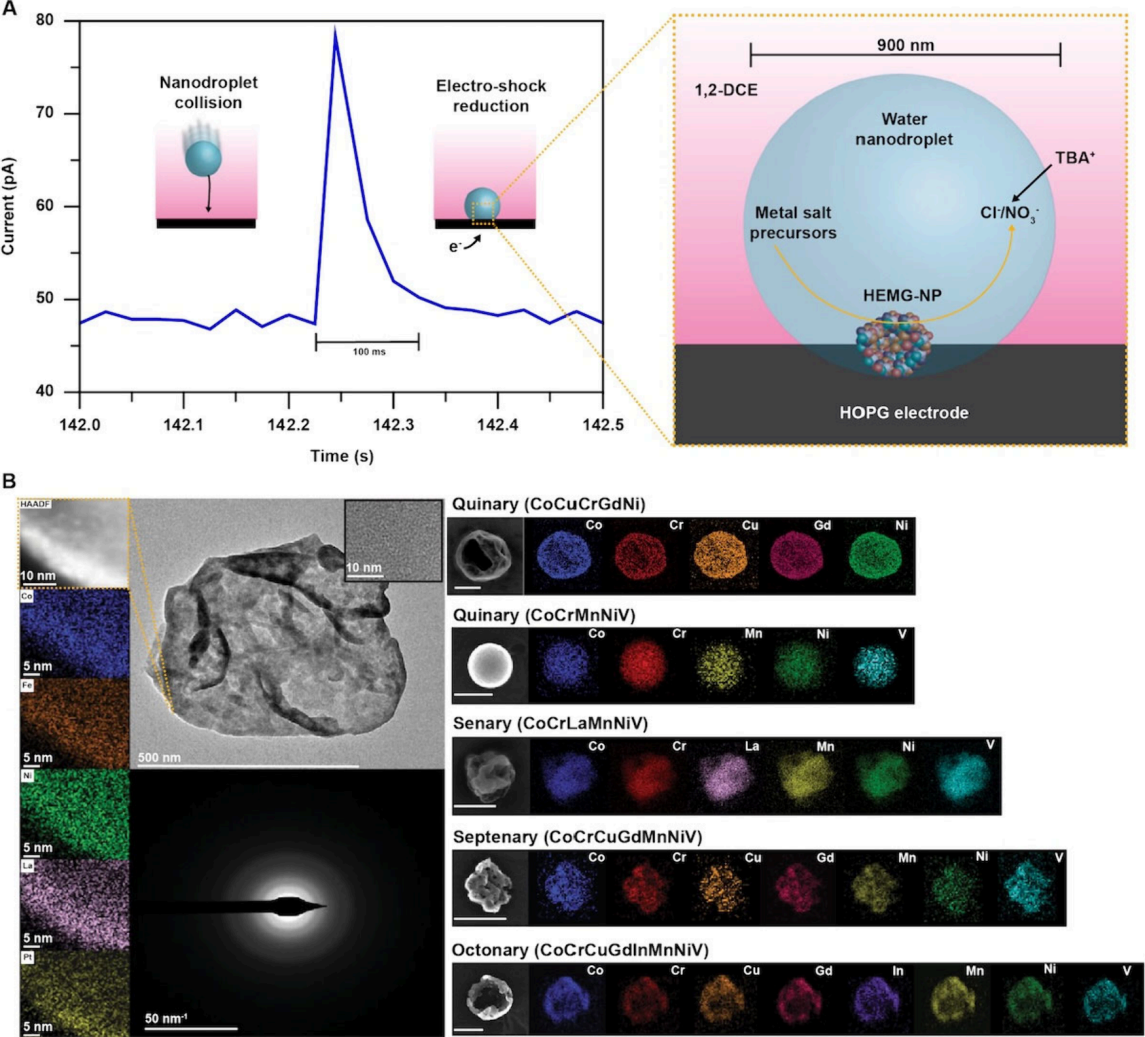
Electrodeposition synthesis of metallic glass HEA nanocrystals. (A) Schematic illustration depicting the formation pathway of metallic glass HEA nanocrystals. (B) TEM and EDS elemental mapping of metallic glass HEA nanocrystals produced by incorporating 5–8 different metal salt precursors into the nanodroplets. All nanocrystals were synthesized under an applied potential of −1.5 V vs Ag/AgCl. Scale bars correspond to 500 nm unless otherwise specified. Reproduced with permission from ref 114. Copyright 2019 Springer Nature.

Liu and co-workers reported the electrochemical synthesis of PdAgAu-based alloy nanocrystals directly on glassy carbon rotating-disk electrodes, enabling systematic investigation of composition-dependent catalytic behavior across 107 unique samples.^187^ The nanocrystals, with diameters ranging from 50–100 nm, were deposited using three distinct electrochemical protocols, potentiostatic, galvanostatic, and pulsed deposition, within a three-electrode cell containing a 0.1 M Na_2_SO_4_ electrolyte mixed with metal precursors. Typical deposition potentials were maintained between −0.6 and −0.7 V vs Ag/AgCl, with a deposition time of approximately 60 s. The addition of NH_4_OH facilitated the formation of soluble [Ag­(NH_3_)_2_]^+^ complexes, preventing AgCl precipitation while ensuring uniform coreduction of all metal species. Among the tested methods, pulsed electrodeposition produced the most homogeneous alloy films, owing to its alternating reduction (−0.65 V) and relaxation (0 V) cycles that promote controlled nucleation and growth.

Although electrodeposition offers a low-temperature and substrate-integrated route for HEA nanocrystal synthesis, it has limited controls over facet exposure, morphology, and size uniformity. Stochastic nucleation, mass-transport gradients, and potential fluctuations often lead to polycrystalline or aggregated deposits. Achieving uniform, facet-engineered HEA nanocrystals will therefore require more advanced strategies, such as template-directed growth, pulse waveform optimization, and confined electrochemical environments.

### 3.3 Wet-Chemical Synthesis

In contrast to ultrafast thermal methods that depend on external energy inputs such as carbothermal shock, laser irradiation, or arc discharge to trigger rapid nucleation, alloying, and phase transition, wet-chemical synthesis is governed by solution-phase reduction. Through the use of molecular reductant(s) combined with stabilizer(s) or ligand(s), this method allows for precise regulation of metal precursor reduction kinetics and interfacial chemistry. These characteristics make wet-chemical synthesis, particularly in the colloidal system, a powerful platform for simultaneously tailoring the composition and surface structure, including crystal facets, of HEA nanocrystals. This strategy builds on over three decades of development in the synthesis of mono- and bimetallic nanocrystals. Key principles related to thermodynamic and kinetic controls have been adapted to address the added complexity of multicomponent systems. Although the conversion of precursors into nanocrystals may seem straightforward, each stage, including precursor reduction, nucleation, growth, and atomic rearrangement through diffusion, critically influences the final elemental distribution, crystal phase, and surface structure of HEA nanocrystals. In the following sections, we begin with a concise overview of the nucleation and growth mechanisms of conventional metal nanocrystals. We then introduce several recent strategies and fundamental design principles that enable precise controls over the composition, facet exposure, and atomic-level uniformity of compositionally complex HEA nanocrystals.

#### 3.3.1 Nucleation and Growth Mechanisms

The formation of metal nanocrystals begins with the reduction (thermal decomposition, or both) of metal precursors, which are typically salts in high oxidation states.^59,67^ In most colloidal syntheses, chemical reduction is preferred due to its superior tunability.^188^ The rate of metal atom generation can be precisely adjusted through the selection of reductant, the concentrations of the precursors and reductant, and the reaction temperature. Compared with thermal decomposition, where precursor activation is primarily temperature-controlled and can accelerate sharply over a narrow temperature range, chemical reduction enables finer control over the timing and extent of metal atom generation through the choice of reductant and coordination chemistry.^188^ This allows more reliable separation of nucleation and growth and is therefore well suited for multimetallic systems that require synchronized reduction to achieve homogeneous alloying. For example, noble-metal precursors such as Pt­(IV), Pd­(II), and Au­(III) generally possess high reduction potentials and can be readily reduced at moderate temperatures. In contrast, base metal precursors like Fe­(III), Co­(II), and Ni­(II) exhibit much lower reduction potentials, often necessitating thermal decomposition routes at elevated temperatures to generate atoms.^189–191^ This high-temperature requirement introduces challenges in maintaining shape control, particularly for regulating exposed crystal facets, which are sensitive to temperature-induced diffusion and coarsening. A key difficulty in wet-chemical synthesis of HEA nanocrystals lies in synchronizing the reduction rates of multiple metal precursors with vastly different redox potentials. Mismatched reduction kinetics can lead to phase segregation, compositional inhomogeneity, or undesired morphological evolution. Thus, achieving compositional uniformity and morphological precision in HEA systems requires careful balancing of reduction conditions, often through the use of tailored reductants or stepwise precursor addition strategies.^24,192^

Following the generation of metal atoms, nucleation begins once their concentration in the solution reaches the critical supersaturation threshold. This process is well described by the LaMer model, which outlines three sequential stages that govern the formation of nanocrystals of a uniform size, as illustrated in Figure 17A.^58^ In the initial stage, metal atoms are gradually produced through the reduction or decomposition of precursors, leading to an increase in their concentration over time. Once this concentration exceeds the minimum required for nucleation, known as the critical concentration, the second stage begins. A burst of homogeneous nucleation occurs rapidly, consuming a significant portion of the metal atoms and thereby sharply reducing their concentration. This nucleation burst is brief but crucial, as it defines the number of nuclei and minimizes the variation in particle size. In the final stage, the remaining precursors continue to reduce, and the newly generated atoms are added to the preformed seeds, leading to crystal growth without triggering further nucleation. The LaMer model emphasizes that temporal separation between nucleation and growth is essential for achieving monodisperse products. If nucleation recurs during growth, a broad size distribution will result, since seeds formed at different time points undergo unequal growth durations. To prevent this, synthetic protocols often employ strategies such as dropwise addition of precursors or using reductants with low reduction powers. These approaches help maintain the concentration of metal atoms within a window that supports seed growth while suppressing new nucleation events. Beyond homogeneous nucleation, heterogeneous nucleation is often favored, in which new nanocrystals form on pre-existing seed surfaces.^193^ Compared with homogeneous nucleation, the nucleation barrier (ΔG*) is reduced because the nucleus forms on a seed surface (e.g., a preformed nanocrystal),^188^ which also decreases the critical nucleus size and promotes site-specific nucleation (Figure 17B). As described by classical nucleation theory, the wetting behavior is governed by the interfacial energies among the nucleus, the surrounding medium, and the seed surface, and is commonly quantified by the contact angle (Figure 17C). After nucleation concludes, the growth stage dictates the size, shape, and surface structure of the resulting nanocrystals. Growth proceeds through two principal mechanisms: solution-phase reduction and surface-catalyzed reduction (Figure 17D).^194^ In the solution-phase pathway, metal precursors are reduced in the bulk solution to generate free atoms, which subsequently diffuse and deposit onto the surfaces of nanocrystal seeds. This mechanism is predominant when the reduction rate is high and the precursor is abundant, often leading to symmetric growth and relatively uniform nanocrystals. In contrast, surface-catalyzed (autocatalytic) growth involves the adsorption of precursors on nanocrystal, where reduction is facilitated by the catalytic activity of surface atoms. This route is favored under slower reduction kinetics and plays a pivotal role in directing asymmetric growth by accelerating atom addition at selective crystallographic facets. The reduction barrier is significantly lowered at the surface of a growing nanocrystal, enabling preferential development along certain directions that give rise to high-index or asymmetrical morphologies. A crucial aspect of the growth process is the competition between kinetic and thermodynamic controls.^67^ Under kinetic control, rapid atom deposition and limited surface diffusion can result in branched, uneven, or metastable nanocrystal shapes. Thermodynamic control, in contrast, allows atoms to rearrange into energetically favorable configurations. Rather than being a mere extension of nucleation, the growth stage represents a critical window for engineering nanocrystals at the atomic level. Precise regulation of reduction pathway, surface energetics, and surface-ligand interaction provides powerful leverage for tailoring the morphology and type of facets exposed on the surface of HEA nanocrystals.

**Figure 17:**
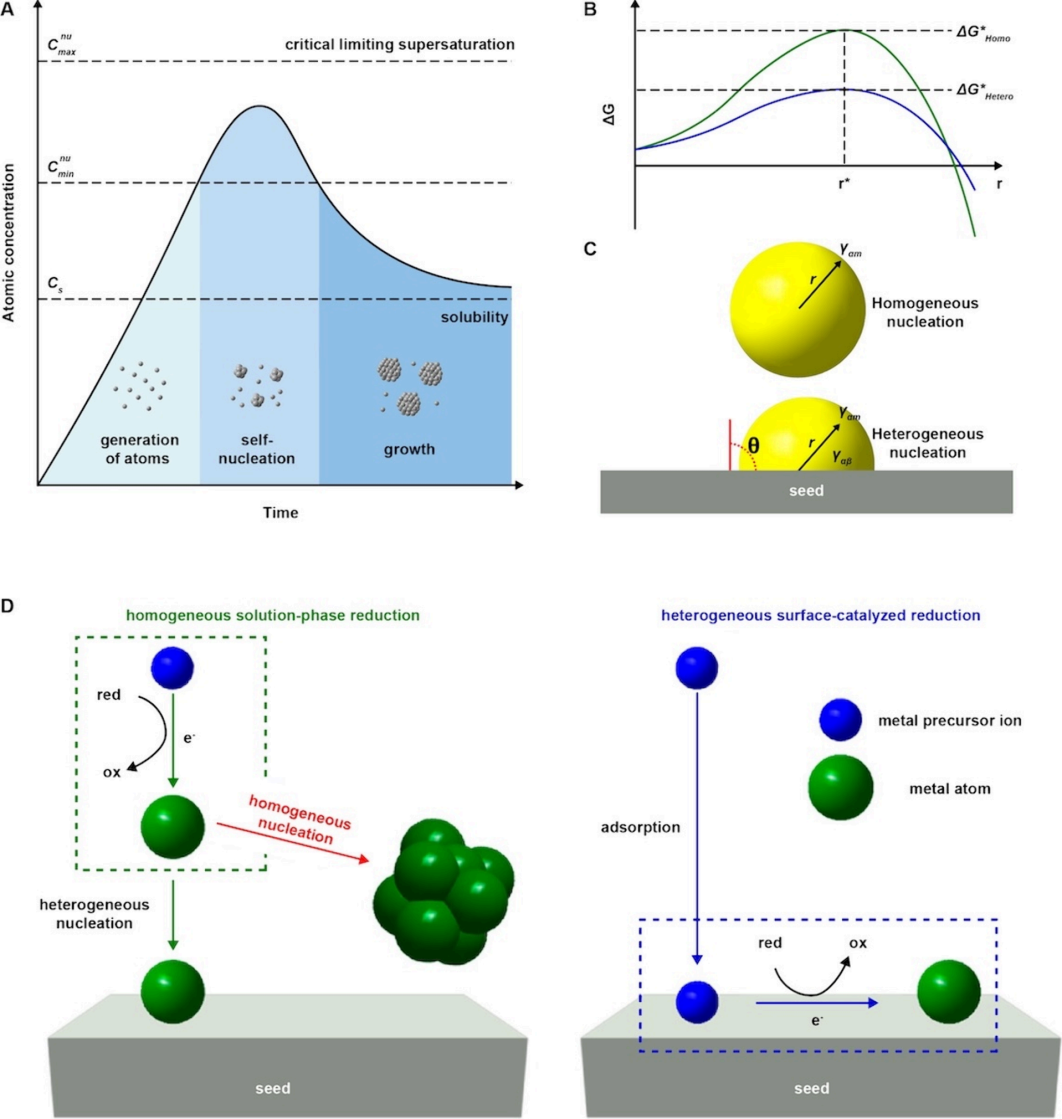
Nucleation and growth mechanisms in wet-chemical synthesis. (A) LaMer model illustrating atomic concentration evolution through generation, nucleation, and growth. (B) Comparison of homogeneous and heterogeneous nucleation energy barriers. (C) Schematic comparison of homogeneous nucleation in the solution and heterogeneous nucleation on the surface of a seed. (D) Schematic illustration of two primary growth pathways: solution-phase reduction and surface-catalyzed reduction. The image in (A) was reproduced with permission from ref 58. Copyright 2008 Wiley-VCH. The images in (B, C) were reproduced with permission from ref 193. Copyright 2016 Wiley-VCH. The image in (D) was reproduced with permission from ref 194. Copyright 2017 American Chemical Society.

#### 3.3.2 One-Shot vs Dropwise Addition of Precursor Solution

Both the formation pathway and compositional homogeneity of HEA nanocrystals are intimately tied to the reduction kinetics of their individual metal precursors. When metal salts with disparate redox potentials are introduced simultaneously, as in the conventional one-shot injection approach, the reduction often proceeds in a sequential rather than simultaneous fashion. This often leads to preferential nucleation of more readily reducible species and, consequently, phase segregation or formation of a core–shell structure instead of a homogeneous alloy. By contrast, the dropwise addition strategy, where metal precursors are introduced gradually into the reaction mixture, offers a tight control over the reduction kinetic.^78,80,82,195–197^ The temporal modulation ensures that the concentrations of all metal species remain low and steady throughout the process, minimizing the risk of rapid precursor depletion and enabling synchronized reduction kinetics among components with disparate redox potentials. Such conditions greatly enhance the likelihood of concurrent co-nucleation, which is crucial for achieving a uniform elemental distribution and generating single-phase solid-solution HEA nanocrystals. Notably, under proper conditions, the dropwise approach can establish a steady-state regime in which the quantity of each metal precursor present in the solution between successive droplet additions remains constant. The steady-state kinetics allows the system to generate comparable numbers of atoms from different precursors at each time point, promoting homogeneous nucleation and growth of compositionally uniform nanocrystals.

The reduction of a metal precursor ion (M^x+^) in solution typically involves a bimolecular electron transfer between the precursor and a reductant molecule, following a second-order rate law. However, in practice, the reductant is often present in large excess, allowing the reaction to be approximated by pseudo-first-order kinetics:

2 $$\mathrm{rate}=k^{\prime }\left[M^{x+}\right]\left[\mathrm{reductant}\right]=k\left[M^{x+}\right]$$

Here, *k* represents an effective first-order rate constant. Integration of this rate law yields an exponential decay function:

3 $$\ln{\left[M^{x+}\right]}_{t}=-kt+\ln{\left[M^{x+}\right]}_{0}$$

This expression illustrates that the precursor concentration decreases exponentially with time (Figure 18A),^196^ and the rate constant *k* can be experimentally extracted by plotting ln [M^x+^]_*t*_ against time and fitting the data to a linear model.

**Figure 18:**
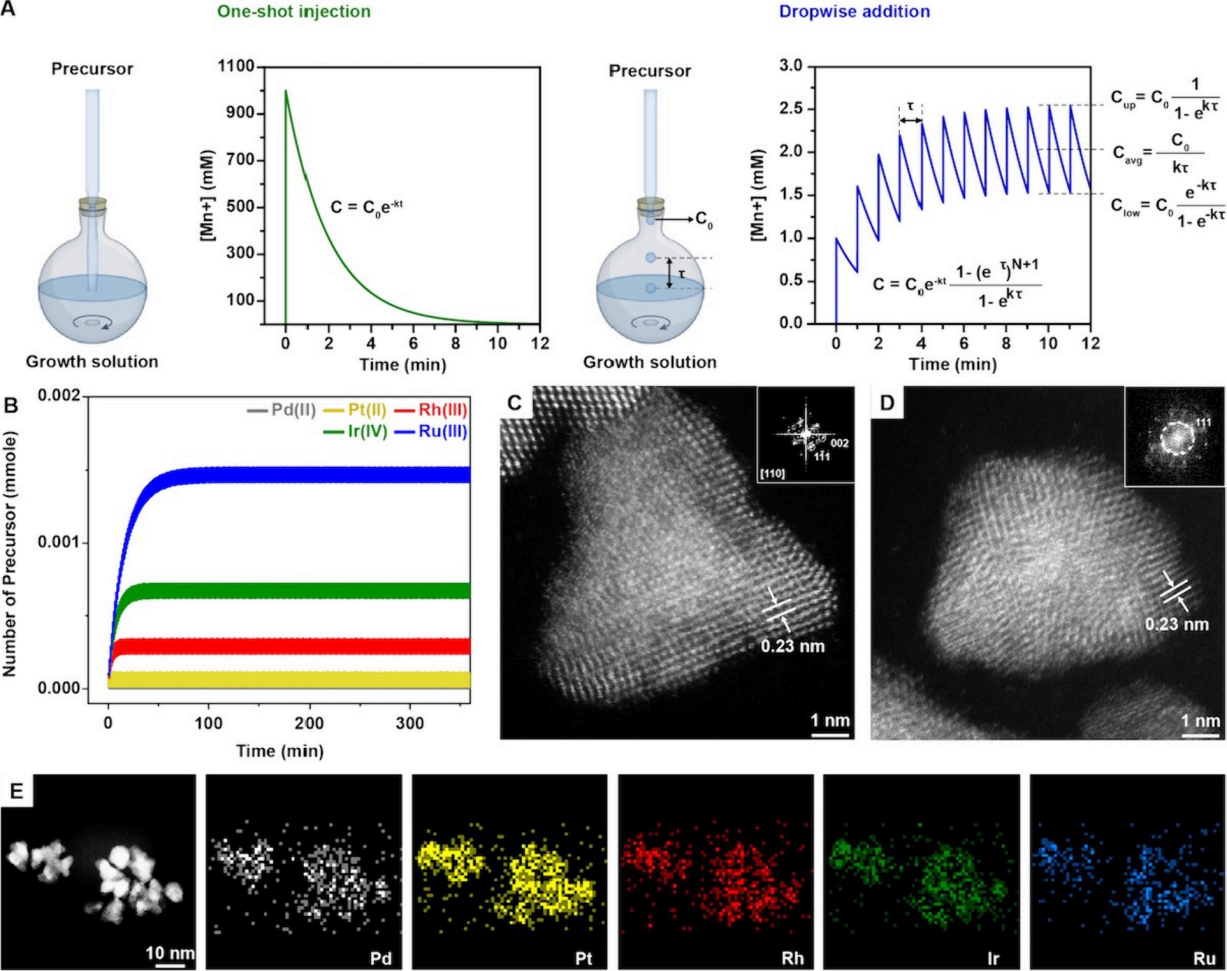
Comparison of one-shot injection and dropwise addition strategies for HEA synthesis. (A) Schematic and kinetic models illustrating precursor concentration evolution in one-shot and dropwise addition systems. (B) Simulated concentration profiles of Pd­(II), Pt­(II), Rh­(III), Ir­(IV), and Ru­(III) precursors during reduction in the dropwise process. (C, D) HAADF-STEM images of representative PdPtRhIrRu nanocrystals synthesized via the dropwise strategy. (E) EDS elemental maps of Pd, Pt, Rh, Ir, and Ru for the dropwise-synthesized HEA PdPtRhIrRu nanocrystals. The image in (A) was reproduced with permission from ref 196. Copyright 2024 Springer Nature. The images in (B–E) were reproduced with permission from ref 78. Copyright 2023 AAAS.

When applying a dropwise strategy, the system can be described as a series of discrete additions of precursor aliquots into the reaction solution (Figure 18A).^196^ Each droplet contains a fixed number of precursor ions *n*_*0*_, introduced at a constant time interval τ. The total precursor population *n*_*t*_ present in the solution at time *t* can be described as a summation of exponentials due to staggered initiation of reduction:

4 $$\begin{array}{ll} n_{t} & =n_{0}e^{-kt}+n_{0}e^{-k\left(t-\tau \right)}+n_{0}e^{-k\left(t-2\tau \right)}+\cdot \cdot \cdot +n_{0}e^{-k\left(t-N\tau \right)}\\ & =\frac{n_{0}e^{-kt}\times \left(1-{\left(e^{k\tau }\right)}^{N+1}\right)}{1-e^{k\tau }} \end{array}$$

Where *N* corresponds to the total number of droplets added. This mathematical framework captures the temporal evolution of precursor concentration under dropwise addition. It reveals that a steady-state concentration of metal ions can be achieved when the reduction rate *k*, droplet frequency τ, and dosage *n*_*0*_ are properly tuned. By tuning *n*_*0*_, τ, and *k*, the system can be tuned to achieve a steady-state regime in which the concentration of precursor ions oscillates within a narrow range. Specifically, this fluctuation occurs between a lower bound (*n*_*low*_ *=* $\frac{n_{0}e^{-k\tau }}{1-e^{-k\tau }}$ ) and an upper bound (*n*_*up*_ *=* $\frac{n_{0}}{1-e^{-k\tau }}$ ), ensuring consistent metal atom generation across all components. Each droplet addition transiently elevates the precursor concentration from *n*_*low*_ to *n*_*up*_, followed by a pseudo-first-order exponential decay due to reduction, returning the concentration back to *n*_*low*_ before the next droplet arrives. The difference between the upper and lower limits is defined by *n*_*0*_, and the rate constant *k* can be experimentally determined through kinetic modeling. This approach facilitates a steady state in which the input and consumption of precursors are balanced, minimizing temporal concentration spikes that may favor one metal over others. Consequently, each droplet contributes equally to the formation of new nuclei and the subsequent growth process, promoting uniform atomic incorporation and suppressing element-specific reduction dominance during the early stages of HEA nanocrystal formation.

To quantitatively validate the kinetic framework described above, Yang and co-workers investigated the reduction behaviors of five noble-metal precursors, Pd­(II), Pt­(II), Rh­(III), Ir­(IV), and Ru­(III), during the one-shot synthesis of phase-segregated PdPtRhIrRu nanocrystals.^78^ The reduction rate constants of these metal ions span nearly 2 orders of magnitude, leading to highly asynchronous nucleation. The reduction hierarchy follows the order Pd­(II) > Pt­(II) > Rh­(III) > Ir­(IV) > Ru­(III), where rapid Pd­(II) reduction initiates early core formation, while Ir­(IV) and Ru­(III) were reduced more slowly, producing compositional gradients characterized by a Pd-enriched core and an outer shell enriched in Pt, Rh, Ir, and Ru. This sequential reduction behavior, confirmed by time-resolved kinetic analysis and elemental mapping, exemplifies how uncontrolled precursor depletion in a one-shot process favors phase segregation over atomic-level mixing. These findings highlight the inherent limitation of the one-shot injection approach for achieving homogeneous alloying when the metal precursors possess markedly different reduction kinetics.

To mitigate the kinetic disparity inherent in one-shot injection systems, dropwise addition has emerged as an effective strategy for achieving steady-state precursor concentrations during HEA synthesis. In this approach, solutions containing multiple metal precursors are introduced gradually into the reaction medium, allowing the instantaneous concentrations of each ionic species to remain low and comparable throughout the process. This temporal modulation ensures synchronized reduction events among metals with dissimilar redox potentials, thereby promoting concurrent co-nucleation and homogeneous alloying rather than sequential deposition. By fine-tuning the injection interval, precursor concentration, and temperature, the system can reach a dynamic equilibrium between precursor supply and consumption, effectively suppressing phase segregation. For instance, a representative study on the dropwise synthesis of PdPtRhIrRu nanocrystals conducted at 180 °C, using an injection dosage *n*_*0*_ of 1.045 × 10^–4^ mmol and a droplet interval τ of 68.4 s, demonstrated that maintaining a nearly 1 min interval between precursor additions enabled the system to enter a steady-state regime, where the supply and reduction of precursor ions became kinetically balanced.^78^ Under these conditions, the more reducible Pd­(II) and Pt­(II) species rapidly attained constant concentrations, while the slower-reducing Rh­(III), Ir­(IV), and Ru­(III) precursors required extended durations of 12, 27, and 80 min, respectively, to achieve similar steady-state conditions (Figure 18B). TEM revealed uniformly dispersed nanocrystals with an average size of a few nanometers, while HAADF-STEM imaging and fast Fourier transform (FFT) analysis confirmed an FCC structure characteristic of single-phase solid-solution HEAs (Figure 18C, D). Complementary EDS mapping further verified the absence of phase segregation and the nearly equiatomic distribution of Pd, Pt, Rh, Ir, and Ru throughout each particle (Figure 18E). Collectively, these findings establish dropwise addition as a robust kinetic control strategy for multicomponent alloy systems. By maintaining steady precursor flux and synchronizing reduction among metals with divergent redox potentials, this method effectively bridges the gap between thermodynamic mixing and kinetic accessibility, enabling the reproducible formation of compositionally uniform HEA nanocrystals. Recent investigations have extended this precursor modulation strategy to the synthesis of multimetallic nanocrystals comprised of noble metals, 3d transition metals, and even p-block elements. These advances underscore the broad applicability of kinetic regulation in facilitating multicomponent reduction and significantly widening the accessible compositional space for synthesizing solid-solution HEA nanocrystals.

#### 3.3.3 Deposition vs Surface Diffusion

Controlling the surface structure of metal nanocrystals is essential not only for tuning their catalytic performance but also for maximizing the atomic utilization efficiency. A critical factor governing the particle shape or morphology during growth is the interplay between the atom deposition rate (*R*_*dep*_) and the surface diffusion rate (*R*_*diff*_). These two kinetic parameters, which are primarily controlled by the injection rate of the precursor solution and reaction temperature, respectively, collectively determine whether adatoms remain near their initial landing sites or migrate across the surface to energetically more favorable positions before being incorporated into the crystal lattice. When the deposition rate significantly exceeds the surface diffusion rate (*R*_*dep*_ > *R*_*diff*_), atoms are likely to be immobilized upon arrival, promoting anisotropic growth modes such as branched or dendritic morphologies, or leading to the formation of isolated islands. Conversely, when surface diffusion is dominant (*R*_*dep*_ < *R*_*diff*_), adatoms have sufficient mobility to redistribute over the nanocrystal surface, favoring thermodynamically controlled growth and the development of compact, low-energy morphologies. The critical role played by the *R*_*dep*_/*R*_*diff*_ ratio in shaping nanocrystals was systematically investigated by Xia and co-workers in their study on seed-mediated growth of Pd nanocrystals.^198^

The deposition–diffusion interplay plays an equally pivotal role in shaping HEA nanocrystals, where structural and compositional complexity further amplifies the sensitivity to kinetic control. In particular, recent studies on the dropwise synthesis of PdPtRhIrRu nanocrystals at 180 °C demonstrated that tuning the injection interval τ of the precursor solution from 68.4 to 11.4 s, while maintaining a constant injection dosage of *n*_0_ at 1.045 × 10^–4^ mmol, effectively increased *R*_*dep*_ without altering *R*_*diff*_. This shift elevated the *R*_*dep*_/*R*_*diff*_ ratio, thereby suppressing the surface mobility of adatoms and favoring localized, site-specific growth. As a result, the morphology transitioned from compact, thermodynamically favored shapes to kinetically trapped dendritic structures with a higher density of low-coordination surface atoms (Figure 19A).^78^ The morphological consequences of this kinetic tuning are clearly reflected in the resulting HEA nanocrystals. Both TEM and HAADF-STEM revealed uniform particle size distributions after 6 h of dropwise synthesis. Lattice fringes with a spacing of 0.23 nm corresponded to the (111) plane of an FCC structure, which was further confirmed by FFT analysis along the [110] direction. Complementary XRD confirmed the single-phase FCC nature, while EDS mapping demonstrated homogeneous elemental distributions across individual particles, validating the formation of a solid-solution HEA phase (Figure 19B–I).^78^ Collectively, these findings illustrate how a precise control over the *R*_*dep*_/*R*_*diff*_ ratio, achieved via modulation of precursor injection parameters, can be leveraged to tailor the morphology in multimetallic systems. Promoting dendritic over compact morphologies not only exposes more catalytically active high-index facets and undercoordinated sites but also offers a rational pathway to enhance performance in energy conversion and electrocatalytic applications. As HEA nanocrystals continue to evolve as a versatile platform for catalysis, mastering the deposition–diffusion kinetics will remain central to structural design and functional optimization.

**Figure 19:**
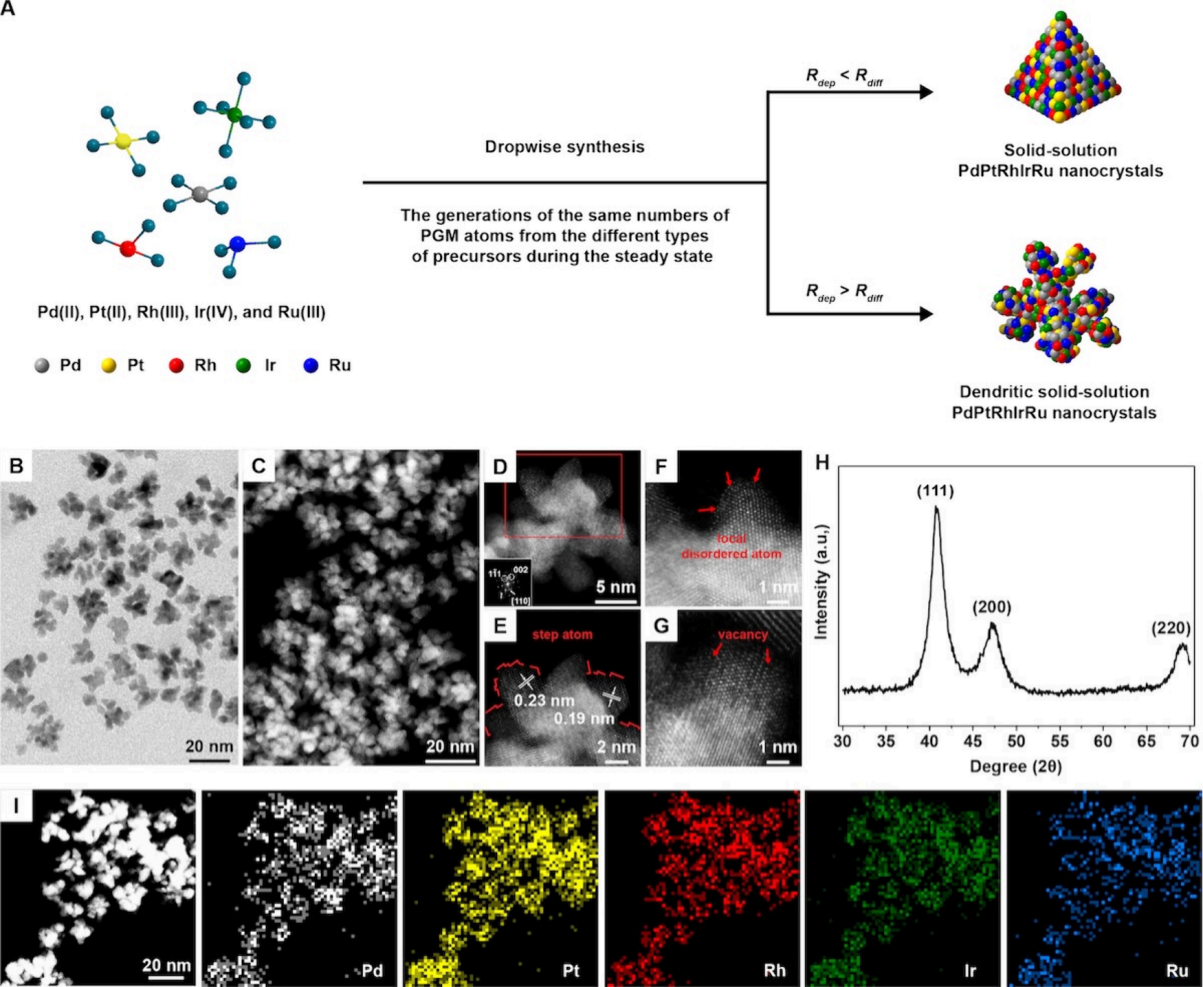
Deposition–diffusion interplay governs the morphology evolution of HEA nanocrystals. (A) Schematic of atomistic growth pathways regulated by the competition between *R*_*dep*_ and *R*_*diff*_. (B–G) TEM and HAADF-STEM images with corresponding FFT pattern of the dendritic PdPtRhIrRu HEA nanocrystals synthesized via dropwise addition at 180 °C. (H) XRD pattern of the dendritic PdPtRhIrRu HEA nanocrystals. (I) EDS elemental mapping of Pd, Pt, Rh, Ir, and Ru of the dendritic PdPtRhIrRu HEA nanocrystals. Reproduced with permission from ref 78. Copyright 2023 AAAS.

#### 3.3.4 Seed-Mediated Growth

In conventional colloidal synthesis, metal nanocrystals are typically produced through a one-pot strategy where nucleation and growth proceed concurrently in a shared reaction medium. This simultaneous progression often complicates synthetic control, as the competing kinetics of homogeneous nucleation and subsequent crystal growth are difficult to decouple. Without tight control of the instantaneous monomer concentration, the resulting nanocrystals frequently exhibit broad variations in size, shape, and internal structure. To address these challenges, seed-mediated growth has emerged as an effective approach by decoupling nucleation from growth both temporally and spatially. In this method, preformed seeds with defined dimensions and crystallographic orientation are introduced into a metal precursor solution. Rather than forming new nuclei, metal atoms generated from precursor reduction preferentially deposit onto the surface of these seeds, promoting heterogeneous nucleation. This process effectively suppresses homogeneous nucleation by lowering its thermodynamic barrier, enabling controlled crystal growth even under relatively dilute or mild conditions. This strategy operates under principles analogous to the macroscopic Czochralski method for single-crystal growth, where a seed crystal is immersed in a supersaturated melt to direct unidirectional solidification. In the colloidal regime, the internal structure of the seed remains largely preserved during growth, while its surface lattice guides atom-by-atom deposition. By tuning parameters such as precursor concentration, ligand chemistry, and reaction temperature, researchers can rationally manipulate the morphology, facet exposure, and even elemental distribution of the resulting nanocrystals with high precision.

Surface diffusion of adatoms further plays a decisive role in determining the final morphology during seed-mediated growth. When adatom mobility is insufficient, newly deposited atoms tend to aggregate locally, leading to island growth rather than conformal layer-by-layer deposition. Moderate heating can enhance surface diffusion and promote the formation of well-defined epitaxial shells. However, under overly aggressive conditions such as excessively high monomer flux or elevated temperatures that accelerate precursor reduction, deposition can become too rapid for adatoms to equilibrate across the surface, resulting in rough shells, defect-rich interfaces, or locally inhomogeneous compositions. Therefore, balancing atom supply with adatom mobility is critical for constructing uniform HEA layers with sharp facets and homogeneous elemental distributions. In addition, redox interactions between metal precursors and the seed must be carefully considered, particularly when the external reducing power is weak or when highly noble precursors are present. If a precursor has a higher reduction potential than the seed metal, galvanic displacement can occur, leading to partial dissolution of the seed and unintended deposition of more noble metals. Such processes may generate porosity, alter the seed surface termination, or disrupt compositional uniformity, and the risk is amplified in multimetallic systems where large differences in reduction potentials exist among constituent elements.

The choice of seed material is also crucial for enabling epitaxial growth of HEA shell layers. A key parameter is the lattice mismatch between the seed and the HEA overlayer, as excessive mismatch can induce interfacial strain, defect formation, and a transition from layer-by-layer growth to island growth. Typically, maintaining the mismatch below 5% favors coherent epitaxy. For instance, Pd is commonly employed as a seed for FCC-structured PdPtRhIrRu HEAs due to its crystallographic compatibility and a lattice constant (3.89 Å) closely aligned with that of the target alloy, minimizing interfacial strain. Beyond lattice considerations, precise kinetic controls over both metal atom deposition and surface diffusion are essential for achieving conformal growth. Among the available strategies, dropwise precursor addition has proven particularly effective. By maintaining a low, steady-state precursor concentration, this method suppresses homogeneous nucleation and promotes site-selective deposition on the seed surface. This helps ensure continuous layer formation while minimizing the generation of random nuclei or sub-3 nm clusters. However, overly rapid injection rates can lead to local supersaturation, triggering uncontrolled nucleation and polycrystalline deposition that compromise the epitaxial structure.

As outlined in the previous section, dropwise precursor addition enables near-concurrent reduction of multiple metal species, promoting the formation of uniform multimetallic nanocrystals while mitigating phase segregation arising from mismatched reduction kinetics. When this kinetic control is integrated with seed-mediated growth, facet-specific deposition of multimetallic atomic layers becomes feasible while preserving the crystallographic registry and surface atomic arrangements templated by the seed.^80^ In a seminal study, this concept was demonstrated by growing a quaternary PdPtRuRh alloy shell via controlled, dropwise addition of multiple precursors into a suspension of Rh cubic seeds covered by {100} facets, yielding multimetallic shells with well-maintained facet identity and high atomic-level uniformity. This combination provides a powerful framework for tailoring the surface structure and composition of multimetallic nanocrystals with atomic precision: the seed not only serves as a preferred nucleation platform but also dictates the crystallographic orientation of the deposited layers, enabling the construction of multimetallic shells with controlled facets. Furthermore, the seed-mediated approach can be extended beyond direct single-phase growth. A two-step synthetic route has also been developed in which initial core–shell nanocrystals, often comprising a well-defined core with a compositionally distinct HEA shell, are first synthesized via colloidal chemistry. These two-phase core–shell structures are then subjected to postsynthetic thermal annealing to facilitate interdiffusion and alloying across the core–shell interface. This transformation yields single-phase HEA nanocrystals.^199,200^

Recent studies have shown that quinary Pd_0.2_Pt_0.2_Ir_0.2_Ru_0.2_Rh_0.2‑nL_ HEA shells, where n denotes the number of atomic layers, can be epitaxially grown on Pd nanocubes and octahedra, resulting in well-defined {100} and {111} surface facets, respectively.^82^ This dropwise, seed-mediated approach is built on our earlier work on atomic layer-by-layer deposition of Pt onto shape-defined Pd nanocrystals, including nanocubes, octahedra, decahedra, and icosahedra, to construct epitaxial Pt overlayers with controlled surface terminations for ORR catalysts with enhanced activity and durability.^74,201–204^ Extending this concept from a single overlayer element to multiple precursors enables the epitaxial growth of compositionally complex HEA shells while retaining the facet identity and crystallographic registry dictated by the Pd seeds. Maintaining a low and steady precursor concentration (0.8 mL h^–1^) at an elevated temperature of 195 °C ensures that atomic deposition occurs preferentially on the seed surface while suppressing homogeneous nucleation. This approach promotes layer-by-layer rather than island growth, and surface diffusion is sufficiently activated to enable uniform incorporation of the reduced atoms. As a result, quinary HEA shells with conformal, atomically smooth surfaces and distinct facet terminations can be obtained on both cubic and octahedral Pd templates, as shown in Figure 20.^82^ The thickness of the HEA shell can be controlled by tuning the precursor amount. Increasing the concentration from 0.021 to 0.084 μmol mL^–1^ yields shell thicknesses of approximately 2, 4, and 6–7 atomic layers, as confirmed by HAADF-STEM imaging and atomic-resolution analysis. The nanocubes retained their well-defined morphology and exhibited a smooth surface atomically, with lattice fringes extending across the Pd–HEA interface, indicating coherent epitaxial growth. FFT patterns further confirm the single-crystal FCC structure. Elemental mapping and inductively coupled plasma-optical emission spectrometry (ICP-OES) analyses revealed uniform distributions of the five constituent metals in near-equimolar ratios, supporting the formation of a solid-solution phase. Importantly, XAS provides deeper insight into the local bonding environment. The coordination numbers extracted from extended EXAFS analysis, 4.70/5.28 for Pt, 3.24/5.54 for Ir, 3.28/4.66 for Ru, and 3.12/3.31 for Rh (M-4d/M-5d shells, respectively), indicate a high degree of atomic-level mixing among the constituent metals. These values reflect a random yet intimately mixed coordination environment characteristic of solid-solution HEA shells. Collectively, these results highlight the importance of finely tuned precursor injection kinetics, thermal conditions, and seed crystallography in directing epitaxial growth of compositionally complex HEA nanocrystals with controlled thickness, crystallinity, and surface orientation.

**Figure 20:**
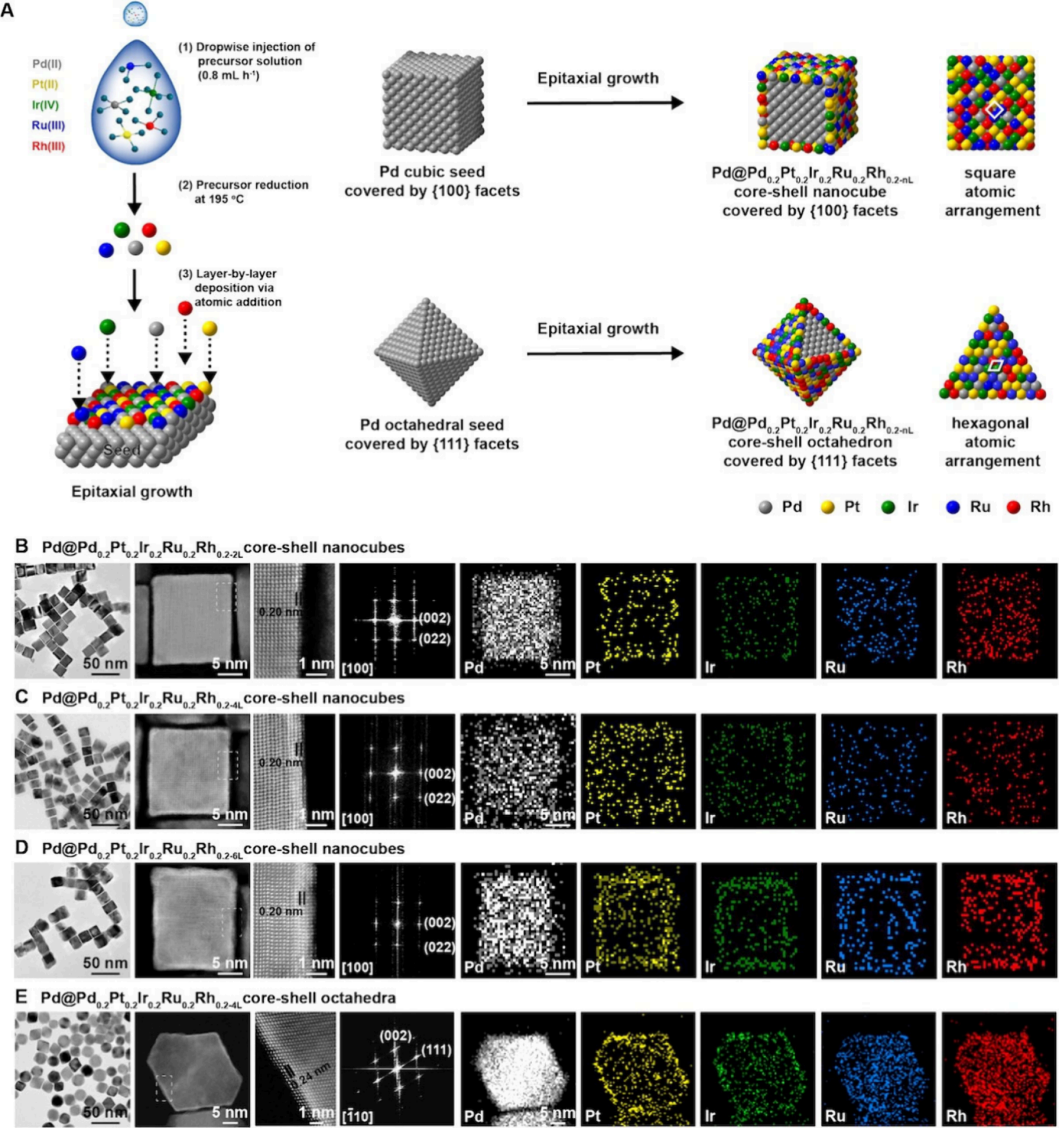
Seed-mediated growth of HEA nanocrystals. (A) Schematic illustration of the dropwise, seed-mediated epitaxial growth process for the synthesis of Pd_0.2_Pt_0.2_Ir_0.2_Ru_0.2_Rh_0.2_ atomic overlayers on Pd nanocubes and octahedra. (B–D) TEM, HAADF-STEM, FFT, EDS analysis of the Pd@Pd_0.2_Pt_0.2_Ir_0.2_Ru_0.2_Rh_0.2_ core–shell nanocubes with (B) 2, (C) 4, and (D) 6 atomic-layer shells. (E) TEM, HAADF-STEM, FFT, and EDS analyses of the Pd@Pd_0.2_Pt_0.2_Ir_0.2_Ru_0.2_Rh_0.2_ core–shell octahedra with 4 atomic-layer shells. Reproduced with permission from ref 82. Copyright 2024 Wiley-VCH.

Building on the success of Pd-seeded epitaxial growth of quinary Pd_0.2_Pt_0.2_Ir_0.2_Ru_0.2_Rh_0.2_ atomic layers, recent studies have expanded this approach to create a broad library of facet-defined HEA nanocrystals with tunable compositions.^82^ By controlling the type and concentration of metal precursors, researchers have demonstrated the feasibility of conformally depositing equimolar multimetallic atomic layers containing five to ten different elements on Pd nanocubes with well-preserved {100} facets. Representative HEA compositions include quinary alloys such as Pt_0.2_Ir_0.2_Ru_0.2_Rh_0.2_Au_0.2_ (Figure 21A) and Pt_0.2_Ir_0.2_Co_0.2_Fe_0.2_Ni_0.2_ (Figure 21B); a senary alloy Pd_0.17_Pt_0.17_Ir_0.17_Ru_0.17_Rh_0.17_Au_0.17_ (Figure 21C); a septenary alloy Pd_0.14_Pt_0.14_Ir_0.14_Ru_0.14_Rh_0.14_Os_0.14_Au_0.14_ (Figure 21D); and a denary alloy Pd_0.1_Pt_0.1_Ir_0.1_Ru_0.1_Rh_0.1_Os_0.1_Au_0.1_Co_0.1_Fe_0.1_Ni_0.1_ (Figure 21E). High-resolution TEM, HAADF-STEM imaging, and FFT analysis confirmed that these core–shell nanocrystals maintain single-crystalline symmetry and exhibit atomically sharp core–shell interfaces. Compositional mapping via EDS and ICP-OES revealed homogeneous, near-equimolar distribution of constituent elements within the HEA shells. Importantly, the stoichiometry could be fine-tuned across a series of samples, where multiple compositions were synthesized by varying precursor ratios. For systems incorporating low-reduction-potential metals such as Co, Ni, and Fe, the use of oleylamine-based thermal decomposition proved essential to ensure complete reduction and uniform deposition, as conventional polyol methods were insufficient. This epitaxial strategy was further extended to Cu@HEA nanocubes by substituting Pd with Cu seeds, illustrating its broader applicability and potential for reducing noble metal content (Figure 21F).

**Figure 21:**
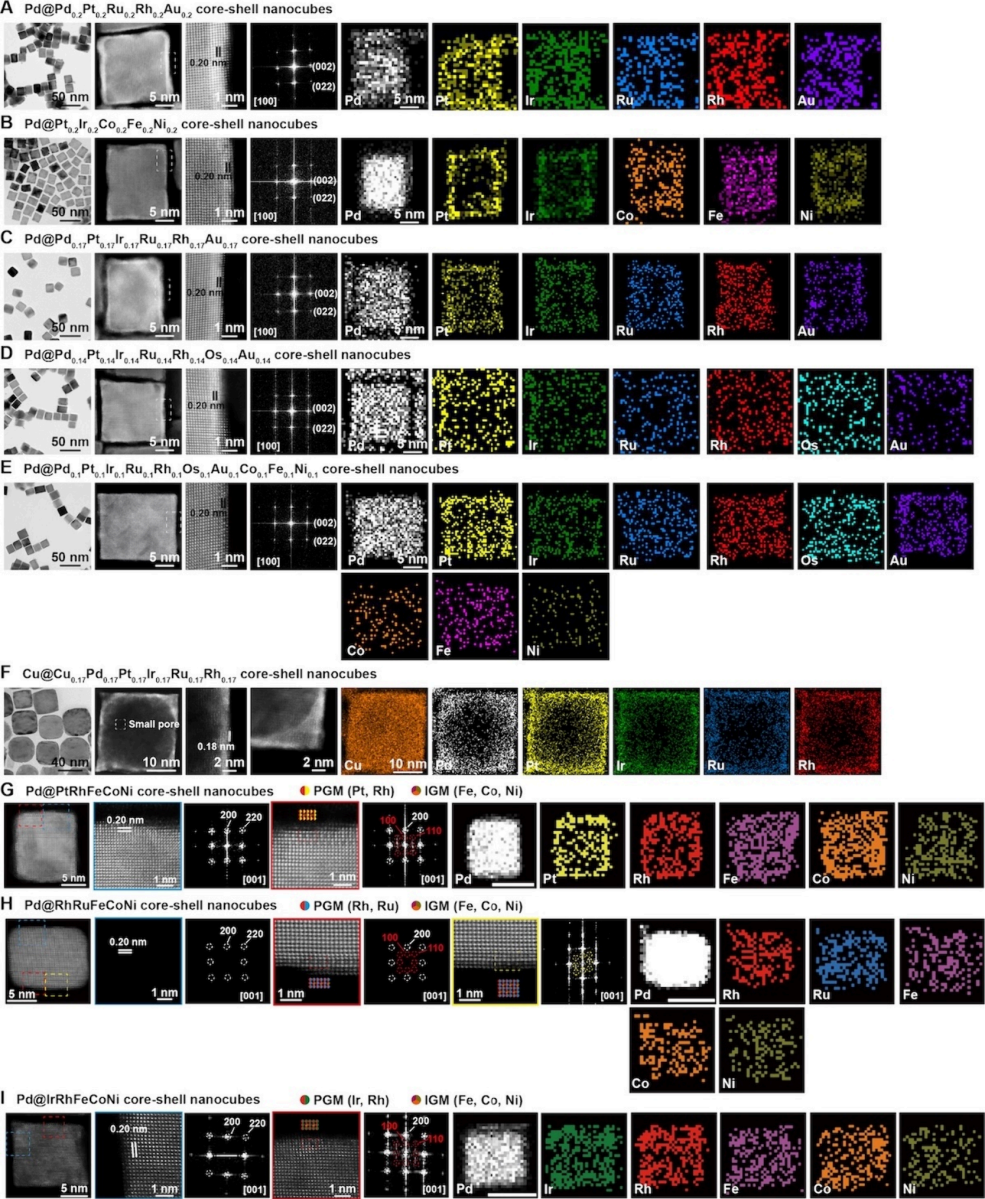
Characterizations of facet-controlled HEA nanocrystals. (A–E) TEM, HAADF-STEM, FFT, and EDS elemental mapping of the Pd@HEA nanocubes with different compositions for the shells: (A) Pt_0.2_Ir_0.2_Ru_0.2_Rh_0.2_Au_0.2_, (B) Pt_0.2_Ir_0.2_Co_0.2_Fe_0.2_Ni_0.2_, (C) Pd_0.17_Pt_0.17_Ir_0.17_Ru_0.17_Rh_0.17_Au_0.17_, (D) Pd_0.14_Pt_0.14_Ir_0.14_Ru_0.14_Rh_0.14_Os_0.14_Au_0.14_, and (E) Pd_0.1_Pt_0.1_Ir_0.1_Ru_0.1_Rh_0.1_Os_0.1_Au_0.1_Co_0.1_Fe_0.1_Ni_0.1_. (F) TEM, HAADF-STEM, and EDS elemental mapping of the Cu@HEA nanocubes. (G–I) HAADF-STEM, FFT, and EDS elemental mapping of the IGM–PGM-HEA with regions exhibiting intermetallic atomic ordering (highlighted in red and yellow frames): (G) PtRhFeCoNi, (H) RhRuFeCoNi, and (I) IrRhFeCoNi. The images in (A–F) were reproduced with permission from ref 82. Copyright 2024 Wiley-VCH. The images in (G–I) were reproduced with permission from ref 81. Copyright 2024 AAAS.

Furthermore, recent work has demonstrated that subnanometer-thick IGM–PGM-HEA layers (incorporating iron-group and platinum-group metals) can also be grown epitaxially on Pd nanocubes, yielding ten distinct compositions with well-preserved {100} facets.^81^ The resulting shells, typically 4–6 atomic layers thick, exhibited conformal coverage and compositional homogeneity, verified through HAADF-STEM contrast, EDS line profiles, and quantitative ICP measurements. Intriguingly, local atomic ordering was observed in selected compositions such as PtRhFeCoNi, RhRuFeCoNi, and IrRhFeCoNi (Figure 21G–I). In these systems, HAADF-STEM imaging revealed alternating bright and dark atomic columns, consistent with L1_2_-type ordering where PGMs preferentially occupy face-centered positions while IGMs reside at corner sites. FFT patterns showed superlattice reflections indicative of (100) and (110) periodicities. These findings suggest that local ordering within HEA atomic layers can emerge spontaneously, driven by composition-dependent site preferences, even at relatively moderate synthesis temperatures. Collectively, these results highlight the compositional flexibility and facet tunability of wet-chemical epitaxial growth, establishing it as a powerful platform for constructing HEA nanocrystal libraries. The ability to tailor both elemental makeup and surface crystallography offers immense opportunities for probing synergistic multimetallic interactions and advancing rational catalyst design.

#### 3.3.5 Post-Synthetic Thermal Conversion

In addition to direct colloidal synthesis, postsynthetic thermal conversion offers a programmable pathway to transform well-defined multimetallic core–shell nanocrystals into structurally uniform, compositionally complex nanocrystals.^199,200^ This method decouples the nucleation and alloying steps, allowing the use of compositionally tailored seeds with predesigned architectures that undergo controlled interdiffusion, crystallization, and atomic mixing upon annealing. As illustrated in Figure 22A, this nanocrystal conversion strategy begins with colloidally synthesized bimetallic or trimetallic core–shell structures, which are subsequently dispersed on carbon supports and annealed at elevated temperatures (typically 700–900 °C) under inert (e.g., N_2_) or reducing (e.g., H_2_/Ar) atmospheres for 1–3 h. Depending on the elemental combination and annealing conditions, the same synthetic route can yield distinct structural outcomes, including Janus-type heterostructures, high-entropy intermetallics (HEIs), and HEAs. This approach effectively addresses challenges related to immiscibility and reduction sequence mismatches by enabling independent control over elemental intermixing and phase evolution. For example, Kar and co-workers demonstrated that nonequimolar core–shell Au_*x*_Cu_1–*x*_@PtPdM (M = Ni or Fe) precursors predominantly yield Janus-type AuCuPd–PtM nanostructures upon annealing at 900 °C under N_2_, highlighting a thermodynamically driven phase separation behavior.^200^ In contrast, when equimolar precursor ratios are used, AuCuPtPdNi and AuCuPtPdFe nanocrystals with homogeneous elemental distributions are obtained by annealing at lower temperatures such as 700 °C, which favor alloying rather than phase segregation. Notably, Fe incorporation tends to favor the formation of ordered HEI phases, while Ni promotes random solid-solution HEA structures (Figure 22B–I). These results underscore the key role of precursor composition and annealing parameters in dictating thermodynamic pathways during nanocrystal conversion.

**Figure 22:**
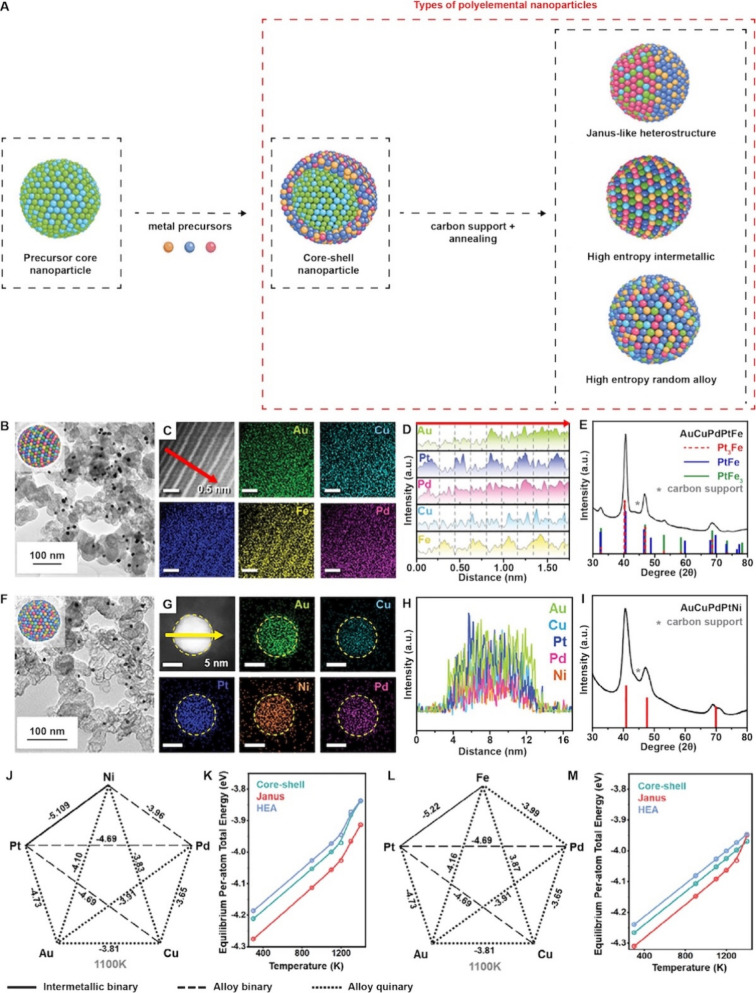
Postsynthetic thermal conversion of core–shell nanocrystals into different polyelemental nanocrystals. (A) Schematic illustration of the conversion process from colloidally synthesized multimetallic core–shell nanocrystals to various poly-elemental products, including Janus-type heterostructures, HEIs, and HEAs, via carbon-supported annealing. (B–I) TEM, HAADF-STEM, EDS elemental maps, line-scanning profiles, and XRD patterns of (B–E) AuCuPdPtFe HEI nanocrystals and (F–I) AuCuPdPtNi HEA nanocrystals. (J–M) DFT-calculated mixing enthalpy and equilibrium per-atom total energy for representative compositions. Reproduced with permission from ref 200. Copyright 2024 Wiley-VCH.

The underlying mechanism governing phase selection is rooted in the competition between enthalpic stabilization from specific atomic pairs and the entropic gain from multicomponent mixing. As shown in Figure 22J–M, DFT calculations indicate that minimizing the Gibbs free energy of mixing requires a balance between configurational entropy and favorable mixing enthalpy, a condition most effectively realized in equimolar multimetallic systems. Importantly, this study provides the first demonstration of a colloidal nanocrystal conversion platform capable of producing Janus, HEI, and HEA structures in a composition-tunable and structurally programmable manner, offering a generalizable strategy for synthesizing complex multimetallic nanocrystals with different phases.

#### 3.3.6 Galvanic Replacement

Galvanic replacement is widely employed for synthesizing metallic nanocrystals, particularly hollow or porous structures, by exploiting the redox potential differences between a sacrificial metal template and more noble metal precursors. During this process, the template metal undergoes oxidation and dissolution, while the incoming metal ions are reduced and deposited onto the evolving surface. Owing to its simplicity and rapid reaction kinetics, the transformation typically completes within minutes. Nevertheless, when extended to multicomponent systems, this approach introduces substantial complexity because of the varying stoichiometries, reduction potentials, and bonding affinities among different metal precursors. As a result, simultaneous redox and diffusion processes involving multiple species often proceed unevenly, making it difficult to achieve homogeneous atomic mixing, surface smoothness, and structural precision.

To address these challenges, researchers have developed a model system based on Ag nanocubes as sacrificial templates and Pd^2+^, Pt^2+^, and Au^3+^ precursors to investigate how precursor addition strategies influence the structural and compositional evolution of quaternary AgPdPtAu hollow nanocrystals (Figure 23A).^205^ Four distinct precursor addition modes were systematically examined to disentangle the effects of redox potential, bonding strength, and diffusion dynamics. In Sequential Addition I, where Pd^2+^ and Pt^2+^ were introduced prior to Au^3+^, the resulting nanocrystals displayed polycrystalline, phase-separated structures with island-like surface morphologies. By contrast, Sequential Addition II, which reversed the addition order by introducing Au^3+^ before Pd^2+^ and Pt^2+^, produced well-defined hollow nanocubes exhibiting single-crystalline structures enclosed by {100} facets and homogeneous solid-solution compositions. One-shot Addition III, involving the simultaneous introduction of all precursors, generated solid-solution nanocrystals but with rougher surfaces due to competitive deposition and uncontrolled alloying dynamics. Finally, Dropwise Addition IV, in which the precursors were added gradually, enabled progressive atomic interdiffusion yet led to structural collapse when the degree of replacement exceeded the framework’s mechanical stability.

**Figure 23:**
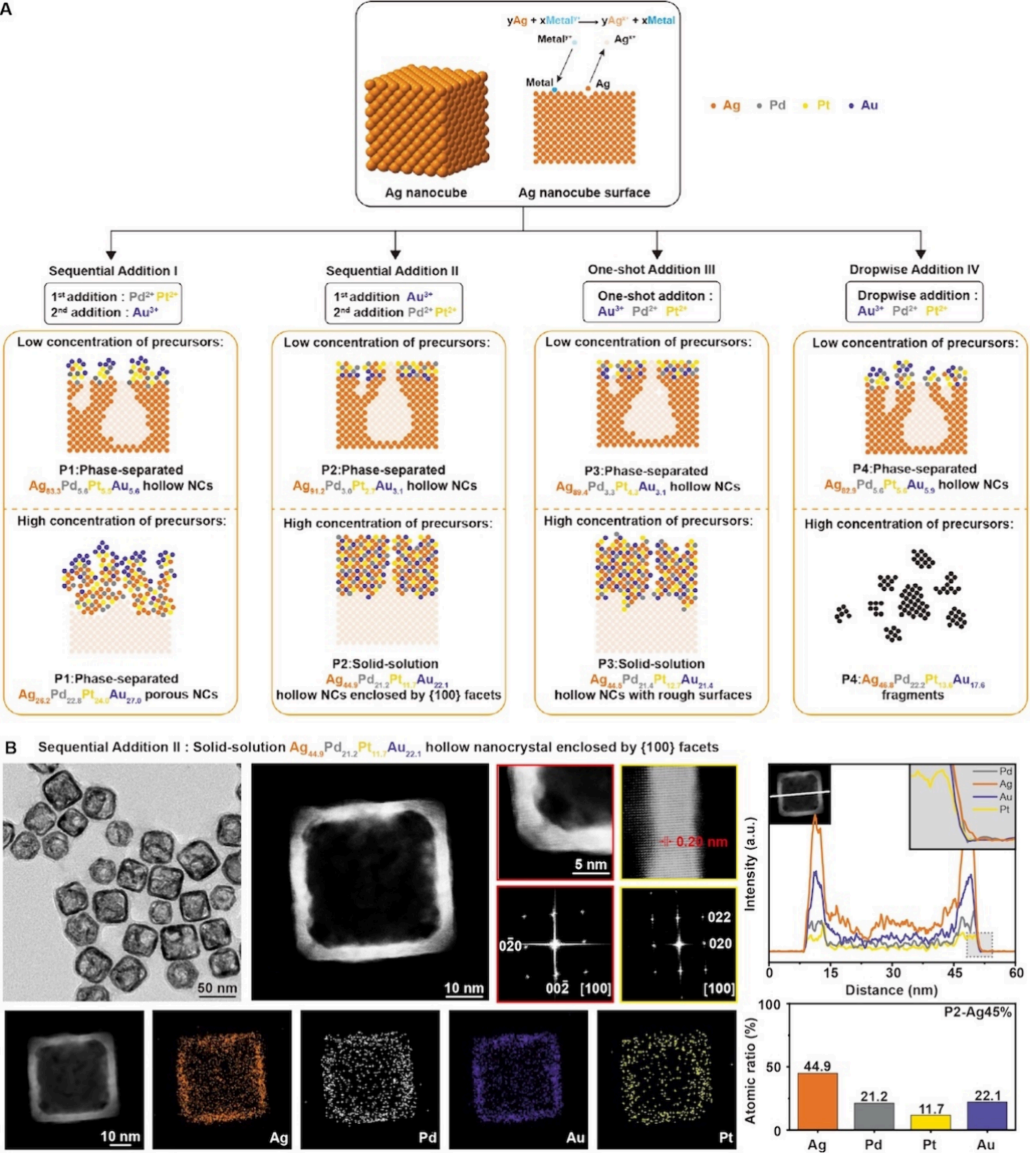
Galvanic replacement synthesis of quaternary AgPdPtAu hollow nanocrystals. (A) Schematic illustration of precursor addition strategies for Ag nanocube-templated galvanic replacement, comparing sequential, one-shot, and dropwise addition modes under different precursor concentrations. (B) TEM, HAADF-STEM, FFT, line-scanning profile, EDS elemental maps, and ICP-OES analysis of Ag_45_PdPtAu nanocrystals synthesized via sequential addition II (Au^3+^ → Pd^2+^/Pt^2+^). Reproduced with permission from ref 205. Copyright 2025 Wiley-VCH.

High-resolution HAADF-STEM imaging, FFT pattern analysis, and elemental mapping collectively demonstrated that Ag_45_PdPtAu nanocrystals synthesized via Sequential Addition II exhibited a well-defined solid-solution structure, featuring uniform elemental distribution and preserved cubic geometry (Figure 23B). Notably, the bonding strength between the deposited noble metals and the underlying Ag template emerged as a critical factor influencing surface structure. Strong intermetallic bonding interactions facilitated the formation of atomically smooth and epitaxially aligned surfaces, whereas weaker bonding affinities resulted in rough, polycrystalline domains. To further corroborate these experimental findings and elucidate the thermodynamic preference for solid-solution formation, DFT calculations were conducted to compare the surface energetics of representative phase-separated and mixed-alloy configurations. The results revealed that the average surface energy of solid-solution slabs (Es = 0.03 eV Å^–2^) was consistently lower than that of the phase-separated counterpart (Es = 0.05 eV Å^–2^), affirming the greater thermodynamic stability of homogeneous mixing under high precursor concentration and {100}-facet exposure.

Recent advances have established a rational synthetic route for constructing ultrathin HEA subnanoribbons with tunable multimetallic compositions and atomic-level uniformity.^125^ As illustrated in Figure 24A, the synthesis follows a three-step design: *i*) galvanic exchange initiates the partial replacement of surface Ag atoms on single-crystalline Ag nanowires with noble metal ions (e.g., Pt^4+^, Pd^2+^, Ir^3+^, Ru^3+^), facilitating the nucleation of multimetallic atoms; *ii*) co-reduction of remaining metal precursors occurs under a controlled reductive environment, enabling the epitaxial deposition of additional metal atoms while preserving the Ag template; *iii*) a selective dealloying process removes the inner Ag core, ultimately yielding hollow HEA subnanoribbons with well-preserved geometry and homogeneous elemental distribution. HAADF-STEM images (Figure 24B–G) demonstrate the formation of quinary (PtPdIrRuAg), senary (PtPdIrRuAuAg), septenary (PtPdIrRuAuRhAg), and octonary (PtPdIrRuAuRhOsAg) HEA subnanoribbons with ribbon-like morphology and uniform thickness. Elemental mapping further confirms the homogeneous dispersion of all constituent elements, highlighting the capability to fine-tune alloy complexity through precursor design. Notably, the lattice fringes observed in high-resolution TEM reveal well-resolved (200) planes with *d*-spacings ranging from 1.85 Å to 1.86 Å, evidencing solid-solution phase formation. The corresponding XRD patterns (Figure 24H) show consistent peak positions and broadened features, confirming the nanocrystalline nature of all HEA subnanoribbons and the preservation of an FCC phase despite compositional complexity. This modular and scalable approach offers exceptional control over HEA composition, enabling the creation of a versatile library of subnanoribbon catalysts. These findings underscore the versatility of galvanic replacement-based strategies for engineering multimetallic nanostructures, where careful control over precursor chemistry, addition sequence, and reduction dynamics enables precise modulation of composition, phase, and morphology. Together, these approaches establish a programmable platform for constructing atomically mixed high-entropy nanomaterials with tunable catalytic and structural properties.

**Figure 24:**
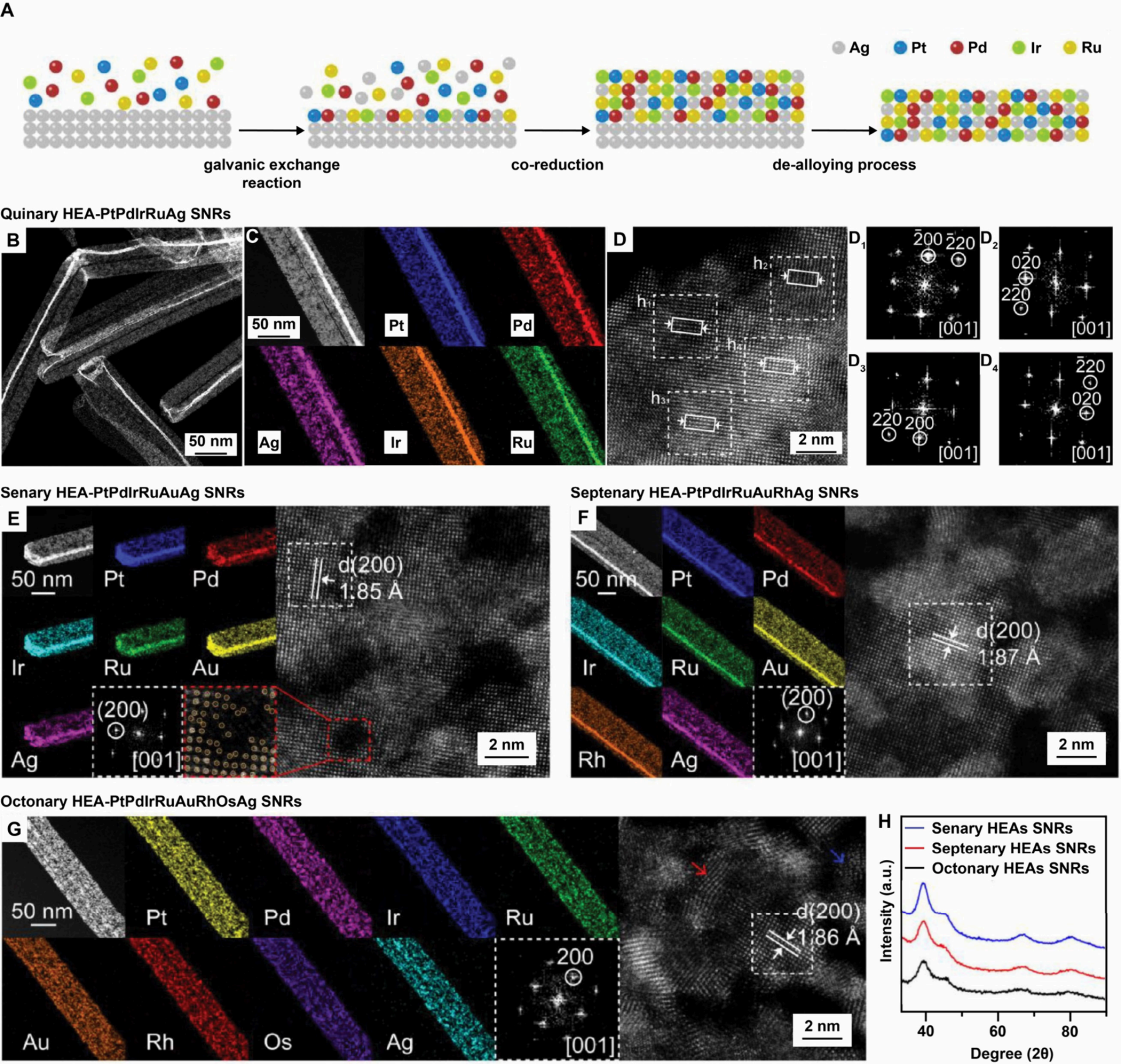
Synthesis and structural characterization of ultrathin HEA subnanoribbons. (A) Schematic illustration of the three-step synthetic strategy involving galvanic exchange, coreduction, and selective dealloying to construct multimetallic HEA subnanoribbons. (B–D) TEM, HAADF-STEM, FFT patterns, and EDS elemental maps of representative quinary PtPdIrRuAg HEA subnanoribbons. (E–G) HAADF-STEM, FFT patterns, and EDS elemental maps of multicomponent HEA subnanoribbons: (E) senary PtPdIrRuAuAg, (F) septenary PtPdIrRuAuRhAg, and (G) octonary PtPdIrRuAuRhOsAg. (H) XRD patterns of senary, septenary, and octonary HEA subnanoribbons. Reproduced with permission from ref 125. Copyright 2022 American Chemical Society.

#### 3.3.7 Continuous-Flow Synthesis

Developing scalable and reproducible synthetic routes is critical for bridging the gap between laboratory studies and the industrial deployment of HEA nanocrystals. Although many wet-chemical approaches offer excellent controls over composition and morphology, most of them are limited to milligram- to subgram-scale production. Such limited throughputs pose a major challenge for industrial application, especially given the intrinsic complexity of HEA synthesis, which requires the coordinated reduction of multiple metal precursors with different standard reduction potentials, atomic radii, and diffusion behaviors while evolving into an alloy. When reaction volume is increased for scale-up, local gradients in temperature, concentration, and mixing efficiency often emerge within batch reactors. These heterogeneities lead to undesirable effects such as phase segregation, uneven atomic mixing, and morphological irregularities, thereby compromising the catalytic performance and reproducibility of the resulting nanocrystals.^206^ To address these challenges, continuous-flow synthesis has emerged as a promising alternative that allows precise control over reaction kinetics and environmental uniformity. Unlike batch systems that rely on bulk mixing and prolonged heating, continuous-flow reactors maintain a steady-state regime wherein reactants are introduced, mixed, and converted under consistent conditions. Parameters such as flow rate, precursor concentration, and reaction temperature can be tightly regulated along the flow path, minimizing local fluctuations and enabling reproducible control over nucleation and growth dynamics. This is particularly advantageous for multicomponent alloy systems, where concurrent reduction of disparate metal species is crucial to achieving atomic-scale homogeneity.

Recent work by Kitagawa and co-workers exemplifies the power of continuous-flow platforms for producing compositionally complex nanocrystals at scale. In a 2022 study, the authors designed a custom continuous-flow reactor to synthesize ultrasmall equimolar IrPdPtRhRu HEA nanocrystals using a strong reducing agent under carefully optimized flow and temperature conditions (Figure 25A, B).^21^ High-resolution STEM and elemental mapping confirmed that the resulting particles, with an average diameter of 1.32 nm, exhibited uniform alloying and a single-phase structure without observable segregation (Figure 25C–H). The continuous-flow configuration effectively eliminated thermal and compositional gradients typically found in larger-scale batch syntheses, enabling gram-scale production while maintaining nanocrystal uniformity. Building upon this approach, the same group subsequently developed a benchtop four-channel flow reactor capable of synthesizing ultramultielement alloy nanocrystals containing up to 15 different elements, spanning both d-block and p-block metals.^207^ The system accommodated sequential injection of individual precursors and operated at relatively low temperatures (ca. 66 °C), successfully yielding uniform 1.9 nm BiCoCuFeGaInIrNiPdPtRhRuSbSnTi nanocrystals. Despite the pronounced differences in precursor solubility and reduction kinetics among these elements, the carefully orchestrated precursor feeding and reaction management ensured homogeneous nucleation and alloy formation. This result highlights the platform’s robustness in handling highly complex precursor systems and producing compositionally diverse nanocrystals under mild, scalable conditions. These advances collectively underscore the advantages of continuous-flow synthesis in producing HEA nanocrystals with high structural integrity, compositional precision, and reproducibility. By eliminating key bottlenecks of batch processes, namely nonuniform reduction environments and limited scalability, this method opens new avenues for the high-throughput preparation of multimetallic nanocatalysts. Furthermore, the modular nature of flow systems facilitates integration with automation and real-time monitoring, making them ideal platforms for systematic exploration of vast compositional spaces and the accelerated advanced functional materials.

**Figure 25:**
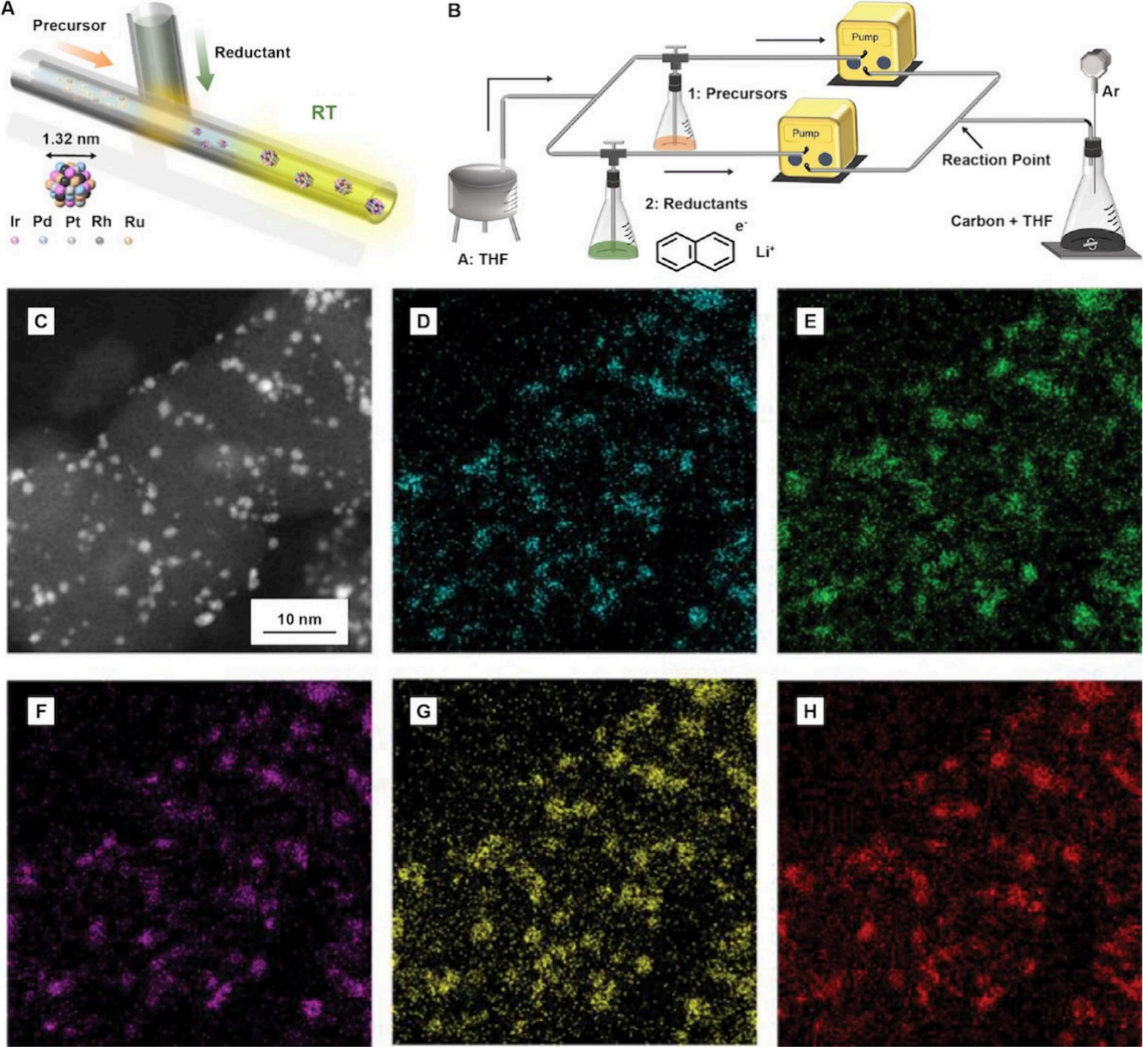
Continuous-flow synthesis of ultrasmall IrPdPtRhRu HEA nanocrystals. (A) Schematic illustration of the continuous-flow reactor design for synthesizing equimolar IrPdPtRhRu HEA nanocrystals under controlled flow and temperature conditions. (B) Experimental setup showing independent precursor and reductant pumps converging at the reaction point in a continuous-flow system. (C–H) HAADF-STEM image and corresponding EDS elemental maps of ultrasmall IrPdPtRhRu HEA nanocrystals. Reproduced with permission from ref 21. Copyright 2022 American Chemical Society.

## 4 Characterizations

Ideally, HEA nanocrystals exhibit a single-phase solid-solution structure, in which constituent elements are uniformly and randomly distributed at the atomic scale. However, experimentally verifying such random atomic configurations and disentangling the synergistic effects among multiple elements remains challenging. Reliable assessment of structural integrity, compositional homogeneity, and active-site distribution, as well as mechanistic elucidation and catalytic optimization, critically depends on advancement in characterization technique. Recent advances in high-resolution diffraction, microscopy, and spectroscopy have greatly expanded the capability to analyze HEA nanocrystals in atomistic details. In the following subsections, we discuss the major techniques used for characterizing HEA nanocrystals from three key perspectives: *i*) Morphology, facets, and elemental distribution, *ii*) crystallographic properties and chemical short-range order structures, and *iii*) surface chemical environment and electronic structure. Each aspect is discussed in terms of its importance, representative characterization techniques, and examples from recent literature.

### 4.1 Morphology, Facets, and Elemental Distribution

#### 4.1.1 Importance

As discussed in the previous sections, the morphology, including size, shape, and facets, of HEA nanocrystals and how the multiple elements are distributed on the surface are fundamental to their performance. A defining characteristic of HEAs is the uniform, atomic-level mixing of all the constituent elements within a single-phase solid-solution structure. Confirming this random distribution is essential, as any deviation, such as elemental segregation, compositional gradient, or core–shell configuration, undermines the high-entropy design principle and can compromise catalytic activity, selectivity, and long-term stability. Additionally, controlled morphologies from zero-dimensional (0D) to 3D (e.g., particles, wires, sheets, and frameworks) are now attainable for HEAs, and each dimensional form may present different facet exposures or element distribution challenges. The ultimate goal of such characterizations is to confirm that the nanoscale HEMs have the intended size/shape and that all elements are homogeneously distributed, or to detect any heterogeneity, such as elemental clustering, surface segregation, or local compositional ordering.

Key characterization techniques for morphology, facets, and elemental distribution include:

(i) Size, shape, facet, and morphology: scanning electron microscopy (SEM), TEM, and STEM are the primary tools for examining nanocrystal shape, size, and internal structure. HAADF-STEM provides atomic-scale imaging with Z-contrast, enabling direct observation of atomic arrangements in HEAs. FFT analysis of lattice images assists in identifying the underlying crystal structure. SEM is suitable for larger particles or porous frameworks, giving low-resolution but wide-area surveys of morphology and size distribution, whereas TEM is essential for resolving sub-10 nm features.
(ii) Elemental distribution: electron microscopy combined with EDS provides spatially resolved elemental mapping and compositional profiles. HAADF-STEM–EDS can be used to visualize the distribution of each element, revealing whether atomic mixing is uniform or if segregation occurs. Line-scan EDS profiles quantify elemental ratios through peak intensities along a defined path. EELS complements EDS by providing oxidation-state information and enhanced sensitivity to light elements such as C, N, and O.^25^
(iii) Elemental content: ICP-OES and ICP mass spectrometry (ICP-MS) can be used to determine overall elemental ratios and confirm bulk composition. ICP-OES detects the light emitted by excited atoms, which reveals the element’s identity and concentration. In contrast, ICP-MS detects ions based on their mass, allowing it to find elements at much lower concentrations (trace levels). As a result, ICP-MS can detect extremely small concentrations (down to parts per trillion or ppt), whereas ICP-OES is typically effective down to parts per billion (ppb).^208^ All together, these techniques provide accurate quantification of all alloying elements and validate the equiatomic or designed compositions of HEA nanocrystals.
(iv) Specific surface area and porosity: BET and Barrett–Joyner–Halenda (BJH) are typically applied to measure specific surface area, pore volume, and pore-size distribution of HEA frameworks, which are directly related to catalytic accessibility and diffusion behavior.
(v) Atomic tomography: atom probe tomography (APT) provides three-dimensional, atomic-scale mapping of elemental distributions, enabling direct visualization of how individual atoms are arranged within HEA nanocrystals. Through field evaporation under ultrahigh vacuum, APT allows for reconstruction of both atomic positions and chemical identities with near-ångström spatial and high mass resolution, revealing nanoscale variations in composition, segregation, and oxidation depth. APT enables researchers to derive depth-resolved compositional gradients, surface oxidation behavior, and interfacial diffusion pathways that are not accessible by XPS or EDS.^209,210^

#### 4.1.2 Examples and Findings

Early studies of HEA nanocrystals often assumed ideal atomic mixing, but detailed analysis has revealed nuance. Through combined imaging and compositional analysis, researchers have revealed both uniform and heterogeneous element distributions across diverse HEA dimensions, from 0D nanocrystals to complex 3D frameworks:

(i) Uniform vs heterogeneous mixing: Dey and co-workers examined Ni_0.19_Pd_0.21_Pt_0.22_Rh_0.20_Ir_0.18_ HEA nanocrystals prepared using solution-based hot-injection approach and found that, many particles exhibited a uniform distribution of all five elements throughout the particle, but some of them in the same sample displayed compositional variations.^96,211^ In particular, certain regions showed Pd enrichment, and a small fraction of the particles exhibited core–shell-like or patchy contrast in HAADF-STEM and EDS elemental maps, suggesting partial segregation and intraparticle heterogeneity, as shown in Figure 26.
(ii) 0D–3D nanostructures: recent advances in synthesis have enabled nanoscale HEMs to be prepared in various structural forms, including 0D particles, 1D wires or ribbons, 2D sheets or overlayers, and 3D porous frameworks. For 0D systems, as shown in Figure 27A, Wu and co-workers synthesized spherical HEA nanocrystals composed of six PGMs (Pt, Pd, Ir, Rh, Ru, and Os).^120^ HAADF-STEM and EDS analyses confirmed that the elements were uniformly distributed throughout individual particles, forming a single-phase solid-solution alloy with a narrow size distribution of 3.1 ± 0.6 nm. ICP-OES measurements showed near-equiatomic composition (16–17 at. % per element), consistent with the designed stoichiometry. For 1D structures, as shown in Figure 27B, Tao and co-workers developed ultrathin subnanoribbons composed of Pt, Pd, Ir, Ru, and Ag. TEM imaging revealed ribbons with widths of 50–150 nm and lengths of several micrometers, while HAADF-STEM and EDS confirmed a continuous alloy structure along their length.^125^ The ribbons exhibited a thickness of approximately 0.8 nm, along with lattice distortion and abundant defects. For 2D structures, as shown in Figure 27C, Qu and co-workers reported a transition-metal dichalcogenide HEA, (MoWReMnCr)­S_2_, synthesized as layered nanosheets.^212^ HAADF-STEM imaging identified a hexagonal 2H-MoS_2_-type lattice with an interlayer spacing of 0.6–0.7 nm, and EDS mapping demonstrated homogeneous distribution of all five transition metals. ICP analysis confirmed near-equiatomic composition (20 at% each metal). For 3D frameworks, as shown in Figure 27D, Li and co-workers fabricated PdCuAuAgBiIn HEA aerogels using a freeze–thaw method.^48^ SEM imaging showed a sponge-like, interconnected network of alloy filaments with an open mesoporous structure. HAADF-STEM–EDS mapping revealed uniform elemental distribution, including less common elements such as Bi and In, throughout the interior and surface regions. BET analysis gave a specific surface area of 137.9 m^2^ g^–1^ and a pore volume of 0.68 cm^3^ g^–1^. Across these examples, the combination of high-resolution imaging and elemental analysis consistently demonstrates that multielement HEAs can achieve near-random atomic mixing across diverse dimensionalities, confirming that their compositional uniformity extends from the 0D single-crystal particles to complex 3D architectures.
(iii) Shape and facet characterizations: high-resolution TEM (HRTEM) combined with EDS is commonly used to identify crystal facets and evaluate shape-dependent features of HEA nanocrystals. Very small HEA nanocrystals (*ca*. 5 nm in diameter) often adopt near-spherical geometries, often with an FCC lattice, owing to their random atomic arrangement and minimization in surface energy. In contrast, larger HEA particles can develop well-defined polyhedral morphologies, exposing specific crystallographic facets. Hsiao and co-workers reported a series of faceted Pd@HEA core–shell nanocrystals, including cubic, octahedral, and concave forms, with {100}, {111}, and high-index {311} and {331} facets, respectively, exposed on the surface.^82^ HRTEM imaging, FFT diffraction patterns, and selected-area EDS analyses confirmed their single crystallinity and well-defined facet orientations. The HEA shells exhibited random atomic distributions across all facets, with no evidence of elemental segregation. A typical example is the Pd@Pd_0.5_Pt_0.5_@Pt_0.2_Ir_0.2_Ru_0.2_Rh_0.2_Au_0.2_ core–shell concave nanocubes. TEM and HAADF-STEM imaging, supported by FFT analysis, confirmed the formation of a uniform HEA shell. Atomic-resolution HAADF-STEM images obtained from the [310] projection revealed the coexistence of high-index {311} and {331} facets on the surface. Corresponding EDS elemental maps verified that these HEA layers consisted of all five metals homogeneously mixed within a single-phase solid-solution structure (Figure 28).

**Figure 26:**
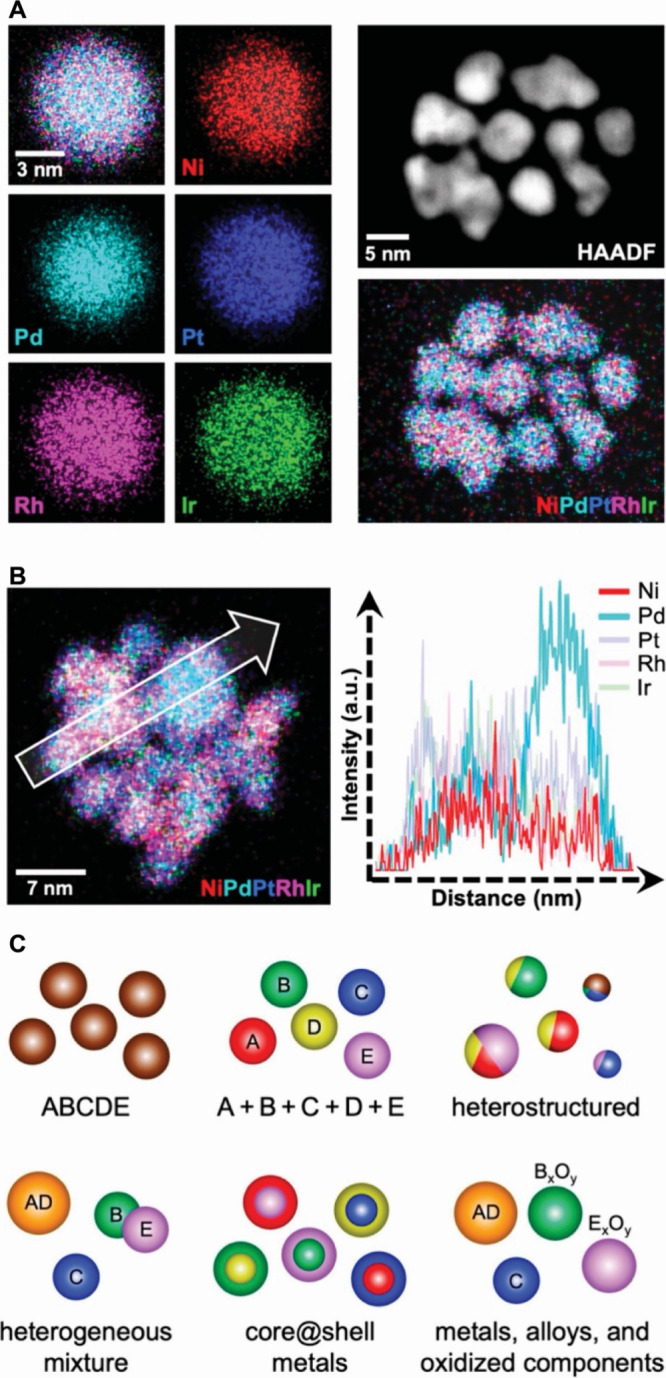
Uniform vs heterogeneous elemental mixing in NiPdPtRhIr nanocrystals prepared using solution-based hot-injection approach. (A) Overlaid STEM–EDS elemental map (Ni Kα, red; Pd Lα, cyan; Pt Lα, blue; Rh Lα, pink; Ir Lα, green) of Ni_0.19_Pd_0.21_Pt_0.22_Rh_0.20_Ir_0.18_ HEA nanocrystals, demonstrating a uniform distribution of all constituent elements. (B) Overlaid STEM–EDS elemental map from another region of the same sample, along with the corresponding EDS map and line scan (indicated by the arrow), revealing localized Pd enrichment. The line scan spans a total distance of 18 nm along the *x*-axis. (C) Schematic illustration showing possible atomic-scale configurations of five constituent metals that can form during HEA synthesis. Although all six types of samples shown in (C) may exhibit identical overall compositions when analyzed by techniques for bulk samples such as ICP-MS or ICP-OES, these methods cannot resolve nanoscale variations in elemental distribution within individual particles. The images in (A, B) were reproduced with permission from ref 96. Copyright 2023 American Chemical Society. The image in (C) was reproduced with permission from ref 211. Copyright 2024 American Chemical Society.

**Figure 27:**
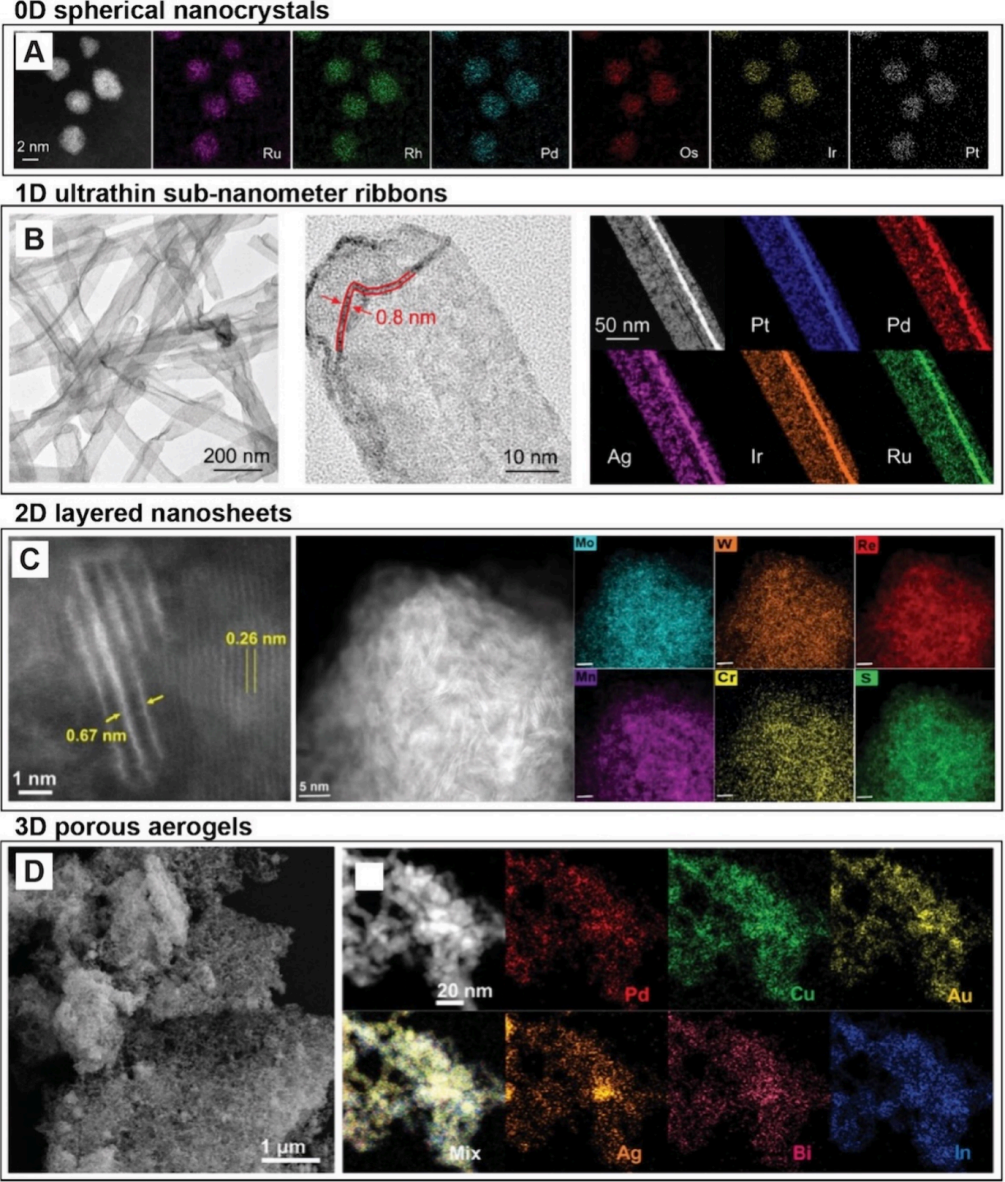
Characterizations of 0D–3D HEA nanostructures. (A) 0D spherical PtPdIrRhRuOs HEA particles. (B) 1D ultrathin PtPdIrRuAg subnanometer ribbons. (C) 2D (MoWReMnCr)­S_2_ nanosheets. (D) 3D PdCuAuAgBiIn porous aerogels. The image in (A) was reproduced with permission from ref 120. Copyright 2020 American Chemical Society. The image in (B) was reproduced with permission from ref 125. Copyright 2022 American Chemical Society. The image in (C) was reproduced with permission from ref 212. Copyright 2023 Wiley-VCH. The image in (D) was reproduced with permission from ref 48. Copyright 2022 Wiley-VCH.

**Figure 28:**
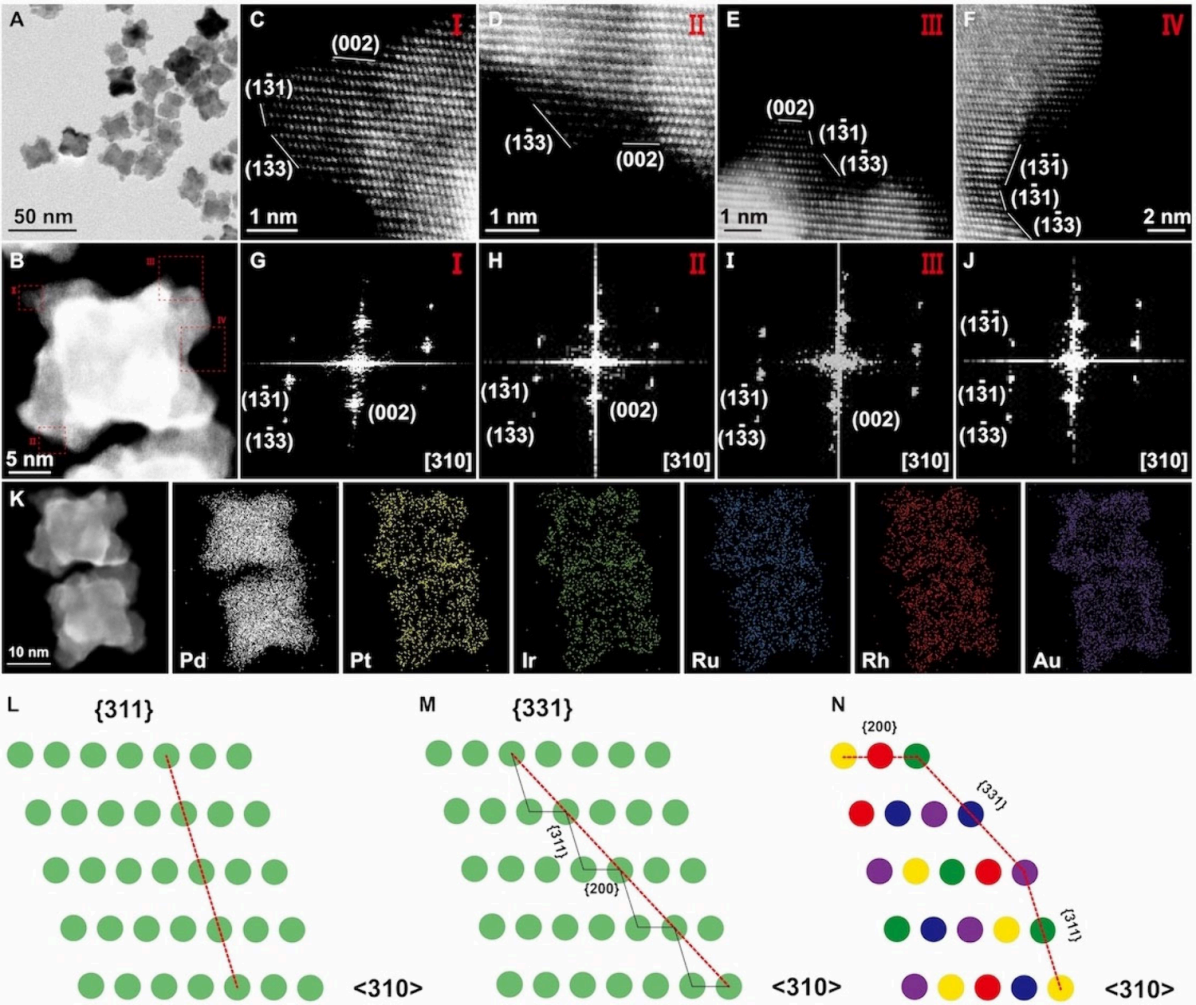
Characterizations of Pd@Pd_0.5_Pt_0.5_@Pt_0.2_Ir_0.2_Ru_0.2_Rh_0.2_Au_0.2_ core–shell nanocubes with a concave structure. (A) TEM image, (B–F) HAADF-STEM images, (G–J) corresponding FFT patterns for (C–F), (K) EDS elemental mapping images, and (L–N) models of the atomic arrangements on the (L) {311}, (M) {331} facets and (N) the multifacet surface of the HEAs. Reproduced with permission from ref 82. Copyright 2024 Wiley-VCH.

### 4.2 Crystallographic Properties and Chemical Short-Range Order Structures

#### 4.2.1 Importance

Beyond morphology, identifying the crystal phase and structural order of HEA nanocrystals is essential for understanding their properties such as unique stability. HEAs are typically designed to form single-phase solid solutions, despite their multielement composition solutions (e.g., typically a single FCC or BCC phase, with a minority of them exhibiting dual phases).^213^ A confirmed single-phase structure signifies successful alloying, whereas the emergence of secondary or ordered phases may indicate elemental segregation or incomplete mixing. However, even when a single-phase structure is achieved, local deviations from ideal atomic randomness, known as chemical short-range order (CSRO), can exist. CSRO refers to subtle atomic-scale preferences among constituent elements that lead to local lattice distortions, strain, or defect formation. These effects can strongly influence physical or catalytic behavior, but are difficult to detect and quantify.^214,215^ Because CSRO does not generate distinct diffraction peaks or new phases, experimental evidence for its presence in HEAs remains limited. Thus, the goals of this part are 2-fold: *i*) identify the crystal structure and phase purity of the HEA (average bulk/long-range structure), and *ii*) investigate any local ordering or distortions in atomic arrangement (short-range structure).

Key characterization techniques for crystallographic properties and chemical short-range order structures include:

(i) XRD: it is a fundamental technique for identifying crystalline phases and assessing phase purity. Diffraction data can be used to extract structural parameters such as lattice constants, crystallinity, grain size, preferred orientation, and microstrain. For HEA nanocrystals, a single set of diffraction peaks indicates a single-phase solid solution, whereas multiple sets of peaks suggest phase separation or intermetallic formation. Shifts in peak positions relative to pure-element standards reflect changes in the average lattice parameter caused by atomic substitution. Likewise, increased peak width and intensity reduction can signal lattice distortion caused by the random incorporation of atoms with different radii (Figure 29A).^29,216^ Taken together, XRD not only identifies the overall phase but also provides indirect information on lattice strain and crystallite size through analysis of peak shape. However, conventional in-house XRD often suffers from limited resolution and high signal-to-noise ratios, making weak secondary peaks difficult to detect and thus reducing refinement reliability. Synchrotron-based XRD can overcome these limitations by providing higher sensitivity and precision for analyzing nanoscale HEA structures.
(ii) Neutron diffraction: similar to X-ray diffraction, neutron diffraction can be employed to identify crystalline phases but offers distinct advantages in multicomponent systems. It is particularly effective for differentiating elements with similar atomic numbers or low X-ray scattering contrast, such as light elements and neighboring transition metals. In HEAs composed of elements with close atomic numbers, neutron scattering provides complementary contrast to XRD. Combining neutron and X-ray diffraction data enhances the accuracy of structural refinement, as the two techniques possess different elemental sensitivities, enabling a more reliable determination of atomic arrangements within complex alloys.^217^
(iii) Pair distribution function (PDF) analysis: this technique extends diffraction measurements to high scattering angles (using X-rays or neutrons) and Fourier-transforms the data into real space, providing quantitative information on interatomic distances and local atomic arrangements. PDF is sensitive to both short- and medium-range order, making it effective for probing structural correlations in disordered, nanocrystalline, or even amorphous materials.^218^ This capability is particularly valuable for HEAs, where local structural heterogeneity often coexists with overall crystallinity. However, interpreting PDF data for HEAs remains challenging, as the presence of numerous element pairs with similar atomic sizes and scattering factors can lead to low contrast, making it difficult to resolve subtle CSRO or local structural distortions that may be obscured in the data.^219,220,221,222^
(iv) Advanced TEM imaging and electron diffraction for probing CSRO: These techniques, particularly, HAADF-STEM, enable atomic-scale visualization of concentrated alloys and, with analytical methods, identify if there is a pattern to which elements occupy which sites. Energy-filtered TEM, which enhances contrast by selecting specific scattering vectors, has been employed to image ordered domains and distinguish locally coordinated regions within an otherwise disordered lattice.^219^ Four-dimensional STEM (4D-STEM) further expands this capability by collecting a diffraction pattern at every probe position as the electron beam is raster across the sample, generating a combined real-space and reciprocal-space data set. This technique allows the extraction of local structural information such as strain, lattice distortions, and symmetry variations across individual nanocrystals.^220,221^ Diffraction patterns obtained from single nanocrystals or selected regions can confirm whether a particle is single crystalline and reveal variations in crystallinity or structure across different particles or domains. Overall, HR-TEM imaging and electron diffraction offer powerful local probes that complement bulk diffraction methods such as XRD. They allow direct visualization of ordered clusters, lattice periodicities, and nanoscale heterogeneities associated with CSRO.
(v) AET and 3D imaging for CSRO: AET, along with focused ion beam (FIB), enables reconstruction of internal structures and 3D elemental distributions with subångström precision, providing 3D morphology visualization within nanocrystals while allowing for direct visualization of both crystal lattice arrangements and chemical identities at the single-atom level. In the study of HEAs, AET has emerged as a powerful technique for probing CSRO. It reveals local atomic correlations, clustering tendencies, and lattice distortions that are otherwise hidden in 2D projections. For instance, Chen and co-workers employed AET to determine the 3D atomic configuration of a medium-entropy alloy (VCoNi), resolving individual atomic positions and chemical species.^222^ The analysis revealed nanoscale CSRO, where V atoms preferentially avoid V–V bonds and favor V–Co/Ni pairs, forming local nonrandom arrangements confined within 1 nm regions. Such findings demonstrate that even nominally random solid-solution alloys can exhibit intrinsic atomic-level ordering, which plays a decisive role in defining their thermodynamic stability, defect behavior, and mechanical or catalytic properties.

**Figure 29:**
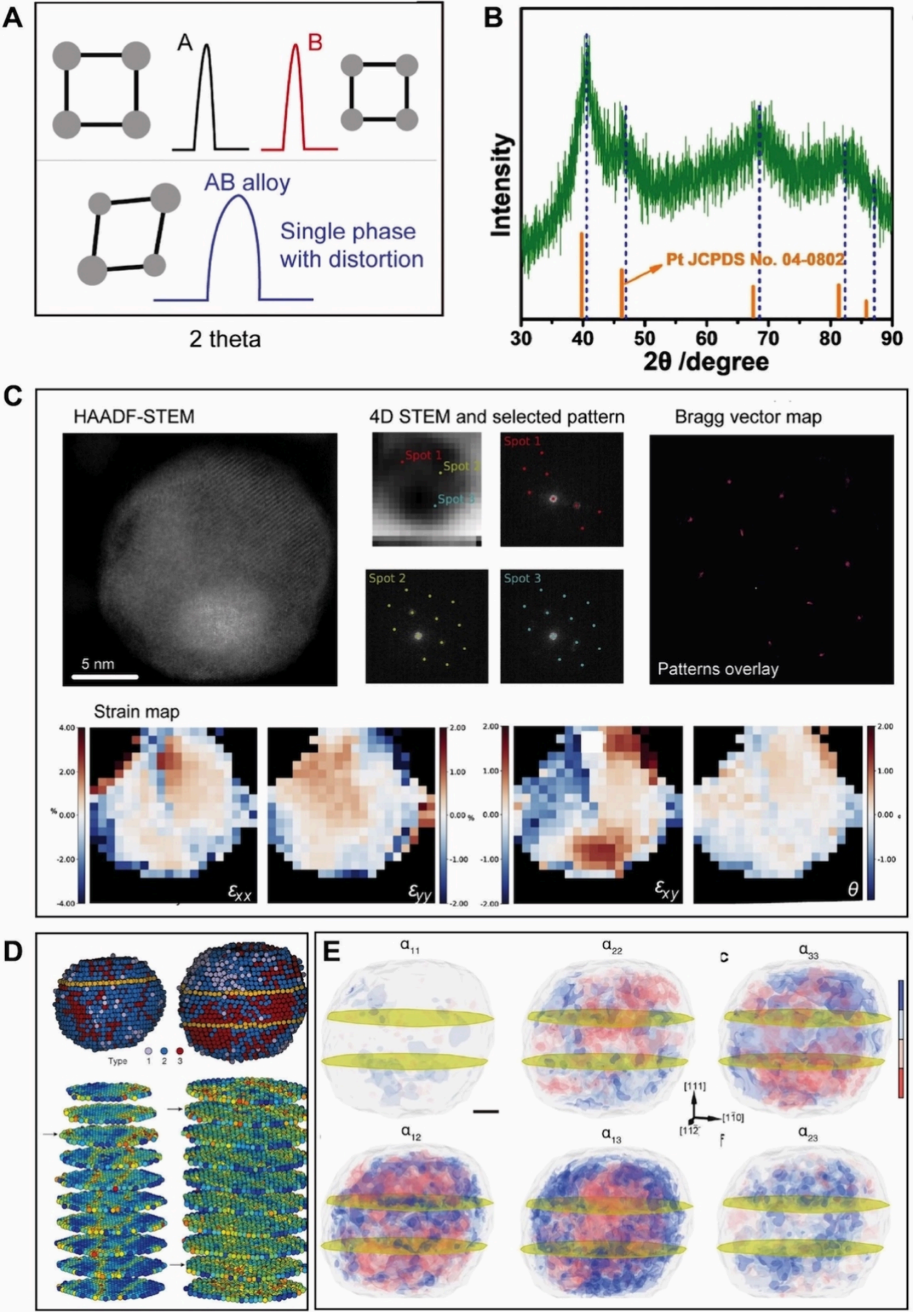
Characterization of crystallographic properties and chemical short-range order in HEA nanocrystals. (A) Schematic illustration showing potential diffraction signal shifts of HEA alloys relative to their single-metallic references in high-resolution XRD. (B) XRD pattern of ultrathin PtRuNiCoFeMo HEA nanowires. (C) 4D-STEM image of 15-element HEA nanocrystals. (D, E) 3D atomic structures and CSRO features revealed by AET. (D) Experimentally reconstructed atomic models of two nanocrystalline HEAs (nHEA-1 and nHEA-2). (E) 3D spatial distribution of six CSRO parameters (α_ij_) in the double-twinned nHEA-2, where yellow circles denote twin boundaries. The image in (A) was reproduced with permission from ref 29. Copyright 2022 AAAS. The image in (B) was reproduced with permission from ref 20. Copyright 2021 Springer Nature. The image in (C) was reproduced with permission from ref 192. Copyright 2021 Elsevier. The image in (D) was reproduced with permission from ref 223. Copyright 2023 Springer Nature.

#### 4.2.2 Examples and Findings

The following examples demonstrate how advanced techniques uncover local lattice distortion, strain variation, and CSRO in HEAs, features that are often hidden from conventional analysis but critically influence material stability and performance:

(i) Lattice distortion evidenced by XRD: in a study by Zhan and co-workers, ultrathin PtRuNiCoFeMo HEA nanowires were found to exhibit clear diffraction signatures of lattice distortion.^20^ The XRD pattern showed FCC peaks that were positively shifted relative to pure Pt, indicating lattice contraction caused by the incorporation of smaller atoms such as Ni and Co. In addition, the diffraction peaks were significantly broadened, beyond what would be expected from size effect alone, implying substantial microstrain within the lattice. These combined features confirm the presence of significant lattice distortion, a symbol of HEA nanowires. Thus, although the material exhibits a single-phase structure, its XRD pattern showed clear evidence of random atomic distributions and lattice strain (Figure 29B).
(ii) Strain mapping by 4D-STEM: Yao and co-workers used 4D-STEM to reveal both the structural and strain distributions within single 15-element HEA nanocrystals (Figure 29C).^192^ By collecting a full diffraction pattern at every probe position, the authors reconstructed spatially resolved strain and lattice distortion maps. The results showed nanoscale tensile and compressive strain fluctuations (±0.5%) throughout the particle despite the overall single-phase FCC structure. These localized distortions indicate CSRO lattice irregularities arising from extreme multielement mixing, confirming that HEA nanocrystals are not perfectly random solutions but contain atomic-scale heterogeneity crucial for understanding stability and catalytic behavior.
(iii) 3D atomic structures and CSRO revealed by AET: Moniri and co-workers utilized AET to reconstruct the 3D atomic configurations of NiPdPt-based medium- and HEA nanocrystals.^223^ The resulting atomic models provided direct visualization of atomic positions, local strain tensors, lattice distortions, dislocations, and twin boundaries within individual nanocrystals (Figure 29D). To quantify local ordering, the authors introduced the CSRO parameter (α_ij_), where positive values denote atomic segregation and negative values represent intermixing between element pairs (Figure 29E). The AET results confirmed that the HEA nanocrystals maintained a single-phase FCC lattice but exhibited stronger atomic-scale lattice distortions and more nanoscale regions of CSRO were identified, with their spatial variations closely correlated to local strain distributions. Notably, twin boundaries were found to form preferentially in areas with energetically unfavorable CSRO, where normally immiscible elements locally mix, whereas regions with favorable ordering remained free of such defects. These findings provide direct, atomically resolved evidence that local chemical ordering governs strain heterogeneity and defect formation in HEAs.

### 4.3 Surface Chemical Environment and Electronic Structure

#### 4.3.1 Importance

The surface of HEA nanocrystals is where interactions with the environment or reactants occur, which is especially vital in catalysis. The surface chemical environment characterization entails which elements are present at the surface quantitatively, their oxidation states, and the electronic structure (e.g., *d*-band occupancy and orbital hybridization). In multielement alloys, it is common that the surface composition deviates from the bulk due to phenomena like surface segregation (certain elements preferentially migrating to or away from the surface), oxidation (surface oxide formation on reactive elements), or energy-driven migration or oxidation throughout the catalytic process. For example, in traditional bimetallic nanocrystals, often the more noble element (lower surface energy) is enriched at the surface, while a more oxophilic element might segregate to the surface, forming a shell and/or even being oxidized.^211^ HEA nanocrystals, being complex mixtures, can exhibit similar effects, but predicting which element will dominate the surface is nontrivial. Moreover, ligand molecules from synthesis or reaction conditions can dynamically change the surface composition. Thus, characterizing the surface is essential to know the “true” composition and chemical state, which in turn controls the catalytic performance. Additionally, understanding the electronic structure, such as valence band properties and orbital hybridization between different metals, helps link the material’s composition to its chemical reactivity. This part of characterization aims to determine *i*) the surface chemistry of HEA nanocrystals in terms of which atoms are present at the surface and what oxidation states they adopt and *ii*) electronic structure and coordination environment in high-entropy mixing.

Key characterization techniques for surface chemical environment and electronic structure include:

(i) XPS: it is a technique for surface analysis (sensitive to the top 5 nm of a material). It provides information on elemental composition at the surface and, importantly, the chemical state of these elements via binding energy shifts. For HEA nanocrystals, XPS can tell us if any component is present in an oxidized form or if there is any electron transfer between elements. By comparing XPS peak areas with sensitivity factors, one can also estimate the surface atomic percentages of each element.^23^ Often, a discrepancy between the XPS-derived surface composition and bulk composition indicates surface segregation. Depth profiling with XPS (sputtering away layers or angling the detector for different escape depths) can further map how composition changes from the surface into the bulk.
(ii) Low-energy ion scattering (LEIS): it is a specialized surface technique that is extremely surface-specific, effectively probing the outermost atomic layer and avoiding the issue of Auger peak overlaps. In LEIS, noble gas ions (mostly, He^+^ or Ne^+^) at low energies (1–8 keV) scatter off surface atoms. By measuring the energy of the scattered ions, one identifies which elements are on the surface. LEIS can also be combined with sputter depth profiling to extract layer-by-layer compositions up to 10 nm, yielding a complete picture of near-surface chemical gradients and structural evolution. This technique is particularly useful for HEAs because the topmost surface could be enriched in a certain element even if the next few layers are different.^224,225^

(iii) XAS: this includes XANES and EXAFS for element-specific information on the local atomic and electronic structure. In the context of surface/electronic characterization, XANES (extending *ca*. 50 eV above the absorption edge) is particularly valuable for determining the oxidation state and electronic structure of specific elements in an HEA. Because XANES features (edge position, shape, white line intensity) are sensitive to the valence state and the local chemical environment, they can be used to infer if an element in the particle is in the zerovalent (metallic) state or partially oxidized. In an alloy, these features might shift or change due to hybridization with other elements. EXAFS (from *ca*. 50 to >1000 eV beyond the edge) provides local structure information (neighbor distances, coordination numbers), and can complement surface analysis by indicating, for example, if a certain element has a high-O coordination (likely an oxidized surface species) or is mostly surrounded by metals (metallic state). The major advantage of XAS is its element selectivity—one can probe each element of the HEA separately by tuning the X-ray to that element’s absorption edge. This is ideal for multicomponent alloys as one can obtain a sort of “partial” view of the structure around each element.^216^

#### 4.3.2 Examples and Findings

In-depth characterization using surface-sensitive and element-specific techniques reveals how oxidation states, coordination environments, and charge distribution vary across atomic layers and between constituent elements:

(i) Surface chemical environment with depth profile: Kogler and co-workers demonstrated that the surface composition of HEA can deviate from its nominal bulk stoichiometry, with significant implications for catalytic and electrochemical behavior.^226^ Using a combination of XPS and LEIS, they analyzed the surface and near-surface composition of a NiFeCoMnCr HEA nanocrystal prepared using vacuum-arc melting under different conditions. XPS, which probes several nanometers in depth, revealed Ni enrichment in subsurface regions, a decrease in oxygen signal with progressive sputtering, and pronounced Cr enrichment within the surface oxide. In contrast, LEIS, offering subnanometer surface sensitivity, showed that the outermost (*ca*. 0.6 nm) oxide layer was enriched in Mn and Cr, with Cr peaking slightly below the extreme surface, suggesting a Mn-rich termination layer. Elements such as Fe, Co, and Ni were found at significantly lower concentrations on the outer surface compared to bulk levels determined by EDS and X-ray fluorescence (XRF), but their concentrations increased gradually toward the metallic interior. These findings indicate a double-shelled surface structure for the CrMnFeCoNi HEA nanocrystals: an outer oxide/hydroxide layer dominated by Mn and Cr, followed by a Ni-rich interfacial region exhibiting partial oxidation, and finally a Cr/Mn-depleted metallic bulk (Figure 30A).
(ii) Local atomic and electronic structures: Hsiao and co-workers employed synchrotron XAS to elucidate the local coordination and electronic structure of Pd@Pd_0.2_Pt_0.2_Ir_0.2_Ru_0.2_Rh_0.2–4L_ core–shell nanocubes.^82^ XANES spectra of all constituent elements differed notably from their metallic and oxide references, revealing modified orbital hybridization and electronic states arising from multimetallic mixing. FT-EXAFS analysis exhibited alloy-type doublet peaks between R = 1.9–2.8 Å, characteristic of high-entropy solid solutions. Coordination number fitting showed strong 4d–5d atomic interactions (Pt, Ir, Ru, Rh), confirming homogeneous atomic mixing within the shell. In contrast, Pd displayed higher Pd–Pd coordination due to contributions from the underlying core. Across all elements, metallic bonding dominated, with only minor oxide components detected. Electronic-structure analysis revealed element-specific charge redistribution within the HEA shell. The Pd K-edge shifted to a lower energy, indicating electron transfer from neighboring atoms. Weak Ru–O, Rh–O, and Ir–O features suggested partial oxidation and low valence states, whereas Pt showed a slightly higher oxidation state but no detectable Pt–O coordination, implying electron donation to adjacent atoms and partial 5d-band depletion. Wavelet-transform (WT) EXAFS further indicated subtle radial and k-space broadening for Pt, Ir, Ru, and Rh, consistent with lattice distortion and heterogeneous local environments. Altogether, these observations confirm that the HEA shells of the nanocubes indeed form a homogeneous solid-solution without phase segregation, together with distinct electronic redistribution among constituent metals (Figure 30B).

**Figure 30:**
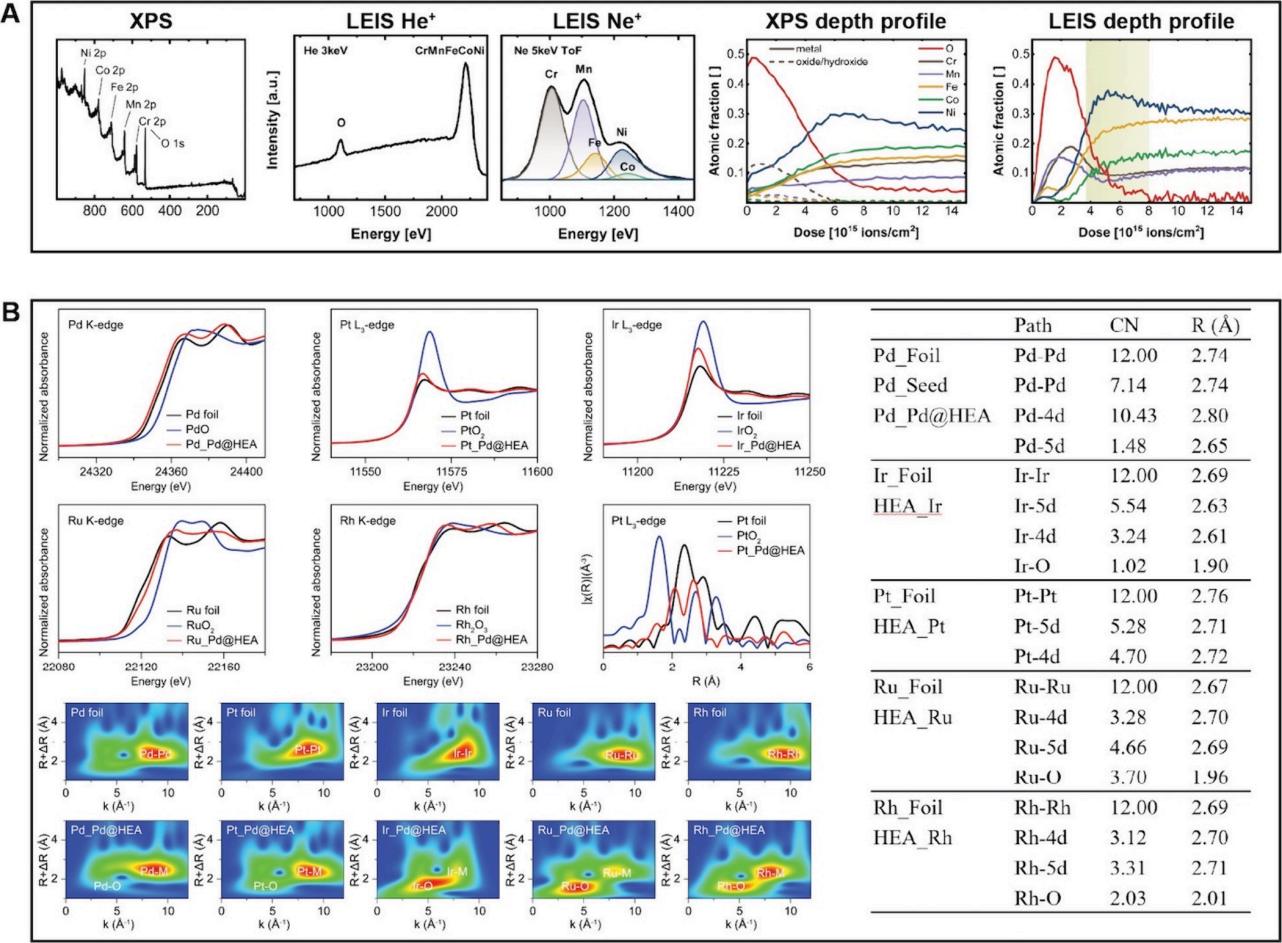
Characterizations of surface chemical environment and electronic structure of HEA nanocrystals. (A) XPS and LEIS to determine the surface composition of a NiFeCoMnCr alloy, including sputter depth profiles. (B) XAS revealing the electronic structure and local coordination environment of constituent elements in Pd@Pd_0.2_Pt_0.2_Ir_0.2_Ru_0.2_Rh_0.2–4L_ core–shell nanocubes. The image in (A) was reproduced with permission from ref 226. Copyright 2024 Royal Society of Chemistry. The image in (B) was reproduced with permission from ref 82. Copyright 2024 Wiley-VCH.

### 4.4 *In Situ* and Operando Characterization

*In situ* and operando characterization techniques are essential for elucidating the dynamic structural and electronic changes that HEAs undergo under synthetic and catalytic conditions. The real-time analyses provide critical insights into formation kinetics, phase transformations, active-site evolution, reaction intermediates, and surface reconstruction, among others. In one study, Liao and co-workers employed *in situ* Raman spectroscopy to monitor OER processes on FeCoNiCuMo–O and CoNiCuMo–O catalysts.^227^ Both catalytic samples were prepared using the CTS method. The Raman spectra collected prior to electrolyte immersion and at open-circuit potential (OCP) showed no significant changes, confirming that laser exposure did not alter the sample structure. New spectral features emerged when the applied potential reached 1.4 V_RHE_, coinciding with the onset of OER activity. As the potential was increased from 1.4 to 1.6 V_RHE_, a distinct Raman band appeared at 566 cm^–1^, corresponding to the lattice vibrational mode of metal oxyhydroxides (M–OOH). Upon reversing the potential sweep, this band disappeared, indicating the reversible formation and reduction of M–OOH species during the reaction. Notably, FeCoNiCuMo–O exhibited a more intense M–OOH signal than CoNiCuMo–O, accompanied by a more pronounced O–O vibrational feature near 1,170 cm^–1^ within the same potential range. The concurrent evolution and disappearance of these peaks confirm that both catalysts follow a dual mechanism involving adsorption evolution and lattice oxygen. The stronger and more persistent features in FeCoNiCuMo–O suggest enhanced participation of both pathways, explaining its superior OER activity (Figure 31A).

**Figure 31:**
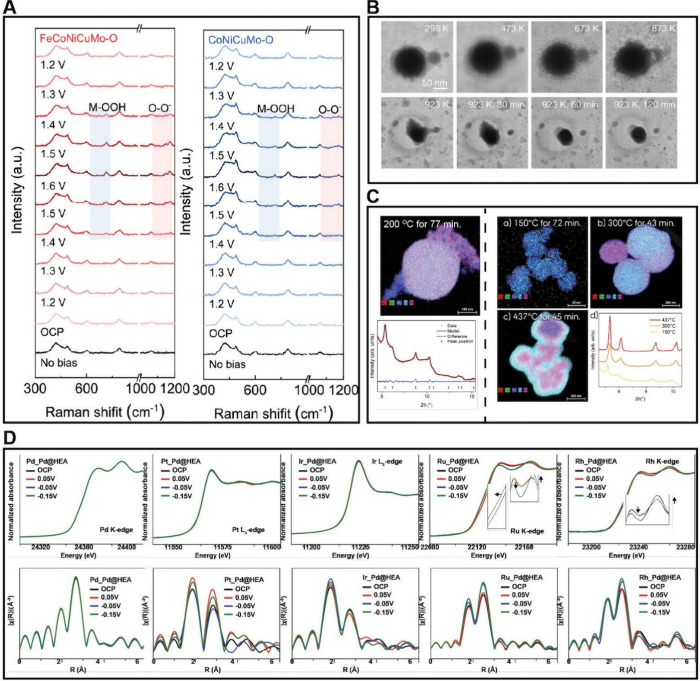
*In situ* and operando characterization of HEA nanocrystals. (A) *In situ* Raman spectra of FeCoNiCuMo–O and reference CoNiCuMo–O catalysts during the OER. (B) *In situ* TEM monitoring the liquid-metal-assisted synthesis process from GaFeMnNiCu precursor to alloy nanocrystals. (C) STEM–EDS elemental mapping of samples synthesized at different reaction temperatures, together with corresponding diffractograms from *in situ* XRD, supporting the autocatalytic formation mechanism of PtIrPdRhRu nanocrystals. (D) *In situ* XANES and FT-EXAFS spectra of Pd@Pd_0.2_Pt_0.2_Ir_0.2_Ru_0.2_Rh_0.2–4L_ core–shell nanocubes during HER. The image in (A) was reproduced with permission from ref 227. Copyright 2025 American Chemical Society. The image in (B) was reproduced with permission from ref 166. Copyright 2023 Springer Nature. The image in (C) was reproduced with permission from ref 228. Copyright 2020 Wiley-VCH. The image in (D) was reproduced with permission from ref 82. Copyright 2024 Wiley-VCH.

*In situ* XRD and TEM have become crucial for uncovering the structural evolution and phase formation mechanisms of HEAs under synthetic conditions. These approaches enable direct, real-time observation of alloy nucleation, growth, and atomic mixing dynamics. Cao and co-workers employed *in situ* TEM to monitor the nucleation and evolution of HEA nanocrystals from a liquid-metal precursor, as shown in Figure 31B.^166^ Using a temperature-controlled heating stage, they visualized a distinct fission–fusion process: larger liquid alloy droplets periodically fragmented into smaller nuclei, which subsequently merged. The repeated splitting and merging facilitated uniform atomic mixing within each droplet, ultimately yielding a homogeneous, solid solution upon cooling. Complementary *in situ* XRD and STEM–EDS studies by Broge and co-workers elucidated the temperature-dependent phase formation and elemental distribution of PtIrPdRhRu nanocrystals.^228^ A single-phase FCC PtIrPdRhRu HEA nanocrystal with uniform elemental distribution formed at 200 °C after 77 min via an autocatalytic reduction mechanism (Figure 31C, left). For the reference groups (150 °C, 300 °C, and 437 °C in Figure 31C, right), an FCC alloy also formed at 150 °C after 72 min but showed compositional imbalance, with Pd enrichment and Ru/Ir depletion. This data implies the autocatalytic reduction of other metal ions initiated by Pd nuclei has a slower incorporation rate at a lower temperature. However, increasing the temperature to 300 and 437 °C led to compositional segregation, with Pd concentrated in the core and Ir enriched in the shell. This trend can be attributed to the higher and lower reduction temperatures for Pd^2+^ and Ir^3+^, respectively. Corresponding powder XRD data confirmed partial phase splitting, as indicated by asymmetric FCC peaks with shoulders caused by crystalline domains with different lattice parameters. New peaks appeared at the highest temperature could be assigned to the HCP structure, as Ru only took the FCC structure at reaction temperatures below 250 °C.

Understanding which elements in an HEA serve as the catalytic active sites and whether the alloy maintains its structural integrity under reaction conditions requires operando XAS. This technique provides element-specific insight into the oxidation states and local coordination environments during catalysis. Hsiao and co-workers applied *in situ* XAS to examine a catalyst based on Pd@Pd_0.2_Pt_0.2_Ir_0.2_Ru_0.2_Rh_0.2–4L_ nanocubes during HER.^82^ Notably, for XANES, at the Ru K-edge, potential-dependent oscillations at 22,135 and 22,160 eV intensified and diminished reversibly when the applied potential was switched from anodic to cathodic conditions, implying hydrogen adsorption at Ru surface sites. Similarly, variations in the Rh K-edge spectra, including an isosbestic point at 23,255 eV, suggested the participation of Rh in hydrogen ion adsorption. Importantly, the Ru absorption edge shifted to lower energy during HER, indicating electron enrichment on these sites, whereas Rh and other metal edges remained largely stationary, confirming that Ru acted as the main catalytic sites during HER. Furthermore, the peak positions of different metal sites in the FT-EXAFS spectra remained essentially unchanged for all elements while the catalyst was held at operating potentials, demonstrating an exceptional coordination stability of the HEA’s surface. This data indicates that none of the alloy constituents was leached out, oxidized, or converted to new phases (Figure 31D).

### 4.5 Computation and Data-Driven Design

Owing to their vast numbers of chemically distinct surface sites, HEA nanocrystals offer a good opportunity to tune catalytic activity and selectivity. However, their compositional complexity and local heterogeneity make rational design of catalysts still challenging. First-principles calculations such as DFT are therefore essential for understanding site-specific adsorption and reaction mechanisms, while machine-learning approaches can accelerate the identification of optimal compositions and key activity descriptors. The following subsections summarize recent advances using DFT and machine learning to model and rationalize the catalytic behavior of HEA nanocrystals.

#### 4.5.1 DFT Calculations

Unlike uniform catalytic surfaces that present only a few discrete adsorption environments, HEA surface, composed of randomly distributed multiple principal elements, exhibits a wide continuum of adsorption energies. This compositional and structural complexity holds great promise for optimizing catalytic activity by enabling surface sites with finely tuned binding energies that align with the Sabatier principle. However, the rational design of HEA catalysts is impeded by the limited understanding of how the local structure influences intermediate adsorption behavior. To address this, quantum-chemical approaches such as DFT are employed to investigate the interactions between reaction intermediates and diverse surface sites. Due to the inherently statistical distribution of atoms on HEA surfaces, it is critical to sample a large number of representative local configurations, often exceeding hundreds or even thousands, to accurately capture the mean adsorption behavior and its statistical distribution. Without such extensive sampling, theoretical predictions may fail to reflect the true nature of the solid-solution HEA surface observed in experiments.

A representative case is the HER under acidic conditions, where hydrogen adsorption free energy (Δ*G*_H*_) serves as a key descriptor of catalytic performance.^133^ It is well established that optimal catalytic activity is achieved when Δ*G*_H*_ is close to zero, indicating a balanced interaction between the catalyst surface and adsorbed hydrogen. To understand the exceptional HER activity of atomically mixed Pd_0.2_Pt_0.2_Ir_0.2_Ru_0.2_Rh_0.2_ cubic nanoframes, DFT calculations were carried out to evaluate Δ*G*_H*_ across several PdPtIrRuRh surface models with different facets and structural features, including {100}, {110}, and {110} containing either atomic vacancies or step sites (Figure 32A). The atomic arrangements of these surfaces give rise to distinct adsorption geometries, such as hollow, bridge, and atop sites, with varying coordination environments. Among over one hundred sampled configurations, as shown in Figure 32B, the {110} surface exhibited the smallest average Δ*G*_H*_ (−0.02 eV), closely approximating the ideal value and thus offering nearly optimal hydrogen binding. Surfaces with defects, such as {110} facets containing vacancies (−0.09 eV) or step sites (−0.14 eV), showed slightly stronger adsorption, while the {100} surface displayed weaker performance with an average Δ*G*_H*_ of −0.20 eV. These findings also suggest that subtle variations in local atomic arrangements, such as coordination number or surface roughness, can significantly influence catalytic performance, in line with experimental observations.

**Figure 32:**
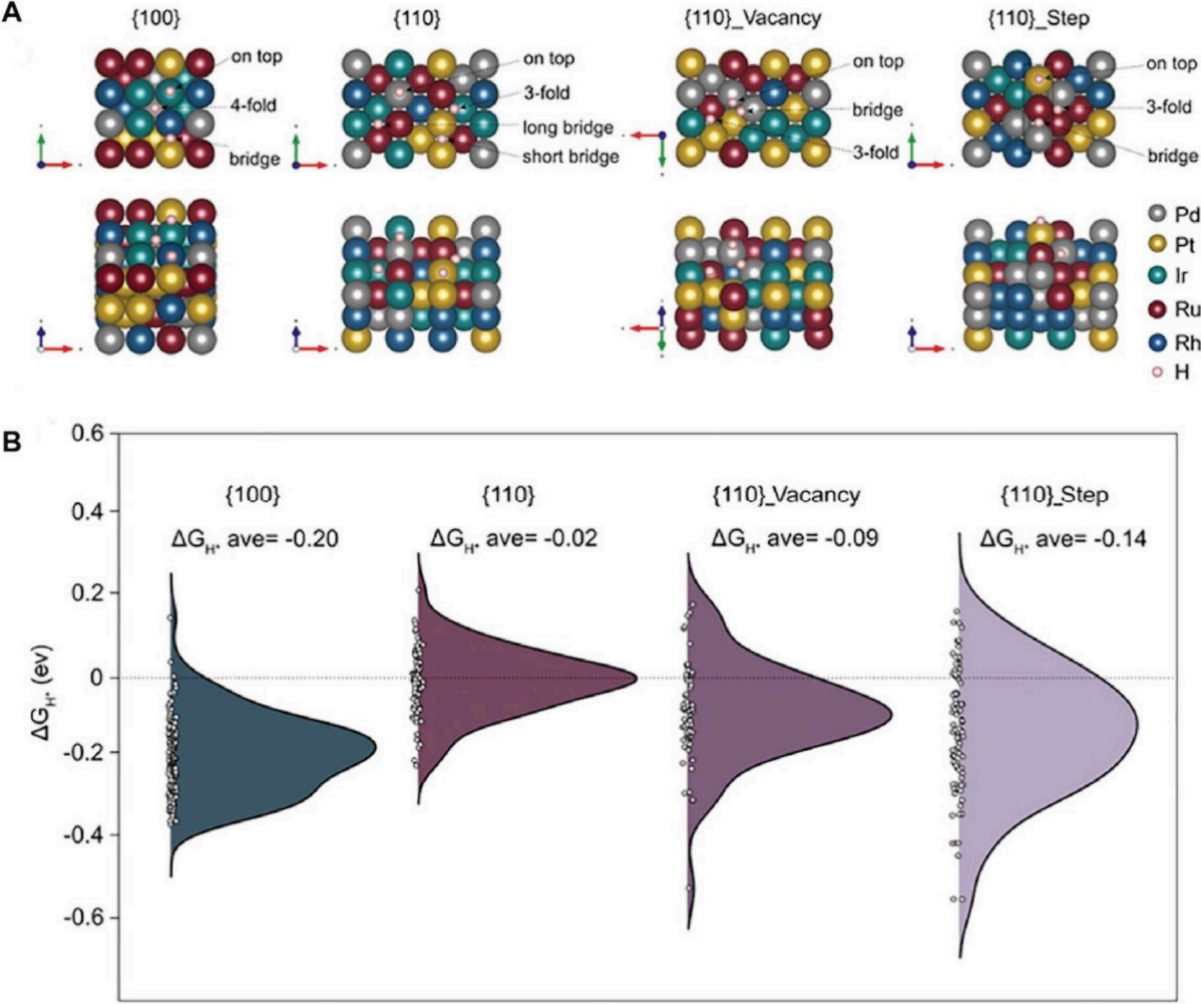
DFT calculations of hydrogen adsorption free energy (Δ*G*_H*_) on the PdPtIrRuRh HEA surface. (A) Configurations of hydrogen adsorption on four representative HEA surface structures: {100}, {110}, {110} with vacancies, and {110} with a step atom. (B) Calculated Δ*G*_H*_ values corresponding to the {100}, {110}, {110} with vacancies, and {110} with step atoms. Reproduced with permission from ref 133. Copyright 2024 Wiley-VCH.

Building upon this understanding, a recent study further challenges the classical view of catalytic design by introducing the concept of an “Unusual Sabatier Principle” in the context of HEA catalysts toward the HER.^33^ Traditionally, the Sabatier principle posits that a catalyst achieves optimal performance when the adsorption free energy of key intermediates, such as hydrogen in acidic HER approaches zero, striking a balance between adsorption and desorption. However, the applicability of this principle becomes less straightforward for HEAs due to their randomly distributed multiple principal elements, which give rise to a broad and continuous spectrum of surface binding environments. Instead of exhibiting a single well-defined Δ*G*_H*_, the HEA surface was shown to possess a Gaussian-like distribution of adsorption energies, characterized by an ensemble mean (μ) and standard deviation (σ). This finding suggests that evaluating a single adsorption site is insufficient for capturing the catalytic behavior of a solid-solution HEA; rather, a statistical distribution of active sites must be considered. Importantly, in this framework, optimal catalytic performance is not solely determined by minimizing the ensemble-averaged hydrogen adsorption free energy (|μ|), but also by achieving a sufficiently broad σ, which reflects the diversity of adsorption environments across the HEA surface. This distribution enables distinct surface sites to specialize in different catalytic roles, strong-binding sites (Δ*G*_H*_ < μ–σ) can facilitate the adsorption and activation of intermediates, while weak-binding sites (Δ*G*_H*_ > μ + σ) promote desorption and product release.

Moreover, a key mechanistic insight is the observation of intermediate spillover behavior between sites of varying binding strengths, allowing spatial decoupling of the Volmer step (proton adsorption/activation) from the Tafel or Heyrovsky steps (H_2_ formation). This multisite cooperation fundamentally departs from classical single-site volcano-type interpretations and reveals a new descriptor space for HEA catalyst design based on both μ and σ. These concepts are illustrated in Figure 33, which summarizes the reaction mechanism derived from DFT calculations. Figure 33A shows the statistical distribution of Δ*G*_H*_ across the FCC-, HCP-, and bridge-type sites under 5.9% compressive strain on PtFeCoNiCu HEA. Unlike the narrow adsorption-energy peak of conventional catalysts, the HEA exhibits a broad, quasi-Gaussian distribution with a broader σ, indicating multiple nonequivalent atomic sites with different local electronic structures. This heterogeneity enables spatial separation of active centers for Volmer (strong H* adsorption) and Heyrovsky/Tafel (weaker adsorption), while the diffusion regions are identified in the green-shaded areas where H* spillover occurs. Figure 33B, C compare the reaction pathways: on Pt(111), HER follows the classic Sabatier volcano, limited by sluggish hydrogen desorption step; in contrast, on the HEA(111), the energy barriers for both Volmer–Heyrovsky and Volmer–Tafel steps are nearly zero, namely with no potential-limiting step, due to efficient H* diffusion. Figure 33D and 33E quantify this spillover, identifying low diffusion barriers (0.124–0.232 eV) of hydrogen between different adsorption sites on the HEA surface via nudged elastic band (NEB) method. This low barrier confirms the feasibility of H* migration between strong- and weak-binding sites, resulting in simultaneous strong adsorption and fast product release, enabling efficient catalytic turnover by leveraging the surface heterogeneity. Therefore, this “Unusual Sabatier Principle” extends the original theory to embrace the intrinsic complexity of HEA surfaces. Rather than averaging out the binding energies, the (μ, σ) descriptor pair provides a practical framework for identifying and engineering HEA compositions with superior and robust catalytic performance across variable conditions.

**Figure 33:**
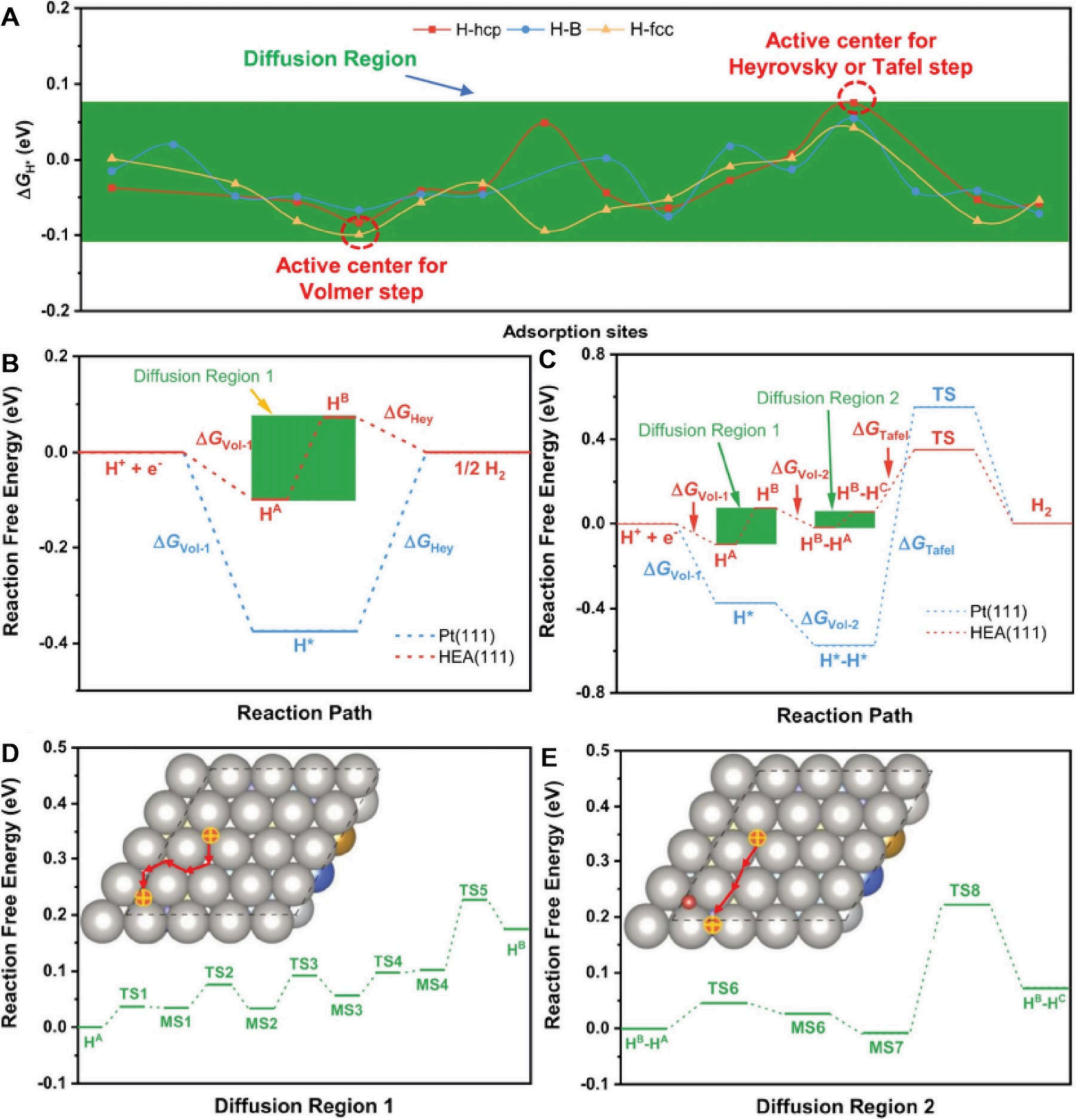
DFT analysis illustrating the unconventional Sabatier behavior of HEA catalysts during the HER. (A) Distribution of Δ*G*_H*_ on an HEA surface subjected to 5.9% compressive strain (5.9%-HEA), showing various adsorption configurations including HCP hollow, FCC hollow, and bridge sites. The red dashed circles highlight the active centers associated with the Volmer and Heyrovsky (or Tafel) steps, while the green-shaded regions denote the hydrogen diffusion region for adsorbed H*. (B) Volmer–Heyrovsky reaction pathway of HER on the 5.9%-HEA (111) surface compared with that on Pt(111). (C) Volmer–Tafel reaction pathway of HER on the 5.9%-HEA(111) surface and Pt(111). (D) H* spillover behavior within the diffusion region for the first adsorbed H* on the 5.9%-HEA(111) surface. (E) H* spillover behavior within the diffusion region for the second adsorbed H* on the 5.9%-HEA(111) surface. Reproduced with permission from ref 33. Copyright 2024 Springer Nature.

Recent advances further support this framework by introducing electronic descriptors that explicitly account for the local coordination environment of active sites on HEA surfaces.^229^ In particular, a descriptor denoted as Ω, defined as a linear combination of the *d*-band filling of the host metal center (*f*_*d*_) and the average electronegativity of neighboring atoms (*χ̃*_N_), has been proposed to rationalize the binding strength of adsorbates such as *O. This formulation captures the synergistic influence of both the central metal’s electronic configuration and its surrounding coordination environment, two aspects that are inherently diverse and spatially distributed on HEA surfaces. By applying this Ω descriptor across a broad sampling of surface configurations in a Pd–Ir–Pt–Au–Ag-based HEA space, the study constructed a predictive activity map that identifies optimal local environments for electrocatalysis. These findings reinforce the notion that catalytic activity on HEAs arises not from average properties, but from a distribution of electronically distinct microenvironments. Such insights not only corroborate the (μ, σ) conceptual framework but also offer a complementary, physically interpretable route for screening and tailoring HEA compositions at the atomic scale. In summary, integrating DFT with advanced descriptors such as adsorption energy distributions and local-environment-sensitive parameters has significantly advanced the theoretical framework for HEA catalyst design. These approaches emphasize the importance of sampling diverse atomic configurations and capturing site-specific properties, rather than relying on averaged values alone. Such a distribution-based understanding enables more accurate prediction and optimization of HEA compositions for high-performance electrocatalysis.

#### 4.5.2 Machine Learning

Recent advances in machine learning are transforming the way researchers approach catalyst discovery, particularly in systems with extreme compositional and structural complexity such as HEA nanocrystals. Traditional first-principles methods like DFT are powerful but computationally prohibitive for the exhaustive exploration of multicomponent surfaces with highly heterogeneous atomic configurations. In this context, machine learning models have emerged as scalable alternatives to efficiently predict key catalytic descriptors, such as the adsorption energies of intermediates, across vast numbers of potential active sites.

As illustrated in Figure 34, a typical machine learning-based prediction pipeline for HEA catalysts begins with the transformation of physical inputs, such as elemental composition, atomic structure, and local coordination environment, into machine-readable numerical formats.^230^ These features can include statistical descriptors of elemental properties (e.g., electronegativity, atomic radius, and valence electron count), as well as geometric metrics derived from pairwise or triplet atom correlations and local site symmetry. Once encoded, these inputs are fed into a variety of machine learning algorithms ranging from conventional models like linear regression, support vector regression, and kernel ridge regression, to more sophisticated techniques such as deep neural networks, gradient boosting regression, and convolutional neural networks (1*D*/2D/3D), depending on the complexity and nature of the data set. Among the latest developments, graph neural networks have proven particularly effective in modeling disordered and nonuniform systems like HEAs. By conceptualizing atomic structures as graphs, where atoms act as nodes and their interactions form edges, graph neural networks enable direct learning from structural representations without the need for predefined feature engineering. Through iterative message passing between neighboring atoms, graph neural networks can efficiently capture local chemical environments and long-range structural correlations, thereby providing accurate predictions of site-specific properties such as adsorption energies or reaction barriers. In parallel, other end-to-end learning frameworks, including variational autoencoders and transformer architectures, are being explored for their ability to model raw structural or electronic data. These models bypass traditional descriptor-based workflows entirely, offering greater flexibility in learning hidden patterns within highly entropic or amorphous systems. Such approaches are especially promising for HEAs, where the diversity of local environments poses a major barrier to conventional modeling strategies.

**Figure 34:**
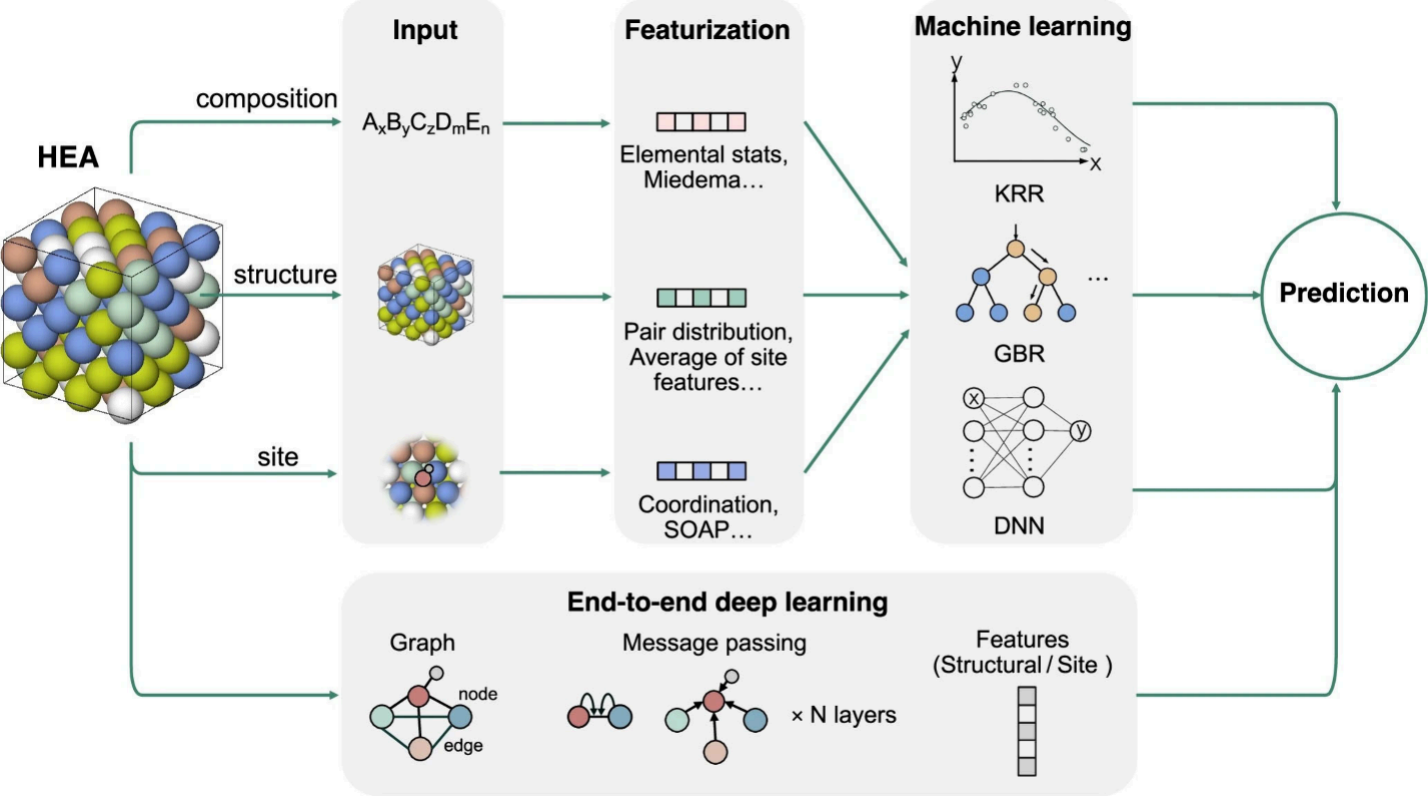
Overview of machine learning procedures applied to HEAs. Reproduced with permission from ref 230. Copyright 2025 Springer Nature.

Beyond predicting adsorption energies, machine learning provides a powerful approach for navigating the vast compositional space of HEAs to identify optimal combinations that balance catalytic activity, durability, and cost. In a representative study, Batchelor and co-workers developed a supervised learning framework using physically meaningful descriptors, such as elemental electronegativity, *d*-band center, and atomic radius, to model the catalytic performance of multimetallic systems toward the ORR.^231^ Through a combination of regression modeling and principal component analysis, they uncovered key compositional trends that correlate with enhanced activity. This approach enabled rapid down-selection from a large design space without the need for exhaustive DFT calculations, which will become impractical for systems beyond ternary or quaternary complexity. Importantly, the trained models demonstrated strong generalizability even with limited data, highlighting the utility of machine learning in efficiently guiding the design of complex alloy catalysts.

This capability has recently been scaled up through autonomous discovery platforms that combine machine learning predictions with automated high-throughput experimentation. A striking example is the Closed-Loop Rapid Electrocatalyst Screening system reported by Zhang and co-workers, which evaluated over 900 distinct catalyst chemistries and conducted more than 3,500 electrochemical tests in just three months.^232^ This system integrates machine learning algorithms to propose new catalyst formulations, robotic synthesis, and testing modules to generate experimental data, and iterative feedback loops that continuously refine the model. Through this approach, an octonary PdPtCuAuIrCeNbCr HEA catalyst was identified with a 9.3-fold enhancement in cost-specific performance toward electrochemical formate oxidation relative to pure Pd benchmark. Such results demonstrate the power of data-driven optimization not only for identifying high-performance compositions, but also for revealing nonintuitive element combinations that may be overlooked in empirical or human-guided workflows.

Collectively, these developments highlight the enormous potential of coupling compositional and structural tunability in HEA systems with data-driven approaches to accelerate rational catalyst design. From predicting adsorption energies at the atomic scale to identifying optimal multimetallic combinations and validating candidates through automated experimental workflows, machine learning has become an indispensable part of the catalyst discovery pipeline. As these methods continue to evolve, the convergence of machine learning, high-throughput experimentation, and advanced characterization is expected to unlock previously inaccessible regions of the HEA design space, leading to the discovery of highly active, robust, and cost-effective electrocatalysts for a wide range of energy conversion and chemical transformation processes.

## 5 Unique Properties

Multimetallic or HEA nanocrystals are characterized by a range of unique properties because of the intricate interplay among four intrinsic effects arising from high entropy, lattice distortion, sluggish diffusion, and cocktail mixing.^5,12^ While these effects are well established in bulk HEAs,^9,233,234^ their manifestations become markedly amplified and strongly coupled at the nanoscale, where surface energy, atomic coordination, and diffusion kinetics dominate material behavior.^235,236^ Recent advances in operando spectroscopy,^19,81^ high-resolution electron microscopy,^166,223^ and theoretical modeling^229,237^ have provided quantitative insights into how these multiscale effects cooperatively govern surface reactivity and structural stability. The increased configurational entropy arising from multielement mixing promotes the thermodynamic stabilization of disordered solid-solution phases over ordered intermetallics, thereby broadening the accessible compositional space for material design. Within such a compositionally complex lattice, the coexistence of atoms with different radii and electronegativities induces pronounced lattice distortions, generating local strain fields and electronic heterogeneity that modulate surface binding and adsorption energetics. Meanwhile, the diverse bonding environments inherent to multielement systems give rise to sluggish diffusion, manifested as a broad distribution of migration barriers that hinder long-range atomic transport and thereby suppress rapid phase transitions. Finally, the cocktail effect, arising from nonlinear coupling among multiple elements, leads to substantial deviations from the rule of mixtures and produces synergistically enhanced catalytic activity.^12^ Together, these effects define the structure–property-performance relationships of HEA nanocatalysts. The following subsections discuss how they govern structural stability, electronic modulation, and surface reactivity.

### 5.1 High-Entropy Effect

The high-entropy effect plays a central role in the thermodynamic stabilization of random solid-solution structures in HEA nanocrystals. In multicomponent systems containing many principal elements, an increase in configurational randomness (*ΔS*_*mix*_) lowers the Gibbs free energy, contributing to the stabilization of disordered solid-solution states within a favorable enthalpy–entropy balance (Figure 35A). The resulting atomic-level mixing gives rise to a highly disordered structure with diverse local chemical environments. In consequence, HEA nanocrystals exhibit a broadened compositional space and a statistical distribution of adsorption sites with distinct electronic structures, enabling tunable catalytic activity and selectivity. For instance, Löffler and co-workers demonstrated that CrMnFeCoNi HEA nanocrystals exhibited ORR activity comparable to Pt/C, whereas systematic element-by-element removal led to a pronounced decrease in catalytic performance across all quaternary variants (Figure 35B).^159^ The single-phase solid-solution structure ensures uniform elemental distribution within the lattice, generating numerous active sites with distinct local coordination environments. Heteroatomic interactions modulate surface electronic states and adsorption energetics, creating a broad spectrum of catalytic motifs that approach optimal intermediate-binding strengths, thereby accelerating overall reaction kinetics.

**Figure 35:**
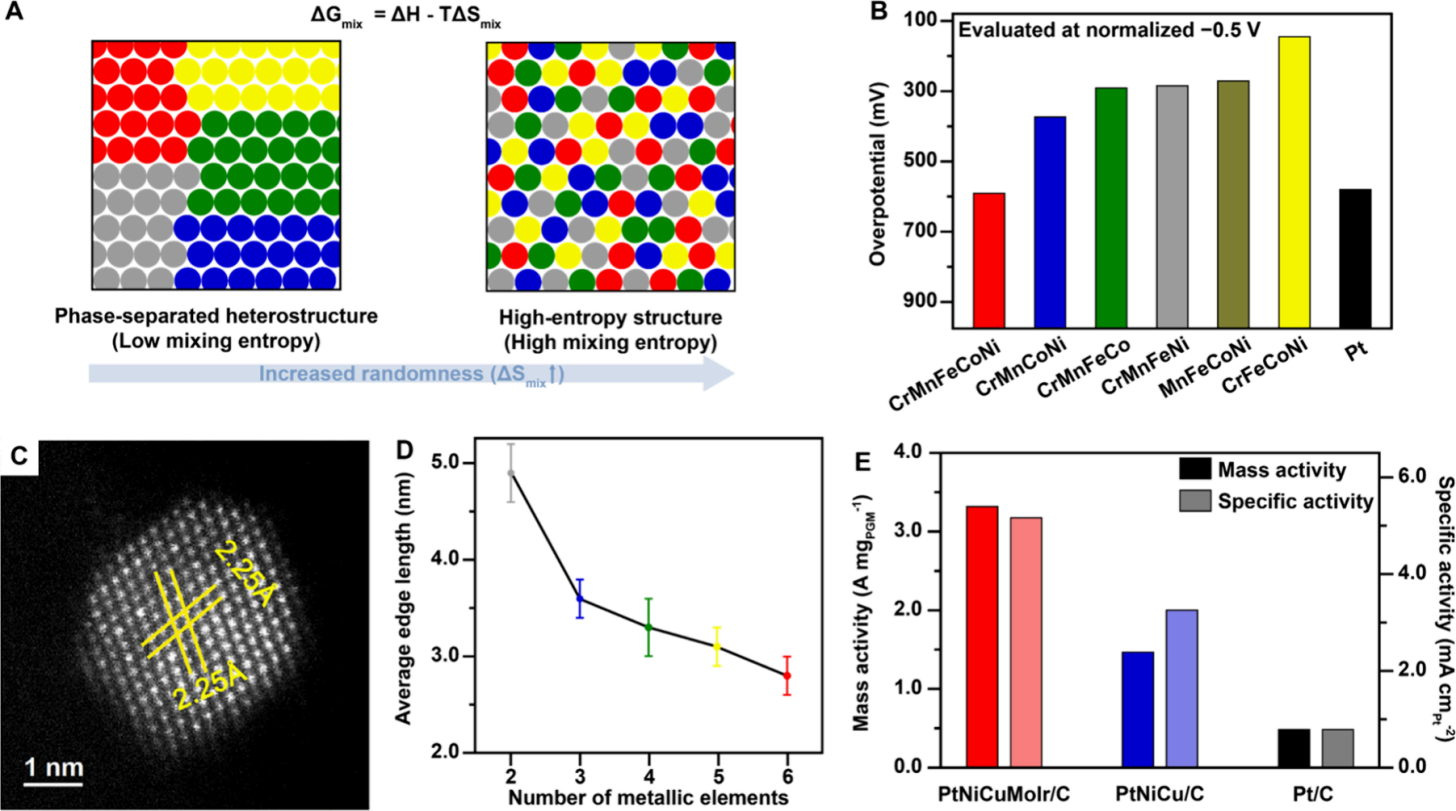
Origins and impacts of high-entropy effect. (A) Schematic illustration of the thermodynamic role of configurational entropy in HEAs. (B) Normalized ORR activity at −0.5 V_RHE_ for entropy-stabilized CrMnFeCoNi HEA, Pt, and quaternary alloys. (C) HAADF-STEM image of an isolated octahedral PtNiCuMoCoIr nanocrystal. (D) Edge-length variation of nanoscale octahedra as a function of the number of constituent elements. (E) Mass and specific activities of PtNiCuMoCoIr/C, PtNiCu/C, and Pt/C catalysts. The image in (B) was reproduced with permission from ref 159. Copyright 2018 Wiley-VCH. The images in (C–E) were reproduced from ref 31. Copyright 2025 Elsevier.

Beyond its thermodynamic role in stabilizing solid-solution phases, the high-entropy effect also plays a crucial role in shaping the catalytic behavior of HEA nanocrystals. The kinetic influence becomes particularly important in catalysis, where the nucleation behavior dictates the dispersion, morphology, and thus the accessibility of active sites. At the nanoscale, it profoundly influences nucleation and growth dynamics, which in turn determine particle size, surface structure, and active site distribution, key parameters that govern catalytic activity and durability.^67,238^ The coexistence of multiple elements increases the total configurational entropy and reduces the overall free energy of formation, thereby enhancing the thermodynamic stability of small nanocrystals. Concurrently, the reduced atomic mobility intrinsic to multielement systems suppresses diffusion-driven processes, such as Ostwald ripening, thereby mitigating grain coarsening and sintering to yield highly dispersed nanocrystals with a uniform size distribution.^59^ This entropy-induced stabilization of ultrasmall crystallites effectively increases accessible surface area and active-site exposure, thereby promoting superior catalytic turnover. Li and co-workers further demonstrated that increasing the number of constituent elements in octahedral HEA nanocrystals led to a pronounced reduction in particle size, accompanied by simultaneous improvements in mass activity and durability (Figure 35C–E).^31^ Collectively, the high-entropy effect enhances catalytic performance by stabilizing solid-solution structures and suppressing particle growth. This coupling between thermodynamic and kinetic effects underlies the superior behavior of HEA nanocrystals.

### 5.2 Lattice Distortion Effect

Lattice distortion is an intrinsic structural feature of HEAs that profoundly influences their electronic structures, reaction kinetics, and catalytic behavior.^223,239^ When elements with different atomic radii, electronegativities, and bonding preferences are incorporated into a common lattice, the ideal periodic arrangement is disrupted, resulting in variations in nearest-neighbor distances and local symmetry breaking.^240^ The atomic-scale heterogeneities create heterogeneous strain fields and variations in bonding strength, thereby reshaping the local electronic landscape. In essence, each atom resides in a chemically distinct coordination environment, leading to charge redistribution and modulation of the *d*-band center, including both upshift and downshift depending on local coordination, elemental interactions, and strain effects (Figure 36A).^29^ The resulting modulation of adsorption energies and activation barriers directly links structure to activity. In addition, lattice distortion creates undercoordinated and strained surface atoms with enhanced reactivity, facilitating bond activation and intermediate stabilization.

**Figure 36:**
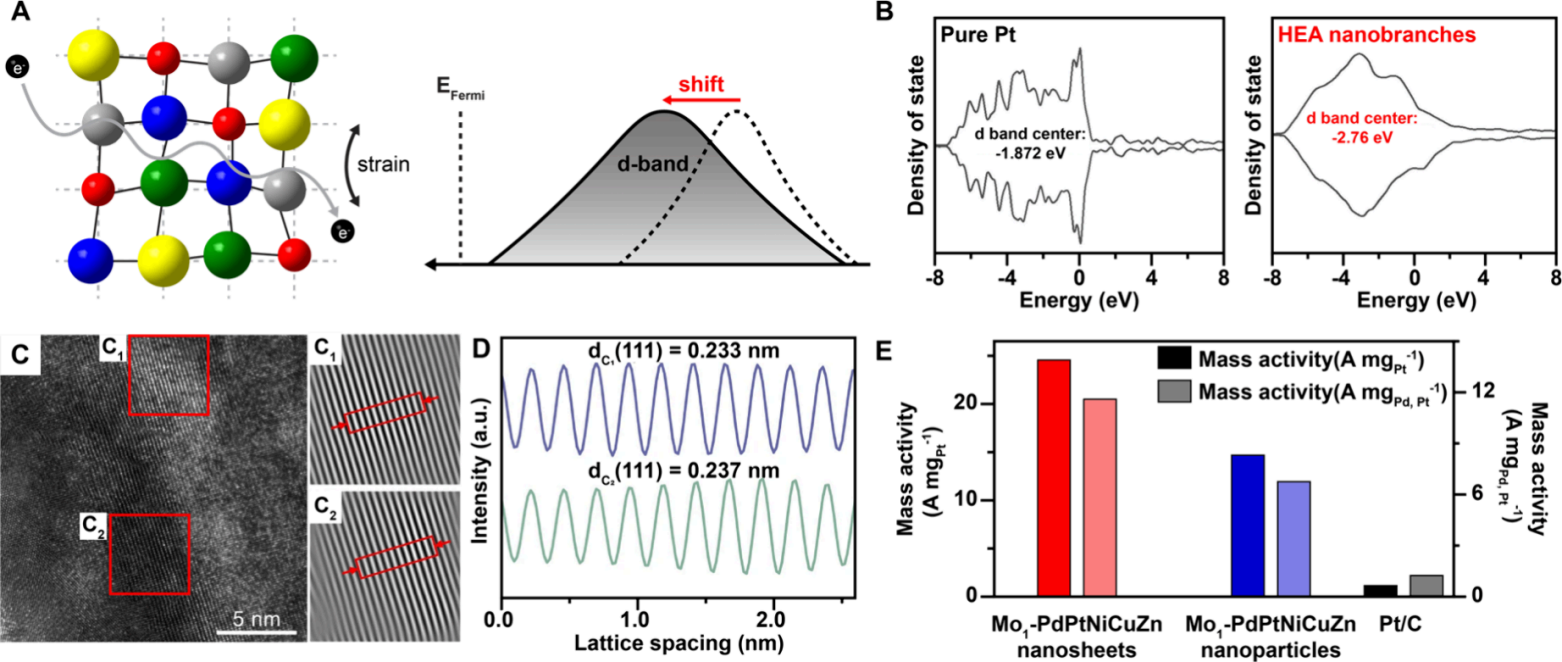
Origins and impacts of the lattice distortion effect. (A) Schematic illustration of size-mismatched atoms within a high-entropy lattice, showing heterogeneous strain fields that induce modulation of the *d*-band center (including both upshift and downshift) and broadening, thereby tuning the adsorption energies of reaction intermediates. (B) Comparison of *d*-orbital characteristics between Pt and HEA nanobranches (branched nanostructures), highlighting the downward shift of the *d*-band center. (C) HAADF-STEM image of the surface atomic arrangement of a single Mo_1_–PdPtNiCuZn nanosheet, and corresponding inverse FFT patterns (C_1_, C_2_) from the red-marked regions. (D) Intensity profiles extracted from the red areas in (C_1_) and (C_2_), showing local lattice distortion and tensile strain. (E) Mass activity toward MOR on Mo_1_–PdPtNiCuZn nanosheets, Mo_1_–PdPtNiCuZn nanocrystals, PdPtNiCuZn nanocrystals, and commercial Pt/C in 1.0 M KOH containing 1.0 M methanol. The image in (B) was reproduced with permission from ref 241. Copyright 2025 Elsevier. The images in (C–E) were reproduced from ref 51. Copyright 2024 Springer Nature.

Experimental and theoretical studies have elucidated the critical role of lattice distortion in mediating *d*-band energetics and catalytic enhancement. Luo and co-workers constructed WPtIrCoAu HEA nanobranches, referring to branched nanostructures consisting of a central framework with multiple outward-growing branches and compositional variation across different regions, to establish a quantitative link between lattice distortion and catalytic performance.^241^ The branched morphology introduced substantial strains and exposed high-index facets, producing a dense distribution of unsaturated active sites. Such structural distortion shifted the *d*-band center and altered the local electronic configuration, effectively lowering adsorption barriers and optimizing the activation energy toward the four-electron ORR pathway (Figure 36B). The HEA nanobranches exhibited a half-wave potential of 0.943 V_RHE_ and a mass activity of 1.75 A mg^–1^_Pt_, 11.7 times that of commercial Pt/C, with only 22% decay after 30,000 cycles. This study highlights how lattice distortion and charge redistribution collectively tune *d*-band energetics and reaction kinetics, establishing a direct link between nanoscale strain and catalytic durability.

Complementarily, He and co-workers demonstrated a synergistic coupling between strain and electronic modulation by incorporating oxophilic Mo single atoms into PdPtNiCuZn HEA nanosheets (Figure 36C–E).^51^ In comparison to the PdPtNiCuZn HEA nanocrystals without Mo, the incorporation of atomically dispersed Mo introduced additional lattice distortion and modified the local electronic structure. The atomic-size mismatch and compositional disorder of multicomponent systems, together with the ultrathin 2D morphology, introduced pronounced lattice distortion and intrinsic tensile strain. The incorporated Mo single atoms further amplified local strain and served as electronic promoters, redistributing charge density around adjacent Pt sites. As a result, both Mo_1_–PdPtNiCuZn HEA nanocrystals and nanosheets exhibited enhanced catalytic activity relative to the PdPtNiCuZn counterpart, highlighting the critical role of Mo incorporation in tuning the catalytic performance. In particular, the Mo_1_–PdPtNiCuZn HEA nanosheets delivered the highest activity, which can be attributed to the combined effects of lattice strain and increased active surface area associated with the ultrathin 2D structure. The dual modulations of lattice strain and charge distribution suppressed CO adsorption, promoted the formate pathway, and optimized the binding energetics of intermediates. The resulting catalyst exhibited high activity, excellent CO tolerance, and remarkable stability under alkaline conditions. These findings establish lattice distortion as a tunable electronic descriptor rather than a structural byproduct. By tailoring local strain and charge distribution, it becomes possible to engineer adsorption energetics and rationally design HEA electrocatalysts with improved activity, selectivity, and durability.

### 5.3 Sluggish Diffusion Effect

The sluggish diffusion effect arises from the kinetic constraints imposed by the chemical and structural complexity inherent to HEAs.^242,243^ Differences in atomic radius, electronegativity, and bonding preference create a rugged potential-energy landscape within the lattice, where each lattice site provides a distinct coordination environment. This local heterogeneity causes atomic and vacancy migration to follow a broad distribution of activation barriers, rather than a single migration energy, resulting in an overall diffusivity significantly lower than that of conventional alloys (Figure 37A).^15^ Although the vacancy concentrations in HEAs are similar to those in pure metals, vacancy migration is strongly hindered by lattice distortion and chemical disorder. The sluggish diffusion suppresses atomic mobility, grain growth, and phase segregation, thereby enhancing structural, thermal, and electrochemical stability.

**Figure 37:**
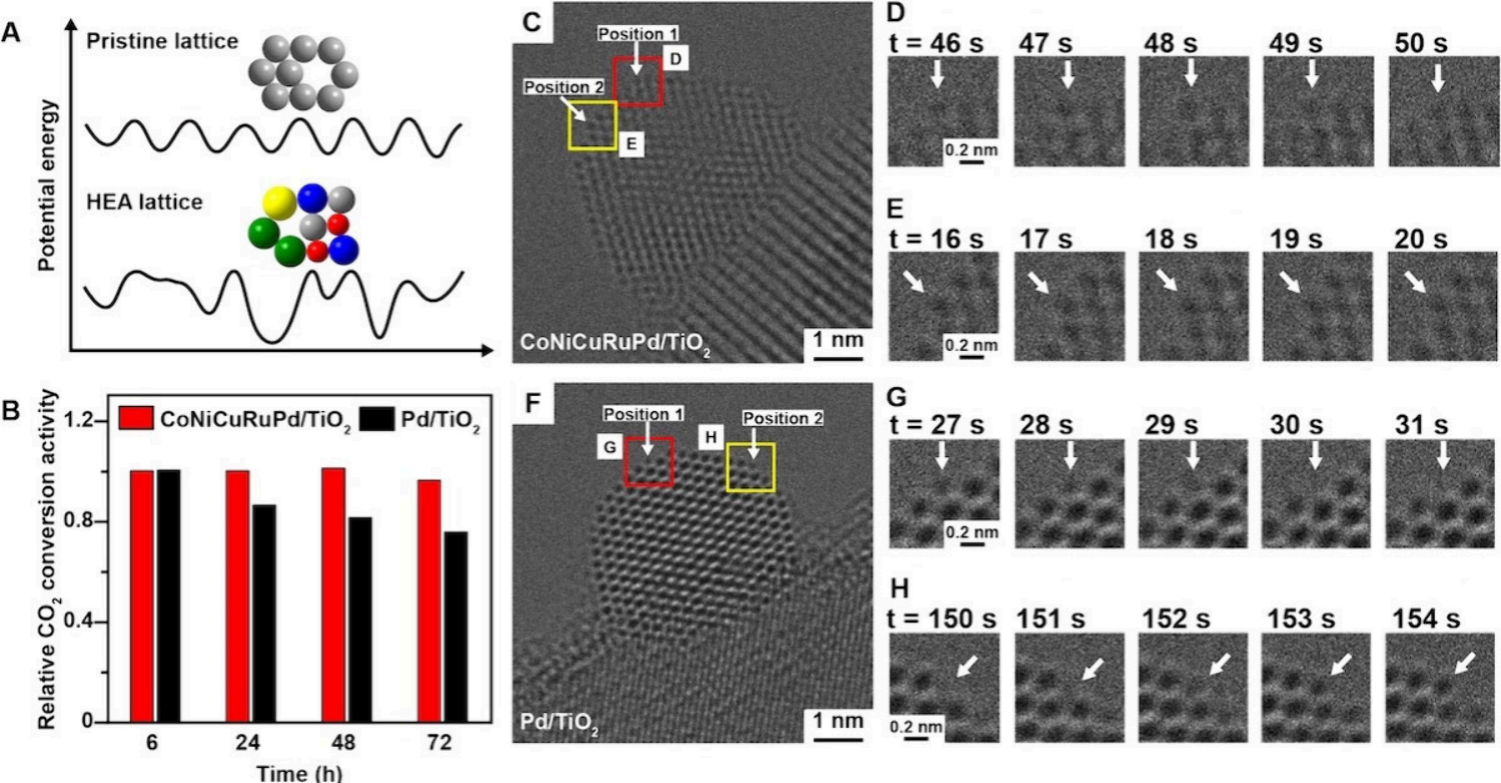
Origins and impacts of the sluggish diffusion effect. (A) Schematic illustration of potential energy fluctuations in pristine and HEA lattices, showing distributed migration barriers and deep traps that hinder atomic diffusion. (B) Long-term CO_2_ hydrogenation stability of CoNiCuRuPd/TiO_2_ and Pd/TiO_2_ catalysts, demonstrating the superior durability of the HEA system. (C) Representative TEM image of an individual nanocrystal in CoNiCuRuPd/TiO_2_. (D, E) Sequential *in situ* TEM frames showing surface-atom motion under electron-beam irradiation of CoNiCuRuPd/TiO_2_, where the atomic configuration remains stable due to intrinsically sluggish diffusion. (F) Representative TEM image of an individual nanocrystal in Pd/TiO_2_. (G, H) Sequential *in situ* TEM frames showing surface-atom motion under electron-beam irradiation of Pd/TiO_2_, exhibiting rapid atomic displacement and structural degradation. The images in (B–H) were reproduced with permission from ref 89. Copyright 2021 Springer Nature.

Such suppressed diffusivity at the atomic level has been directly correlated with the macroscopic catalytic durability of HEA. Mori and co-workers experimentally demonstrated this correlation by developing CoNiCuRuPd HEA nanocrystals supported on TiO_2_, which exhibited both high activity and exceptional stability during CO_2_ hydrogenation.^89^ The CoNiCuRuPd/TiO_2_ catalyst retained over 95% of its initial activity and constant selectivity after prolonged operation, in stark contrast to the severe deactivation observed for Pd-based counterparts (Figure 37B). This remarkable stability was further examined by *in situ* TEM. It was revealed that under intense electron-beam irradiation, conventional Pd/TiO_2_ rapidly underwent atomic displacement and structural collapse, whereas CoNiCuRuPd/TiO_2_ preserved a well-ordered atomic configuration even at edge and corner regions (Figure 37C–H). The persistence of atomic order under such extreme conditions highlights the intrinsically sluggish atomic diffusion in multicomponent HEA lattices, which effectively suppresses surface atom migration and structural reconstruction. Consequently, particle coarsening and phase transformation are largely inhibited, ensuring superior durability under both catalytic and environmental stress. This study provides direct atomic-level evidence to support the claim that the sluggish diffusion effect serves as a key kinetic origin underlying the exceptional structural and functional stability of HEA-based catalysts.

### 5.4 Cocktail Mixing Effect

The cocktail mixing effect refers to the nonlinear, emergent behavior exhibited by HEAs, whose properties cannot be simply predicted by the rule of mixtures.^244,245^ In a multicomponent system, the interplay among local chemical, structural, and electronic environments gives rise to nonadditive coupling, meaning that the overall behavior cannot be described as a simple sum or linear combination of the contributions from the individual elements, leading to overall behaviors that transcend the contributions from individual elements (Figure 38A). This emergent phenomenon is closely coupled with other key features of HEAs and underpins their cooperative catalytic behavior. Through nonadditive electronic and geometric interactions, the cocktail effect creates diverse active sites with tunable adsorption energies, enabling different reaction steps to proceed synergistically to enhance catalytic activity, selectivity, and durability.

**Figure 38:**
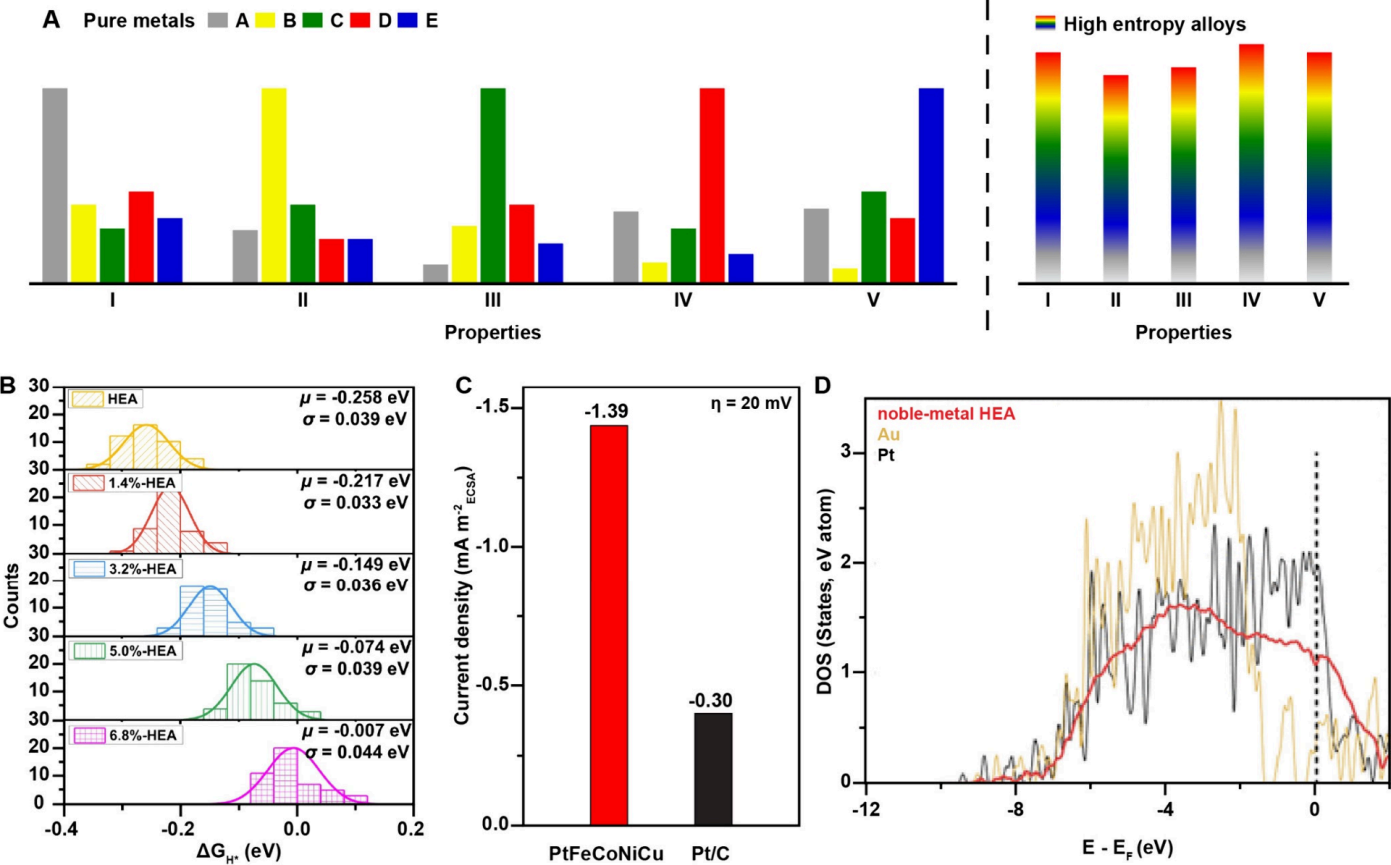
Origins and impacts of the cocktail mixing effect. (A) Schematic illustration of the effect, showing the nonlinear coupling among various physicochemical properties in HEA catalysts. (B) Gaussian distributions of the Δ*G*_H*_ on HEA surface, showing the corresponding expectation (μ) and standard deviation (σ) values that govern the unusual Sabatier principle. (C) Specific electrochemical activities of PtFeCoNiCu HEA and commercial Pt/C catalysts measured at an overpotential of 20 mV_RHE_, demonstrating the superior activity of the HEA catalyst. (D) DOS profiles calculated by DFT for noble-metal HEA, Au, and Pt nanocrystals, revealing broadened electronic states and enhanced orbital overlaps in HEA systems. The images in (B, C) were reproduced with permission from ref 33. Copyright 2024 Springer Nature. The image in (D) was reproduced with permission from ref 123. Copyright 2022 American Chemical Society.

Since the strength of these synergistic interactions is governed by the degree of chemical disorder and the statistical distribution of local environments, rational adjustment of elemental ratios can further optimize adsorption energetics and reaction barriers without altering the overall crystal phase. The classical Sabatier principle dictates that the optimal catalytic activity lies at the volcano-plot apex, where the adsorption free energy of intermediates approaches 0 eV.^59,246,247^ However, in monometallic or conventional alloy catalysts, the linear scaling relation between electronic structure and adsorption energy inherently limits catalytic activity to this theoretical peak. In contrast, HEAs, with their diverse local electronic distributions and heterogeneous coordination environments, can break this linear constraint. Chen and co-workers proposed an unusual Sabatier principle, wherein the hydrogen adsorption free energy on HEA surfaces follows a Gaussian distribution, described as *N*(μ, *σ* ^*2*^), where μ represents the expectation value and σ denotes the standard deviation, reflecting local heterogeneity (Figure 38B).^33^ Strong-binding sites favor H* adsorption, whereas weak-binding sites facilitate H_2_ formation. When μ approaches 0 eV and σ increases, the overall HER activity can surpass the traditional volcano-plot limit. Guided by this theory, the authors synthesized a PtFeCoNiCu HEA catalyst with compositional and electronic gradients, achieving a specific electrochemical activity of −1.39 mA m^–2^_ECSA_ at an overpotential of 20 mV_RHE_, and exhibiting an intrinsic activity more than four times higher than commercial Pt/C (Figure 38C). This clearly demonstrates the catalytic potential of multielement synergy in energy conversion.

To further elucidate the electronic origin of such nonadditive catalytic behavior, Wu and co-workers investigated the atomic-level electronic synergy in noble-metal HEA nanocatalysts.^123^ They revealed that HEA nanocrystals exhibit lower energy-level degeneracy and a more widely distributed local density of states (LDOS) relative to monometallic counterparts (Figure 38D). Atoms of the same element exhibit distinct LDOS profiles depending on their local coordination, whereas atoms of different elements may share similar electronic structures, indicating that atoms in HEAs effectively lose their elemental identity. This electronic delocalization and neighbor-dependent tunability enable the LDOS to be optimized through compositional design, thereby forming ideal electronic configurations for enhanced reactivity. The noble-metal HEA catalyst exhibited an HER activity of 10.8 times higher than that of commercial Pt/C, providing direct experimental and theoretical evidence to validate that the cocktail effect can be realized at the atomic scale through electronic complementarity and orbital coupling. Overall, the cocktail effect is a central synergistic mechanism in HEAs, enabling tunable adsorption energetics and reaction kinetics through nonadditive electronic and structural interactions. This effect highlights the nonlinear coupling among structure, electronic configuration, and catalytic activity, providing a useful framework for the rational design of advanced HEA catalysts.

Altogether, the four core effects, including high-entropy, lattice-distortion, sluggish-diffusion, and cocktail, endow HEAs with thermodynamic stability, structural adaptability, and tunable surface chemistry. Collectively, these intertwined factors not only underpin the intrinsic structural and electronic complexity of HEAs but also define their catalytic landscapes, governing activity, selectivity, and durability. This synergy bridges fundamental materials science with practical catalysis, establishing the mechanistic foundation for the performance analyses discussed in the following section.

## 6 Catalytic Applications

The remarkable catalytic performance of HEA nanocrystals arises from their four intrinsic effects, high configurational entropy, sluggish atomic diffusion, lattice distortion, and cocktail mixing, which collectively manifest at the surface.^23,25,29^ These thermodynamic and kinetic effects provide the basis for the exceptional activity, selectivity, and durability of HEA catalysts. Accordingly, HEA nanocrystals have shown broad promise in electro-, thermo-, and photocatalysis, partly because they can relax the linear scaling relations that limit conventional catalysts. The presence of multiple nonequivalent active sites with distinct electronic structures allows the adsorption energies of key intermediates (such as H*, OH*, CO*, OOH*, and N*) to vary independently. This decoupling enables alternative reaction pathways with lower overall energy barriers. As a result, high-entropy compositions often exhibit catalytic activities that exceed predictions based on simple scaling relations or the rule of mixtures, underscoring the unique potential of HEA nanocrystals as next-generation catalytic materials. Beyond compositional complexity, facet exposure and structural design also play critical roles in modulating surface atom coordination and optimizing catalytic behavior.^81,82,129^ Together, these features enable HEA nanocrystals to generate statistically favorable adsorption ensembles while maintaining structural integrity under operating conditions.

This section systematically explores the catalytic behaviors of HEA nanocrystals, emphasizing how their compositional complexity and surface structure, particularly the elemental makeup and exposed facets, influence reaction pathways and performance metrics. By analyzing representative reactions such as hydrogen and oxygen evolution and reduction, carbon dioxide and nitrogen reduction, and small-molecule oxidation, we highlight how the design principles of HEA nanocrystals can be tailored to meet specific catalytic challenges. Particular attention is paid to the unique ways by which HEAs modulate adsorption energetics, bypass conventional scaling relations, and stabilize active sites under practical reaction environments. This section aims to provide design guidelines for engineering next-generation HEA nanocatalysts for a wide spectrum of catalytic reactions.

### 6.1 Electrocatalysis

Electrocatalysis involves the acceleration of redox reactions at electrode–electrolyte interface via electron transfers between a catalytic surface and molecular reactants.^248^ These reactions typically proceed through multiple elementary steps involving adsorbed intermediates, and their rates are governed by the adsorption strength, activation energy, and stability of these species under operating conditions.^249^ Achieving efficient electrocatalysis requires catalysts that not only possess high intrinsic activity but also maintain stability in harsh electrochemical environments.^250^ HEA nanocrystals have emerged as a class of attractive electrocatalysts due to their compositional complexity and structural tunability.^22^ Unlike conventional binary or ternary alloys with uniform active sites, HEAs consist of multiple principal elements randomly distributed within a single lattice. This arrangement produces a continuum of chemically distinct surface configurations that enable broad tuning of adsorption energies for key reaction intermediates. In addition, local lattice distortion and electronic interactions among dissimilar atoms induce unique charge redistribution, allowing HEA surface to approach or even surpass the thermodynamic optima for both activity and selectivity. This subsection covers eight representative electrocatalytic reactions: HER, HOR, ORR, OER, CO_2_RR, NRR, NO_3_RR, and AOR.

#### 6.1.1 Hydrogen Evolution Reaction (HER)

HER is a half-reaction in water electrolysis, playing a central role in the sustainable production of hydrogen fuel.^251^ Developing efficient HER catalysts is essential for advancing hydrogen-based energy technologies across both acidic and alkaline systems. In acidic media, the HER typically follows two sequential steps: the Volmer step, in which protons (H^+^) are reduced to adsorbed hydrogen atoms (H*) on the catalyst surface, and either the Heyrovsky step, where H* reacts with another proton and an electron to form H_2_, or the Tafel step, where two adsorbed H* atoms recombine to release H_2_.^28^ The overall reaction is driven by the availability of protons and the ability of a catalyst to balance hydrogen adsorption and desorption. Optimal HER performance in acid media therefore hinges on the achievement of a near-thermoneutral hydrogen adsorption free energy (Δ*G*_H*_ ≈ 0), where neither adsorption nor desorption is rate-limiting.^252^

In alkaline media, the HER primarily proceeds via the Volmer–Heyrovsky or Volmer–Tafel pathways, both of which involve water dissociation and the adsorption/desorption of hydrogen intermediates on the catalyst surface. A key kinetic bottleneck arises from the energy-intensive cleavage of the H–OH bond during the Volmer step, making water activation a critical barrier that slows down the overall reaction rate.^253^ Recent theoretical studies further reveal that the adsorption free energy of hydroxyl species significantly influences HER kinetics.^254^ When OH* binds the catalyst surface too weakly, water dissociation becomes the rate-limiting step. On the other hand, overly strong OH* binding leads to sluggish desorption, hindering catalytic turnover. These findings converge in a three-dimensional volcano relationship, where the HER rate is jointly governed by the adsorption energies of both hydrogen and hydroxyl intermediates. Accordingly, achieving optimal alkaline HER activity requires catalysts that exhibit balanced binding strengths for both H* and OH*, strong enough to facilitate water activation, yet moderate enough to allow efficient desorption and release of hydrogen.

Owing to their compositional complexity, HEAs offer a unique platform for tuning the energetics. Their multielement surface is able to create a broad distribution of adsorption sites, statistically centering Δ*G*_H*_ and Δ*G*_OH*_ values around the Sabatier optimum rather than a single fixed value.^34,126,255^ Specifically, oxophilic constituents facilitate water activation, while adjacent metal sites promote H* adsorption and recombination, achieving cooperative functions rarely accessible in unary or binary catalysts. From a design standpoint, high configurational entropy ensures homogeneous dispersion of active elements within a stable solid-solution phase, maximizing atomic utilization and preventing segregation. Lattice distortion and charge redistribution further adjust the *d*-band center and hydrogen-binding strength, while sluggish atomic diffusion preserves structural integrity under potential bias. Altogether, these features impart outstanding activity and durability for HER in both acidic and alkaline environments.

By tailoring their composition, HEA nanocrystals can significantly enhance catalytic performance. For example, Feng and co-workers reported 2 nm PtRhFeCoNi HEA nanocrystals that exhibited outstanding HER activity in 0.5 M H_2_SO_4_, achieving a mass activity of 28.3 A mg^–1^ at −0.05 V_RHE_, approximately 40 times higher than that of commercial Pt/C.^19^ After the harsh test for 85 h, the PtRhFeCoNi nanocatalysts could maintain 100% of the initial current density. In comparison, Pt/C only maintained 74.8% of the initial current density. Operando XAS and theoretical analysis identified Rh and Pt as the primary active sites, while Fe, Co, and Ni contributed to electronic structure modulation and configurational entropy. These multimetallic interactions collectively optimize hydrogen-binding energetics and enable synergistic catalytic behavior, accounting for the exceptional activity and durability of the HEA nanocatalysts.

In addition to compositional tuning, facet engineering also plays a vital role in optimizing HER activity. In a recent study, Hsiao and co-workers synthesized facet-controlled Pd@Pd_0.2_Pt_0.2_Ir_0.2_Ru_0.2_Rh_0.2‑nL_ core–shell nanocrystals (where *n* is the number of HEA atomic overlayers) to expose {100} or {111} facets.^82^ Electrochemical measurements in 0.5 M H_2_SO_4_ revealed that the {100}-faceted Pd@Pd_0.2_Pt_0.2_Ir_0.2_Ru_0.2_Rh_0.2–4L_ core–shell nanocubes exhibited superior HER activity (Figure 39A), achieving the highest specific activity with a value of 267.8 A F^–1^ at −0.1 V_RHE_, which was 1.5 and 6.1 times higher than those of their {111}-faceted octahedral counterpart (179.4 A F^–1^) and commercial Pt/C (44.2 A F^–1^), respectively (Figure 39B). Notably, the core–shell nanocubes and octahedra also exhibited negligible loss in catalytic performance under acidic conditions, even after 15,000 cyclic voltammetry (CV) cycles, highlighting their exceptional electrochemical durability (Figure 39C). These results collectively validate that facet-controlled atomic configurations play a decisive role in governing HER kinetics under acidic conditions.

**Figure 39:**
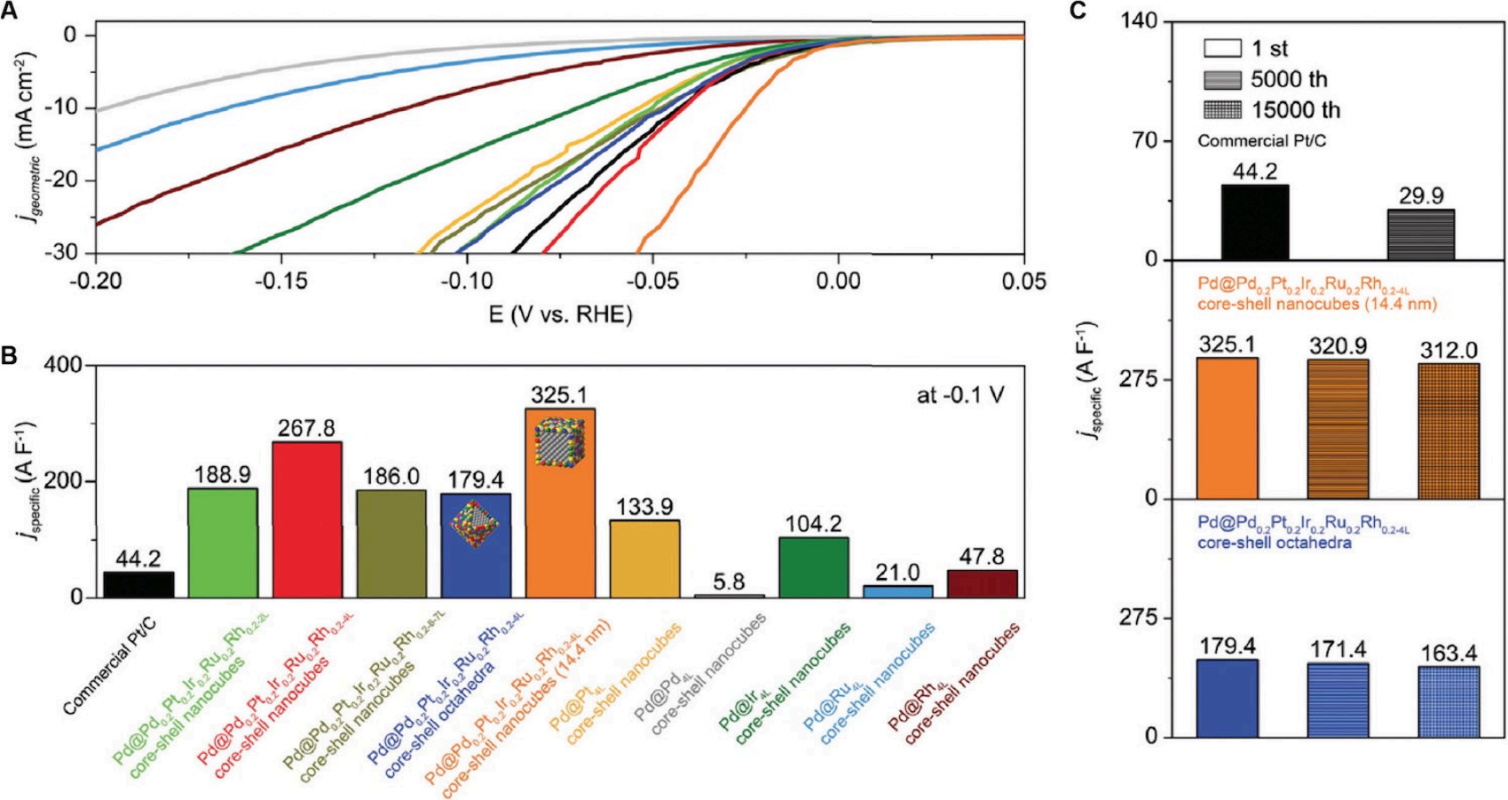
Electrocatalytic HER performance of Pd@HEA core–shell nanocrystals in 0.5 M H_2_SO_4_. (A) Polarization curves normalized to geometric areas of 0.07 cm^2^. (B) Comparison of specific (normalized to ECSA) activities at −0.1 V_RHE_, and (C) durability tests. Reproduced with permission from ref 82. Copyright 2025 Wiley-VCH.

Building upon this facet-dependent understanding, PdPtIrRuRh nanoframes featuring {110} facets were also synthesized, and they exhibited remarkable acidic HER activity.^133^ At an overpotential of −0.1 V_RHE_, their specific activity was nearly 10 times greater than that of commercial Pt/C, and they maintained excellent stability even after 15,000 potential cycling tests. When integrated into proton-exchange membrane water electrolyzer systems, these nanoframes also delivered strong cathodic performance. Computational analysis based on DFT indicated that the atomically mixed {110} facets effectively tuned the hydrogen adsorption free energy toward optimal values, thereby enhancing both reactivity and durability.

To further reduce the usage of PGMs, recent efforts have focused on alloying them with earth-abundant IGMs, which are neighboring elements to PGMs on the periodic table, to form single-phase HEA nanocrystals. This strategy not only reduces the contents of PGMs but also leverages the synergistic interactions among constituent elements to enhance catalytic performance. A series of nanocrystals with well-defined {100} facets was synthesized via a wet-chemical approach, in which quinary IGM–PGM atomic layers were epitaxially grown on Pd nanocubes.^81^ For example, Pd@PtRuFeCoNi core–shell nanocubes with a square atomic arrangement associated with the {100} facets. Among the 10 compositions tested, PtRuFeCoNi atomic overlayers exhibited superior catalytic activity (Figure 40A, B) and durability (Figure 40C, D) toward acidic HER compared to other HEA catalysts and commercial Pt/C. In 0.5 M H_2_SO_4_ electrolyte, the Pd@PtRuFeCoNi catalyst displayed the lowest HER overpotential of 41.3 mV_RHE_ at −10 mA cm^–2^. When normalized to electrochemical surface area (ECSA), its intrinsic activity reached 21.95 mA cm^–2^ at −0.1 V_RHE_, which was 4.5 times higher than that of commercial Pt/C (4.93 mA cm^–2^). After durability test over 15,000 potential cycles, the catalyst retained 92.7% of its initial activity, whereas Pt/C retained only 77.7%. Operando synchrotron XAS and DFT calculations further revealed that the enhanced performance arose from the multimetallic cocktail mixing. In particular, the Δ*G*_H*_ was computed for over 100 surface configurations per composition. The representative Pd@PtRuFeCoNi (Figure 40E) exhibited a median Δ*G*_H*_ of −0.01 eV, which was much closer to the thermoneutral ideal (0 eV) than those of other systems such as Pd@IrRuFeCoNi (−0.10 eV), consistent with the experimentally observed trend in activity (Figure 40F).

**Figure 40:**
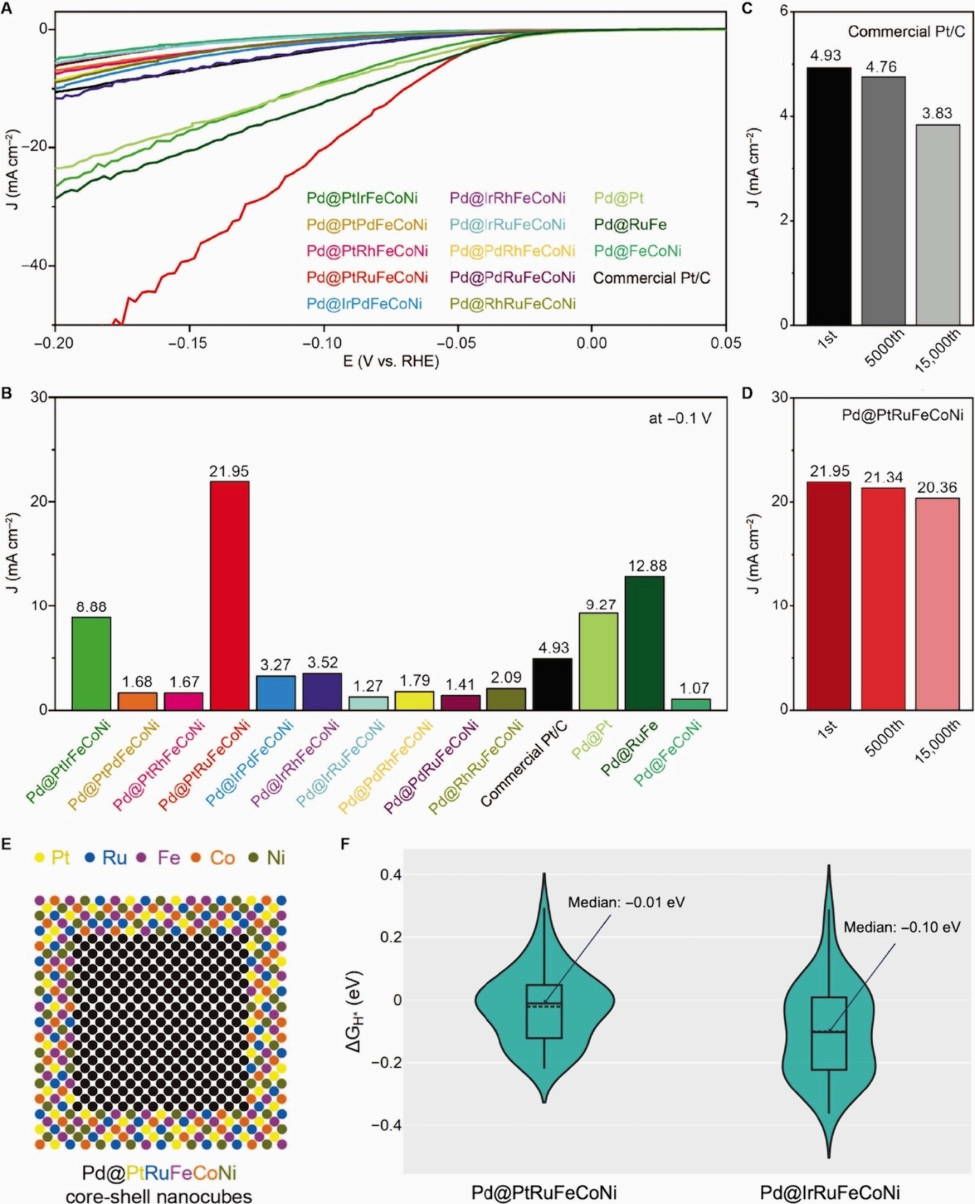
Catalytic performance and mechanism of IGM–PGM-HEA atomic overlayers in 0.5 M H_2_SO_4_. (A) Polarization curves, (B) comparison of intrinsic activities at −0.1 V_RHE_, and durability tests of (C) commercial Pt/C, and (D) Pd@PtRuFeCoNi core–shell nanocubes. (E) Schematic of epitaxial growth to obtain PtRuFeCoNi atomic overlayers on Pd nanocubes enclosed by {100} facets. (F) Hydrogen adsorption behaviors on Pd@PtRuFeCoNi and Pd@IrRuFeCoNi investigated using DFT. The distribution of hydrogen-adsorption free energy Δ*G*_H*_. Reproduced with permission from ref 81. Copyright 2024 AAAS.

To overcome the structural constraints of FCC system, unconventional HCP HEA atomic overlayers composed of five PGMs (Ru, Rh, Pd, Pt, and Ir) were synthesized via layer-by-layer epitaxial growth on HCP-Ru seeds.^129^ Despite the intrinsic thermodynamic preference of Rh, Pd, Pt, and Ir for FCC lattice, the synthetic strategy successfully created an HCP phase, as confirmed by synchrotron XAS, which revealed atomic-level mixing and altered coordination environments. The HCP Ru@Ru_0.2_Rh_0.2_Pd_0.2_Pt_0.2_tIr_0.2–4L_ core–shell nanocrystals exhibited significantly enhanced HER activity (Figure 41A, B) and durability (Figure 41C–F) in alkaline media compared to the FCC counterpart. The HER overpotential at −10 mA cm^–2^ was reduced to 50.8 mV_RHE_, and the specific activity reached 1.35 mA cm^–2^ at −0.1 V_RHE_, outperforming FCC analogs by 2.8-fold and commercial Pt/C by over 6-fold. Notably, durability tests over 15,000 cycles showed only a 9.1% activity loss, while FCC samples and commercial benchmarks suffered performance drops exceeding 25%. DFT calculations and operando XAS provided mechanistic insights into the structure–activity relationship (Figure 41G, H). Over 150 surface configurations were screened for hydrogen and hydroxide adsorption. The Pt sites on the HCP surface showed nearly ideal Δ*G*_H*_ (−0.0791 eV), enabling optimal H* adsorption consistent with the Sabatier principle. In contrast, Ir exhibited stronger binding. While less ideal for H*, it played a crucial role in OH* activation. For hydroxide adsorption, Ru and Ir sites showed strong oxophilicity. However, excessive OH binding to Ru hindered desorption, corroborated by its unresponsive operando XAS signals under applied bias. With a moderately strong Δ*G*_OH*_ (*ca.* 0.475 eV), Ir facilitated OH_ad_-to–OH^–^ conversion through effective electron donation, accelerating the Volmer step. These findings demonstrate that structural engineering not only stabilizes unconventional HCP phases but also tailors the surface atomic configuration and electronic structure to modulate key intermediates in HER. The synergistic combination of nonactive (Ru, Rh, Pd) and active (Pt, Ir) elements in a highly mixed HCP matrix offers a promising blueprint for next-generation multifunctional electrocatalysts (Figure 41I).

**Figure 41:**
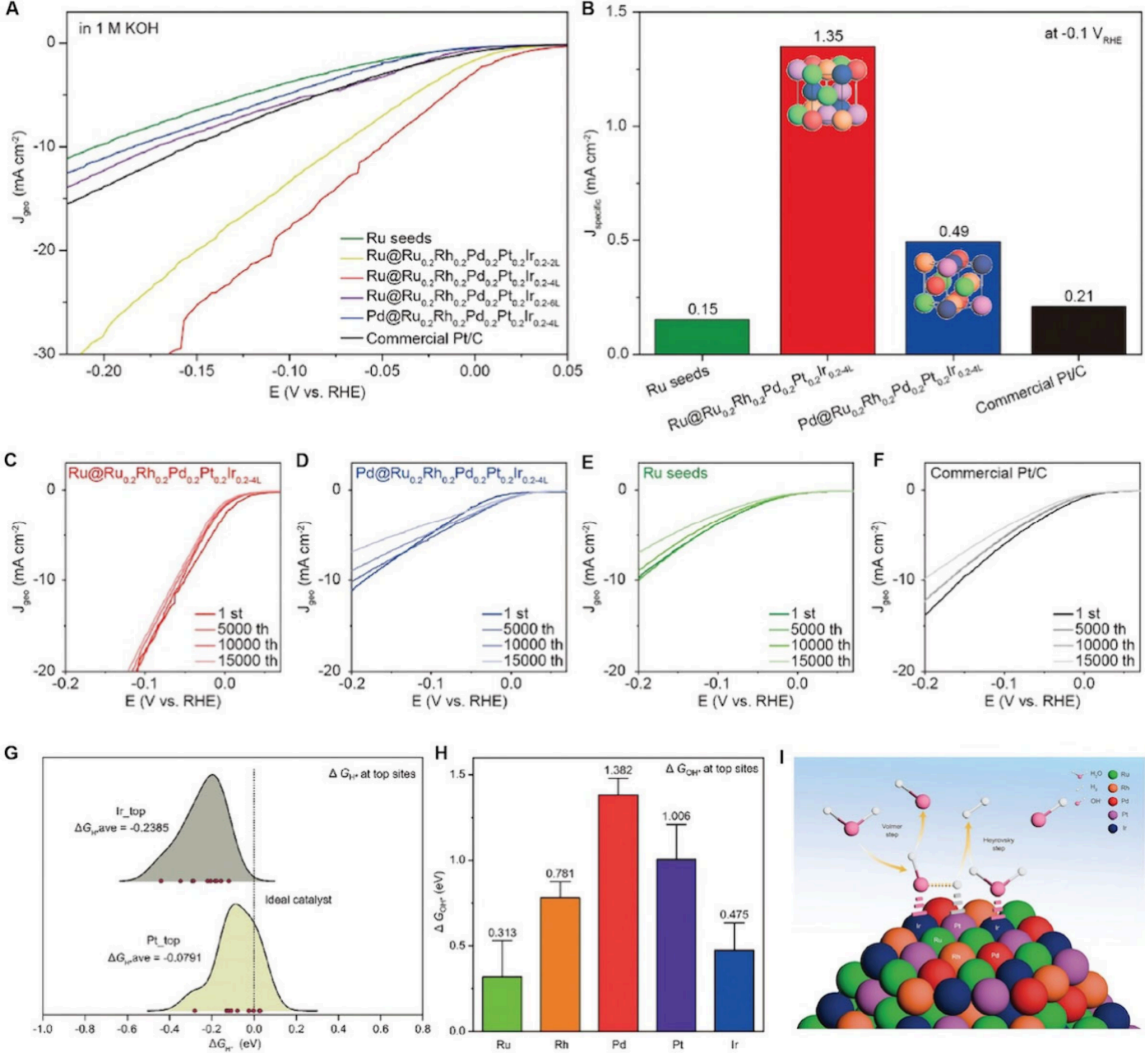
Electrocatalytic HER performance and mechanism of Ru@Ru_0.2_Rh_0.2_Pd_0.2_Pt_0.2_Ir_0.2‑nL_ core–shell nanocrystals in HCP and FCC phases, respectively. (A, B) HER in 1.0 m KOH solution: (A) Polarization curves and (B) specific activities at −0.1 V_RHE_. (C, D) Durability tests of (C) HCP Ru@Ru_0.2_Rh_0.2_Pd_0.2_Pt_0.2_Ir_0.2–4L_, (D) FCC Pd@Ru_0.2_Rh_0.2_Pd_0.2_Pt_0.2_Ir_0.2–4L_, (E) Ru seeds, and (F) commercial Pt/C in 1.0 m KOH solution. (G) Adsorption strength of H* at the top sites of Pt and Ir. (H) Adsorption strength of OH* at the top sites of elements on the HCP RuRhPdPtIr surface. (I) Schematic of the alkaline HER catalytic mechanism: Pt sites for H* adsorption while Ir sites facilitate water dissociation and the adsorption and desorption of OH*. Reproduced with permission from ref 129. Copyright 2024 Wiley-VCH.

In addition to the effects of composition, crystal facets, and structural ordering in HEA nanocrystals, recent studies also revealed an unconventional interpretation of the Sabatier principle for HER. Traditionally, optimal HER activity is achieved when Δ*G*_H*_ is close to 0 eV. However, HEA surfaces inherently feature a broad distribution of Δ*G*_H*_ values due to their atomic-level disorder and diverse local environments.^33^ This distribution can be modeled as a Gaussian function characterized by a mean (μ) and standard deviation (σ). When μ is near 0 eV and σ is large, the coexistence of both strong and weak H-binding sites enables efficient HER through a division of function: strong sites facilitate H* adsorption whereas weak sites support H_2_ desorption. Moreover, a low barrier (*ca.* 0.23 eV) to H* migration enhances spillover between sites, further improving kinetics. These insights suggest that optimizing the Δ*G*_H*_ distribution, rather than a single ideal value, can be a more effective strategy for HEA catalyst design. The intrinsic compositional complexity and lattice strain in HEAs naturally promote such energetic heterogeneity. When combined with tailored atomic structures, it unlocks new opportunities for achieving superior HER performance.

#### 6.1.2 Hydrogen Oxidation Reaction (HOR)

In hydrogen–oxygen fuel cells, HOR proceeds through the Volmer, Heyrovsky, and Tafel steps, with the overall kinetics governed by how the catalyst mediates H_2_ dissociation, proton–electron transfer, and *OH-assisted recombination.^256^ In alkaline media, sluggish HOR kinetics arise from an imbalance between hydrogen and hydroxide adsorption, motivating alloy-based strategies that promote coupling between bifunctional sites. Within this framework, two complementary descriptors are often invoked: the hydrogen-binding energy, which correlates rate with *H adsorption strength, and the bifunctional mechanism, which requires adjacent sites capable of handling *H and *OH cooperatively.^257^ Naturally, HEA nanocatalysts satisfy these design criteria. Their multicomponent surfaces generate distributed ensembles containing both hydrogen-binding and oxophilic sites in close proximity, allowing the Volmer and Heyrovsky steps to proceed concertedly. Electronic coupling among dissimilar metals tunes the *d*-band center and work function, aligning *H adsorption energies with the thermoneutral regime while simultaneously promoting *OH activation. Consequently, HEA nanocrystals can achieve high HOR activity in alkaline electrolytes with strong resistance to poisoning and surface degradation.

Recent progress has demonstrated the remarkable HOR activity of subnanometer HEA nanowires in alkaline media. In one study, PtRuNiCoFeMo HEA subnanowires supported on carbon (HEA SNWs/C) exhibited a mass activity of 6.75 A mg_Pt,Ru_^–1^ and a specific activity of 8.96 mA cm^–2^ at 0.05 V_RHE_, surpassing those of commercial Pt/C and PtRu/C by nearly 20- and 4-fold, respectively (Figure 42A–C).^20^ These catalysts also demonstrated strong tolerance against CO poisoning and excellent durability over 2,000 cycles of accelerated durability test (Figure 42D, E), highlighting their potential for practical applications in alkaline fuel cells. DFT calculations provided mechanistic insight into this enhanced performance. The hydrogen binding energies (HBE) (Figure 42F) and the oxhydryl binding energy (OHBE) (Figure 42G) on the HEA nanowire surface, evaluated across multiple adsorption sites including hollow and bridge geometries, were found to be optimized relative to Pt (111) and PtRu (111). Compared with the strong bindings of H* on Pt (111) and PtRu (111), the binding strength of H* on the HEA nanowires was significantly weakened to an appropriate level near the 0 eV (an ideal value for H* binding), leading to the improved HOR activity for the HEA nanowires. For OHBE, although the OH* binding strength displayed a trend of Pt < Co < Fe < Ni < Mo < Ru, the binding energy of OH* on Ru was almost −0.6 eV, suggesting that the moderate adsorption of OH* on the HEA nanowires. It has been well-known that the adsorbed OH* plays a vital role in removing the adsorbed protons in the form of H_2_O, and thereby significantly enhances the alkaline HOR activity. On the other hand, the overall reaction on HEA is exothermic with an energy release of 0.22 eV, indicating the rapid desorption of H_2_O. In sharp contrast, the overall reactions on Pt and PtRu were endothermic with energies of 0.57 and 0.27 eV, respectively (Figure 42H). Note that the energy barrier to H_2_O formation on HEA surface was only 0.08 eV, much lower than those on Pt and PtRu, further confirming the superior HOR activity of HEA to Pt and PtRu. These findings collectively highlight how compositional complexity and site heterogeneity in HEAs give rise to favorable adsorption energetics and reaction thermodynamics, enabling their remarkable activity in alkaline hydrogen oxidation.

**Figure 42:**
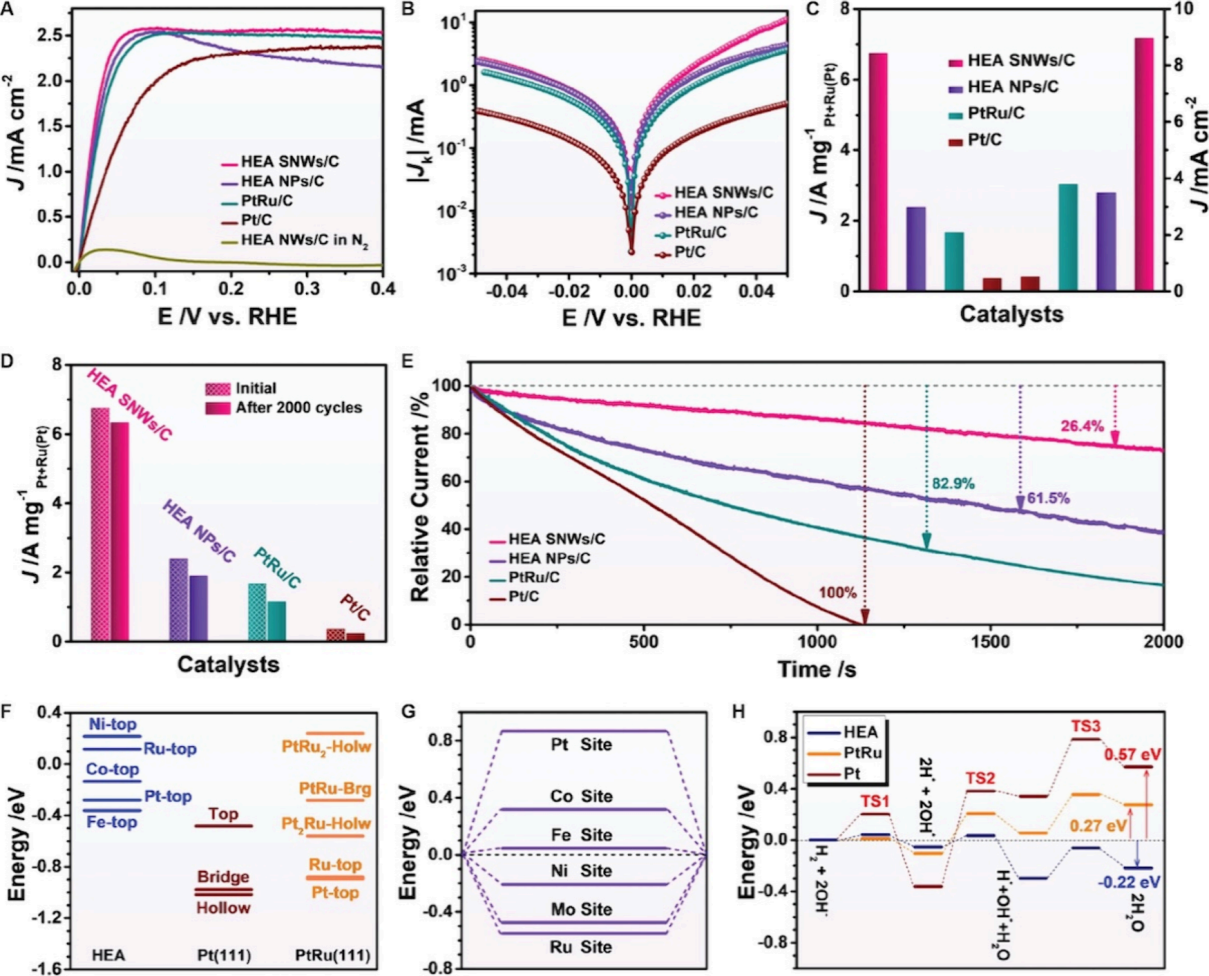
(A) Polarization curves in H_2_-saturated 0.1 M KOH. (B) Tafel plots. (C) Normalized mass activity and specific activity at an overpotential of 50 mV_RHE_. (D) Normalized mass activity and specific activity before and after 2000 cycles of ADTs. (E) Relative current–time chronoamperometry response of different catalysts in 1000 ppm of CO/H_2_-saturated 0.1 M KOH at 100 mV_RHE_. (F) The HBE comparison between HEA SNWs/C, Pt (111), and PtRu (111). Holw = Hollow sites. Brg = Bridge sites. (G) The OHBE on HEA SNWs/C. (H) The energetic trend of HOR on HEA SNWs/C, Pt (111), and PtRu (111). Reproduced with permission from ref 20. Copyright 2021 Springer Nature.

Again, controlling atomic configurations plays a critical role in optimizing catalytic performance, particularly for the HOR in alkaline media. A recent study proposed a compositional diversification strategy to enhance both the HOR activity and CO tolerance of unconventional FCC Ru-based catalysts.^134^ By synthesizing a library of 14 multicomponent FCC nanocrystals, the researchers systematically investigated how compositional variation influences catalytic behavior. The introduction of diverse elements was shown to induce synergistic electronic and geometric effects: the electronic tuning weakens the adsorption strength of key intermediates (*H, *OH, and CO), while compositional expansion optimizes atomic configurations, thereby altering the CO adsorption mode to reduce its binding strength at active sites. Among the synthesized compositions, the quinary RuInPtNiCu nanocrystals demonstrated outstanding HOR performance. The HEA catalyst exhibited markedly improved HOR performance relative to commercial Pt/C in H_2_-saturated 0.1 M KOH (Figure 43A, B). According to the Koutecky–Levich analysis, the HEA catalyst exhibited a kinetic current density of 5.36 A mg_PGM_^–1^, which was respectively 1.2, 1.9, 2.9, and 15.9 times higher than those of FCC-RuInPt (4.33 A mg_PGM_^–1^), FCC-RuIn (2.76 A mg_PGM_^–1^), FCC-Ru (1.88 A mg_PGM_^–1^), and commercial Pt/C (0.338 A mg_PGM_^–1^). Furthermore, CO tolerance tests revealed the resilience of the HEA under CO-containing environments. While FCC Ru catalysts suffered rapid activity loss upon exposure to 1,000 ppm of CO in H_2_, the RuInPtNiCu HEA retained its activity with no apparent change in polarization behavior, highlighting its superior resistance to CO poisoning (Figure 43C). To elucidate the origin of the enhanced HOR activity, DFT calculations were conducted on the FCC RuInPtNiCu HEA model. The schematic in Figure 43D illustrates a bifunctional Volmer–Heyrovsky mechanism, where *H and *OH species adsorb at adjacent sites, enabling concerted hydrogen dissociation and water formation. As shown in Figure 43E, the Δ*G*_H*_ on the HEA surface lies near the thermoneutral value (ca. 0 eV), indicating moderated *H binding that promotes efficient adsorption–desorption dynamics. The overall free energy profile implies reduced reaction barriers and exergonic steps throughout the HOR pathway, in contrast to monometallic or binary catalysts (Figure 43F). This energetically favorable landscape, coupled with the synergistic *H and *OH interactions among multiple metal sites, rationalizes the high activity, low overpotential, and excellent durability of the FCC RuInPtNiCu HEA observed experimentally.

**Figure 43:**
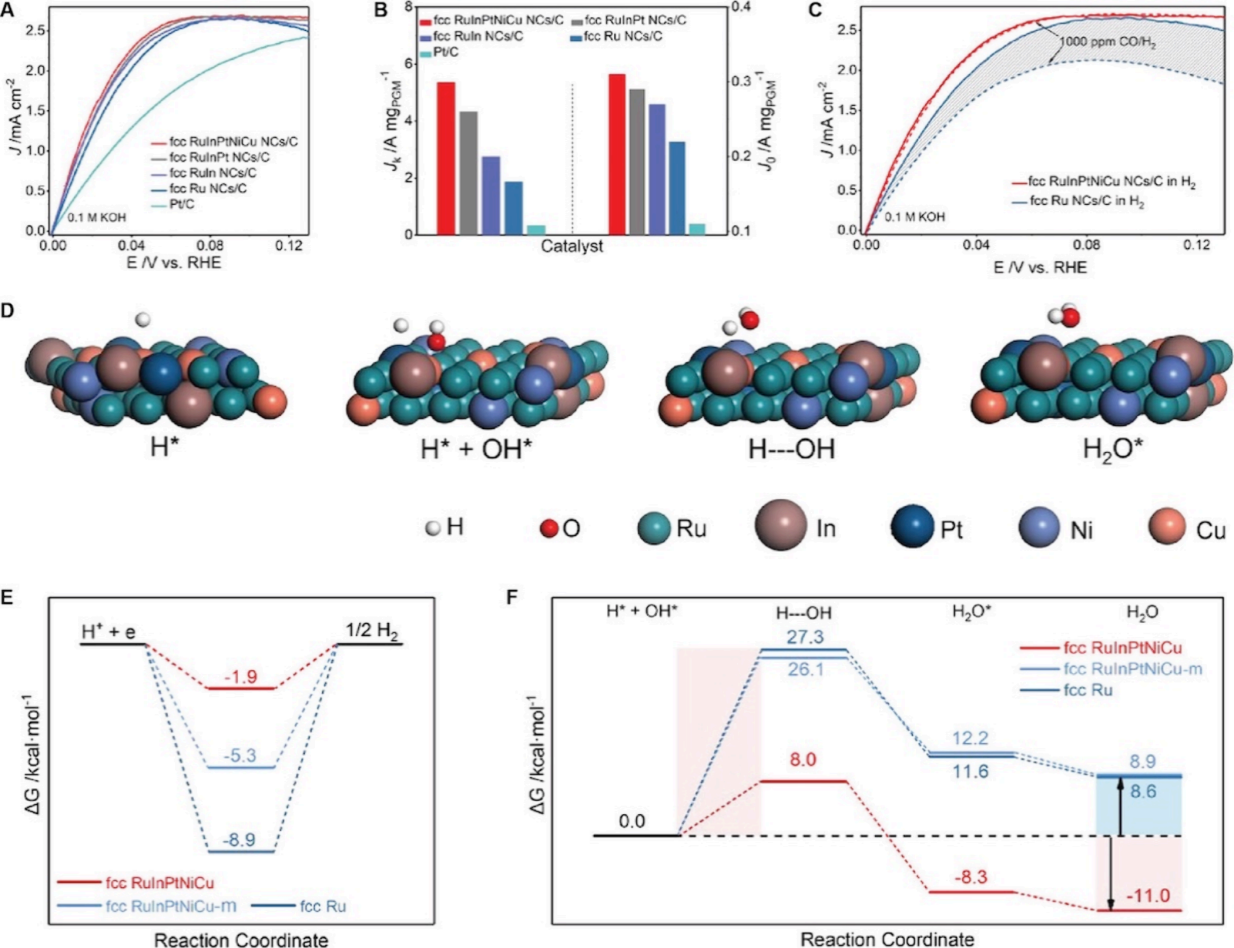
Alkaline HOR performance and CO tolerance of FCC Ru-based catalysts. (A) HOR polarization curves of FCC Ru-based catalysts and commercial Pt/C in H_2_-saturated 0.1 M KOH. (B) Comparison of the *J*_*k*_ at 50 mV_RHE_ and *J*_*0*_ of FCC RuInPtNiCu NCs/C with other catalysts. (C) Comparison of HOR polarization curves of FCC RuInPtNiCu NCs/C and FCC Ru NCs/C in pure H_2_ and 1000 ppm of CO/H_2_. (D) Schematic illustrations of the HOR mechanism on FCC RuInPtNiCu models. (E) Free energy diagrams of H* adsorption, (F) free energy diagrams for reaction pathways. Reproduced with permission from ref 134. Copyright 2025 American Chemical Society.

Comparable strategies involving the control of surface atomic configurations have also been shown to enhance HOR performance. For instance, Wu and co-workers demonstrated that Pd@PtRuFeCoNi core–shell nanocubes with PtRuFeCoNi surface layers exposing square atomic arrangements on {100} facets achieved an intrinsic activity of 0.82 mA cm^–2^ at 0.1 V_RHE_, 5.5 times higher than commercial Pt/C, and maintained 84% of the activity after 3000 cycles.^81^ These findings collectively underscore that metastable multimetallic atomic layers with tailored local coordination environments can serve as highly active and durable catalysts for alkaline HOR.

#### 6.1.3 Oxygen Reduction Reaction (ORR)

ORR is widely recognized as one of the most kinetically sluggish electrochemical processes, primarily due to its multistep nature involving the transfer of multiple electrons and protons and the sequential transformation of O_2_ into adsorbed *OOH, *O, and *OH intermediates.^258^ Depending on the catalytic surface and operating environment, the ORR can proceed through either a four-electron pathway to produce water or a two-electron pathway to generate hydrogen peroxide. In line with the Sabatier principle, an effective ORR catalyst must strike a balance in binding strength, strong enough to facilitate O–O bond activation, yet sufficiently weak to allow the efficient desorption of reaction products.^246,259^ HEA nanocrystals offer a promising platform for tuning adsorption energetics relevant to oxygen electrocatalysis. Owing to their multicomponent nature, HEA surfaces present a broad distribution of adsorption sites, which can be adjusted through compositional variation to modulate the binding strengths of *O, *OH, and *OOH intermediates. The diversity of sites may allow HEAs to approach the optimal oxygen-binding regime observed in the Pt-based system, while potentially offering improved durability. Moreover, their high configurational entropy can help stabilize single-phase solid solutions, while lattice distortions and reduced atomic mobility may contribute to enhanced structural stability and resistance to degradation during extended cathodic operation.

For example, Wang and co-workers recently reported a catalyst based on PtFeCoNiMn HEAs on ordered mesoporous carbon (OMC) support.^115^ As shown in Figure 44A, B, this catalyst outperformed Pt/C in half-wave potential (0.88 vs 0.86 V_RHE_), mass activity (1.12 vs 0.30 A mg_Pt_^–1^), and specific activity (2.84 vs 0.44 mA cm_Pt_^–2^). The enhancement arose from the intrinsically higher catalytic activity of Pt when incorporated into the PtFeCoNiMn nanocrystals relative to that in conventional Pt/C. Both density of states and *d*-band analyses revealed that the incorporation of multiple elements in HEA structures significantly altered the electronic characteristics of Pt relative to its monometallic form (Figure 44C–F). Specifically, Pt atoms in the HEA exhibited a broader and more heterogeneous 5*d* density of states profile due to diverse local atomic configurations. This electronic heterogeneity reduced energy level degeneracy, enabling more flexible interactions with various ORR intermediates and facilitating optimized binding across available Pt sites. Additionally, the electronic coupling between Pt and non-noble metals in the HEA induced a downward shift of the *d*-band center by approximately 0.2 eV, which weakened Pt–O adsorption and enhanced intermediate desorption. Free energy calculations indicated that the rate-determining step shifted from *OH formation on monometallic Pt (Δ*G* ≈ 0.3 eV) to OOH/OH formation on HEA (Δ*G* ≈ 0.7 eV), consistent with the enhanced kinetics expected in an exothermic ORR pathway. Moreover, analysis of surface atoms showed that the *d*-band center of Pt in HEA spanned a wider range from −2.3 to −3.4 eV than in monometallic Pt (−2.4 to −2.6 eV), further supporting improved activity toward the intermediates. Notably, some Fe, Co, Ni, and Mn atoms in HEA acquired less positive *d*-band centers compared to their monometallic counterparts, suggesting that inert metals may also become catalytically active via multimetallic electronic interactions.

**Figure 44:**
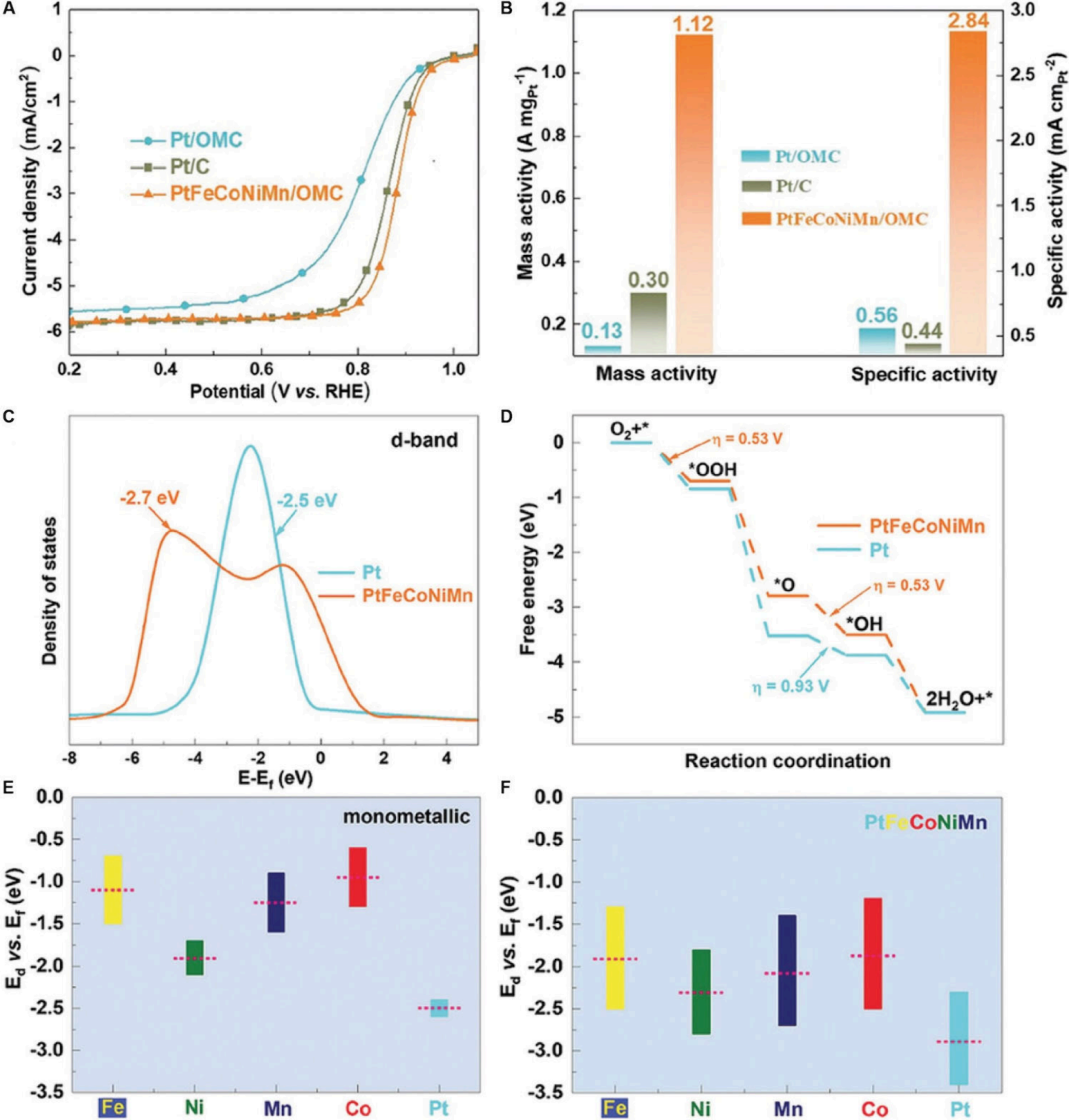
Characterizations of ORR activity. (A) Linear sweep voltammetry (LSV) in O_2_-saturated 0.1 M HClO_4_ solution. (B) Mass and specific activities of Pt/OMC, Pt/C, and PtFeCoNiMn/OMC. (C) The projected electronic density of states for Pt atoms and (D) free-energy diagrams of monometallic Pt and PtFeCoNiMn HEA. (E, F) The *d*-band center values of surface atoms in monometallic and PtFeCoNiMn HEA. The pink dotted lines are the average surface *d*-band center values of each element, respectively. Reproduced with permission from ref 115. Copyright 2024 Wiley-VCH.

In addition to HEA nanocrystals featuring disordered atomic solid-solution structures, recent studies have also explored ordered HEI nanocrystals as promising electrocatalysts toward the ORR. These materials combine the advantages of multielement synergy with well-defined atomic arrangements to enable multiple active sites and enhanced stability. A notable example involved the synthesis of ultrasmall (ca. 2 nm) PtFeCoNiCuZn HEI nanocrystals using a space-confinement strategy to precisely regulate the ordering at the atomic scale.^260^ This approach enabled the formation of a highly ordered structure despite the complexity of incorporating six elements. The resulting PtFeCoNiCuZn nanocrystals exhibited a remarkable ORR mass activity of 2.4 A mg_Pt_^–1^ at 0.90 V_RHE_, which was approximately 19 times higher than that of commercial Pt/C. When integrated into a proton exchange membrane fuel cell at a low Pt loading of 0.03 mg_Pt_ cm^–2^, the catalyst delivered an impressive power density of 1.4 W cm^–2^ and a rated power of 45 W mg_Pt_^–1^, demonstrating its exceptional mass-specific performance and practical applicability. This work highlights the potential of HEI nanocrystals in overcoming the intrinsic limitations of conventional Pt-based catalysts through multimetallic synergy, paving the way toward high-performance and resource-efficient energy conversion technologies.

#### 6.1.4 Oxygen Evolution Reaction (OER)

OER dictates the efficiency of overall water splitting and remains among the most kinetically sluggish electrochemical processes.^261–263^ It proceeds through four coupled proton–electron transfers involving *OH, *O, and *OOH intermediates, following either the adsorbate-evolution or lattice-oxygen-mediated mechanism. HEA nanocrystals provide a versatile platform to circumvent these constraints. Their multicomponent lattices generate heterogeneous coordination and electronegativity environments that enable *OH and *OOH adsorption on distinct atomic sites, thereby decoupling their energetics and mitigating scaling relations. The combination of metals with different oxophilicity stabilizes disordered solid-solution frameworks capable of operating under both acidic and alkaline conditions.

Recently, a rapid and facile approach was developed to synthesize a quinary high-entropy Ru-based alloy (RuMnFeMoCo) supported on carbon felt (CF) toward OER under acidic conditions.^44^ The OER activity was assessed in 0.5 M H_2_SO_4_ using a three-electrode configuration. The RuMnFeMoCo/CF catalyst showed greatly enhanced OER activity compared to its counterparts. As shown in Figure 45A, RuMnFeMoCo/CF achieved current densities of 10 and 100 mA cm^–2^ at low overpotentials of 170 mV_RHE_ and 225 mV_RHE_, respectively, substantially outperforming RuMnFeMo/CF (184 and 259 mV_RHE_), Ru/CF (238 and 334 mV_RHE_), and commercial RuO_2_ (291 and 414 mV_RHE_). The corresponding Tafel slopes further confirmed the improved kinetics (Figure 45B), with RuMnFeMoCo/CF exhibiting the lowest value of 49.7 mV dec^–1^, in contrast to 57.2 mV dec^–1^ for RuMnFeMo/CF and 83.4 mV dec^–1^ for commercial RuO_2_. The electrochemical surface area estimated via the double-layer capacitance reached 98.1 mF cm^–2^ for RuMnFeMoCo/CF, about 2.5 times higher than that of Ru/CF, suggesting a greater number of accessible active sites (Figure 45C). Long-term chronopotentiometric tests revealed superior durability, as RuMnFeMoCo/CF remained stable operation at 10 and 100 mA cm^–2^ for over 1,000 and 150 h, respectively, with negligible potential increase, in stark contrast to the rapid degradation observed for RuO_2_ (Figure 45D, E). Their DFT calculations indicated that the incorporation of corrosion-resistant elements such as Ru, Mo, and Co enhanced the catalyst’s thermodynamic stability in acidic media. Meanwhile, the inclusion of Mn, Fe, and Co effectively lowered the activation energy of the rate-determining step, facilitating faster OER kinetics and reducing the required overpotential. These results collectively highlight the role of multimetal synergy and high-entropy-induced site heterogeneity in promoting both activity and durability under acidic conditions.

**Figure 45:**
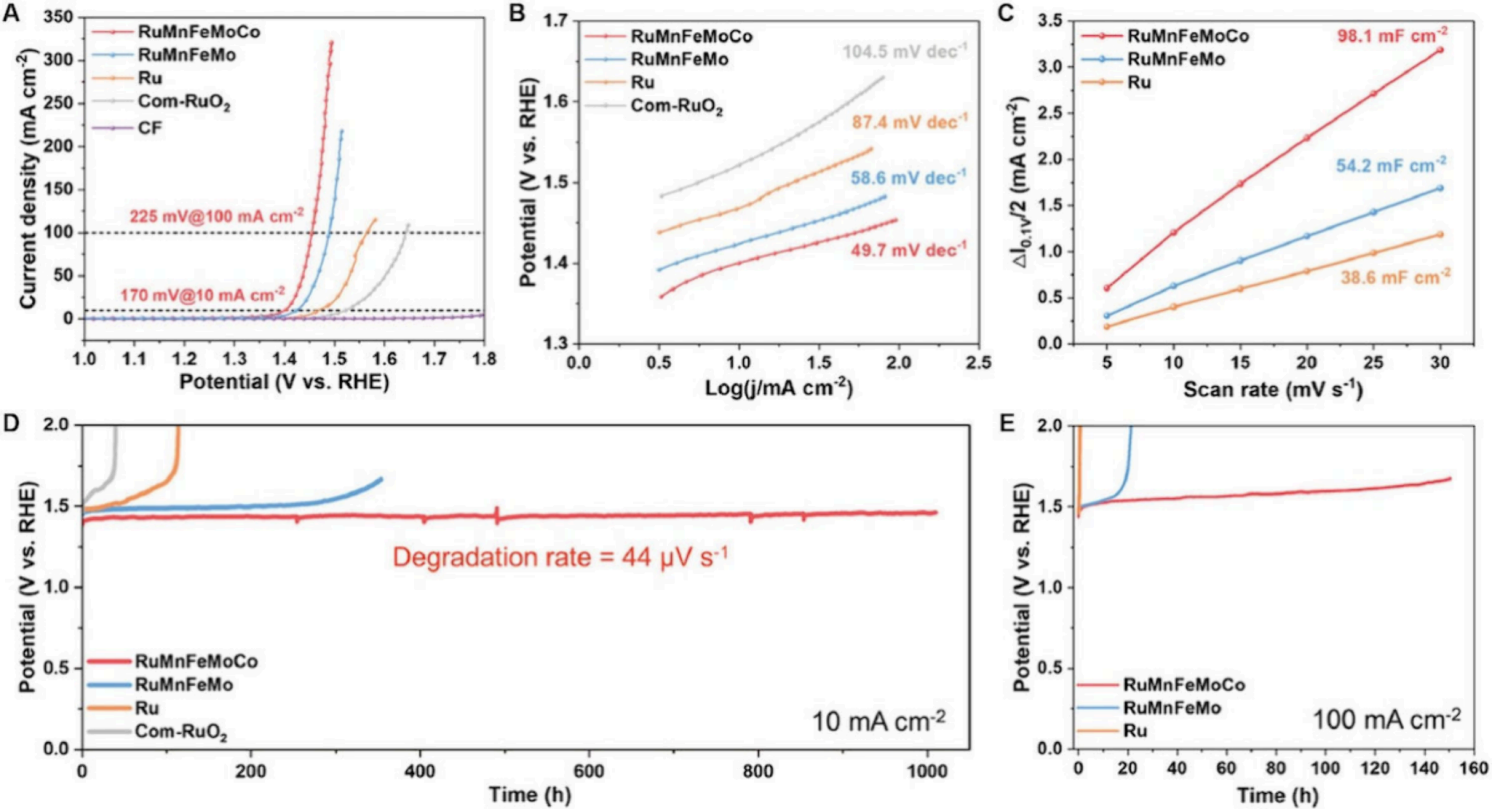
OER performance of RuMnFeMoCo/CF in three-electrode system and PEMWE. (A) OER polarization curves of RuMnFeMoCo/CF, RuMnFeMo/CF, Ru/CF, commercial RuO_2_/CF, and pure CF substrate. (B) Tafel plots of RuMnFeMoCo@CF, RuMnFeMo@CF, Ru@CF, and commercial RuO_2_@CF. (C) Capacitive current at 0.9 V_RHE_ against the scan rate and the corresponding double-layer capacitance values estimated through linear fitting of the plots. (D) Chronopotentiometry test of RuMnFeMoCo@CF, RuMnFeMo@CF, Ru@CF, and commercial RuO_2_@CF at 10 mA cm^–2^ and (E) 100 mA cm^–2^. Reproduced with permission from ref 44. Copyright 2025 Wiley-VCH.

Understanding the structural evolution of HEA nanocrystals under acidic OER conditions is a critical issue in catalyst design. A recent study systematically investigated this phenomenon using IrFeCoNiCu nanocrystals, which demonstrated superior OER activity and stability compared to monometallic Ir and even Ir-based bimetallic alloys.^264^ TEM and EDS analyses revealed that under acidic OER conditions, the carbon encapsulation layers delaminated and the surface 3*d* transition metals (Fe, Co, Ni, and Cu) gradually dissolved (Figure 46A). This process resulted in the formation of an Ir-enriched shell, while the core retained its original homogeneous single-phase HEA structure (Figure 46B). These findings highlight that despite their intrinsic thermodynamic stability, HEA nanocrystals remain vulnerable to surface reconstruction in harsh oxidative and acidic environments. The configurational entropy near the surface is insufficient to fully counteract electrochemical redox-induced degradation. Careful selection of corrosion-resistant and electrochemically stable elements is essential when designing HEA compositions for long-term acidic OER applications.

**Figure 46:**
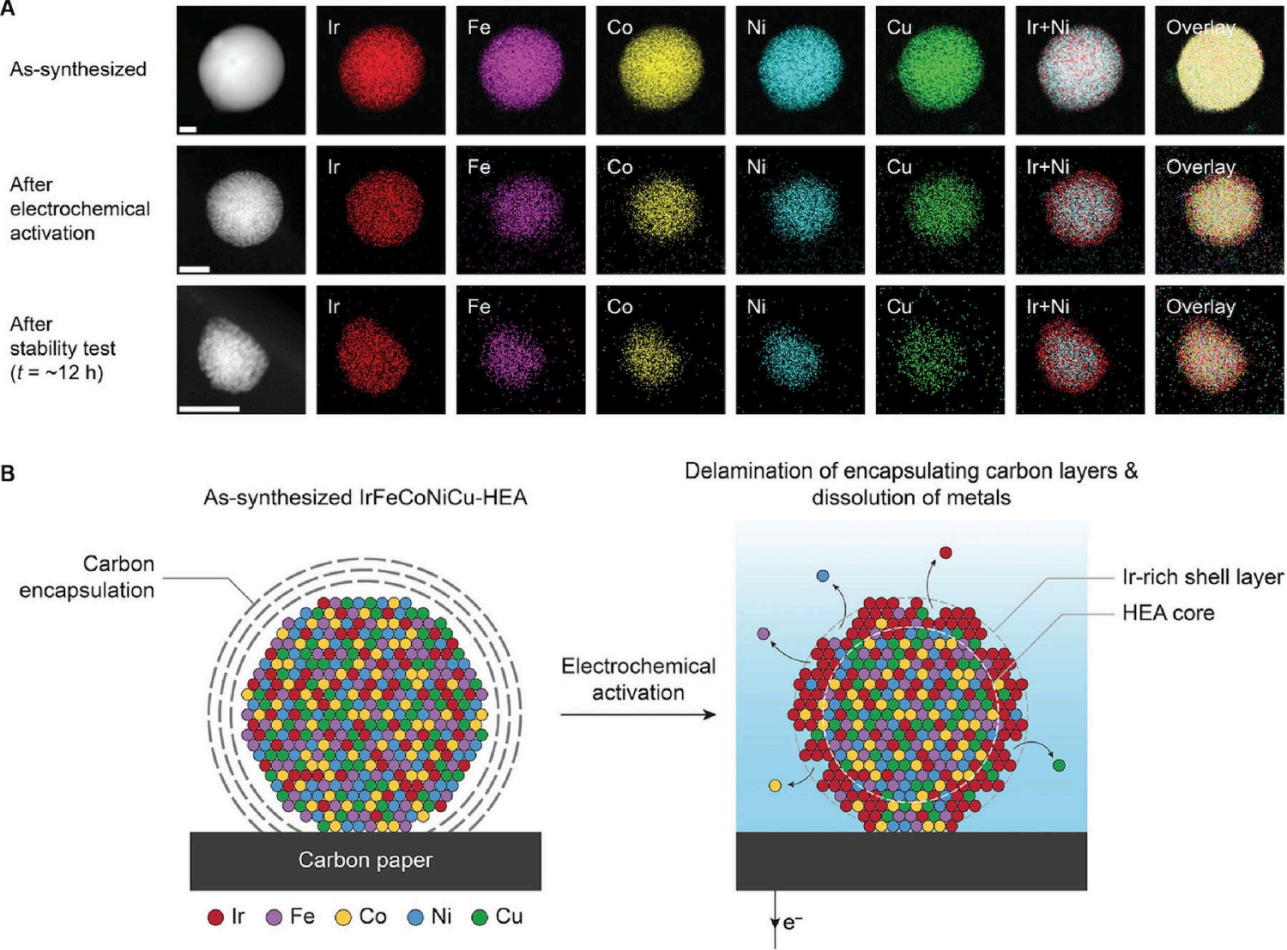
(A) STEM–EDS maps showing the elemental distribution of IrFeCoNiCu-HEA at different time points of the OER stability test (all scale bars: 20 nm). (B) Illustration of the structural evolution of IrFeCoNiCu-HEA nanoparticle under acidic OER conditions. Reproduced with permission from ref 264. Copyright 2023 American Chemical Society.

#### 6.1.5 Carbon Dioxide Reduction Reaction (CO2RR)

CO_2_RR represents a promising route toward sustainable carbon-neutral fuel and chemical production, yet it remains kinetically complex and limited by product selectivity.^72,265^ The reaction proceeds through multiple proton–electron transfer steps that generate diverse products, ranging from CO to hydrocarbons and oxygenates, while competing with the HER. Achieving high activity and selectivity requires precise regulation of the adsorption and conversion energetics of key intermediates (*CO_2_, *COOH, *CO, *CHO, *OCCO, *HCOO), which govern reaction pathways and determine product distributions.^266,267^ HEA nanocrystals offer a versatile platform to address these challenges. Their multicomponent surfaces provide a continuum of nonequivalent active sites with broad distributions of adsorption energies, allowing selective stabilization of reaction intermediates. Subtle compositional tuning can shift site populations responsible for *CO formation, C–C coupling, or hydrogenation, enabling cooperative control over both activity and selectivity within a single catalyst. In addition, the inherent structural stability of entropy-stabilized lattices supports long-term operation under electrochemical conditions. Computational and experimental studies have substantiated these advantages.^47,268^ DFT analyses reveal that compositional modulation not only alters the average binding strengths of intermediates but also reshapes the overall energy landscape, thereby steering selectivity toward desired products. Correspondingly, noble-metal HEAs have been shown to optimize *CO and *HCOO binding to favor CO or formic acid formation while suppressing HER, whereas non-noble systems exploit synergistic 3d–4d interactions to balance *CO and *H adsorption for cost-effective CO_2_ conversion.

Nellaiappan and co-workers synthesized AuAgPtPdCu HEA nanocrystals that exhibited markedly improved selectivity compared with monometallic Cu.^47^ The catalyst achieved nearly 100% FE at −0.3 V_RHE_ (Figure 47A, B), producing CO, CH_4_, C_2_H_4_, and H_2_ with a distinct product distribution from that of pure Cu (Figure 47C), highlighting the synergistic role of multimetallic active sites. Li and co-workers prepared PdCuAuAgBiIn HEA aerogels via a freeze–thaw method, demonstrating high activity and durability for CO_2_RR (Figure 47D–F).^48^ The catalyst achieved nearly 100% FE for C_1_ products over −0.7 to −1.1 V_RHE_, with formic acid as the dominant product and a maximum FE_HCOOH_ of 98.1% at −1.1 V_RHE_ (Figure 47D). The corresponding partial current density reached 8.6 mA cm^–2^ at −1.0 V_RHE_ (Figure 47E). Valence-band photoemission spectra revealed a positive d-band center shift relative to reference catalysts, enhancing interaction with adsorbates (Figure 47F). The strong metal–metal coupling and surface unsaturated sites in PdCuAuAgBiIn HEA aerogels optimized HCOO* adsorption and desorption, thus boosting formic acid formation. Despite these advances, the field of HEA-based CO_2_ reduction catalysis remains in its early stages, and further systematic exploration is needed to fully realize its potential.

**Figure 47:**
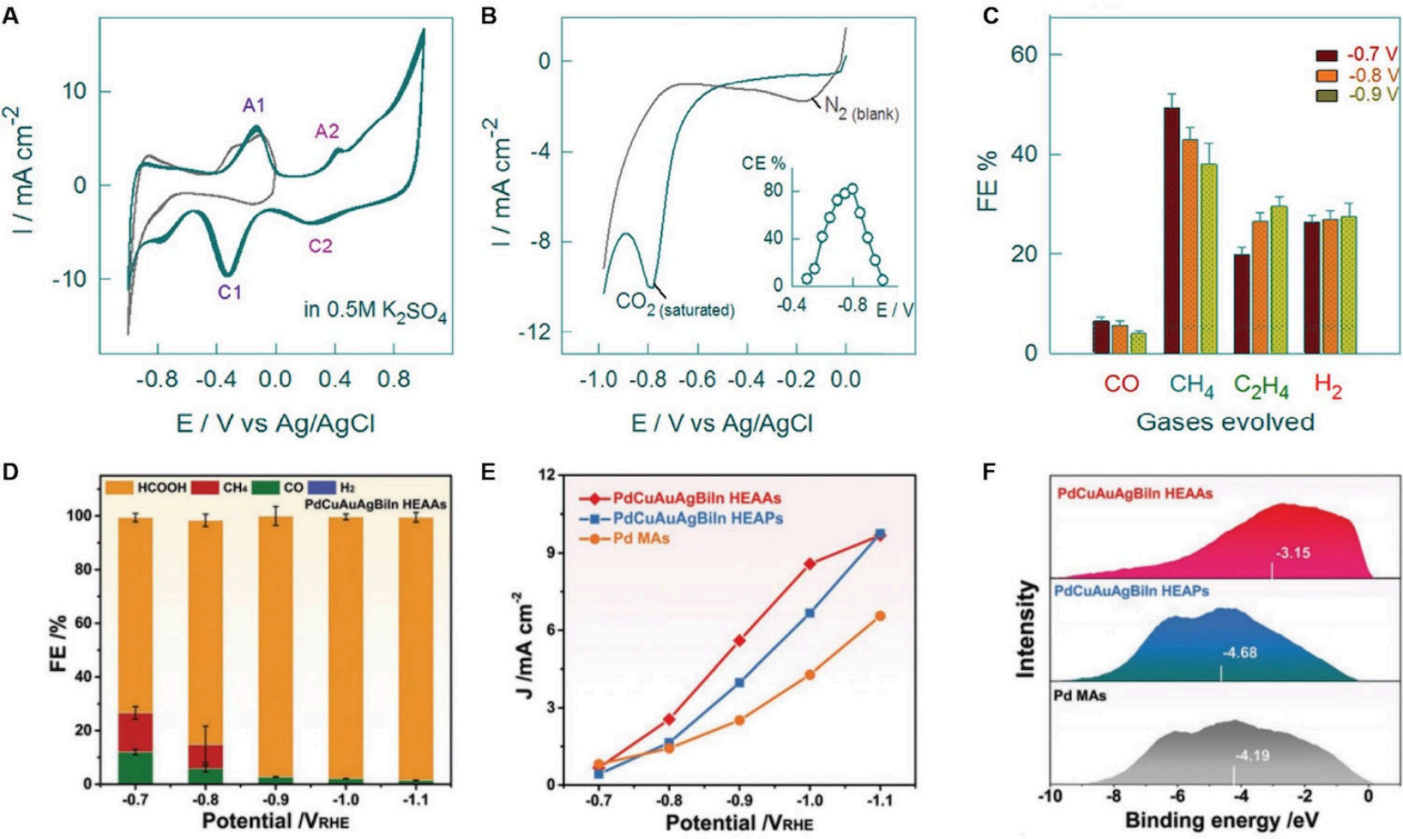
Representative examples of HEA catalysts for the electrochemical CO_2_RR. (A-C) AuAgPtPdCu HEA nanocrystals (A) CV responses recorded in blank 0.5 M K_2_SO_4_ at a scan rate of 40 mV s^–1^. (B) LSV curves for CO_2_ reduction after 30 min CO_2_ saturation at a scan rate of 20 mV s^–1^ in comparison with N_2_-purged electrolyte; inset shows current efficiency (CE%) vs applied potential. (C) Bar plots of Fes for carbonaceous and hydrogen products. (D–F) PdCuAuAgBiIn HEA aerogels. (D) Faradaic efficiency (FE) of products under different reduction potentials on PdCuAuAgBiIn HEA aerogels. (E) Partial current densities of HCOOH at −0.9 V_RHE_ for PdCuAuAgBiIn HEA aerogels, PdCuAuAgBiIn HEA particles, and Pd monometallic catalysts (MAs). (F) d-band center positions of PdCuAuAgBiIn HEA aerogels, HEA particles, and Pd MAs determined from surface valence-band photoemission spectra. The images in (A–C) were reproduced with permission from ref 47. Copyright 2020 American Chemical Society. The images in (D–F) were reproduced with permission from ref 48. Copyright 2022 Wiley-VCH.

#### 6.1.6 Nitrogen Reduction Reaction (NRR)

Electrochemical nitrogen reduction to ammonia presents a sustainable alternative to the energy-intensive Haber–Bosch process.^269^ However, activating the thermodynamically stable N≡N bond under ambient conditions remains a major challenge.^270^ On heterogeneous catalysts, the reaction typically proceeds via N_2_ adsorption, stepwise hydrogenation, and NH_3_ desorption, following either a dissociative or associative pathway. At low temperatures, the associative route, where the N–N bond is retained through early intermediates, is generally more favorable, yet it requires catalysts capable of activating N_2_ while simultaneously suppressing the competing HER.^271^

Designing an effective NRR catalyst thus involves balancing three key requirements: adequate N_2_ binding strength for activation, moderate hydrogen affinity to enable controlled hydrogenation, and facile NH_3_ desorption to maintain active site turnover.^272,273^ Such a balance is difficult to achieve in unary systems. For example, transition metals like Fe, Mo, Ru, and Rh strongly adsorb N_2_ but also tend to overbind hydrogen, which promotes HER.^274^ In contrast, early transition metals (e.g., Ti, Zr, Y) exhibit low HER activity but often fail to activate N_2_ efficiently.^275^ HEA nanocrystals have recently attracted attention as potential candidates to address these trade-offs, owing to their diverse compositional environments. Their multicomponent nature may allow the integration of distinct catalytic functions within a single disordered lattice, where certain elements facilitate N_2_ activation, others modulate hydrogen adsorption, and the overall entropy-stabilized structure supports stability under electrochemical conditions. While these features suggest promising avenues, further mechanistic studies are needed to validate the effectiveness of HEA nanocrystals in nitrogen reduction and to clarify the role of their local atomic environments.

A recent study reported the synthesis of RuFeCoNiCu HEA nanocrystals via a low-temperature (≤250 °C), atmospheric-pressure oil-phase route.^121^ The resulting nanocrystals exhibited uniform morphology with an average size of *ca*. 16 nm and were evaluated for NRR performance. The catalyst showed higher current density and stability in the N_2_-saturated electrolyte and achieved an NH_3_ yield of 57.1 μg h^–1^ mg^–1^ and an FE of 38.5% at 0.05 V_RHE_ in N_2_-saturated 0.1 M KOH (Figure 48A–D). In addition to alkaline media, the material also showed stable activity across different electrolytes, including 0.1 M Li_2_SO_4_, Na_2_SO_4_, and HCl, reflecting good electrolyte compatibility. Notably, during the 100-h continuous electrolysis test, the time–current response remained stable without noticeable decay, and the catalytic activity measured before and after the reaction showed negligible loss (Figure 48D), confirming the excellent chemical and electrochemical stability of the RuFeCoNiCu/CP catalyst. DFT calculations suggest that Fe atoms coordinated by neighboring alloying elements are key sites for N_2_ adsorption and activation, while Co–Cu and Ni–Ru pairs promote efficient surface hydrogenation at low overpotential, collectively supporting the enhanced NRR performance.

**Figure 48:**
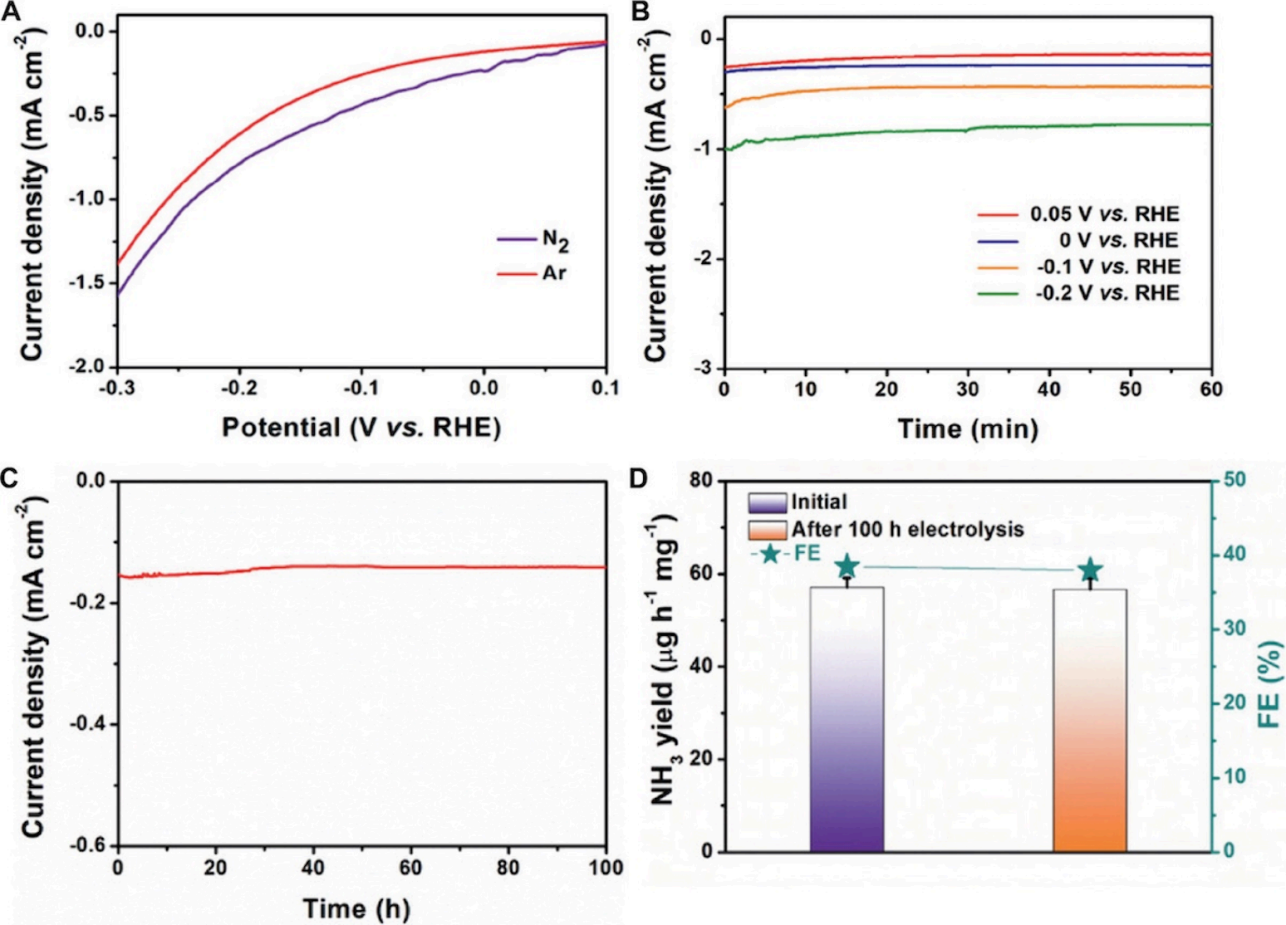
(A) LSV curves of RuFeCoNiCu/CP in N_2_- and Ar-saturated 0.1 M KOH with a scan rate of 5 mV s^–1^. (B) Time-dependent current density curves at various potentials in N_2_-saturated 0.1 M KOH. (C) Time-dependent current density curve for RuFeCoNiCu/CP at the potential of 0.05 V_RHE_. (D) NH_3_ yields and FEs after reacting at 0.05 V_RHE_ for 1 and 100 h. Reproduced with permission from ref 121. Copyright 2021 Wiley-VCH.

#### 6.1.7 Nitrate Reduction Reaction (NO3RR)

NO_3_RR presents a promising pathway for sustainable ammonia synthesis and wastewater nitrate removal, potentially offering a more energy-efficient alternative to the conventional Haber–Bosch process.^276^ Nevertheless, the reaction mechanism is complex, involving an eight-electron, nine-proton transfer that generates multiple intermediates, such as *NO_3_, *NO_2_, *NO, and *NH_2_OH, and is often accompanied by competing reactions like the HER. This multistep sequence makes it challenging to achieve both high conversion efficiency and product selectivity.

To date, catalyst development has largely focused on noble metals (e.g., Pd, Au, Ag) and base-metal alloys (e.g., Fe, Co, Ni, Cu), where alloying strategies are employed to adjust adsorption energetics.^277–279^ However, these systems are typically limited to binary or ternary combinations, leaving the broader design space of multicomponent catalysts relatively unexplored. HEA nanocrystals offer a compelling platform to address these constraints. Their chemically diverse surfaces introduce a wide distribution of active sites with varying adsorption properties, which can facilitate selective stabilization of reaction intermediates and decoupling of adsorption energies. In addition, synergistic interactions between constituent elements may modify charge transfer characteristics and weaken scaling relationships, thereby enhancing NO_3_RR selectivity while concurrently suppressing HER. The entropy-stabilized solid-solution structure also contributes to mechanical and chemical durability under reductive electrochemical conditions. These attributes suggest that HEAs hold significant potential for the development of efficient and stable electrocatalysts for reduction of NO_3_^–^ to NH_3_, although further studies are needed to fully understand and optimize their performance.

Recently, Jiang and co-workers reported a wet-chemical synthesis strategy to prepare PtFeCoNiCu HEA nanocrystals doped with various rare-earth elements.^135^ Among these, Ce-doped HEA exhibited the most promising catalytic performance for electrochemical NO_3_RR. The Ce incorporation led to a notable improvement in onset potential (−0.04 V_RHE_), outperforming both the undoped HEA (−0.10 V_RHE_) and PtCu reference (−0.50 V_RHE_) (Figure 49A). In addition, the catalyst delivered a high FE of 98.8%, an ammonia yield rate of 0.22 mmol h^–1^ cm^–2^, and nearly complete nitrate conversion with 97.8% NH_3_ selectivity (Figure 49B, C). To elucidate the origin of the enhanced performance, DFT calculations were conducted (Figure 49D, E). Results indicated that Ce doping shifts the d-band center of the overall HEA and Pt sites, leading to stronger binding of key NO_3_RR intermediates and weaker *H adsorption, which helps suppress the competing HER. Moreover, specific active sites were identified: Ni sites promote water dissociation, Cu and Fe sites favor *NO_3_ adsorption, and Pt sites facilitate *NO protonation. Notably, the energy barrier for the critical *NO → *NOH step was significantly reduced on Ce-doped HEA compared to undoped HEA, favoring ammonia formation through a preferred reaction pathway. These results highlight that the improved catalytic performance originates from the synergistic interaction among multiple active sites within the Ce-doped HEA matrix (Figure 49F). Each constituent metal contributes a distinct role such as facilitating water dissociation, enhancing nitrate adsorption, or enabling intermediate hydrogenation, promoting a self-tandem reaction mechanism for NO_3_RR. The proposed mechanism, illustrated schematically, underscores how spatially distributed functionalities across the multicomponent surface cooperate to accelerate sequential reaction steps, thereby achieving high efficiency and selectivity.

**Figure 49:**
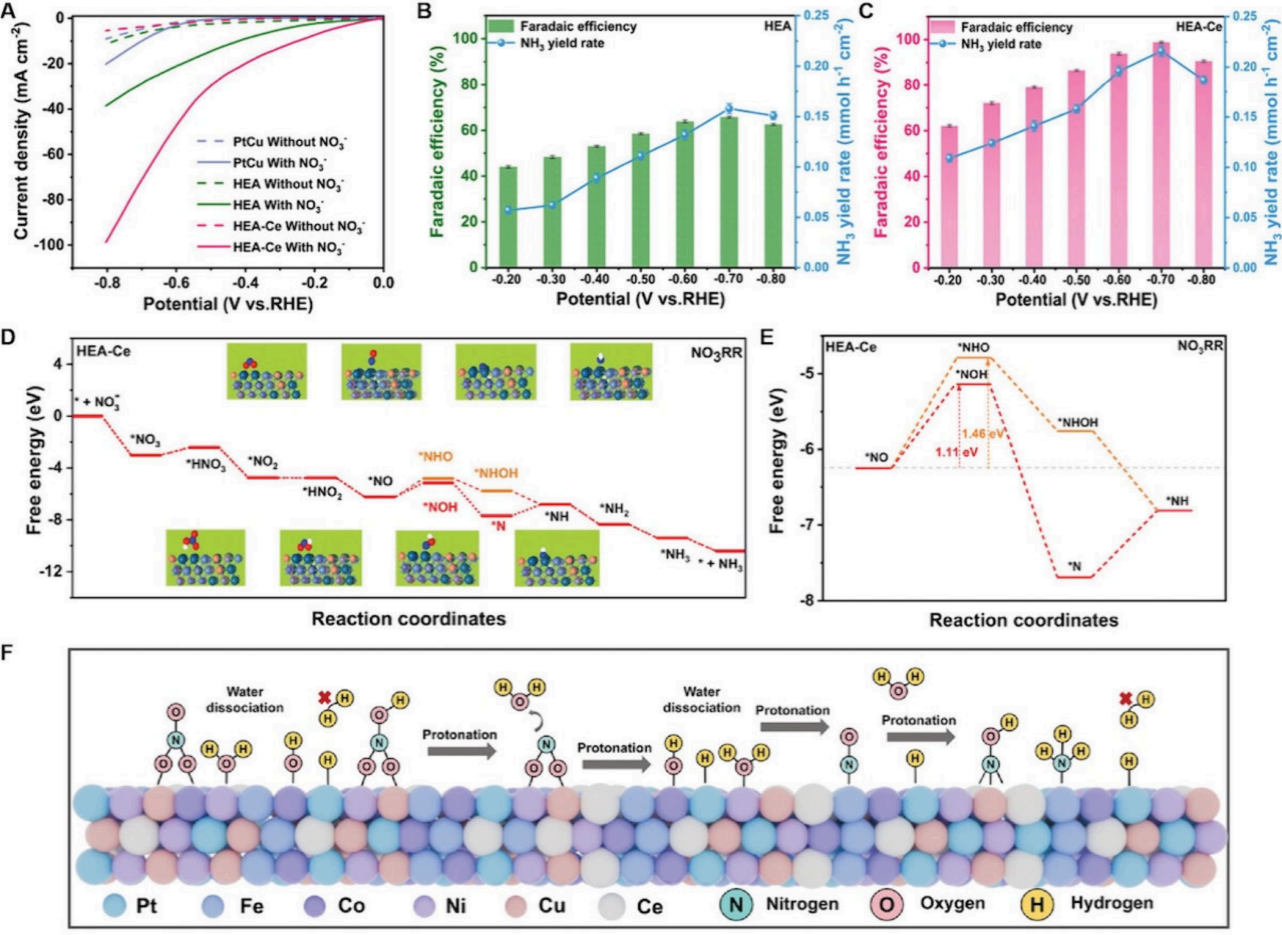
(A–C) Comparison of PtCu, PtFeCoNiCu HEA, and Ce-modified HEA (HEA-Ce) catalysts. (A) LSV curves obtained in the respective electrolytes. (B,C) FE and NH_3_ yield rate for HEA and HEA–Ce catalysts. Reaction coordinates in NO_3_RR for each step on (D) HEA-Ce and (E) corresponding free energy change for *NO protonation step. (F) Schematic illustration of intrinsic self-tandem catalytic mechanism for NO_3_RR on HEA-Ce. Reproduced with permission from ref 135. Copyright 2025 Wiley-VCH.

For another example, Qiu and co-workers fabricated a transition-metal HEA catalyst by integrating Mn, Fe, Co, Cu, and Ni into a Prussian blue analogue followed by pyrolysis.^280^ The resulting Co_1_Ni_2_Cu_1_Mn_1_Fe_1_ HEA displayed the highest NO_3_RR activity, achieving a low Tafel slope of 216 mV dec^–1^ and an NH_3_ yield rate of 3.25 mg h^–1^ cm^–2^ at −0.6 V_RHE_. The enhanced performance was attributed to synergistic interactions among Co, Ni, and Cu sites, which promoted NO_3_^–^ activation and *NO_2_ hydrogenation, while the homogeneous HEA framework facilitated efficient electron transfer and structural stability during prolonged operation. These findings suggest that rational design of multicomponent alloys, with site-specific functionalities and optimized intermediate binding energies, may unlock new strategies for achieving selective, efficient, and durable ammonia synthesis from nitrate. Further mechanistic studies and material optimization will be essential to fully realize the promise of HEAs in practical nitrogen conversion technologies.

#### 6.1.8 Alcohol Oxidation Reactions

AOR plays a pivotal role in the advancement of direct alcohol fuel cells, offering potential advantages such as liquid fuel flexibility and reduced emissions.^281^ However, the electrooxidation of small alcohols like methanol and ethanol involves intricate multielectron transfer steps and numerous adsorbed intermediates (e.g., *CO, *CHO, *CH_*x*_, *CH_3_CO), which pose kinetic challenges and increase the susceptibility to surface poisoning, particularly by CO-like species. Traditional catalysts based on Pt or Pd, while effective, often encounter a fundamental trade-off between catalytic activity, selectivity toward complete oxidation, and long-term durability.^282^

To address these limitations, alloy-based approaches have been widely explored to tune the surface electronic structure and intermediate binding energies.^283,284^ In this context, HEA nanocrystals present a compelling extension, offering a multicomponent platform in which a diverse set of elements can contribute distinct catalytic roles. For example, certain elements may promote initial alcohol dehydrogenation, while others facilitate the generation of reactive *OH species necessary for subsequent oxidation steps, and yet others help mitigate CO adsorption. This cooperative interaction among multiple active sites may allow HEAs to achieve a more favorable balance between intermediate stabilization and poisoning resistance. Moreover, the electronic interactions and lattice distortions inherent to the HEA structure can influence the d-band center and local coordination environments, potentially enhancing both reactant activation and the oxidative removal of poisoning species. While further investigation is needed to fully elucidate these mechanisms, HEA nanocrystals represent a promising direction for the rational design of next-generation AOR electrocatalysts.

Wu and co-workers synthesized RuRhPdOsIrPt HEA nanoparticles via a polyol method for EOR (Figure 50A–C).^120^ The catalyst exhibited the highest current density among the tested systems (Figure 50A), with surface (mass) activities 2.5 (3.2) and 12.8 (3.4) times higher than Pd/C and Pt/C, respectively (Figure 50B). CV curves in the positive scan for HEA and monometallic nanoparticles, revealing distinct CO-stripping behaviors and more efficient CO oxidation on the HEA surface (Figure 50C).

**Figure 50:**
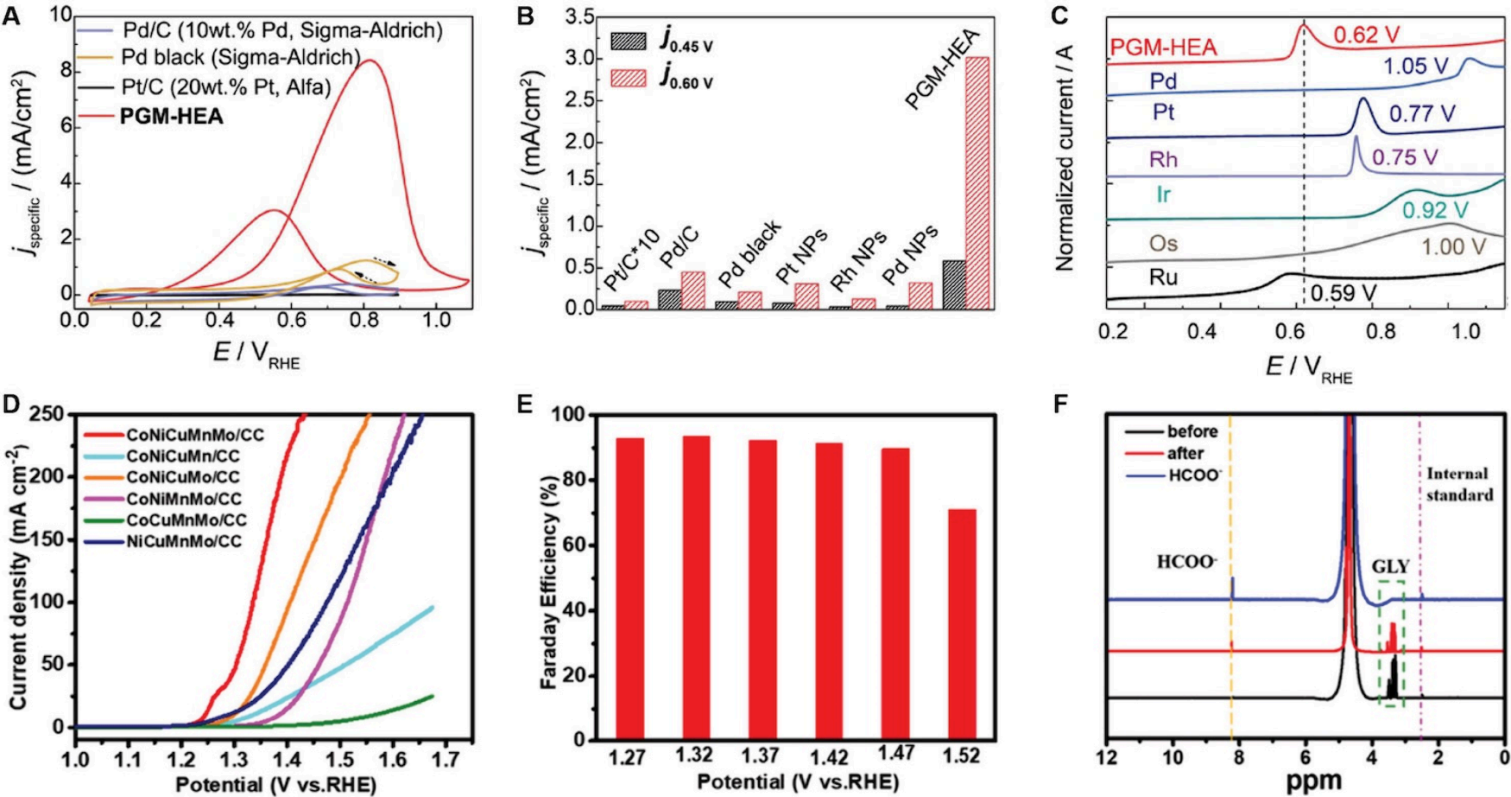
Representative examples of HEA catalysts for the AOR. (A–C) Noble-metal RuRhPdOsIrPt HEA (PGM-HEAs) catalysts. (A) Comparison of specific current densities (*j*_*specific*_) of PGM-HEA with those of commercial catalysts; arrows indicate the scan directions of CVs. (B) Specific current densities of PGM-HEA at 0.45 V_RHE_ and 0.6 V_RHE_ in the forward scan compared with monometallic counterparts, with Pt/C currents magnified 10-fold for clarity. (C) CV curves in the positive scan for PGM-HEA and monometallic nanoparticles (NPs). (D–F) CoNiCuMnMo-HEA catalyst. (D) Comparison of anodic glycerol oxidation activities for various catalysts in 1 M KOH containing 0.1 M glycerol (IR-corrected, 2 mV s^–1^). (E) FEs at different applied potentials. (F) ^1^H nuclear magnetic resonance (NMR) spectra of glycerol oxidation products, with dimethyl sulfoxide used as an internal standard (purple). The images in (A–C) were reproduced with permission from ref 120. Copyright 2020 American Chemical Society. The images in (D–F) were reproduced with permission from ref 285. Copyright 2022 American Chemical Society.

As another representative case, the glycerol oxidation reaction (GOR) has emerged as a promising anodic alternative to the OER owing to its lower thermodynamic potential and the abundance of biomass-derived glycerol. Fan and co-workers developed a cost-effective, high-performance GOR catalyst by *in situ* growth of quinary CoNiCuMnMo metal–organic frameworks on carbon cloth, followed by pyrolytic reduction (Figure 50D–F).^285^ The resulting HEA catalyst delivered a current density of 10 mA cm^–2^ at 1.25 V_RHE_ and an FE exceeding 90% across a wide potential range (Figure 50D, E) and converted glycerol to HCOO^–^ (Figure 50F). These studies collectively highlight the promising potential of HEA-based catalysts for AOR. By harnessing the synergistic interactions among multiple elements, HEAs can effectively integrate various catalytic functions, enhancing activity, improving CO tolerance, and enabling selective oxidation of alcohols such as ethanol and glycerol. Nevertheless, the potential of HEA nanocatalysts toward AOR remains underexplored. A deeper understanding of active-site distributions, surface restructuring under reaction conditions, and alcohol-conversion mechanisms will be essential to the rational design of HEA catalysts and their broader application in biomass-derived alcohols and fuel cell technologies.

### 6.2 Thermocatalysis

Thermocatalysis presents some of the most demanding conditions for heterogeneous catalysts, requiring sustained activity, thermal stability, and resistance to sintering and poisoning at elevated temperatures.^286^ Traditional catalysts often suffer from agglomeration, phase segregation, and active-site loss under harsh conditions. In contrast, HEA nanocrystals provide improved thermal stability because sluggish diffusion and lattice distortion help suppress sintering and grain growth, while their multielement surfaces offer diverse active sites and may undergo adaptive surface reconstruction under reaction conditions. These features make HEAs promising thermocatalysts. This section highlights ammonia decomposition and carbon monoxide oxidation as representative examples.

#### 6.2.1 Ammonia Decomposition

Ammonia serves dual functions in thermocatalysis, as a hydrogen carrier via decomposition (NH_3_ → 1/2N_2_ + 3/2H_2_).^287^ Conventional catalysts based on Ru or Pt often suffer from sintering, phase segregation, and support degradation under high-temperature conditions, limiting their long-term performance.^288,289^ In contrast, HEA nanocrystals provide enhanced thermal stability and catalytic efficiency through their entropy-stabilized single-phase structures and cooperative multisite architectures. The atomic-level mixing ensures proximity between oxidation and dehydrogenation sites, facilitating efficient N–H bond activation and O–O coupling while mitigating particle coarsening. During ammonia decomposition, HEA nanocrystals enable complete conversion at lower temperatures, as electronic coupling modulates the energetics of N_2_ desorption and H recombination. Meanwhile, lattice distortion and sluggish atomic diffusion inhibit phase separation across redox cycles. These features collectively endow HEA nanocrystals with both high activity and exceptional durability under thermally demanding conditions.

For example, a recent study demonstrated efficient ammonia decomposition using a series of HEA nanocrystals composed entirely of earth-abundant elements.^55^ Quinary CoMoFeNiCu nanocrystals were successfully synthesized as single-phase solid solutions, maintaining compositional uniformity even at Co/Mo ratios that are typically immiscible in conventional Co–Mo bimetallic systems. The catalytic performance of these HEA-Co_*x*_Mo_*y*_ nanocrystals was evaluated in a plug-flow reactor using 5 vol % NH_3_ as the feed gas at temperatures ranging from 250 to 600 °C. All HEA nanocrystals exhibited an onset of NH_3_ decomposition near 300 °C, with conversion increasing steadily as temperature rose. The conversion order between 300 and 500 °C followed HEA-Co_25_Mo_45_ > HEA-Co_35_Mo_35_ > HEA-Co_15_Mo_55_ > HEA-Co_45_Mo_25_ > HEA-Co_55_Mo_15_ (Figure 51A). Notably, most HEA catalysts displayed substantially higher activity than both the bimetallic Co–Mo and monometallic Ru benchmarks, which achieved only 46% and 73% NH_3_ conversion at 600 °C, respectively. The mass-specific reaction rates at 500 °C (Figure 51B) further highlight the superior performance of the HEA catalysts. The HEA-Co_25_Mo_45_ and HEA-Co_35_Mo_35_ variants delivered rates of 22.1 and 16.7 g_NH_3__ g_metals_^–1^ h^–1^, corresponding to enhancements of roughly 19-fold and 14-fold relative to Co–Mo. Beyond their high activity, the HEA catalysts also demonstrated excellent operational stability. Continuous testing of HEA-Co_25_Mo_45_ at 500 °C showed negligible degradation in NH_3_ conversion over a 50-h run, confirming the strong structural and chemical robustness of the HEA system under high-temperature reaction conditions (Figure 51C). Based on their DFT calculations and experimental findings, the proposed mechanism for ammonia decomposition over HEA-Co_*x*_Mo_*y*_ catalysts highlighting the role of nitrogen adsorption strength in determining catalytic performance (Figure 51D). In accordance with the Sabatier principle, a volcano-shaped relationship is observed: when nitrogen binds too weakly (in Co-rich compositions), the activation of the N–H bond becomes rate-limiting; conversely, in Mo-rich systems where nitrogen binds too strongly, N_2_ desorption is hindered. The optimal performance of HEA-Co_25_Mo_45_ is attributed to its intermediate nitrogen binding energy, which balances NH_3_ activation and N_2_ release, thereby enabling efficient and stable ammonia decomposition.

**Figure 51:**
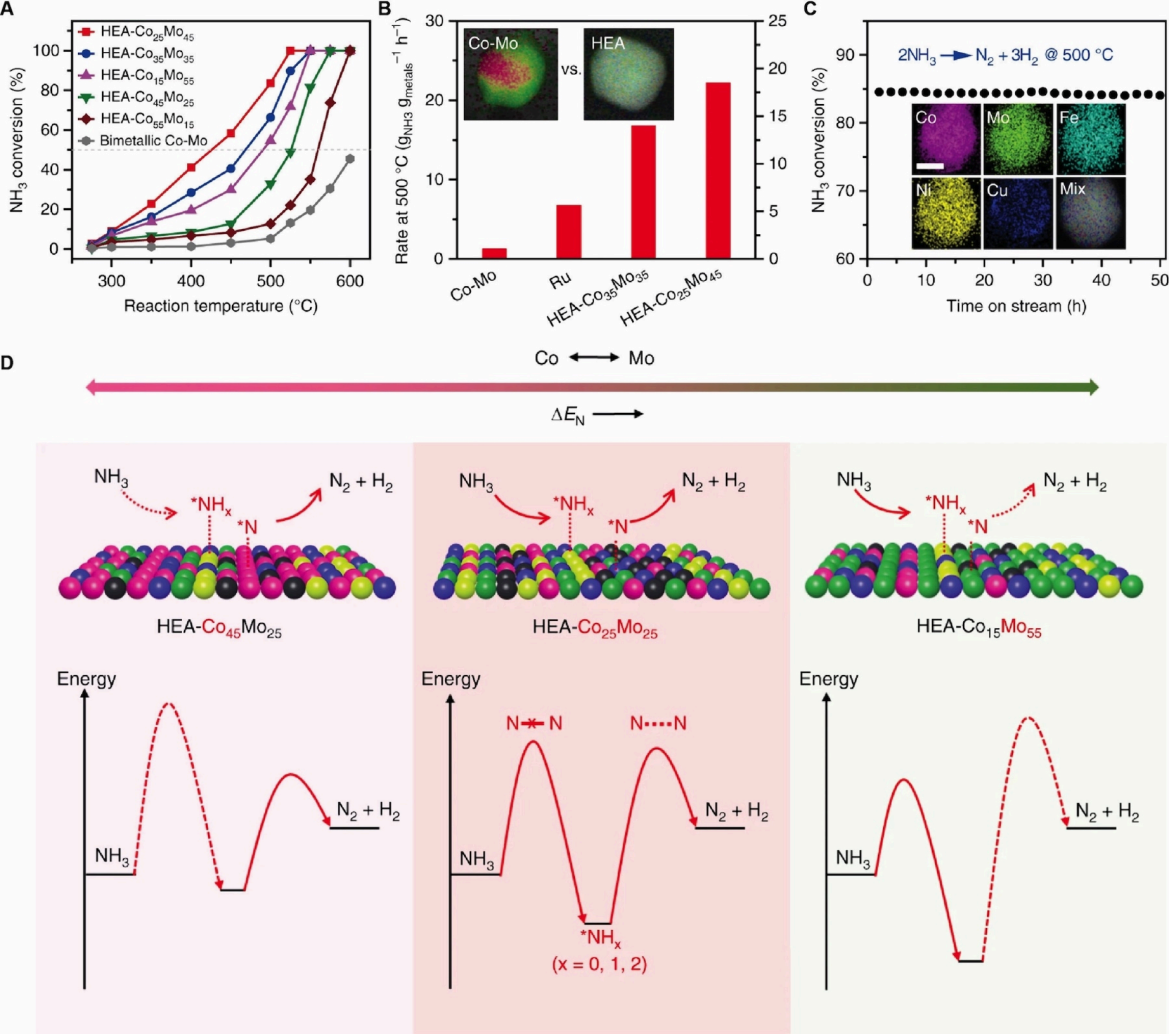
Performance of HEA-Co_*x*_Mo_*y*_ catalysts for NH_3_ (5 vol %) decomposition. (A) NH_3_ conversions over different HEA-Co_*x*_Mo_*y*_ nanoparticles and bimetallic Co–Mo (Co/Mo = 25/45) depending on the reaction temperature (Space velocity = 36 L g_cata_^–1^ h^–1^). (B) Comparison of reaction rates measured in the kinetic regime among bimetallic Co–Mo, Ru, and HEA-Co_*x*_Mo_*y*_ catalysts (*T* = 500 °C). Inset: element maps for the bimetallic Co–Mo (Co/Mo = 25/45) and HEA-Co_25_Mo_45_ catalysts. (C) Stability test performed at 500 °C for the HEA-Co_25_Mo_45_ catalyst. Inset: element map of the catalyst after the stability test; scale bar = 10 nm. (D) Schematic illustration of the rate-limiting factors in NH_3_ decomposition. The rate-limiting factors are labeled with dash lines in the lower panel. On Co-rich surface (left), the rate is limited by activation or dehydrogenation of NH_3_; on Mo-rich surface, the rate is limited by the recombinative desorption of *N; Balance for these two steps is reached on an intermediate composition (HEA-Co_25_Mo_45_). Reproduced with permission from ref 55. Copyright 2019 Springer Nature.

For another example, Yao and co-workers synthesized single-phase PtPdRhRuCe HEA nanocrystals via a carbothermal-shock process and applied them to NH_3_ decomposition.^147^ The uniform elemental distribution imparted exceptional stability and performance, achieving nearly 100% NH_3_ conversion and over 99% NO_*x*_ selectivity for 30 h at a relatively low operating temperature of 700 °C. These studies highlight the potential of HEA nanocrystals in combining high activity with sustained stability toward ammonia decomposition. Their tunable composition, structural robustness, and multisite synergy make them promising thermocatalysts under harsh conditions.

#### 6.2.2 Carbon Monoxide Oxidation

The oxidation of CO exemplifies how HEAs integrate multiple catalytic functionalities, including oxygen activation, poison removal, and structural resilience, within a single framework. The essential design logic is to spatially distribute redox and adsorption functions across neighboring elements, ensuring a continuous oxygen supply and sustained activity under thermal stress.

For example, Qiu and co-workers synthesized an AlNiCuPtPdAu nanoporous HEA that exhibited exceptional CO oxidation activity and high-temperature stability.^105^ The high-entropy stabilization achieved through the incorporation of multiple immiscible elements prevented phase separation and coarsening, maintaining a single-phase structure even at elevated temperatures. During or after dealloying, a thin γ-Al_2_O_3_ spinel layer formed on the surface through reaction with oxygen, enhancing catalytic performance by promoting O_2_ dissociation while acting as a protective barrier against thermal degradation (Figure 52A). As a result, the AlNiCuPtPdAu np-HEA maintained 100% CO conversion at 150 °C (Figure 52B) for over 600 min (Figure 52C), demonstrating the dual benefits of entropy-driven stability and interfacial synergy between the HEA core and oxide shell. Although current research on HEA nanocrystals for CO oxidation remains limited, the field holds significant promise. In particular, the development of noble-metal-free HEA compositions could further expand the practical applicability of these materials, offering a cost-effective and thermally robust platform for catalytic oxidation reactions.

**Figure 52:**
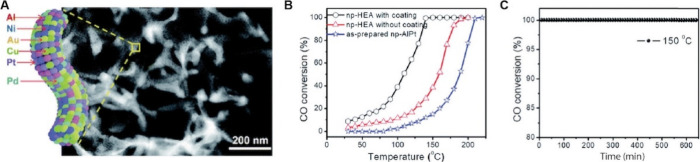
AlNiCuPtPdAu nanoporous HEA (np-HEA) catalysts. (A) Schematic representation of the np-HEA architecture. (B) CO conversion as a function of reaction temperature over np-HEA with and without oxide coating. (C) Long-term CO conversion stability test of oxide-coated np-HEA at 150 °C. Reproduced with permission from ref 105. Copyright 2019 Royal Society of Chemistry.

### 6.3 Photocatalysis

Typical HEA nanocrystals lack intrinsic light absorption, limiting their direct application in photocatalysis. To overcome this, HEA nanocrystals are often integrated with semiconductor supports such as commonly used TiO_2_ to form composite structures. In such systems, HEA nanocrystals can promote efficient charge separation of photoexcited electron–hole pairs and facilitate rapid interfacial charge transfer during photocatalytic reactions. The high-entropy design further enhances these effects by introducing atomic-level disorder, lattice distortion, and charge redistribution. These features prolong carrier lifetimes, stabilize the electronic structure under illumination, and improve photochemical durability. Moreover, the multielement synergy can tailor the surface density of states to optimize reaction selectivity. Together, these properties render HEA nanocrystals as compositionally tunable, structurally robust, and functionally versatile cocatalysts for next-generation photocatalysis. In the following subsections, we highlight two representative applications: photocatalytic hydrogen evolution and CO_2_ reduction.

#### 6.3.1 Photocatalytic Hydrogen Evolution

Photocatalytic hydrogen evolution represents a sustainable and carbon-neutral strategy for converting solar energy into chemical energy by generating hydrogen fuel.^290^ However, traditional semiconductor photocatalysts face intrinsic limitations, including narrow light absorption ranges, rapid electron–hole recombination, and insufficient redox potentials to simultaneously drive hydrogen and oxygen evolution reactions. These issues result in low quantum efficiency and poor long-term stability under illumination. While metallic cocatalysts can enhance charge separation and provide active sites for redox reactions, conventional monometallic or bimetallic nanocrystals often suffer from fixed electronic structures and a limited diversity of active sites. HEA nanocrystals offer a promising alternative by incorporating multiple elements into a single solid-solution lattice, yielding tunable electronic structures and high configurational entropy. Their compositionally heterogeneous surfaces enable the formation of multifunctional catalytic sites that can facilitate both hydrogen and oxygen evolution reactions under light irradiation. Moreover, strong electronic coupling among the constituent elements promotes efficient charge separation, modulates the work function, and optimizes hydrogen adsorption energies. The entropy-stabilized framework also provides enhanced resistance to photocorrosion and structural degradation. Collectively, these features make HEA nanocrystals efficient photocatalysts for hydrogen production.

Recently, Lin and co-workers designed Pd@Pt_0.4_Pd_0.15_Ir_0.15_Ru_0.15_Rh_0.15_ core–shell nanocubes enclosed by {100} facets loaded on TiO_2_ supports, which exhibited the highest hydrogen evolution activity among tested systems.^291^ The catalyst achieved a hydrogen production rate of 2.31 ± 0.46 μmol h^–1^, surpassing that of Pd@Pt/TiO_2_ (1.71 ± 0.33 μmol h^–1^) and Pd nanocubes/TiO_2_ (1.46 ± 0.22 μmol h^–1^) (Figure 53A). Despite identical composition, the core–shell nanocubes also outperformed their octahedral counterparts covered by {100} facets (*ca*. 1.31 μmol h^–1^), underscoring the decisive role of surface atomic arrangement in catalytic activity (Figure 53B). The turnover frequency, defined as hydrogen production per second per surface metal atom, further confirmed superior intrinsic activity, reaching 90 ± 19.4 h^–1^, the highest among all photocatalysts evaluated. Ultraviolet photoelectron spectroscopy measurements show that Pd@Pt_0.4_Pd_0.15_Ir_0.15_Ru_0.15_Rh_0.15_ core–shell nanocubes exhibit a high work function of 4.81 eV, which favors efficient charge transfer at the interface with TiO_2_. Complementary transient absorption spectroscopy reveals a markedly extended carrier lifetime of approximately 4 ms, substantially longer than that of pristine TiO_2_ (65 ms), indicating enhanced charge separation and suppressed recombination. Moreover, *in situ* X-ray photoelectron spectroscopy demonstrates that photoexcited electrons predominantly accumulate on Pt and Ir atoms, enriching their electron density and identifying them as the key active sites for subsequent photocatalytic processes. DFT calculations of Δ*G*_H*_ were performed on over 100 atomic configurations for hydrogen adsorption on Pt_0.4_Pd_0.15_Ir_0.15_Ru_0.15_Rh_0.15_ {100} surfaces, yielding an average Δ*G*_H*_ of −0.212 eV (Figure 53C, D). The shorter Pt–H (1.752 Å) and Ir–H (1.745 Å) bond lengths, compared to Ru–H, Rh–H, and Pd–H, indicate stronger hydrogen binding at Pt and Ir sites, supporting their role as primary adsorption centers, consistent with the *in situ* XPS data. Hydrogen was found to preferentially adsorb at bridge sites, which exhibit a narrower Δ*G*_H*_ distribution than on-top sites, indicating more uniform adsorption. To identify the dominant active sites, Δ*G*_H*_ values were compared across nine Pt- and Ir-based bridge-site pairs. Ir-containing bridges showed stronger hydrogen binding, while Pt-based bridges gave values closer to zero (e.g., −0.187 eV for PtRu vs −0.314 eV for IrRu), suggesting that Pt offers more optimal adsorption energetics (Figure 53E). Figure 53F illustrates the photocatalytic hydrogen evolution over Pd@Pt_0.4_Pd_0.15_Ir_0.15_Ru_0.15_Rh_0.15_ core–shell nanocubes enclosed by {100} facets/TiO_2_ using ascorbic acid as a sacrificial agent. Under illumination, TiO_2_ generates photoexcited electrons that transfer across the Schottky barrier to the HEA nanocubes, where they are stabilized, prolonging carrier lifetimes. These electrons accumulate mainly on Pt and Ir sites, with Pt-based sites serving as dominant active centers due to their near-optimal Δ*G*_H*_ values. Meanwhile, ascorbic acid efficiently scavenges holes, completing the redox cycle and sustaining hydrogen production.

**Figure 53:**
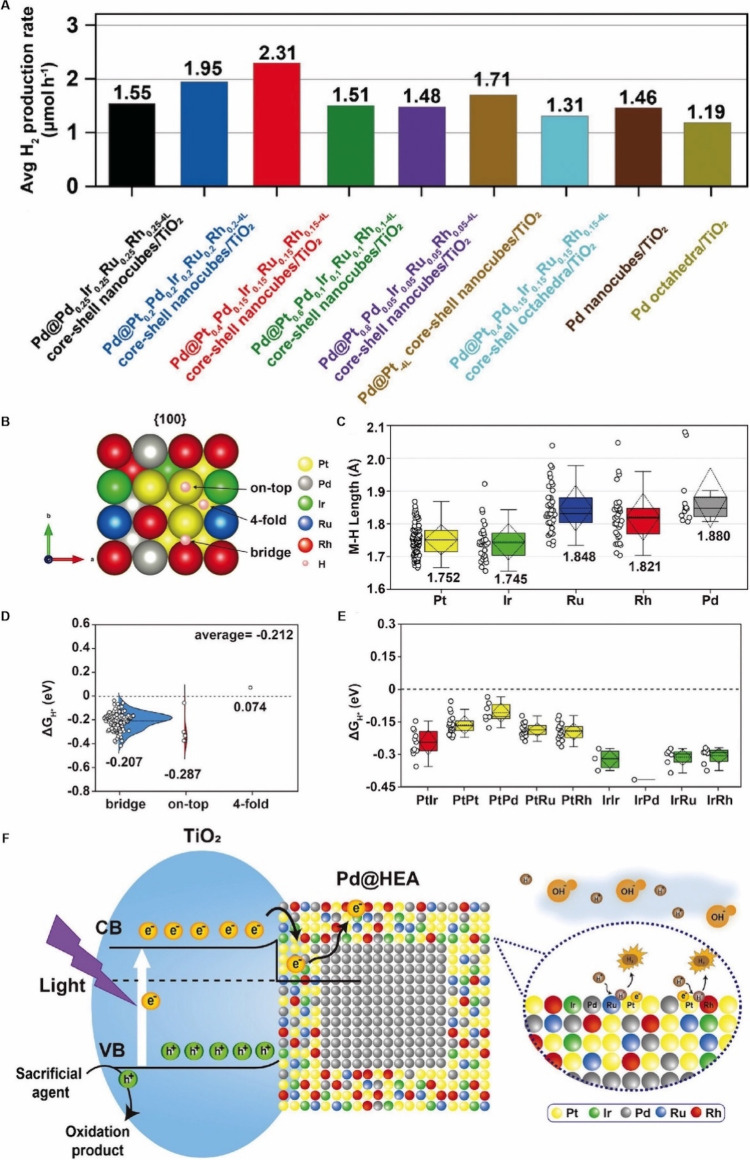
(A) Average hydrogen production rate over the 4-h duration DFT calculations and schematic illustration of the photocatalytic hydrogen production mechanism for the Pd@Pt_0.4_Pd_0.15_Ir_0.15_Ru_0.15_Rh_0.15–4L_ core–shell nanocubes/TiO_2_. (B) Configurations of hydrogen adsorption on Pt_0.4_Pd_0.15_Ir_0.15_Ru_0.15_Rh_0.15_ {100} surfaces. (C) Bond lengths of hydrogen adsorbed at bridge sites on different atomic species. (D) Δ*G*_H*_ values and their distributions across the bridge, on-top, and 4-fold adsorption sites. (E) Comparative analysis of Δ*G*_H*_ values at Pt-based and Ir-based bridge sites. (F) Schematic mechanism of Pd@Pt_0.4_Pd_0.15_Ir_0.15_Ru_0.15_Rh_0.15–4L_ core–shell nanocubes/TiO_2_ for photocatalytic hydrogen production. Reproduced with permission from ref 291. Copyright 2025 Wiley-VCH.

In addition to photocatalysis, photoelectrochemical (PEC) systems also benefit from the integration of HEA nanocrystals with semiconducting scaffolds. A recent study demonstrated a hybrid PEC architecture combining PtFeCoNiCu HEA nanocrystals with TiO_2_ nanofilms through sequential anodization and electrodeposition.^292^ In this design, the TiO_2_ nanofilm serves not only as a photoactive semiconductor but also as a nanostructured substrate that directs the nucleation and island-like growth of HEA nanoparticles via a Volmer–Weber mechanism. This controlled interface enables efficient charge transfer and catalyst-substrate integration, leading to exceptional hydrogen evolution performance. Under light illumination and elevated electrolyte temperature, the hybrid system achieved an ultralow overpotential of −11 mV at 10 mA cm^–2^, underscoring the potential of HEA-based photoelectrocatalysts for high-efficiency solar-driven hydrogen production.

Despite these advances, the field remains in its early stages, with substantial opportunities for future exploration. Key directions include the rational design of low-cost, noble-metal-free HEA nanocrystals; atomic-level understanding of charge transfer and interfacial dynamics; precise control over HEA–semiconductor junctions; and the development of broadband light absorbers through compositionally engineered HEA systems. Moreover, coupling HEA nanocrystals with emerging semiconductor architectures, such as heterojunctions, quantum dots, and 2D materials, could unlock synergistic effects not yet realized. In sum, HEA nanocrystals offer a versatile and largely untapped material space for advancing photocatalytic and photoelectrochemical technologies.

#### 6.3.2 Photocatalytic Carbon Dioxide Reduction

Photocatalytic CO_2_ reduction into value-added fuels and chemicals provides a promising strategy for mitigating carbon emissions and promoting a closed carbon cycle.^293,294^ While conventional semiconductor photocatalysts have been widely studied, their practical performance is often hindered by limited light absorption, fast charge recombination, and sluggish surface reaction kinetics.^295^ These issues result in low efficiency and limited product selectivity. Cocatalysts can improve photocatalytic performance by promoting charge separation and surface reactions, but conventional ultrasmall nanocrystals often suffer from instability and aggregation under irradiation. HEA nanocrystals may overcome these limitations because their multicomponent structures can enhance stability and tune local electronic properties for improved interfacial charge transfer and CO_2_ activation. These features make HEAs promising cocatalysts for solar-driven CO_2_ conversion, albeit further studies are still needed.

Recently, Huang and co-workers reported noble-metal-free FeCoNiCuMn HEA nanocrystals that exploit overlapping 3d-state hybridization to create continuous electron-transfer pathways.^296^ Under illumination, Cu serves as an electron reservoir, Mn/Fe/Co act as electron-deficient acceptor sites, and Ni bridges intersite charge transport (Figure 54A). Photocatalytic CO_2_ reduction with H_2_O vapor under simulated solar light was conducted using HEA/TiO_2_ composites. Bare TiO_2_ exhibited moderate CO and CH_4_ production rates of 10.9 and 0.7 μmol g^–1^ h^–1^, respectively. Incorporation of 0.25 wt % HEA increased the rates to 80 and 16 μmol g^–1^ h^–1^, while 0.5 wt % loading achieved optimal activity, 235.2 μmol g^–1^ h^–1^ for CO and 19.9 μmol g^–1^ h^–1^ for CH_4_, representing *ca*. 23-fold enhancement over pristine TiO_2_ (Figure 54B). The composite also exhibited excellent photostability, maintaining activity over nine consecutive 4-h cycles (36 h total) without detectable degradation (Figure 54C). These findings underscore the potential of HEA nanocrystals, particularly those based on earth-abundant elements, as robust platforms for photocatalytic CO_2_ reduction. With continued advances in compositional tuning and integration with semiconductor hosts, it may become possible to not only enhance efficiency and durability, but also to steer product selectivity toward more complex multicarbon molecules, an exciting direction for future research.

**Figure 54:**
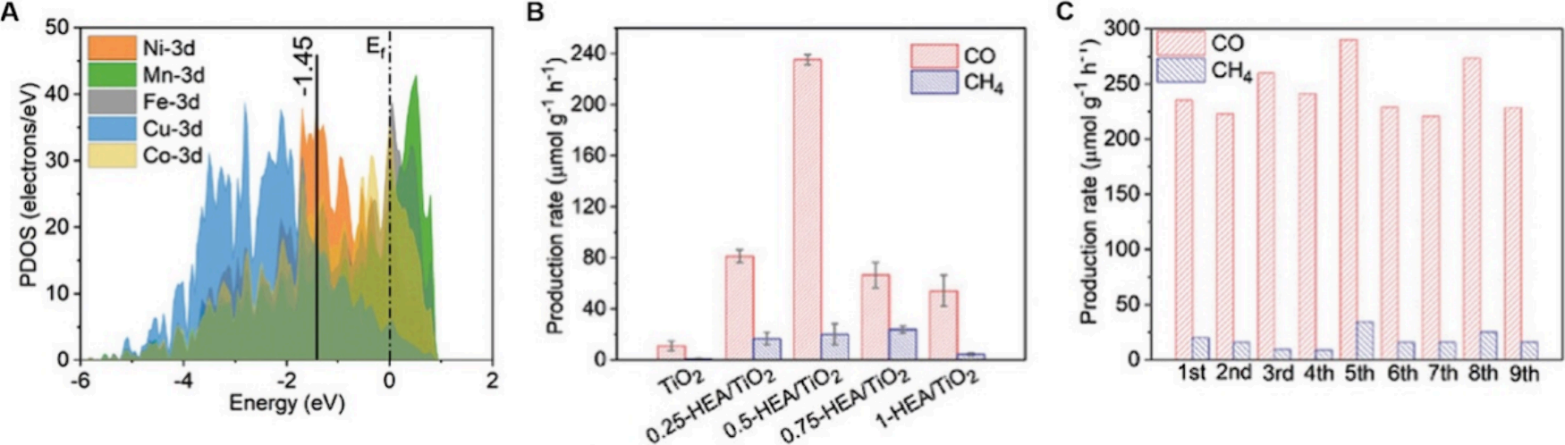
Representative examples of HEA nanocatalysts for photocatalytic reaction. (A–C) FeCoNiCuMn HEA-modified TiO_2_ photocatalysts. (A) Calculated d-band structure of HEA nanoparticles. (B) CO_2_ photoreduction performance of pure TiO_2_ and x-HEA/TiO_2_ composites under AM 1.5G simulated sunlight (100 mW cm^–2^, 1 atm CO_2_, 10 μL H_2_O, 4 h). (C) Recycling stability of 0.5-HEA/TiO_2_ over multiple cycles. Reproduced with permission from ref 296. Copyright 2024 Wiley-VCH.

In summary, HEA nanocrystals represent a transformative platform for catalytic design, where composition, facet, and structural control synergistically govern performance. Unlike conventional elemental catalysts with uniform surface chemistries and narrow adsorption landscapes, HEA nanocrystals feature a vast distribution of local atomic environments and broadened electronic states. This compositional diversity creates a nearly continuous range of adsorption strengths and promotes cooperative interactions among multiple active sites. Such multisite behavior helps overcome the linear scaling limitations found in conventional catalysts, enabling more balanced intermediate adsorption that follows the Sabatier principle and facilitates reactions with lower energy barriers. Beyond compositional tuning, facet and structural engineering can be utilized to further regulate active-site environments and catalytic behavior. Together with lattice distortion and sluggish diffusion, these features enable HEA nanocrystals to provide diverse near-optimal adsorption sites with high stability, offering a robust platform for a wide range of catalytic reactions.

## 7 Conclusions and Outlook

HEA nanocrystals have emerged as a transformative class of catalytic materials that naturally integrate compositional complexity with structural tunability.^25^ The four principal effects, including high entropy, lattice distortion, sluggish diffusion, and cocktail mixing, collectively enable unique stability and synergistic functions.^12^ By tailoring the elemental combinations, their physicochemical properties can be systematically varied and optimized, leading to enhanced catalytic activity, selectivity, and durability.^23,29,297^ The vast compositional space of HEA nanocrystals offers an unprecedented platform for exploring composition–structure–property relationships that are unattainable in conventional alloy systems. Over the past several years, tremendous progress has been made in the development of synthetic strategies capable of tightly controlling the composition and structure of HEA nanocrystals.^143^ Both top-down and bottom-up approaches have been actively explored, among which wet-chemical synthesis has emerged as a powerful route due to its ability to finely regulate the reduction, nucleation, growth, and alloying processes in a solution phase.^59,197^ This route allows for the preparation of HEA nanocrystals with uniform composition and well-defined surface structures, providing atomic level precision that is crucial for understanding the catalytic behavior.

Among the wet-chemical methods, the use of dropwise addition of precursors has emerged as an effective means to modulate the reduction kinetics associated with different metal ions.^196^ By introducing the precursors dropwise rather than in one shot, the synthesis can reach a steady state growth regime in which the production and deposition of each component proceed in a well-balanced and synchronized manner.^80,195^ This dynamic control effectively suppresses phase segregation and promotes the formation of HEA nanocrystals in homogeneous solid solution. More importantly, such a well-controlled growth pattern allows for quantitative prediction of precursor reduction behaviors, enabling transition from empirical synthesis toward controllable and predictable production of multicomponent nanocrystals.^78^ Building upon this concept, a combination of dropwise addition with seed-mediated growth provides an additional level of control to the surface structure. The preformed seeds serve as energetically favorable sites for heterogeneous nucleation, followed by anisotropic growth and facet-selective deposition.^67,298^ This hybrid strategy has been demonstrated to yield HEA nanocrystals with uniform atomic mixing and well-defined facets, establishing a direct correlation between surface atomic configuration and catalytic performance.^78,81,82,120,123,133^

Despite the remarkable progress, fundamental challenges remain before HEA nanocrystals can be fully utilized as advanced catalysts. While recent advances have established HEAs as a versatile platform for tuning composition, structure, and catalytic properties, the development of effective and generalizable design strategies is still at its early stage. To further advance this rapidly evolving field, it is critical to identify and overcome key scientific and technological barriers that limit precision synthesis, in-depth characterization, and practical deployment. In the following sections, we summarize recent progress and highlight outstanding challenges and emerging trends in HEA nanocrystal research, with particular emphasis on how integrated experimental, characterization, and theory-guided approaches can accelerate the rational development of HEA catalysts with controllable atomic configurations and predictable catalytic behavior.

### 7.1 Manipulating the Degree of Atomic-Level Mixing

Although diverse synthetic strategies have been developed for the preparation of HEA nanocrystals, the degree of atomic level mixing can vary substantially, resulting in distinct catalytic behaviors and structure-dependent selectivity. Such compositional heterogeneity arises from several intrinsic factors, including the difference in precursor reduction potentials, reaction kinetics under distinct synthetic environments, preferred crystallographic structures of the constituent elements (e.g., BCC, FCC, or HCP), and variation in interatomic binding energies. These factors collectively govern how atoms distribute and interact during nucleation and growth, ultimately determining whether a multicomponent solid solution or a partially ordered structure will be formed.

Recent studies have revealed that even a subtle change in composition can dramatically alter the local atomic configuration. For instance, replacing FCC-Rh with HCP-Ru in RuPtPdFeCo HEA induces a transition from a randomly mixed atomic arrangement to a locally ordered structure with element specific clustering.^92^ The degree of local ordering can be further tuned by adjusting the Ru content, effectively toggling between homogeneous solid solutions and heterostructured systems. In another example, pulsed annealing of PdSnFeCoNi HEA for short durations (for instance, 0.5 s heating at 1,300 K for 30 cycles) exploits the differences in enthalpic interactions and surface energies to form ultrafine PdSn clusters within the HEA matrix, generating a heterostructured HEA/c-PdSn catalyst with enhanced catalytic activity.^90^ Excessive annealing, however, drives the system toward thermodynamic equilibrium, leading to phase separation and diminished catalytic performance. These examples demonstrate that the degree of atomic mixing is a dynamic outcome governed by both thermodynamic and kinetic factors. Accordingly, achieving uniform atomic mixing remains one of the most critical and challenging goals in HEA nanocrystal synthesis. Controlling the extent of mixing not only determines electronic uniformity but also governs both the reactivity and stability of surface active sites.

To address this issue, our groups have recently developed a deterministic and controllable strategy based on dropwise precursor addition.^78,80–82^ By quantitatively analyzing the reduction kinetics of individual metal precursors and establishing mathematical models that describe the instantaneous generation rates of metal atoms and the corresponding entropy of mixing as a function of reaction time, it becomes possible to tailor experimental parameters to regulate the compositional uniformity of the resulting HEA nanocrystals. This kinetically controlled strategy enables quantitative regulation of atomic mixing through kinetic tuning, offering a rational route to achieving compositionally uniform and stable HEA nanocrystals.

### 7.2 Controlling Facets Using Surface Capping Agents

Achieving precise control of facets on HEA nanocrystals without relying on preformed seeds remains one of the grand challenges for multicomponent systems. Most of the existing methods rely on seed-mediated growth, in which the crystallographic orientation of the seeds, such as the {100} facets on cubic seeds or the {111} facets on octahedral seeds,^202^ can be used to dictate the subsequent epitaxial deposition of HEA overlayers.^74,291^ This approach, while effective for obtaining specific types of facets, inevitably limits structural diversity and scalability, as it requires access to monometallic or bimetallic seeds with well-defined shapes. Developing seedless routes that can still enable anisotropic or facet-selective growth is essential for realizing compositionally complex and morphologically diverse HEA nanocrystals.

Compared with mono- or bimetallic systems, facet control in multicomponent HEAs presents unique difficulties due to the random distribution of constituent elements across the surface. The varied local chemical environments weaken the selective binding affinity of a surface capping agent, making it challenging to direct growth along specific crystallographic planes similar to what has been achieved in the conventional colloidal synthesis. As a result, the proportion of nanocrystals exposing the desired surface configurations is often low, which reduces the impact of selective adsorption and hinders the ability to achieve uniform shape control across large ensembles. Recent studies have attempted to address this limitation through alternative seedless routes. For instance, the selective adsorption of carbon monoxide has been employed to guide the formation of octahedral Ru-doped PtFeNiCuW HEA nanocrystals on carbon nanotube supports.^100^ These studies demonstrate that small-molecule adsorption can influence facet development even in compositionally complex multimetallic systems. However, the underlying mechanism remains poorly understood and appears to depend strongly on the specific chemical interactions between the HEA surface and the carbon support.

Future efforts should focus on developing predictive models that couple theoretical simulation with experimental validation to identify suitable capping agents for stabilizing the facets on HEA nanocrystals. Integrating DFT calculations to screen potential ligands or surfactants capable of preferential adsorption on high-energy surfaces can significantly reduce empirical trial-and-error in shape-controlled synthesis. By combining computational analysis with controlled wet-chemical synthesis, it may become feasible to select facet exposure without relying on preformed seeds, enabling scalable production of HEA nanocrystals with well-defined surface structures.

### 7.3 Engineering High-Index or Low-Coordination Surfaces

The rational construction of HEA nanocrystals with high-index facets or low-coordination surface atoms represents one of the most challenging yet rewarding goals in catalysis. These surfaces, enriched with atomic steps, kinks, and terraces, offer abundant low-coordination sites that capable of lowering the activation barriers to bond cleavage and formation, thereby boosting intrinsic catalytic activity. However, the creation and stabilization of such surfaces on multicomponent systems such as HEA nanocrystals are complicated by the competing influences of thermodynamics and kinetics during the growth process. For methods such as thermal annealing or carbothermal shock, the elevated temperatures involved facilitate extensive atomic diffusion that promotes solid-solution formation. However, these conditions simultaneously drive the system toward thermodynamic equilibrium, favoring the evolution of smooth, low-index facets that minimize surface free energy. Conversely, wet-chemical synthesis conducted under kinetic control and relatively low temperatures (typically, below 200 °C) can produce well-defined morphologies with controlled facet exposure. However, limited diffusion under such conditions restricts the degree of atomic mixing, especially in systems composed of metals with markedly different reduction potentials. This kinetic imbalance often results in incomplete alloying or even phase segregation, giving rise to spatially heterogeneous compositions rather than the desired atomically mixed solid solution.

The synthesis of HEA nanocrystals with high-index facets is further complicated by the inherently high surface energies of these crystallographic planes. High-index surfaces such as {210}, {310}, {320}, {510}, or {720} tend to reconstruct or relax to low-index configurations unless they are kinetically stabilized during the growth process. Achieving both atomic-level compositional uniformity and exposure of high-index surfaces therefore requires a precise control over the reduction, nucleation, growth, and diffusion processes. Recent advances have provided promising strategies to address this trade-off. For example, kinetic regulation through dropwise precursor addition enables control over both atomic deposition and diffusion rates, facilitating the formation of solid-solution dendritic HEA nanocrystals that expose multiple low-coordination sites.^78^ Another effective approach involves the use of concave nanocrystals as structural templates, followed by epitaxial deposition of HEA overlayers to form surfaces enriched in high-index or under-coordinated facets.^82,99^ Future studies should focus on understanding facet-dependent growth kinetics and exploring ligand- or solvent-mediated stabilization of high-energy surfaces, opening the door to maximizing catalytic performance.

### 7.4 Tailoring Unconventional Crystal Phases

Crystal phase engineering has recently emerged as a powerful means to modulate the intrinsic physicochemical properties of metal nanocrystals and consequently enhance their catalytic performance. Numerous studies on monometallic systems have demonstrated that unconventional crystal phases, such as FCC-Ru or HCP-Rh, can exhibit greater catalytic performance than their conventional counterparts in a variety of electrochemical reactions. Extending this concept to HEA nanocrystals provides new opportunities to fine-tune electronic structures, atomic arrangements, and lattice strain effects, thereby optimizing intrinsic catalytic properties through deliberate phase control. However, achieving unconventional crystal phases in HEA nanocrystals remains challenging. During synthesis, the metal species that reduce first typically form initial seeds whose crystal structure then dictates the phase of the final nanocrystal.^299^ For example, in platinum-group-metal HEA nanocrystals such as PdPtIrRhRu, the earlier reduction of Pd, Pt, Ir, and Rh precursors leads to the formation of seeds that adopt an FCC structure, which subsequently directs the growth of FCC-type HEA nanocrystals.^120^ The thermodynamic preference of these constituent elements for FCC structure further stabilizes this common phase, making it difficult to achieve alternative lattices such as HCP. Consequently, how to direct the formation of unconventional phases through synthetic control is a critical challenge in advancing HEA nanocrystal design.

Our recent work demonstrates a viable route to overcoming this limitation through controlled wet-chemical synthesis.^129^ By integrating dropwise precursor addition with seed-mediated epitaxial growth, we achieved the synthesis of RuRhPdPtIr HEA nanocrystals adopting an unconventional HCP phase. This was made possible by the layer-by-layer heteroepitaxial deposition of HEA atomic layers onto preformed HCP Ru seeds under finely tuned reduction kinetics (Figure 55). Despite the fact that four of the constituent metals (Rh, Pd, Pt, and Ir) are thermodynamically stable in an FCC lattice, the epitaxial registry with the HCP-Ru template and the nonequilibrium nature of the dropwise synthesis enabled the preservation of an HCP structure in the resulting HEA nanocrystals. This strategy provides a practical framework for directing lattice phase evolution through kinetic control rather than thermodynamic equilibrium.

**Figure 55:**
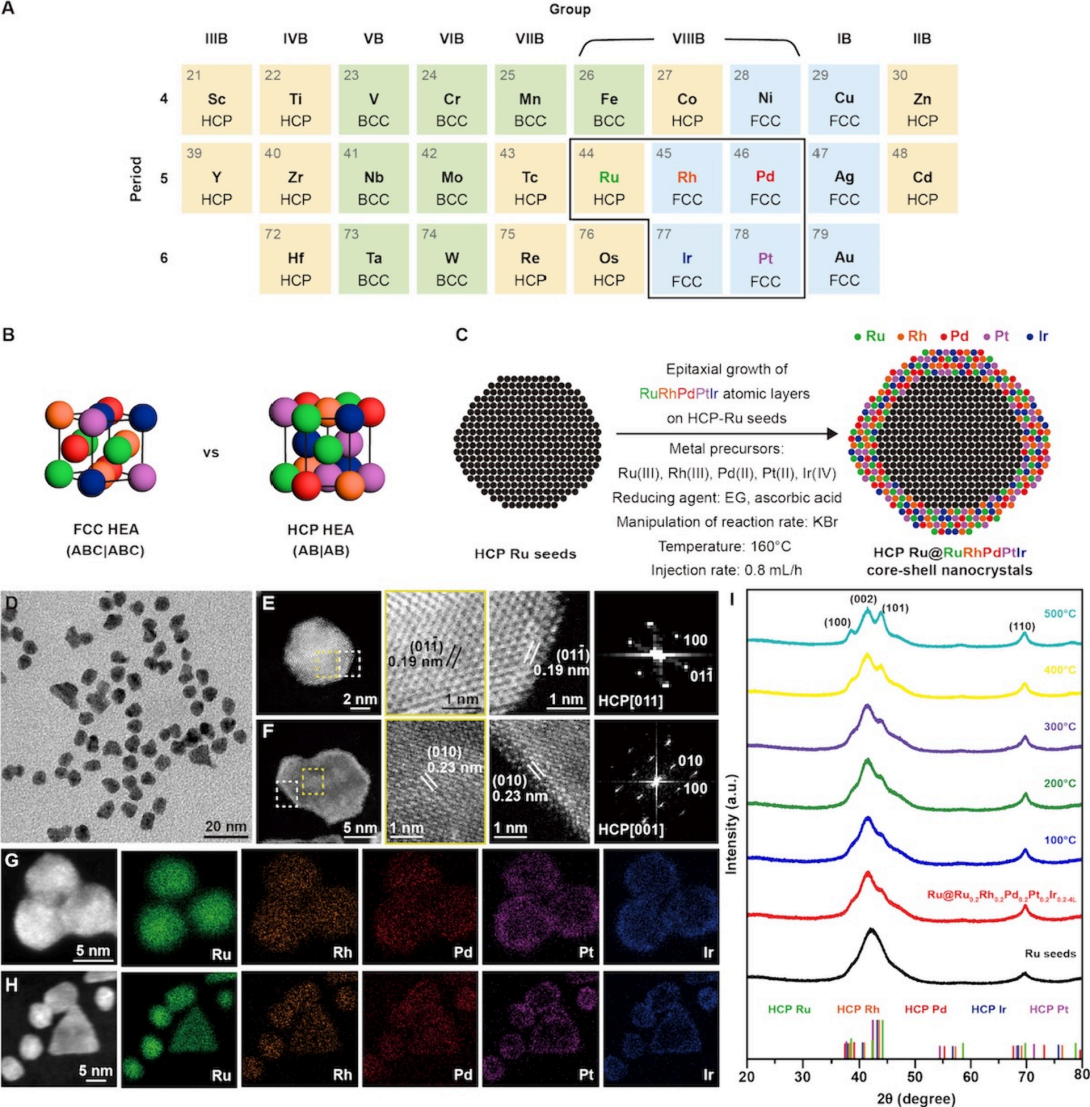
Achieving unconventional crystal phases in RuRhPdPtIr HEA nanocrystals. (A) Periodic table highlighting the crystal structures (FCC, HCP, and BCC) of transition metals commonly used in HEA synthesis. (B) Schematic comparison between FCC and HCP stacking sequences. (C) Schematic illustration of the seed-mediated epitaxial growth of RuRhPdPtIr HEA overlayers on HCP-Ru seeds. (D–F) TEM, HAADF-STEM images of Ru@RuRhPdPtIr core–shell nanocrystals, with atomic-resolution images and FFT patterns confirming the HCP lattice. (G–H) EDS elemental maps of HCP Ru@RuRhPdPtIr core–shell nanocrystals. (I) XRD patterns of the HCP-Ru@RuRhPdPtIr HEA core–shell nanocrystals annealed at different temperatures. Reproduced with permission from ref 129. Copyright 2024 Wiley-VCH.

In addition to epitaxial templating, unconventional phases may also be stabilized through purely kinetic pathways that suppress atomic rearrangement. Rapid reduction processes, such as laser or microwave-assisted synthesis, can quench metastable atomic configurations before structural relaxation occurs, yielding nonequilibrium crystal phases. Alternatively, chemical modulation of surface energy through selective ligand coordination or solvent–metal interactions could influence nucleation dynamics and favor alternative stacking sequences. Combining these kinetic approaches with theoretical modeling to predict phase stability under nonequilibrium conditions will further expand the accessible structural space of HEA nanocrystals. Overall, mastering phase control at the atomic level holds great promise for unlocking new catalytic paradigms by coupling compositional complexity with lattice engineering.

### 7.5 Mastering Surface Strain

Strain engineering has emerged as an effective approach to tailor the activity of metal catalysts, as even slight lattice deformation can markedly influence the electronic structure and binding strength of adsorbed intermediates. In general, compressive strain tends to weaken adsorption by lowering the metal *d*-band center, while tensile strain enhances adsorption through an upward shift of the electronic states. These variations directly modulate the reaction energetics and can lead to volcano-like or M-shaped dependency between catalytic activity and lattice strain. A notable example is the use of controlled strain in ultrathin Pt shells conformally grown on Pd nanocubes.^300^ Reversible lattice expansion and contraction induced by phosphorization and dephosphorization could generate a tunable strain field ranging from compressive to tensile. The resulting strain enabled fine adjustment of the catalytic performance of the Pt(100) surface, demonstrating clear strain–activity relationships in hydrogen evolution and methanol oxidation reactions. Such findings illustrate how precisely controlled strain can serve as a design parameter for optimizing electrocatalytic performance.

For HEA nanocrystals, strain control becomes even more intricate. Because multiple elements with distinct atomic sizes coexist within the same lattice, intrinsic lattice distortion gives rise to a spatially nonuniform strain distribution. This heterogeneity differentiates HEAs from single- or bimetallic catalysts and introduces complex local electronic environments that can either activate or inhibit surface reactions. Achieving a precise control over strain thus requires a good understanding of how atomic-size mismatch, elemental composition, and local coordination jointly affect surface energetics. Potential strategies include tuning elemental combinations to introduce desired lattice mismatches or employing structural templates that impose compressive or tensile deformation at the surface. Both approaches can adjust the balance between adsorption and desorption energies and enhance catalytic activity. Looking forward, elucidating the underlying mechanisms behind the strain–adsorption–activity relationship, through a combination of advanced characterization, theoretical modeling, and data-driven analysis, represents an essential step toward rationally designing HEA nanocrystals with optimized surface strain and superior catalytic performance.

### 7.6 Creating Hollow and/or Porous Structures to Increase Specific Surface Area

Hollow and porous metal nanostructures are highly attractive for catalysis because of their large surface-to-volume ratios, abundant accessible active sites, and efficient mass-transport pathways. These structural features significantly enhance the utilization efficiency of surface atoms and facilitate diffusion of reactants and intermediates during catalytic reactions. Translating these design principles into HEA nanocrystals, however, presents additional complexity because of the simultaneous involvement of multiple elements with different redox potentials and diffusion behaviors. Achieving precise control over both morphology and elemental distribution during the formation of hollow and/or porous architectures remains a major synthetic challenge.

Several strategies have been developed to construct porous HEA nanocrystals. One notable example involves the soft-templating approach, in which a diblock copolymer is used as a structure-directing agent to guide the coreduction and assembly of multiple metal precursors into ordered mesostructures. For instance, Yamauchi and co-workers reported a facile solution-based synthesis of mesoporous HEA nanospheres with tunable pore size and composition (Figure 56A–E).^301^ In their strategy, the diblock copolymer micelles serve as a soft template to organize multimetallic species during reduction, enabling cooperative nucleation and phase interdiffusion while preserving compositional homogeneity. The subsequent removal of the polymer template generates interconnected mesopores and a three-dimensional HEA framework with a high specific surface area. This approach effectively balances the kinetics of atomic diffusion and template-guided organization, providing a reliable route to fabricate compositionally uniform, mesoporous HEA nanocrystals.

**Figure 56:**
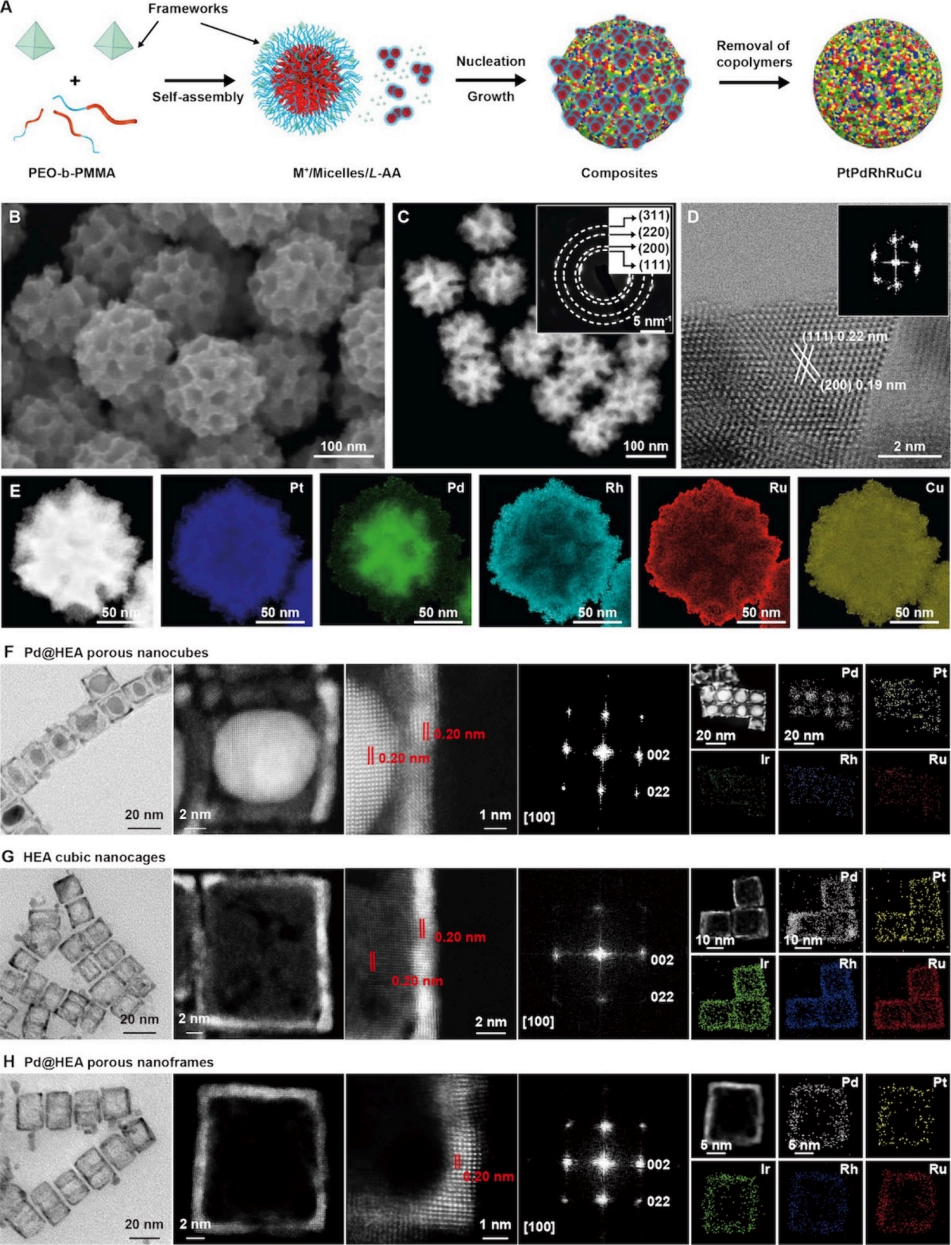
Hollow and/or porous HEA nanostructures synthesized via soft-templating and controlled etching strategies. (A) Schematic illustration of the PEO-*b*-PMMA micelle-directed soft-templating approach for synthesizing mesoporous PtPdRhRuCu HEA nanospheres. (B–E) SEM, HAADF-STEM, FFT, and EDS elemental maps of the PtPdRhRuCu HEA nanospheres. (F) TEM, HAADF-STEM, FFT, and EDS elemental maps of porous Pd@HEA nanocubes. (G) TEM, HAADF-STEM, FFT, and EDS elemental maps of HEA cubic nanocages. (H) TEM, HAADF-STEM, FFT, and EDS elemental maps of HEA cubic nanoframes. The images in (A–E) were reproduced with permission from ref 301. Copyright 2023 Springer Nature. The images in (F–H) were reproduced with permission from ref 133. Copyright 2024 Wiley-VCH.

An effective route to generating hollow or cage-like HEA nanostructures relies on galvanic replacement or controlled chemical etching. In these redox-driven reactions, the less noble elements are selectively oxidized and dissolved, while the more noble metals are deposited on the surface, gradually creating hollow interiors. The outcome of such processes can be tuned by adjusting parameters such as oxidant concentration, temperature, and/or reaction time. Nevertheless, achieving a uniform composition across the shell remains difficult because the coexistence of metals with different redox potentials often leads to uneven dissolution or redeposition, resulting in structural distortion and irregular facet exposure. To overcome these limitations, a kinetically regulated galvanic replacement strategy was recently developed to control both atomic mixing and surface structure in multicomponent systems.^205^ Through systematic modulation of precursor feeding sequences in the AgPdPtAu system, it was found that the order of ion introduction governed the balance between alloy homogeneity and facet stability, leading to the formation of uniform hollow AgPdPtAu nanocubes enclosed by {100} planes. On the other hand, by employing a Fe­(III)-mediated etching process, our group demonstrated that Pd@PdPtIrRhRu HEA core–shell nanocrystals could undergo sequential transformation into porous PdPtIrRhRu nanocubes, hollow PdPtIrRhRu nanocages, and ultimately into open PdPtIrRhRu nanoframes composed solely of atomic-scale ridges (Figure 56F–H).^133^ These findings emphasize that controlling the etching kinetics is essential for balancing dissolution and diffusion, thereby preserving structural integrity and compositional uniformity. Altogether, these strategies lay the groundwork for constructing hollow and porous HEA nanocrystals with enhanced specific surface areas, paving the way for rational catalyst design and improved reaction efficiency. Continued development of such approaches will enable the creation of a diverse library of HEA nanomaterials with tunable structures, compositions, and catalytic functionalities.

### 7.7 Elucidating the Impacts of Experimental Conditions

HEA nanocrystals, comprised of five or more principal elements, exhibit intrinsic chemical complexity that distinguishes them from traditional alloys. The complexity introduces a multitude of possible local configurations and coordination environments, which are highly sensitive to the conditions under which the materials are synthesized. Even if the overall composition remains nominally identical, variations in synthetic methods, each involving distinct thermodynamic and kinetic regimes, can lead to pronounced differences in local atomic arrangement, compositional homogeneity, and defect landscape. These differences, often imperceptible at the average structural level, can significantly influence the resulting physicochemical properties. Recent work has systematically explored this phenomenon by synthesizing a prototypical spinel-structured high-entropy oxides using five distinct synthetic approaches (Figure 57), including solid-state reaction, high-pressure treatment, hydrothermal crystallization, molten salt flux, and combustion synthesis.^302^ Despite the formation of materials with the same average crystal structure, these methods produced samples with marked discrepancies in their microstructures, cation distribution uniformity, and local coordination environments, as revealed using advanced synchrotron-based techniques such as XRD, XAS, and XRF microscopy. For instance, only the sample synthesized by combustion achieved near-ideal cation homogeneity, while other routes yielded compositions with evident microsegregation or clustering. These microstructural and local-scale distinctions translated into substantial differences in material functionality, as demonstrated by variations in their magnetic behaviors, including Curie temperature, coercivity, and saturation magnetization, despite that all samples retained the ferrimagnetic order.

**Figure 57:**
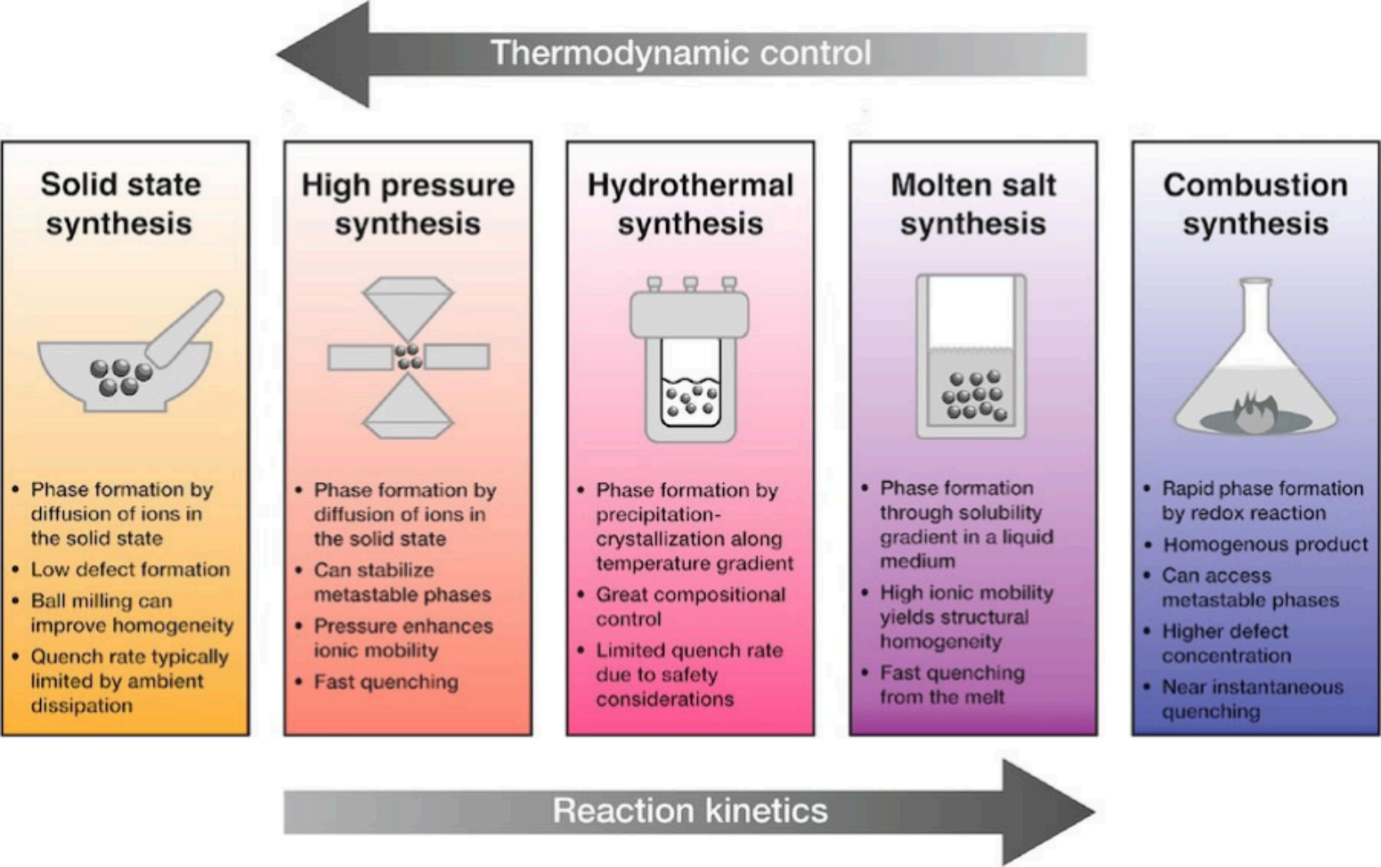
Influence of synthetic methodology on thermodynamic and kinetic controls in high-entropy oxide formation. Schematic illustration of the five synthetic approaches being compared, including solid-state reaction, high-pressure synthesis, hydrothermal crystallization, molten-salt synthesis, and combustion synthesis, arranged along a continuum from thermodynamic control to kinetic control. Reproduced with permission from ref 302. Copyright 2024 American Chemical Society.

These findings underscore the critical role of synthetic methodology in determining the functional landscape of HEA materials. The synthetic route does not merely dictate crystallinity or phase purity but serves as a fundamental axis of control over atomic-level uniformity, defect chemistry, and ultimately, macroscopic properties. Therefore, for HEA nanocrystals intended for catalytic or electronic applications, it is imperative to systematically investigate and optimize the synthetic parameters. Advanced characterization tools like XAS offer essential insights into the element-specific coordination environments and oxidation states that underpin performance. Establishing clear structure–property–synthesis correlations will be vital for developing the next-generation HEA-based materials with tailored functionality.

### 7.8 Understanding and Improving Structural and Compositional Stability

Maintaining the structural and compositional stability of HEA nanocrystals is crucial for long-term catalytic performance and is particularly important in industrial applications, where catalysts must operate reliably under harsh conditions for extended periods. Phase segregation, surface reconstruction, and ordering transition often occur at elevated temperatures or during electrochemical operation, leading to degradation in activity, selectivity, and catalyst lifetime. Understanding these transformations at the atomic scale is therefore essential for designing HEA nanocrystals with improved thermal and chemical robustness. When exposed to heat or reactive environments, atoms in HEA nanocrystals gain kinetic energy that facilitates diffusion and rearrangement into more thermodynamically stable configurations. Structural evolution generally proceeds through two sequential processes, with surface reconstruction occurring first because surface atoms, with lower coordination numbers, possess higher mobility. This is followed by bulk reconstruction involving atoms in the interior lattice, which diffuse more slowly due to their higher coordination numbers. Both processes can significantly alter the composition and structure, as observed in an early study.^303^ In HEA systems, such transformations are further complicated by differences in diffusion coefficients, bonding energies, and melting points among the constituent elements, resulting in intricate and often nonuniform transformation kinetics. Recently, *in situ* analyses by our group provided valuable insights into these dynamic processes. A stable FCC PtRuFeCoNi solid solution was maintained up to 200 °C, followed by the formation of L1_0_- and L1_2_-type intermetallics at 300–400 °C due to elemental interdiffusion. At 500 °C, phase separation occurred, indicating the metastability of the high-entropy configuration under thermal annealing.

While understanding these transformations offers critical mechanistic insights, improving structural stability requires strategies to suppress undesirable diffusion and segregation. Tailoring the elemental composition to minimize differences in atomic size, surface energy, and diffusion coefficients can effectively slow down interdiffusion and stabilize the high-entropy configuration. Incorporating refractory or high-melting-point elements, or introducing stronger interatomic interactions through selective alloying, are also promising approaches. Moreover, *in situ* spectroscopic and microscopic techniques combined with atomistic simulations should be used to elucidate kinetic barriers and diffusion pathways, enabling predictive control over phase evolution. Ultimately, establishing compositional guidelines for mitigating atomic diffusion and developing kinetic stabilization strategies will be key to achieving durable and compositionally uniform HEA nanocrystals for catalytic applications.

### 7.9 Developing High-Entropy Intermetallics (HEIs) with Ordered Phases

While HEAs are typically random solid solutions, introducing long-range atomic ordering through controlled intermetallic formation offers a new dimension in catalyst design. HEIs integrate the ordered lattice structure of conventional intermetallics with the compositional complexity of HEAs, combining configurational entropy stabilization with well-defined atomic sites. This unique hybrid structure enables a precise control over site-specific coordination, orbital hybridization, and charge localization, thereby providing a powerful handle to tune catalytic activity and selectivity.^304^ In contrast to solid-solution or phase-segregated HEAs, HEIs possess fixed atomic ratios and crystallographically ordered arrangements, which can yield uniformly distributed active sites, pronounced lattice distortion, sluggish diffusion, and distinct synergistic effects among constituent elements.

Recent advances have demonstrated the feasibility of synthesizing ordered HEIs at the nanoscale. Hu and co-workers successfully transformed disordered HEA nanocrystals into ordered multicomponent intermetallics containing up to eight elements (PtPdAuFeCoNiCuSn) through controlled thermal annealing, establishing a model system for elucidating the order–disorder transition and its influence on catalytic behavior (Figure 58).^305^ Likewise, structurally ordered PtIrFeCoCu HEI nanoparticles exhibited exceptional activity and durability toward both the ORR and H_2_/O_2_ fuel cell operation, underscoring the catalytic advantages of ordered multielemental lattices.^306^ More recently, Chen and co-workers further highlighted the potential of defect engineering within HEIs by synthesizing a PtZnFeCoNiCr intermetallic containing intentionally introduced Pt antisite defects, which achieved an outstanding mass activity of 4.12 A mg_Pt_^–1^ toward ORR, more than 30-fold higher than that of commercial Pt/C, together with excellent structural stability during accelerated cycling.^307^ Looking forward, achieving compositional homogeneity and facet control in HEI nanocrystals remains a formidable challenge due to competing thermodynamic and kinetic factors during multielement ordering. Developing synthetic routes that enable precise controls of atomic site occupancy, diffusion dynamics, and interfacial strain during ordering transitions will be critical. Furthermore, understanding the role of each element, whether as an active site, electronic modifier, or structural stabilizer, requires integration of advanced characterization techniques with first-principles modeling. Collectively, such efforts will establish the foundation for rationally designing high-entropy intermetallic catalysts with tailored structures, superior stability, and high intrinsic activity for diverse electrochemical and thermal catalytic applications.

**Figure 58:**
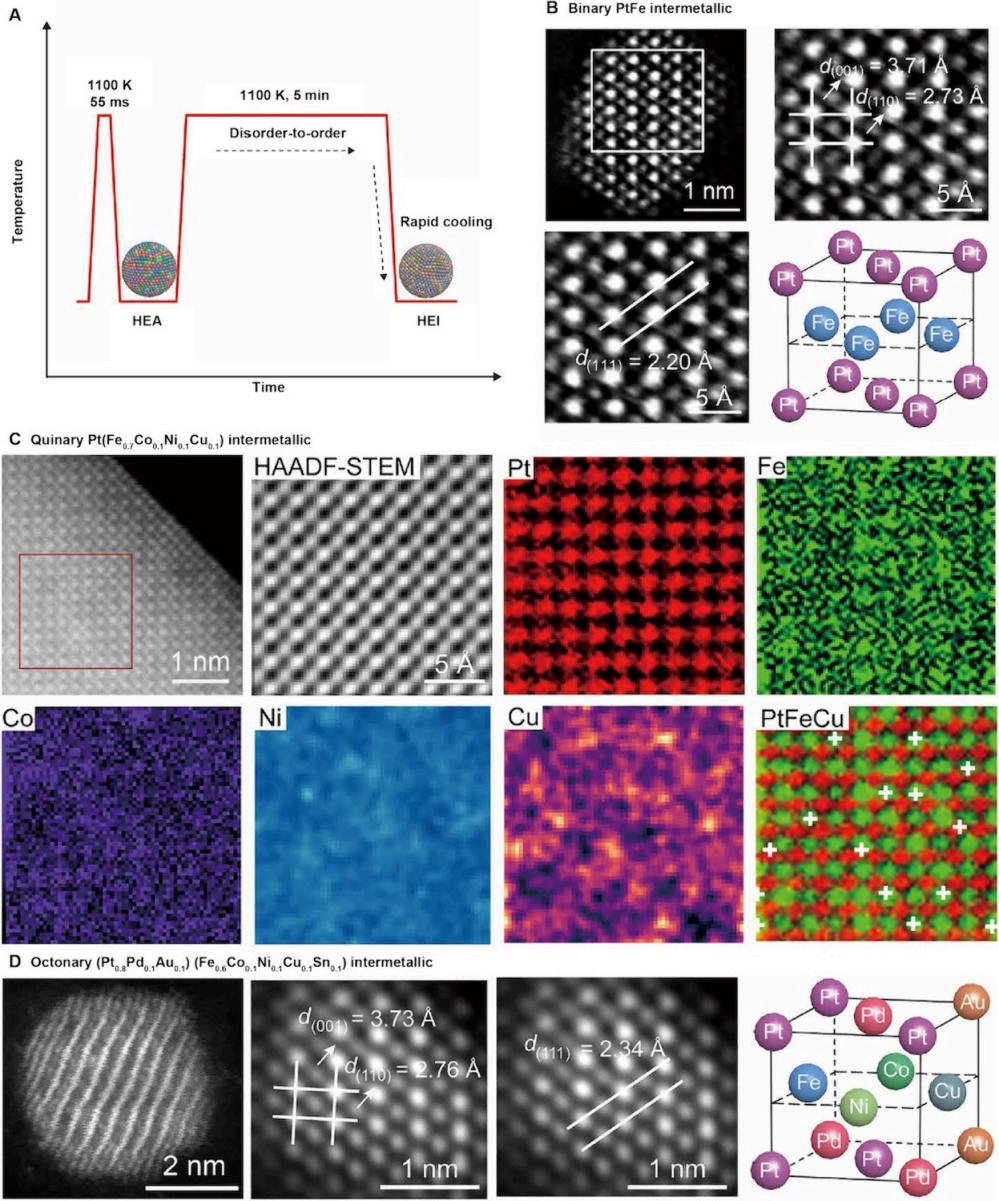
Synthesis and structural characterizations of ordered HEI nanocrystals. (A) Schematic of the Joule-heating-induced disorder-to-order transition used to convert disordered HEA nanoparticles into ordered HEIs. (B) HAADF-STEM image and atomic-resolution lattice structure of an ordered binary PtFe intermetallic nanoparticle. (C) HAADF-STEM image and corresponding atomic-resolution EDS elemental maps of a quinary Pt­(Fe_0.7_Co_0.1_Ni_0.1_Cu_0.1_) HEI nanocrystal. (D) HAADF-STEM image and lattice structure of an octonary (Pt_0.8_Pd_0.1_Au_0.1_)­(Fe_0.6_Co_0.1_Ni_0.1_Cu_0.1_Sn_0.1_) HEI nanocrystal. Reproduced with permission from ref 305. Copyright 2022 AAAS.

### 7.10 Probing Surface Atomic Configuration and Arrangement

Understanding the surface atomic arrangement and compositional depth distribution of HEA nanocrystals remains a formidable task. The expected discrepancies between bulk and surface compositions, along with the lack of analytical tools capable of resolving surface atoms at atomic precision, hinder the establishment of clear correlations between surface structure and catalytic performance. Moreover, probing the compositional depth profile and possible short-range order within HEAs is essential for elucidating the origin of their catalytic behavior.

As a state-of-the-art characterization technique, APT offers near-atomic spatial resolution and single-atom detection capability, providing a powerful means to reconstruct the 3D distribution of elements within a material. By sequentially evaporating and detecting individual atoms, APT enables the visualization of three-dimensional atomic arrangements and identification of local compositional heterogeneity, buried interfaces, and nanoscale precipitates that are inaccessible by conventional techniques. Its high sensitivity also allows quantitative detection of trace dopants and minor elements down to parts-per-million levels. Recent advances in APT have demonstrated its potential in mapping the nanoscale chemical complexity of HEAs with unprecedented precision.^210,308^ Extending this technique to HEA nanocatalysts could provide critical insights into the surface and subsurface atomic configurations that govern the catalytic activity, offering a promising pathway toward the rational correlation between structure, composition, and function. Moreover, theoretical calculations such as DFT can complement experimental observations by predicting the preferred atomic distributions and bonding tendencies of different elements at the surface or subsurface, providing fundamental insights into the compositional stability and catalytic origins of HEAs.

### 7.11 Identifying the Optimal Combination of Composition and Facet toward a Catalytic Reaction

The vast compositional and structural diversity of HEA nanocrystals provides an extraordinary platform for tailoring their catalytic properties.^309^ With multiple metallic elements that can be combined in various ratios and arranged on distinct crystallographic facets, HEAs offer an immense design space for optimizing active sites, adsorption energetics, and reaction pathways. To illustrate this tunability, we have achieved the synthesis of a large library of facet-controlled seed@HEA core–shell nanocrystals containing up to ten dissimilar metallic elements. By selecting Pd, Pt, Ir, Ru, Rh, Os, Au, Co, Fe, and Ni, elements commonly used in catalytic systems, 638 (C_5_^10^+C_6_^10^+C_7_^10^+C_8_^10^+C_9_^10^+C_10_^10^) distinct Pd@HEA and Cu@HEA nanocrystals with 5–10 elements in equimolar ratios can be obtained. Their stoichiometry within the HEA atomic layers is readily tunable, and the resulting nanocrystals can be engineered to preferentially expose well-defined facets such as {100} and {111}. This capability enables the systematic exploration of numerous composition-facet pairings for catalytic optimization. As an example, we identified that PtRuFeCoNi overlayers enclosed by {100} facets exhibit outstanding catalytic activity and durability toward HER, outperforming the commercial Pt/C catalyst. DFT analysis further indicates that the multielement cocktail effect, together with the {100} facet configuration, optimizes the hydrogen adsorption free energy (Δ*G*_H*_ = −0.01 eV), which explains the superior catalytic performance observed.

However, even with the aid of DFT calculations, exploring the vast combinatorial chemical space of HEAs remains a time-consuming process. The integration of data-driven discovery provides a powerful path forward. Recent advances in artificial intelligence and machine learning now allow for efficient navigation through complex compositional landscapes, accelerating the identification of active and durable catalysts.^232,310^ For example, the Closed-Loop Rapid Electrocatalyst Screening platform was used to evaluate more than 900 catalyst chemistries and 3,500 electrochemical tests within three months, identifying an octonary PdPtCuAuIrCeNbCr catalyst with a 9.3-fold improvement in cost-specific performance for electrochemical formate oxidation.^232^ Collectively, these results highlight the enormous potential of coupling compositional and facet tunability with data-driven approaches to accelerate the rational design of the next-generation HEA nanocatalysts.

### 7.12 Regulating Multivalent Oxidation States for Catalytic Enhancement

In HEA nanocatalysts, the coexistence of multiple oxidation states among constituent elements introduces an additional degree of freedom for tailoring surface reactivity. Even with the same elemental composition, variations in oxidation state can markedly influence the electronic structure, adsorption behavior, and catalytic pathway. Systematically investigating how different oxidation states affect catalytic activity is therefore essential for understanding and optimizing the intrinsic functionality of HEA surfaces. In the case of PtRuFeCoNi HEA systems, for example, XPS analysis reveals that Pt and Ru predominantly exist in metallic states, whereas Fe, Co, and Ni exhibit higher oxidation states due to their greater tendency toward oxidation under ambient conditions. This heterogeneity in oxidation state has been widely observed across HEA catalysts.^311^ Recent studies using *in situ* gas-cell TEM have further elucidated the oxidation kinetics of HEA nanocrystals (Figure 59). Initially, the HEA nanocrystals displayed atomic-level homogeneous mixing at room temperature; however, upon heating to 400 °C in air, outward diffusion of transition metals such as Fe, Co, Ni, and Cu led to the formation of an oxide layer enriched with these elements. The process is governed by the Kirkendall effect, which creates a composition gradient across the oxide shell. During oxidation, transition metals diffuse outward to form a disordered oxide layer with local ordering, while the FCC HEA core gradually shrinks and less reactive elements such as Pt remain metallic. This oxide layer acts as a diffusion barrier, leading to logarithmic oxidation kinetics and highlighting the distinct oxidation and diffusion behaviors of the constituent elements in HEAs.

**Figure 59:**
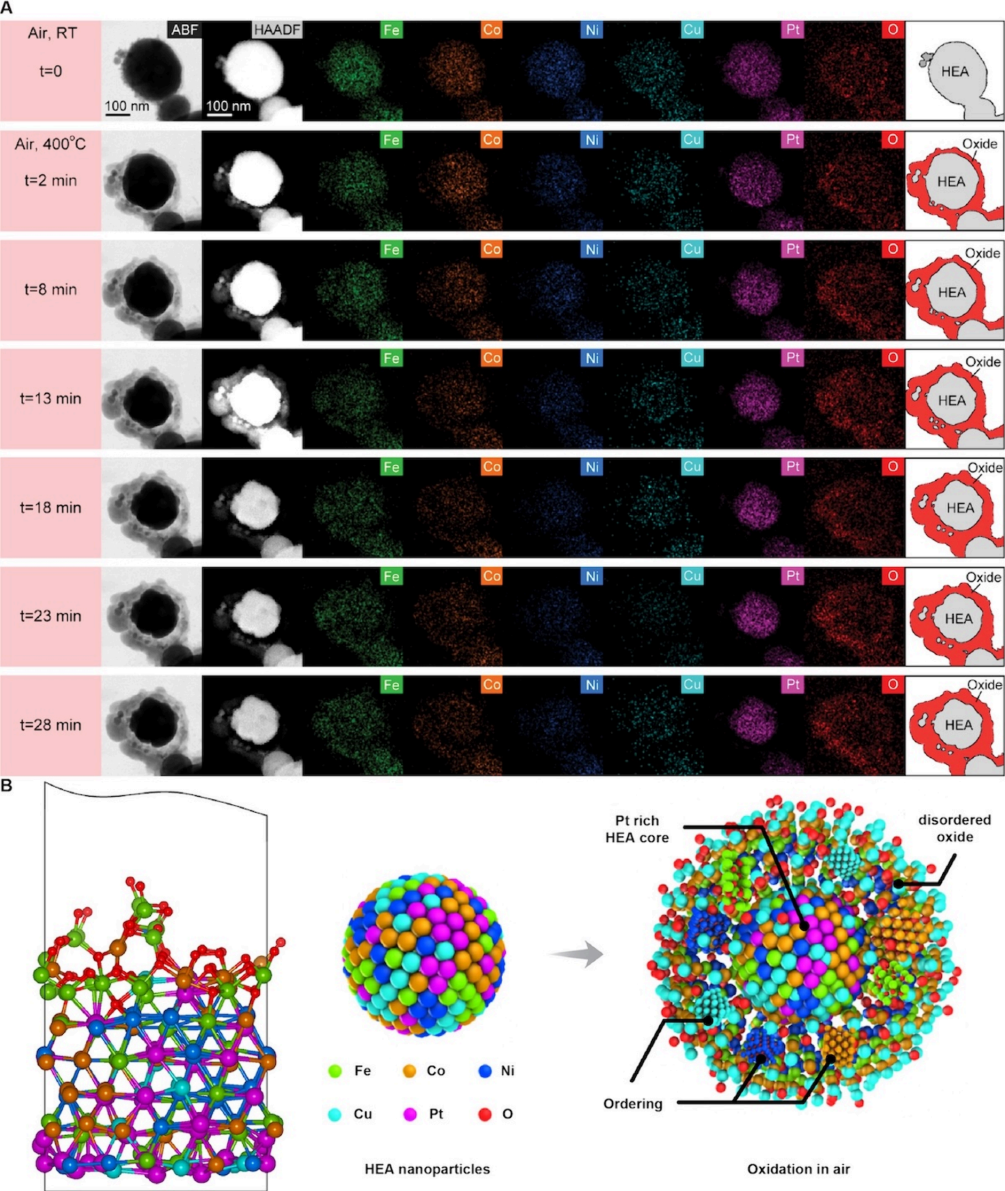
*In situ* oxidation dynamics of FeCoNiCuPt HEA nanoparticles. (A) Time-resolved *in situ* annular bright-field (ABF) and HAADF imaging with corresponding EDS elemental maps (Fe, Co, Ni, Cu, Pt, O) of HEA nanocrystals during annealing in air at 400 °C. Schematics on the right side highlight the evolution of the oxide layer extracted from ABF images. (B) Atomistic model and schematic illustration of the oxidation process, depicting selective outward migration of Fe/Co/Ni/Cu, retention of Pt in the metallic core, and the development of a compositionally graded, partially ordered oxide shell. Reproduced with permission from ref 311. Copyright 2020 American Chemical Society.

Comparative studies show that HEA surfaces with different oxidation states exhibit markedly different catalytic behaviors. Metallic surfaces favor conductivity and charge transfer whereas partially oxidized surfaces can provide beneficial metal–oxide synergy and heavily oxidized surfaces often suffer from reduced conductivity and overly strong adsorption. Optimal performance has therefore be achieved through a balanced interplay between metallic and oxidized regions. Future research should aim to establish quantitative correlations between oxidation-state distribution and catalytic performance across various HEA systems. Integrating operando techniques such as temperature-programmed oxidation (TPO), XPS, and related spectroscopic or microscopic analyses with theoretical modeling will enable precise identification of oxidation states and their dynamic evolution under reaction conditions. Through controlled oxidation treatments or redox cycling, it may become possible to selectively stabilize desired valence states or to form amorphous and crystalline oxide phases with targeted functionalities. Such controlled regulation of oxidation state represents a powerful strategy for fine-tuning surface electronic structures and optimizing catalytic performance in multielement systems.

### 7.13 Establishing Rational Design Principles for Low-Noble-Metal and Noble-Metal-Free HEA Catalysts

Developing HEA catalysts with reduced or no noble metal content without compromising catalytic performance has become an urgent objective to address both economic and sustainability challenges. This issue is especially critical for industrial applications, where catalyst cost, elemental abundance, and long-term supply stability are key factors in governing the practicality and scalability of large-scale implementation. The Sabatier principle, a cornerstone in heterogeneous catalysis, highlights the importance of achieving an optimal balance between adsorption and desorption of reaction intermediates. If intermediates bind too strongly, product desorption is hindered, whereas overly weak binding suppresses reactant activation. This balance is often illustrated by volcano plots, which serve as valuable design maps linking catalytic activity with adsorption energetics. Taking acidic HER as a typical example, the Δ*G*_H*_ is a key descriptor of catalytic activity, with optimal performance achieved when Δ*G*_H*_ approaches zero. PGMs such as Pt and Ir fall near this ideal range, placing them at the top of the HER volcano plot and accounting for their superior activity. However, their high cost and limited availability hinder widespread use. Alloying PGMs with non-noble metals offers a promising strategy to lower noble metal content while retaining activity. Yet, many non-noble metals either bind hydrogen too strongly (e.g., Mo, Fe, Co) or too weakly (e.g., Cu, Al, Bi), making it difficult to achieve the optimal adsorption–desorption balance required for efficient HER catalysis.^312^

Although numerous studies have reported promising low- or non-noble-metal HEA catalysts, these developments remain largely empirical, lacking a robust theoretical framework to rationalize and predict performance trends across diverse catalytic reactions. Unlike conventional monometallic or bimetallic catalysts, HEAs exhibit a broad distribution of active sites with distinct local coordination environments. Consequently, their adsorption energies follow a Gaussian-like distribution rather than a single characteristic value, reflecting the inherent heterogeneity of multielement surfaces.^33,229^ This complexity renders the conventional Sabatier framework insufficient for accurately describing the catalytic behaviors of HEA nanocatalysts. To overcome this limitation, it is essential to establish an extended theoretical framework that incorporates the statistical distribution of adsorption energies and multielement interactions intrinsic to HEA surfaces. By integrating DFT-based energetics with machine learning or descriptor-based models, one can construct quantitative correlations among composition, atomic configuration, and catalytic reactivity. Such a generalized theoretical approach would not only enable predictive optimization of low-noble-metal or noble-metal-free HEA catalysts but also provide transferable design principles applicable to a broad spectrum of catalytic reactions, including hydrogen evolution, oxidation, and carbon-based transformations, thereby paving the way for cost-effective, compositionally tunable, and high-performance catalytic systems.

### 7.14 Elucidating Catalytic Mechanisms and Element-Specific Roles

Understanding the mechanisms of catalytic reactions is essential for rational design, particularly in complex HEA systems where multiple elements contribute to the overall activity in distinct yet interconnected ways. A significant knowledge gap remains regarding how the synergistic interactions among constituent elements enhance catalytic performance and how these elements individually or collectively participate in the catalytic process. To bridge this gap, in-depth investigation of the electronic and structural dynamics of HEA catalysts under working conditions is required. Leveraging advanced characterization tools such as XAS, XPS, Raman spectroscopy, and *in situ* or operando techniques enables detailed examination of the catalytic mechanism and clarification of element-specific roles. These methods provide insights into chemical states, coordination environments, and adsorbate–metal interactions, revealing how each element contributes to adsorption, activation, and product formation. However, despite remarkable progress, careful interpretation of such data is still essential, especially for multicomponent HEA systems. In many cases, signals attributed to specific elements bonding with reactants, intermediates, or products may represent adsorption events rather than real active sites, as transient reactions are difficult to capture in real time.

The coexistence of diverse adsorption environments on the multimetallic surface likely calls for a synergistic mechanism for the catalysis. Strong-binding sites may serve as primary adsorption centers that help anchor key intermediates, whereas bridge or weak-binding sites comprised of mixed-affinity metals provide energetically favorable pathways for subsequent reaction steps.^33,313^ This variation in adsorption strength enables intermediates to migrate across HEA surfaces and promotes cooperative catalysis by multiple site ensembles rather than a single active site. As a result, HEA catalytic kinetics are more complex than those of monometallic systems, arising from the interplay of diverse atomic environments. In addition, some elements may contribute indirectly by tuning the electronic or geometric properties of neighboring sites. Clarifying these roles remains a major challenge and is essential for the rational design of HEA nanocrystals with optimized catalytic performance.

### 7.15 Tuning Adsorption Energies of Multiple Intermediates for Multistep Catalysis

One of the distinctive advantages of HEA nanocrystals lies in their capability to simultaneously modulate the adsorption energies of multiple reaction intermediates, thereby enabling efficient control over complex, multistep catalytic processes. For conventional mono- or bimetallic catalysts, optimization is often limited by a single descriptor, typically the binding strength of one key intermediate, which constrains the overall reaction rate by forcing a compromise between individual steps. In contrast, the compositional diversity of HEAs generates a broad spectrum of adsorption sites with varying affinities, allowing different intermediates to preferentially interact with specific surface regions. This multisite characteristic provides intrinsic flexibility to balance the energetics of sequential reaction steps, lowering activation barriers, and facilitating continuous conversion along the catalytic pathway. The ability to decouple the energetics of distinct intermediates is particularly beneficial for reactions involving multiple electron transfer steps, such as HER, CO_2_RR, and various oxidation reactions including MOR or EOR. Both theoretical and operando spectroscopic analyses suggest that such selective adsorption tuning originates from local variations in electronic structure and charge redistribution among neighboring elements within the HEA framework.

For example, in our recent investigation of the alkaline HER, operando XAS combined with DFT analysis revealed that the adsorption strengths of H* and OH* intermediates could be finely adjusted within an HCP HEA structure. Incorporating Ru, Rh, and Pd atoms into an active Pt–Ir matrix moderately weakened the H* binding on Pt sites while strengthening the OH* binding on Ir sites, achieving an optimal energetic balance. Operando XAS further confirmed electron accumulation at Pt sites and electron depletion at Ir sites under reaction conditions, consistent with preferential adsorption of H* and OH* on these respective sites. Complementary hydrogen underpotential desorption (HUPD) and CO-stripping analyses corroborated these results, confirming that the tunable adsorption energetics arose from multielement synergy. These findings highlight the importance of engineering HEA catalysts with tailored local electronic structures and balanced intermediate binding strengths to achieve efficient and selective multistep catalysis. While demonstrated here for the alkaline HER, the same principle can be extended to other complex electrocatalytic reactions, such as CO_2_RR and alcohol oxidation, providing a general framework for the rational design of next-generation catalysts with enhanced activity and selectivity.

In summary, the past decade has witnessed remarkable advances in the synthesis, characterization, and theoretical modeling of HEA nanocrystals, firmly establishing them as a transformative frontier in catalytic materials research. The 15 key aspects discussed above collectively underscore that realizing the full potential of HEA catalysts requires the synergistic integration of precise synthetic control, advanced operando characterization, and predictive theoretical modeling. From achieving uniform atomic mixing and mastering surface strain to tailoring oxidation states, crystal phases, and multistep reaction energetics, each direction contributes to a deeper and more holistic understanding of how compositional complexity governs catalytic performance.

Looking ahead, the convergence of data-driven discovery, machine learning-assisted design, and in situ spectroscopy will play pivotal roles in transforming empirical observations into a predictive framework for catalyst design. Equally important will be the development of scalable and sustainable synthetic routes that can translate laboratory discoveries into practical, industrially relevant catalysts. In this context, key challenges remain in achieving reproducible large-scale synthesis, maintaining long-term structural and compositional stability under operating conditions, reducing reliance on costly or scarce elements, and integrating HEA catalysts into practical device architectures. By integrating these interdisciplinary efforts, the next generation of HEA nanocatalysts will not only advance fundamental insight into multimetallic interactions but also accelerate the realization of efficient, durable, and economically viable technologies for clean energy conversion and chemical transformations.

## Abbreviations

0D: Zero-dimensional
1D: One-dimensional
2D: Two-dimensional
3D: Three-dimensional
4D: Four-dimensional
Δ*G*: Gibbs free energy
Δ*G*_*H**_: Hydrogen adsorption free energy
ΔH: Enthalpy of mixing
Δ*S*_*mix*_: Entropy of mixing
AA: Ascorbic acid
ABF: Annular bright-field
AET: Atomic electron tomography
AFM: Atomic force microscopy
AOR: Alcohol oxidation reaction
APT: Atom probe tomography
BCC: Body-centered cubic
BET: Brunauer–Emmett–Teller
BJH: Barrett–Joyner–Halenda
CE: Current efficiency
C_dl_: Double-layer capacitance
CF: Carbon fiber
CO_2_RR: CO_2_ reduction reaction
CSRO: Chemical short-range order
CTS: Carbothermal shock
CV: Cyclic voltammetry
DFT: Density functional theory
DMSO: Dimethyl sulfoxide
DOS: Density of states
ECSA: Electrochemical surface area
EDS: Energy-dispersive X-ray spectroscopy
EELS: Electron energy loss spectroscopy
EG: Ethylene glycol
EOR: Ethanol oxidation reaction
EXAFS: Extended X-ray absorption fine structure
FCC: Face-centered cubic
FCD: Full charge density
FE: Faradaic efficiency
FFT: Fast Fourier transform
FIB: Focused ion beam
FT: Fourier transform
GO: Graphene oxide
GOR: Glycerol oxidation reaction
HAADF: High-angle annular dark-field
HBE: Hydrogen binding energy
HCP: Hexagonal close-packed
HEA: High-entropy alloy
HEI: High-entropy intermetallic
HEMs: High-entropy materials
HER: Hydrogen evolution reaction
HOR: Hydrogen oxidation reaction
HRTEM: High-resolution transmission electron microscopy
HUPD: Hydrogen underpotential deposition
ICP-MS: Inductively coupled plasma mass spectrometry
ICP-OES: Inductively coupled plasma-optical emission spectrometry
IGMs: Iron-group metals
LDOS: Local density of states
LEIS: Low-energy ion scattering
LSV: Linear sweep voltammetry
MA: Mechanical alloying
MOR: Methanol oxidation reaction
NEB: Nudged elastic band
NMR: Nuclear magnetic resonance
NO_3_RR: Nitrate reduction reaction
NPs: Nanoparticles
NRR: Nitrogen reduction reaction
OCP: Open-circuit potential
OER: Oxygen evolution reaction
OHBE: Hydroxyl binding energy
OMC: Ordered mesoporous carbon
ORR: Oxygen reduction reaction
PDF: Pair distribution function
PGMs: Platinum-group metals
PVD: Physical vapor deposition
PVP: Poly­(vinylpyrrolidone)
R: Gas constant
RDS: Rate-determining step
rGO: Reduced graphene oxide
RHE: Reversible hydrogen electrode
SEM: Scanning electron microscopy
SHE: Standard hydrogen electrode
SNWs: Subnanowires
STEM: Scanning transmission electron microscopy
TEM: Transmission electron microscopy
TPR: Temperature-programmed reduction
TPO: Temperature-programmed oxidation
UPS: Ultraviolet photoelectron spectroscopy
WT: Wavelet-transform
XANES: X-ray absorption near-edge structure
XAS: X-ray absorption spectroscopy
XPS: X-ray photoelectron spectroscopy
XRD: X-ray diffraction
XRF: X-ray fluorescence
